# Metal‐Free Aryl Cross‐Coupling Directed by Traceless Linkers†

**DOI:** 10.1002/chem.201903582

**Published:** 2019-11-21

**Authors:** Veit G. Haensch, Toni Neuwirth, Johannes Steinmetzer, Florian Kloss, Rainer Beckert, Stefanie Gräfe, Stephan Kupfer, Christian Hertweck

**Affiliations:** ^1^ Department of Biomolecular Chemistry Leibniz Institute for Natural Product Research and Infection Biology, HKI Beutenbergstrasse 11a 07745 Jena Germany; ^2^ Institute for Physical Chemistry Friedrich Schiller University Jena Helmholtzweg 4 07743 Jena Germany; ^3^ Transfer Group Antiinfectives Leibniz Institute for Natural Product Research and Infection Biology (HKI) 07745 Jena Germany; ^4^ Institute for Organic and Macromolecular Chemistry (IOMC) Friedrich Schiller University Jena 07743 Jena Germany; ^5^ Chair of Natural Product Chemistry Friedrich Schiller University Jena 07743 Jena Germany

**Keywords:** biaryls, cross-coupling, density functional calculations, photochemistry, synthetic methods

## Abstract

The metal‐free, highly selective synthesis of biaryls poses a major challenge in organic synthesis. The scope and mechanism of a promising new approach to (hetero)biaryls by the photochemical fusion of aryl substituents tethered to a traceless sulfonamide linker (photosplicing) are reported. Interrogating photosplicing with varying reaction conditions and comparison of diverse synthetic probes (40 examples, including a suite of heterocycles) showed that the reaction has a surprisingly broad scope and involves neither metals nor radicals. Quantum chemical calculations revealed that the C−C bond is formed by an intramolecular photochemical process that involves an excited singlet state and traversal of a five‐membered transition state, and thus consistent *ipso*–*ipso* coupling results. These results demonstrate that photosplicing is a unique aryl cross‐coupling method in the excited state that can be applied to synthesize a broad range of biaryls.

## Graphical abstract

**Without a trace**: The scope and mechanism of (hetero)biaryl synthesis by photochemical fusion of aryl substituents tethered to a traceless sulfonamide linker (photosplicing) are reported. The reaction has a broad scope and involves neither metals nor radicals. Theoretical calculations revealed that the C−C bond is formed by an intramolecular photochemical process that is initiated by formation of a U‐shaped conformation, involves an excited singlet state and traversal of a five‐membered transition state, and thus consistently results in *ipso*–*ipso* coupling.

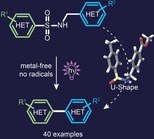

## Introduction

**Figure 1:**
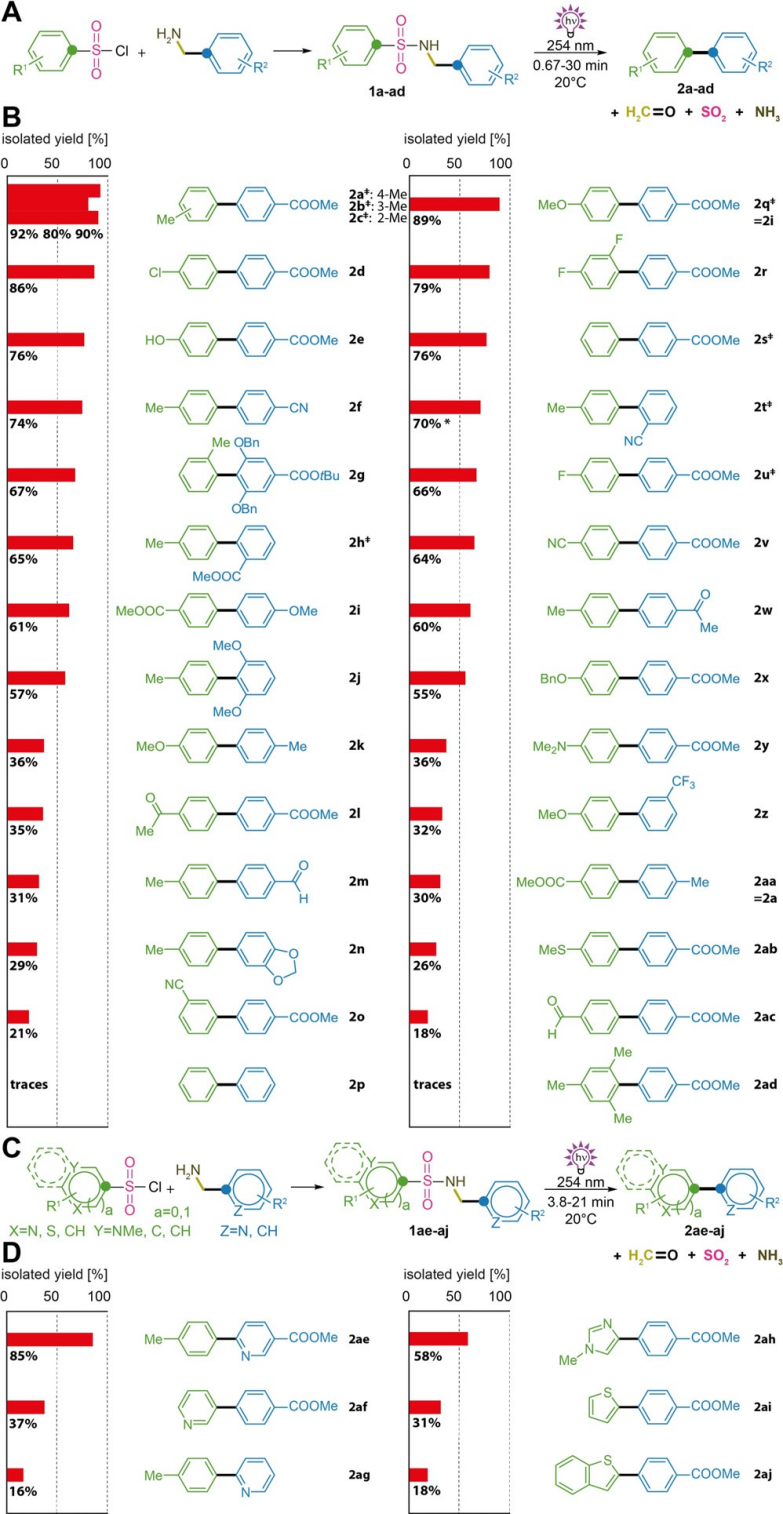
General scheme and scope of photosplicing. A) General reaction scheme. B) Biaryl target compounds and comparison of yields obtained by photosplicing. C) General reaction scheme for heterobiaryls. D) Heterobiaryl target compounds and comparison of yields obtained by photosplicing. *Based on recovered starting material. ^≠^Known example, included for comparison.

The enormous economic role of biaryls in chemical and pharmaceutical products^1^ has propelled the development of a large number of cross‐coupling methodologies.^2^ In general, metal catalysis offers high substrate tolerance and scalability of biaryl synthesis.^3^ Yet, in large‐scale manufacture and the production of bioactive ingredients, removal of heavy‐metal impurities, catalyst recycling, and the provision of suitable starting materials can be challenging and costly.^4^ Thus, there is high demand for alternative, metal‐free cross‐coupling methods.^5^ Most known metal‐free approaches, however, suffer from disadvantages such as narrow scope and low selectivity because of the harsh conditions required.5a Recent approaches to transition‐metal‐free biaryl syntheses involve, for example, Grignard reagents,^6^ oxidative coupling of electron‐rich arenes,^7^ base‐mediated coupling of aryl halides,^8^ and phosphorus ligand coupling.^9^ Furthermore, photolytic reactions have been employed to generate radicals or aryl radical cations,^10^ which set the stage for C−C bond formation. Regioselective biaryl syntheses have been achieved through intramolecular aryl coupling and rearrangement, yet parts of the linkers remain in the product.^11^ Recently, we found that bis‐aryl‐substituted sulfonamides can be contracted photochemically, and thereby permit the regioselective fusion of two aryl groups.^12^ Using a UV photoreactor, we demonstrated the preparative applicability of the reaction in the synthesis of building blocks for a series of clinically relevant pharmaceuticals. Remarkably, all required anchor groups to tether the aryls as sulfonamides are cleanly extruded as volatile fragments (ammonia, sulfur dioxide, formaldehyde) to yield the *ipso*–*ipso*‐coupled biaryl product (Figure 1 A). Yet, the scope and the molecular mechanism of this unusual photosplicing reaction have remained elusive. Herein, we address these important questions by a combination of chemical synthesis and quantum mechanical calculations, and present photosplicing as a highly versatile method for the selective, metal‐free preparation of biaryls.

## Results and Discussion

### Photosplicing permits the synthesis of a broad range of (hetero)biaryls

To test the scope of the photochemical aryl coupling, we prepared and compared a library of 30 sulfonamides with different substitution patterns and chemically diverse substituents at both aryl residues (Figure 1 B). We found that irradiation (254 nm) of *p*‐alkyl carboxylate‐substituted starting materials gives the best results, in particular when this substituent is located at the benzylic residue (blue). In lieu of a carboxyester, various electron‐withdrawing groups (nitrile, trifluoromethyl, acyl, and formyl) can be employed. Likewise, electron‐releasing substituents (methoxyl, benzyloxy, methyl) afford the desired products, albeit in reduced yields.

As to the benzenesulfonyl residue (green), photosplicing tolerates methyl, ethyl, methoxyl, benzyloxy, hydroxyl, fluoro, chloro, nitrile, carboxymethyl, acetyl, dimethylamino, methyl thioether, and trifluoromethyl moieties, except for photolabile groups such as nitro moieties. Photosplicing is even possible in the presence of two *ortho*‐methoxyl or benzyloxy groups (**2 g**, **2 j**). In contrast, two *ortho*‐methyl groups (**2 ad**) and the lack of substituents (**2 p**) hamper the reaction and yield only traces of the corresponding biaryls. In some cases, when the irradiation of sulfonamides afforded low biaryl yields, diverse degradation products resulting from S−N cleavage, oxidative deamination and other oxidative processes could be observed. Overall, photosplicing proved to be highly versatile, as it tolerates a broad range of photostable substituents. Surprisingly, even substrates with reversed polarity, such as **1 i** and **1 aa**, afford the corresponding biaryls.

To evaluate the possibility of coupling heteroaromatic units we prepared a suite of sulfonamides with pyridines, imidazoles, thiophenes, and their benzannulated derivatives (Figure 1 C). Irradiation with UV light (254 nm) led to the expected photosplicing products in moderate to excellent yields (Figure 1 D). The best results were obtained with pyridine and imidazole moieties. These findings illustrate the remarkably broad substrate scope for photosplicing.

### Experimental evidence for metal‐ and radical‐free reaction

**Figure 2:**
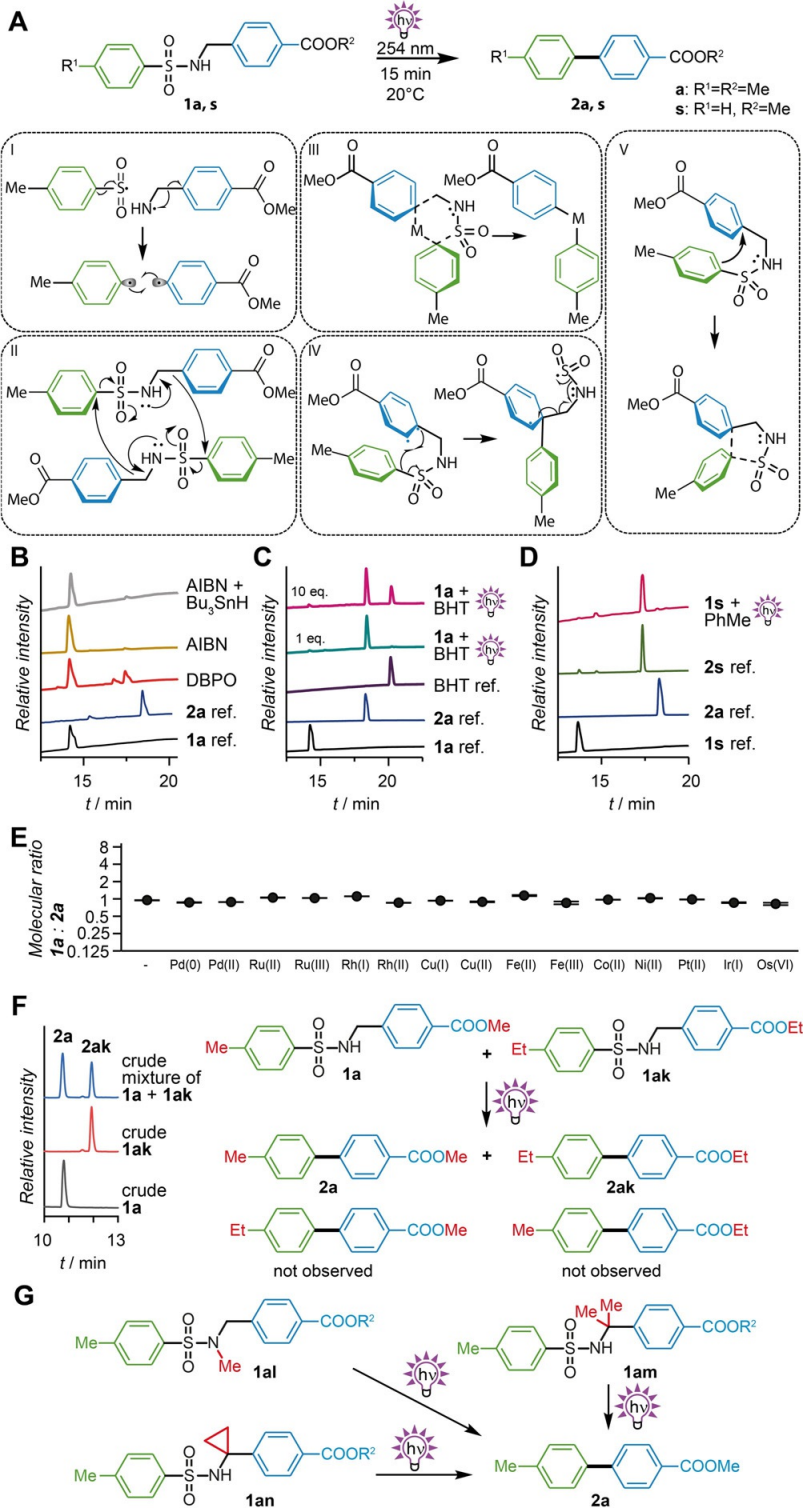
Evaluation of potential reaction mechanisms. A) General reaction scheme and different mechanistic scenarios for the photosplicing process. I) radical, II) intermolecular, III) metal‐catalyzed, IV) Smiles‐like rearrangement, V) regiochemically controlled intramolecular reaction. B) Impact of radical initiators (≈1 equiv of radical initiator with **1 a** in benzene at 70 °C for 25 h). C) Radical quenchers (1 equiv or 10 equiv BHT with **1 a** in MeOH, 254 nm, 15 min, 20 °C). D) Reactive solvent (**1 a** in MeCN/toluene (v/v=50:50), 254 nm, 15 min, 20 °C). E) Addition of metals (5 mol % or saturated solution of metal salts with **1 a** in MeOH, 254 nm, 15 min, 20 °C). F) Cross‐reactivity. G) Modification of linkers to hamper tautomer formation and to install a radical trap.

For the photoactivated aryl coupling, various reaction mechanisms are conceivable. UV excitation could potentially lead to homolytic fission of the linker followed by formation of carbon‐centered radicals and coupling of phenyl radicals (Figure 2 A, I),^13^ or the observed *ipso*–*ipso* substitution could involve intermolecular reactions favored by dispersive interactions (Figure 2 A, II).^14^ Although no metal additives were used, trace metal impurities in solvents and reagents could be sufficient to facilitate photocatalytic effects (Figure 2 A, III).^15^ Alternatively, the biaryl could be formed in analogy to a radical Smiles‐type rearrangement (Figure 2 A, IV).11b, 16 Another plausible route could involve an intramolecular *ipso*–*ipso* attack of the excited sulfonamide leading to a five‐membered transition state or intermediate, which undergoes further fragmentation (Figure 2 A, V).

To unravel whether UV irradiation of sulfonamide **1 a** afforded reactive aryl radicals in a Norrish type I reaction,^17^ first we meticulously analyzed the reaction mixture; yet, biaryls that would arise from random radical recombination processes could not be detected. Furthermore, neither radical starters such as dibenzoyl peroxide (DBPO) or azobisisobutyronitrile (AIBN), nor a mixture of tributyltin hydride and AIBN, which is used for the radical Smiles rearrangement,11a initiated biaryl coupling (Figure 2 B). Moreover, we found that the course of the photoreaction is not affected by the presence of the radical quencher 2,6‐di‐*tert*‐butyl‐4‐methylphenol (BHT),^18^ even if it is added in large excess (Figure 2 C). To trap potential intermediate radicals, an excess of toluene was added to the photoreaction with sulfonamide **1 s**. Even with toluene as cosolvent in acetonitrile (v/v=50:50), no side products of the coupling reaction were detectable (Figure 2 D). Finally, we installed a cyclopropyl group, which serves as a radical trap,^19^ at the linker and noted that it does not hamper the photoreaction (Figure 2 G). Thus, the involvement of (long‐lived) radicals in the course of the aryl coupling can be excluded.

To investigate the potential involvement of metal catalysts in the aryl coupling, we added different late d‐block metals (Fe, Co, Ni, Pd, Pt, Ir, Ru, Cu, Rh, Os) that are used for cross‐coupling reactions^20^ to sulfonamide **1 a**. In the absence of light, biphenyls were not formed, and after irradiation no increased ratio of sulfonamide to biphenyl could be observed (Figure 2 E). These findings provide strong support for a metal‐free reaction mechanism.

As an alternative to the templating effect of metals, we reasoned that the aromatic rings of the sulfonamide starting materials could be positioned by dispersive interactions. To address the possibility of potential intermolecular reactions, we prepared a pair of sulfonamides with complementary substitution and monitored the course of the photoreaction with a mixture of starting materials. The photoreaction of sulfonamides **1 a** and **1 ak** in one batch with the same molar ratio showed no photoproducts with mixed substituents (Figure 2 F). This cross‐reactivity experiment is in line with an intramolecular recombination. Consequently, the linker plays a key role in the aryl coupling.

To estimate the impact of *C*‐ and *N*‐substitutions in the linker and thus facilitation of the *ipso*‐carbon attack by tautomer formation, we replaced all protons by methyl groups (Figure 2 G). Therefore, we prepared an *N*‐methyl sulfonamide (**1 al**) and sulfonamides with two methyl groups at the carbon atom of the linker (**1 am**). As both substrates afforded the corresponding biphenyl (**2 a**), we concluded that the influence of potential tautomers on the reaction is negligible.

### Theoretical model of the reaction mechanism

To elucidate the mechanism underlying the photosplicing at a molecular level, we performed DFT and TDDFT calculations. As model reaction we investigated the light‐driven formation of biphenyl **2 a** from sulfonamides **1 a** and **1 aa** featuring inverse electronic structures. Initially, we calculated the potential energies of the initial states (**1 a** and **1 aa**) by GFN‐xTB,^21^ which permits a detailed analysis of the possible conformers in the ground state. The energies of selected conformers were verified by coupled cluster calculations^22^ and subsequently analyzed by means of a principal component analysis (Figures S9–S12 in the Supporting Information).

**Figure 3:**
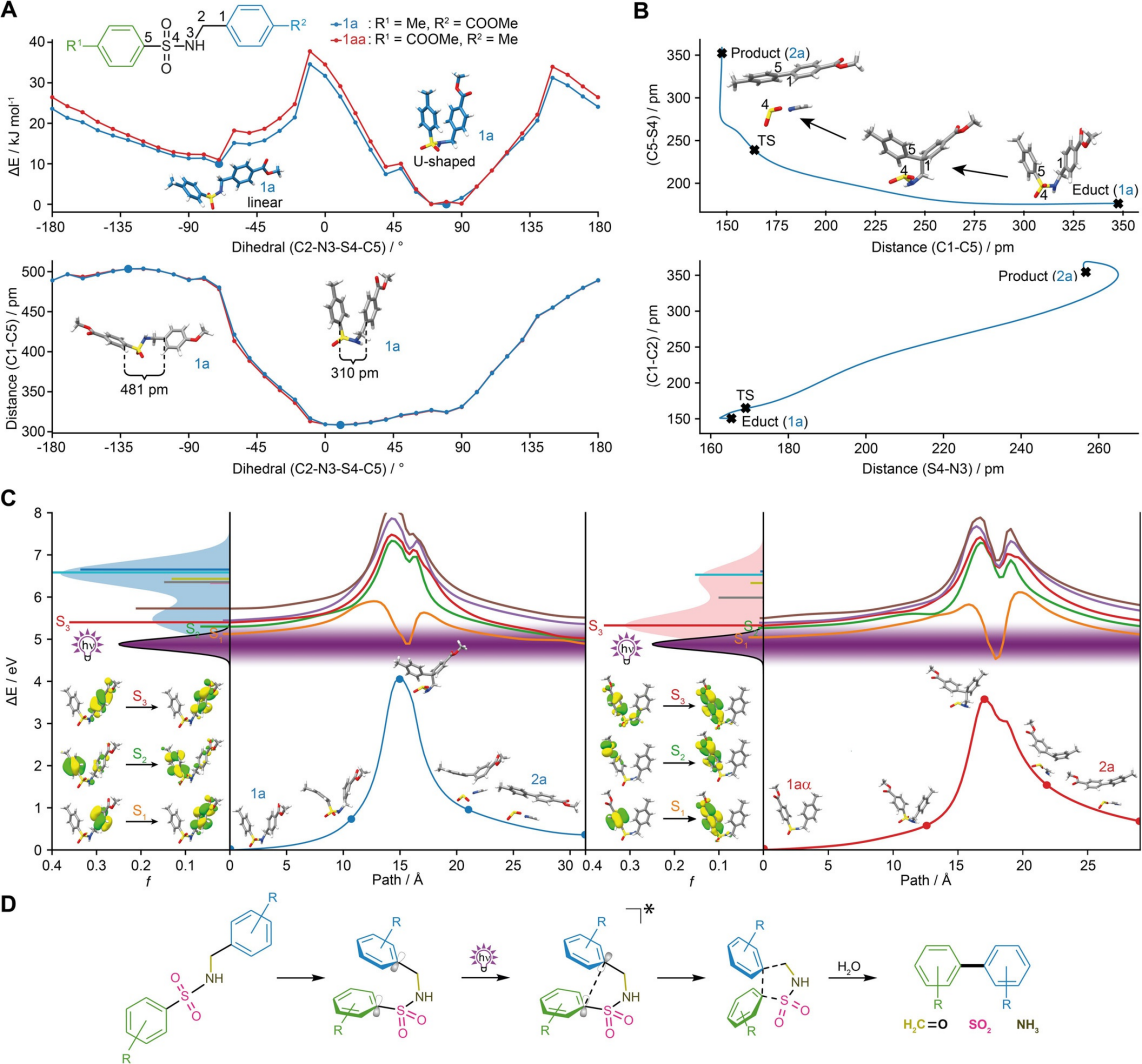
In silico analysis of photosplicing. A) Relaxed scan along the dihedral angle of the linker from sulfonamide **1 a** and **1 aa** with energy differences and C1−C5 distance. B) C1−C5 versus C5−S4 distances and S4−N3 versus C1−C2 distances during the photosplicing reaction. C) Reaction coordinate in the ground state and low‐lying excited (singlet) states of the photosplicing reaction with the calculated absorption spectra and the emission of the light source. D) Deduced model for the reaction mechanism of photosplicing.

Relaxed scans along the dihedral angle *φ* (given by C2‐N3‐S4‐C5) revealed that the flexible sulfonamide linker adopts preferably linear and U‐shaped horseshoe conformations (Figure 3 A). The relaxed potential‐energy curves (PECs) obtained by DFT (Figure 3 A) indicated that in **1 a** and **1 aa** the horseshoe conformer (*φ*=80°, *φ*=70° for **1 aa**) is energetically more favored (by 10 kJ mol^−1^) than the linear conformer (*φ*=−70°). The **1 a** horseshoe conformer exhibits a short C1−C5 distance (326 pm), which is pivotal for the C−C coupling of the aromatic rings.

To evaluate the potential course of the aryl coupling, we subsequently assessed the formation of photoproducts **2 a** and **2 aa** along a reaction pathway given by an intrinsic reaction coordinate (IRC). The IRC connects the starting and product states via a cyclic, C1‐C2‐N3‐S4‐C5‐containing transition state (TS) optimized by DFT. In the five‐membered TS of the reaction from **1 a** to **2 a** the C1−C5 distance decreases to 164 pm, which is associated with C−C bond formation. Simultaneously, partial cleavage of the C−S bond is indicated by the increase of the bond length from 176 to 239 pm (Figure 3 B). In stark contrast, changes within the linker are less pronounced until the TS is reached. Only on relaxation towards the product state, and thus formation of the photoproduct (**2 a**), are increased S−N and C1−C2 distances observed, indicating extrusion of SO_2_ and CH_2_NH, which can be hydrolyzed to ammonia and formaldehyde (Figure 3 B). According to our calculations, formation of the TS requires an activation energy of 4.08 eV in the ground state.

To gain insight into the excited states involved in the photoreaction, we first studied the low‐lying excited singlet states of **1 a** and **1 aa** in the Franck–Condon region. The simulated absorption spectrum of **1 a** (Figure 3 C) shows several bright states, that is, S_2_ at 5.30 eV (234 nm) and S_3_ at 5.40 eV (230 nm), in the vicinity of the irradiating light source centered at 4.88 eV (254 nm). Light‐driven coupling of the aryl moieties is feasible by the leading transition underlying S_2_ of **1 a**, which features a bonding character between C1 and C5 (Figure 3 C). In addition, the S_2_ state coincides with the experimentally determined maximum turnover at 5.28 eV (235 nm).^12^ Evolution of the adiabatic PECs of the low‐lying excited states reduces the energetic barrier to enable photosplicing of **1 a** in the excited states to only 0.69 eV along the IRC. It is notable that reversal of the substrate polarity (**1 aa** in lieu of **1 a**) leads to a congruent model and activation barrier (0.77 eV for S_1_).

To corroborate the quantum chemical simulations, we chose **1 p** as reference, since the bis‐phenyl‐substituted sulfonamide yields merely traces of the photoproduct (**2 p**). Compared to **1 a** and **1 aa**, we found that in **1 p** the determined excited states are shifted hypsochromically and have lowered oscillator strengths (Table S3 in the Supporting Information). Therefore, the disadvantageous excited‐state properties of **1 p** prevent an efficient photoreaction. The computational results are in full accordance with the experimental observations and thus allowed the mechanism of photosplicing to be elucidated.

Thus, our quantum chemical simulations show that electron‐withdrawing and electron‐donating groups are a prerequisite for the photoreaction, because these substituents control the energy of the excited states and determine the overlap with the excitation wavelength. It is noteworthy that the substitution pattern and thus polarity of the sulfonamide have no impact on the IRCs or energies of the excited‐state relaxation cascades.

The course of the photoreaction can be illustrated by means of the frontier orbitals of **1 a** (Figure S13 in the Supporting Information) contributing to S_2_. Specifically, the HOMO exhibits antibonding character between C1 and C5, whereas the LUMO shows bonding character between these two carbon atoms. The photoinduced population of the LUMO lowers the activation energy substantially and enables the formation of **2 a**. The small energy gap (1.0 eV) between the ground state and the excited state (S_1_) in the vicinity of the TS facilitates the relaxation into the product state.

## Conclusion

We have demonstrated that photosplicing is a highly versatile method for the selective synthesis of a broad range of biaryls. With 40 examples, we have shown that a large number of residues with different regiochemical orientation and even heteroaromatic rings are tolerated. Notably, photosplicing is an exceptional, traceless, *ipso*–*ipso*‐selective aryl cross‐coupling method that is truly metal‐free and, more importantly, independent of free‐radical formation. By combining synthesis and computational methods we have shed light on the mechanism of this novel photochemical reaction. It is initiated by the formation of a U‐shaped conformation that leads to a cyclic intermediary state. With only a few exceptions, the energy of a UV‐C light source is sufficiently high to achieve population of excited states, which drive the reaction towards the targeted biaryls. Beyond providing a general model for photosplicing, our results demonstrate that this highly selective and versatile sulfonamide contraction is an important addition to the current toolbox for metal‐free biaryl syntheses.

## Experimental Section

Experimental details are given in the Supporting Information.

## Conflict of interest

The authors declare no conflict of interest.

## Supporting information

As a service to our authors and readers, this journal provides supporting information supplied by the authors. Such materials are peer reviewed and may be re‐organized for online delivery, but are not copy‐edited or typeset. Technical support issues arising from supporting information (other than missing files) should be addressed to the authors.

Supplementary

Supplementary
