# Supplementary material for: Metal‐Free Aryl Cross‐Coupling Directed by Traceless Linkers
Source: Chemistry. 2019 Nov 21;25(70):16068–73. doi: 10.1002/chem.201903582 (PMC6973076; doi:10.1002/chem.201903582)
Supplement: Supplementary file 1 — Supplementary [file CHEM-25-16068-s001.pdf]

CHEMISTRY
A European Journal

Supporting Information

Metal-Free Aryl Cross-Coupling Directed by Traceless Linkers**

Veit G. Haensch+,[a] Toni Neuwirth+,[a] Johannes Steinmetzer+,[b] Florian Kloss,[c]
Rainer Beckert,[d] Stefanie Gräfe,[b] Stephan Kupfer,*[b] and Christian Hertweck*[a, e]

chem_201903582_sm_miscellaneous_information.pdf
chem_201903582_sm_Video.mp4

Table of Contents

Experimental Procedures.......3
General synthesis procedures.......3
General analytical procedures.......3
Effect of metals on photosplicing.......4
Procedures for radical studies.......5
Presence of radical quencher during the irradiation of sulfonamide **1a**.......5
Presence of radical starters with sulfonamide **1a**.......5
Excess of toluene during the photosplicing of sulfonamide **2s**.......6
Cross experiment.......6
Compound **1ak.**.......6
Compound **2ak.**.......6
Modification of the linker.......8
Compound **1al.**.......8
Compound **2a** (=**2al**).......8
Compound **1am.**.......8
Compound **2a** (=**2am**).......8
Compound **1an.**.......9
Compound **2a** (=**2an**).......9
Chemical synthesis procedures for SAR studies.......10
Compound **1d.**.......10
Compound **2d.**.......10
Compound **1e.**.......10
Compound **2e.**.......11
Compound **1f.**.......11
Compound **2f.**.......11
Compound **1g.**.......11
Compound **2g.**.......12
Compound **1i.**.......12
Compound **2i.**.......12
Compound **1j.**.......13
Compound **2j.**.......13
Compound **2k.**.......13
Compound **1l.**.......14
Compound **2l.**.......14
Compound **1ao.**.......14
Compound **1m.**.......14
Compound **2m.**.......15
Compound **1n.**.......15
Compound **2n**.......15
Compound **1o**.......16
Compound **2o**.......16
Compound **2p.**.......16
Compound **1r.**.......16
Compound **2r.**.......17
Compound **1v.**.......17
Compound **2v.**.......17
Compound **1w.**.......18
Compound **2w.**.......18

Compound **1x.** 18
Compound **2x.** 19
Compound **1y.** 19
Compound **2y.** 19
Compound **1z.** 20
Compound **2z.** 20
Compound **1aa.** 20
Compound **2a** (=**2aa**). 20
Compound **1ab.** 21
Compound **2ab.** 21
Compound **1ac.** 21
Compound **2ac.** 22
Compound **1ad.** 22
Compound **2ad.** 22
Compound **1ae.** 22
Compound **2ae.** 23
Compound **1af.** 23
Compound **2af.** 23
Compound **1ag.** 24
Compound **2ag.** 24
Compound **1ah.** 24
Compound **2ah.** 24
Compound **1ai.** 25
Compound **2ai.** 25
Compound **1aj.** 25
Compound **2aj.** 26
Computational details 27
Ground state calculations 28
Conformational analysis 28
Definition of internal coordinates for the PCA 28
Conformers of **1p** 29
Conformers of **1a** 30
Frontier molecular orbitals 34
Intrinsic Reaction Coordinate of **1p** 35
Intrinsic Reaction Coordinate of **1a** 35
Intrinsic Reaction Coordinate of **1aa** 36
Excited state calculations 37
Comparison between experimental and calculated spectrum 37
Nomenclature 38
TDDFT spectrum of **1p**, horseshoe 38
TDDFT spectrum of **1p**, linear 39
TDDFT spectrum of **1a**, horseshoe 41
TDDFT spectrum of **1a**, linear 42
Comparison of excited states along the IRC for **1a** and **1p** 43
TDDFT spectrum of **1aa**, horseshoe 43
TDDFT spectrum of **1aa**, linear 45
Video of the mechanism 46
References 47
^{1}H and ^{13}C NMR Spectra 48

Experimental Procedures

General synthesis procedures

All reagents were obtained from commercial suppliers (Sigma Aldrich, TCI, Alfa Aesar, etc.) and used without further purification unless otherwise explained. Reactions were carried out under inert gas (argon) by using the Schlenk technique in dried solvents. Dichloromethane (DCM), acetonitrile (MeCN), methanol (MeOH) and chloroform were used from a solvent purification system (Innovative Technologies). Open column chromatographic separations were executed on silica gel (Kieselgel 60, 15-40 µm, Merck KGaA). Reaction progresses were monitored by thin layer chromatography (TLC) (silica gel on aluminium sheets 20 × 20 cm with fluorescent indicator 254 nm, Merck KGaA), GC-MS or HPLC-(HR)MS. Photoreactions were performed on a self-made photo reactor made from a milled aluminum block (16.9 × 31.9 × 0.03 cm, reaction volume ~15 mL) covered with a quartz glass slide. A SIMDOS 02 dosing pump from KNF was attached to the photo reactor with FEP tubes. A Herolab UVT-40 S equipped with 6 tubes Philips TUV 15 W/G15 T8 (total power 90 W; 44 W/m^2 at 245-260 nm) was used as the UV light source to illuminate the samples from above. A cryostat attached to the aluminum block allowed the control of the temperature and a small container stopped any air bubbles from the pump from reaching the photo reactor. Flow rates (0.5-10 mL/min) were selected in the course of reaction optimization.

General analytical procedures

All 1D (^{1}H, ^{13}C, ^{19}F, DEPT) and 2D NMR (^{1}H-^{1}H COSY, HSQC, NOESY, HMBC) have been recorded in deuterated solvents on a Bruker AVANCE II 300, AVANCE III 500 or 600 MHz instrument equipped with Bruker Cryo Platform. The chemical shifts are reported in ppm relative to the solvent residual signal (^{1}H: δ ($CHCl_3$) = 7.26 ppm, δ (CH_2Cl_2) = 5.32 ppm, δ (MeOH) = 3.31 ppm, δ (DMSO) = 2.50 ppm, δ (MeCN) = 1.94 ppm. ^{13}C: δ ($CDCl_3$) = 77.16 ppm, δ (CD_2Cl_2) = 53.84 ppm, δ (MeOD) = 49.00 ppm, δ (DMSO-d6) = 39.52 ppm, δ (CD_3CN) = 1.32 ppm or 118.26 ppm. ^{19}F: δ ($PhCF_3$) = -62.61 ppm as an external standard).[1-2] Following abbreviations are used for multiplicities of resonance signals: s = singlet, d = doublet, t = triplet, q = quartet, qt = quintet, br = broad. Preparative HPLC purification was achieved by using Gilson Abimed device with Binary Pump 321 and Dual Wavelength Detector 156 (column: Phenomenex Luna C18, 10 µm, 250 × 21.2 mm, eluent: water, MeCN (10–100%)). Gas-chromatographic measurements were executed on Thermo Trace GC Ultra equipped with CombiPAL autosampler and coupled with FID and Thermo Polaris Q electron impact ion trap mass spectrometer. GC conditions: column SGE BPX5 30 m × 0.25 mm ID; carrier gas helium; split injection with split ratio 1:10 and injection volume 5 µL; 1.5 mL/min carrier gas flow; temperature profile 0–1 min: 40 °C, 1-3 min: heating up to 100 °C (30 °C/min), 3–28 min: heating up to 350 °C (10 °C/min). LC-MS measurements were performed using Exactive Orbitrap High Performance Benchtop LC-MS with electrospray ion source and Surveyor HPLC system (Thermo Fisher Scientific, Bremen), Q Exactive Orbitrap High Performance Benchtop LC-MS with electrospray ion source and Accela HPLC system (Thermo Fisher Scientific, Bremen), Q Exactive HF-X Orbitrap High Performance Benchtop LC-MS with electrospray ion source and Surveyor HPLC system (Thermo Fisher Scientific, Bremen), or LTQ Velos Ion Trap Benchtop LC-MS with electrospray ion source and Surveyor HPLC system (Thermo Fisher Scientific, Bremen). HPLC conditions using Exactive: C18 column (Thermo Fisher Betasil C18, 3 µm, 150 × 2.1 mm) and gradient elution (MeCN (0.1 % (*v*/*v*) HCOOH)/H_2O (0.1 % (*v*/*v*) HCOOH)) starting with 5:95 for 1 min, going up to 99:1 in 16 min, then 99:1 for 15 min; flow rate 0.2 mL/min; injection volume: 3 µL). HPLC conditions using Q Exactive: C18 column (Thermo Fisher Accucore C18, 2.6 µm, 100 × 2.1 mm) and gradient elution (MeCN (0.1 % (*v*/*v*) HCOOH)/H_2O (0.1 % (*v*/*v*) HCOOH)) starting with 5:95, going up to 98:2 in 10 min, then 98:2 for 12 min; flow rate 0.2 mL/min; injection volume: 3 µL). HPLC conditions using Q Exactive HF-X: C18 column (Phenomenex Kinetex C18, 1.7 µm, 50 × 2.1 mm) and gradient elution (MeCN (0.1 % (*v*/*v*) HCOOH)/H_2O (0.1 % (*v*/*v*) HCOOH)) starting with 5:95, goint to 0:100 in 4.5 min, then 0:100 for 2 min; flow rate 0.7 mL/min; injection volume: 2 µL). HPLC conditions using LTQ: C18 column (Phenomenex Kinetex XB-C18, 2.6 µm, 100 × 3 mm) and gradient elution (MeCN (0.1 % (*v*/*v*) HCOOH)/H_2O (0.1 % (*v*/*v*) HCOOH)) 10:90 for 1 min, going up to 100:0 in 8 min, then 100:0 for 4 min; flow rate 0.6 mL/min; injection volume: 5 µL) or (MeCN (0.1 % (*v*/*v*) HCOOH)/H_2O (0.1 % (*v*/*v*) HCOOH) 48:52 for 11 min; flow rate 0.6 mL/min; injection volume: 5 µL). HPLC conditions for regioisomer separation using Q Exactive: C18 column (Thermo Fisher Accucore C18, 2.6 µm, 100 × 2.1 mm) and isocratic elution (MeCN (0.1 % (*v*/*v*) HCOOH)/H_2O (0.1 % (*v*/*v*) HCOOH)) with 50:50 for 20 min, then 100:0 for 10 min; flow rate 0.2 mL/min; injection volume: 3 µL). UV-vis spectra were recorded with a UV-1800 UV-vis-spectrometer from Shimadzu using fused quartz glass cuvettes with 1 cm path length. The samples were measured at 20 °C in MeCN. The emission spectrum of the light source was recorded with a specbos 1211UV broadband radiometer from JETI Technische Instrumente GmbH. The spectral range is from 230 nm to 1000 nm, the optical bandwidth is 4.5 nm and the wavelength resolution is 1 nm.

Effect of metals on photosplicing

Methyl 4-(((4-methylphenyl)sulfonamido)methyl)benzoate (**1a**) (500.2 mg, 1.57 mmol) was dissolved in MeOH (250.6 mL). 10 mL of this solution were transferred into 18 tubes with ~5 mol% of different metal salts (see Table S1), mixed gently and stored at room temperature for 24 h. In some cases, the metal salts were not completely soluble. These samples were centrifuged to remove the solid. All samples were measured by HPLC-MS.
All samples were loaded on the photo reactor (reduced light intensity (only 2 Philips TUV 15 W tubes), 5 mL/min) and irradiated until 75 mL of the solution were collected. 2 µL of each 18 solutions were mixed with MeOH (98 µL) and were measured by HPLC as triplicates. The results are shown in Figure S1.

Table S1: Used additives with molar mass, amount in mg, mol% and solubility.

Number	Substance	Amount	Mol%	Solubility
1	No additive	-	-	-
2	$RuCl_2(PPh_3)_3$	3.036 mg	5.1	Not completely soluble
3	$RuCl_3$	0.637 mg	4.9	Good
4	$RhCl(PPh_3)_3$	2.926 mg	5.1	Not completely soluble
5	$Rh_2(OAc)_4$	0.791 mg	5.7	Good
6	$Cu(OAc)_2{\cdot}H_2O$	0.668 mg	5.3	Good
7	CuOAc	0.461 mg	6.0	Not completely soluble
8	$Co(OAc)_2$	0.567 mg	5.1	Good
9	$NiCl_2{\cdot}6H_2O$	0.514 mg	3.5	Good
10	$Fe(acac)_2$	0.796 mg	5.0	Good
11	$FeCl_3{\cdot}6H_2O$	0.865 mg	5.1	Good
12	$Pd(PPh_3)_4$	3.289 mg	4.5	Not completely soluble
13	$Pd(OAc)_2$	0.755 mg	5.4	Not completely soluble
14	$PtCl_2$	0.870 mg	5.2	Not completely soluble
15	IrClCOD	1.138 mg	5.4	Not completely soluble
16	K_2OsO_4	1.132 mg	4.9	Not completely soluble

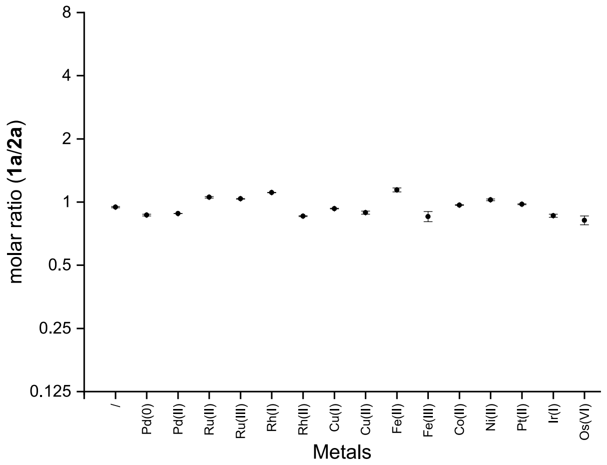

Figure S1. Ratio of sulfonamide **1a** and biphenyl **2a** after the photoreaction with different additives.

Procedures for radical studies

*Presence of radical quencher during the irradiation of sulfonamide **1a***

Sulfonamide **1a** (20.6 mg, 64.6 µmol, 1 eq.) and 2,6-di-*tert*-butyl-4-methylphenol (BHT, 14.3 mg, 64.9 µmol, 1 eq.) were dissolved in MeOH (4 mL). Sulfonamide **1a** (20.0 mg, 62.7 µmol, 1 eq.) and BHT (139.5 mg, 633.1 µmol, 10.1 eq.) were dissolved in MeOH (4 mL). BHT (50 mg, 227 µmol) was dissolved in MeOH (10 mL). The prepared solutions were loaded on the photo reactor with a flow rate of 1 mL/min and irradiated with UV light (254 nm) at room temperature. The crude products were analyzed by HPLC-HRMS in comparison with **1a** and **2a** and the results are shown in Figure S2.

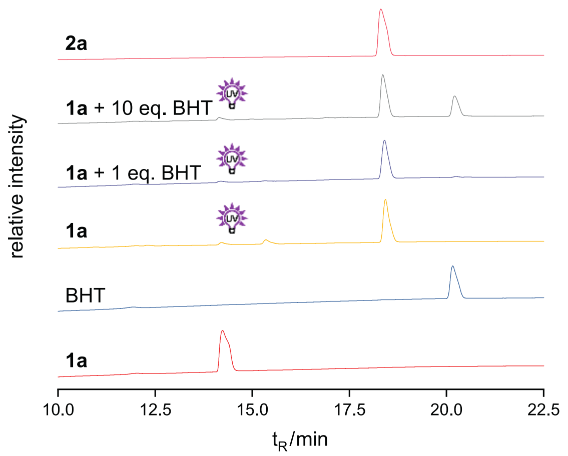

Figure S2. PDA traces from the addition of BHT as a radical quencher to sulfonamide **1a** with BHT, **1a** and **2a** for comparison.

*Presence of radical starters with sulfonamide **1a***

A stock solution of sulfonamide **1a** in benzene (3.1 mmol/L) was prepared. To aliquots of the stock solution (1 mL each) were added: a) 2,2´-azobis(2 methylpropionitrile) (AIBN, 0.38 mg, 2.3 µmol, 0.74 eq.) and tributylstannane (TBS, 1.1 mg, 1 µL, 3.8 µmol, 1.2 eq.), b) AIBN (0.38 mg, 2.3 µmol, 0.74 eq.) or c) benzoyl peroxide (DBPO, 0.64 mg, 2.64 µmol, 0.84 eq.). The resulting mixtures were stirred and heated to 70 °C for 25 h. 500 µL of a washing solution (saturated aqueous solution of NH_4F (300 µL), saturated aqueous solution of $NaHCO_3$ (300 µL) and water (3 mL)) was added to each reaction mixture and stirred vigorously. The organic layer was separated and the solvent was removed under a flow of nitrogen. The residue was analyzed by HPLC-HRMS in comparison with sulfonamide **1a** and biphenyl **2a**. The results are shown in Figure S3.

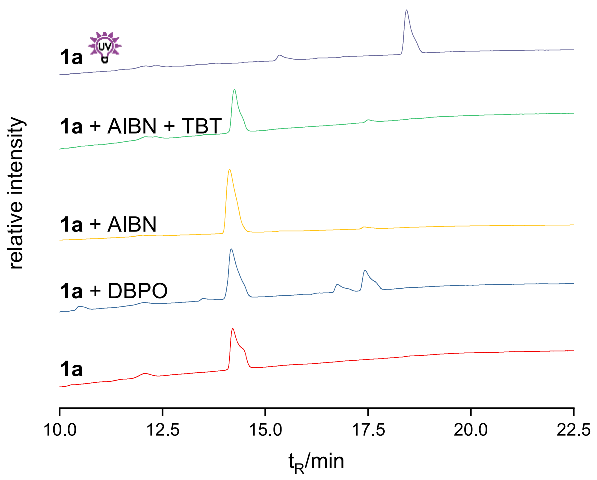

Figure S3. PDA traces from the reaction of radical initiators to sulfonamide **1a** in comparison with **1a** and irradiated **1a**.

*Excess of toluene during the photosplicing of sulfonamide **2s***

A solution of sulfonamide **2s** (18.9 mg, 61.9 µmol) in a mixture of MeCN and toluene (*v*/*v* = 1:1) (4 mL) was prepared. The solution was loaded on the photo reactor with a flow rate of 1 mL/min (MeCN/toluene (*v*/*v* = 1:1)) and irradiated with UV light at room temperature. The solvent fraction containing the photoproducts was collected and the solvent was removed under reduced pressure. The crude product was analyzed by HPLC-HRMS in comparison with **1s**, **2s** and **2a**. The results are shown in Figure S4.

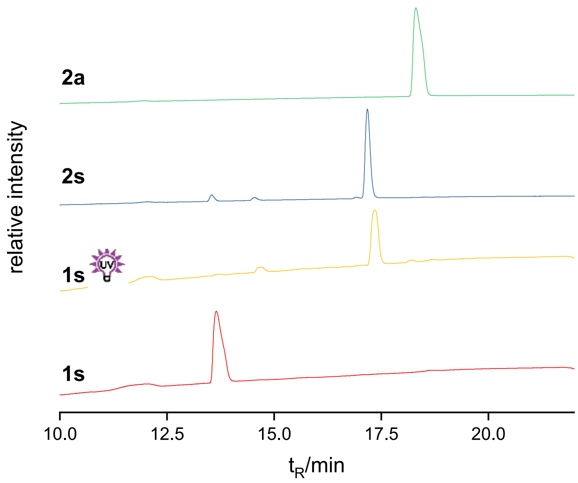

Figure S4. PDA traces from the reaction of **1s** in toluene/methanol in comparison with **1s** and **2a**.

Cross experiment

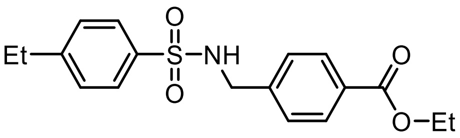

*Compound **1ak.*** Ethyl 4-(((4-ethylphenyl)sulfonamido)methyl)benzoate. A solution of ethyl 4-(aminomethyl)benzoate (105.4 mg, 0.49 mmol, 1 eq.), *N*,*N*-diisopropylamine (139 mg, 1.07 mmol, 2.2 eq., 183 µL) and 4-ethylbenzenesulfonyl chloride (100 mg, 0.49 mmol, 1 eq.) was stirred for 1 h at room temperature. The solution was washed with water (2 mL), aqueous hydrochloric acid (1 M, 2 mL) and brine (2 mL). The organic solvent was removed under reduced pressure and purified by column chromatography (silica, R_f=0.5, DCM/ethyl acetate 20:1) to give the title compound (154 mg, 0.44 mmol, 91%) as a white powder.
^{1}H NMR (300 MHz, $CDCl_3$): δ = 1.21–1.30 (t, $^3J_{HH}$ = 7.6 Hz, 3H, **H_3**C-CH_2-C), 1.33–1.42 (t, $^3J_{HH}$ = 7.1 Hz, 3H, **H_3**C-CH_2-O), 2.65–2.77 (q, $^3J_{HH}$ = 7.6, 2H, H_3C-C**H_2**-C), 4.15–4.22 (d, $^3J_{HH}$ = 6.3 Hz, 2H, N-C**H_2**), 4.29 - 4.41 (q, $^3J_{HH}$ = 7.1 Hz, 2H, H_3C-C**H_2**-O), 5.02–5.11 (t, $^3J_{HH}$ = 6.4 Hz, 1H, N**H**), 7.22–7.33 (m, 4H, CH_3-CH_2-C-(C**H**)$_2$ & N-CH_2-C-(C**H**)$_2$), 7.71–7.79 (d, $^3J_{HH}$ = 8.4 Hz, 2H, SO_2-C-(C**H**)$_2$), 7.88-7.95 (m, 2H, CO_2-C-(C**H**)$_2$) ppm. ^{13}C NMR (75 MHz, $CDCl_3$): δ = 14.4 (1C, H_3**C**-CH_2-O), 15.3 (1C, H_3**C**-CH_2-C), 28.9 (1C, H_3C-**C**H_2-C), 47.0 (1C, N-**C**H_2), 61.2 (1C, H_3C-**C**H_2-O), 127.4 (2C, N-CH_2-C-(**C**H)$_2$), 127.7 (2C, H_3C-CH_2-C-(**C**H)$_2$), 128.7 (2C, SO_2-C-(**C**H)$_2$), 130.0 (2C, CO_2-C-(**C**H)$_2$), 130.1 (1C, **C**-CO_2), 137.1 (1C, **C**-SO_2), 141.5 (1C, N-CH_2-**C**), 149.9 (1C, H_3C-CH_2-**C**), 166.3 (1C, C-**C**O_2) ppm. HRMS (ESI$^+$) calcd. for $C_{18}H_{22}NO_4S^+$: 348.1264; found: 348.1262.

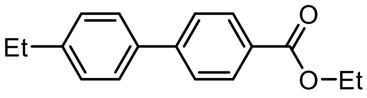

*Compound **2ak.*** Ethyl 4'-ethyl-[1,1'-biphenyl]-4-carboxylate. A solution of ethyl 4-(((4-ethylphenyl)sulfonamido)methyl)benzoate (**1ak**) (19.9 mg, 57 µmol) in MeCN (10 mL) was loaded on the photo reactor with a flow rate of 5 mL/min and irradiated with UV light (254 nm) at room temperature. The solvent fraction containing the photoproduct was collected and the solvent was removed under reduced pressure. The residue was purified by open column chromatography (silica, R_f=0.7, DCM) to yield the title compound (7.8 mg, 31 µmol, 54%).

^1H NMR (300 MHz, $CDCl_3$): δ = 1.23–1.34 (t, $^3J_{HH}$ = 7.6 Hz, 3H, **H₃**C-CH₂-C), 1.36–1.47 (t, $^3J_{HH}$ =7.1 Hz, 3H, **H₃**C-CH₂-O), 2.64–2.79 (q, $^3J_{HH}$ = 7.6 Hz, 2H, H₃C-C**H₂**-C), 4.33–4.49 (q, $^3J_{HH}$ =7.1 Hz, 2H, H₃C-C**H₂**-O), 7.27–7.36 (m, 2H, H₃C-CH₂-C-(C**H**)₂), 7.52–7.60 (m, 2H, H₃C-CH₂-C-(CH-C**H**)₂), 7.61–7.70 (m, 2H, CO₂-C-(CH-C**H**)₂), 8.05–8.15 (m, 2H, CO₂-C-(C**H**)₂) ppm. ^{13}C NMR (75 MHz, $CDCl_3$): δ = 14.5 (1C, H₃**C**-CH₂-O), 15.7 (1C, H₃**C**-CH₂-C), 28.7 (1C, H₃C-**C**H₂-C), 61.1 (1C, H₃C-**C**H₂-O), 126.9 (2C, CO₂-C-(CH-**C**H)₂), 127.3 (2C, H₃C-CH₂-C-(CH-**C**H)₂), 128.6 (2C, H₃C-CH₂-C-(**C**H)₂), 129.1 (1C, H₃C-CH₂-C-(CH-CH)₂-**C**), 130.2 (2C, CO₂-C-(**C**H)₂), 137.5 (1C, CO₂-C-(CH-CH)₂-**C**), 144.6 (1C, H₃C-CH₂-**C**), 145.6 (1C, CO₂-**C**), 166.7 (1C, **C**O₂-C) ppm. HRMS (ESI$^+$) calcd. for $C_{17}H_{19}O_2^+$: 255.1380; found: 255.1379.

A solution of methyl 4-(((4-methylphenyl)sulfonamido)methyl)benzoate (**1a**) (10 mg, 31 µmol, 1 eq.) and ethyl 4-(((4-ethylphenyl)sulfonamido)methyl)benzoate (**1ak**) (10.8 mg, 31 µmol, 1 eq.) in MeCN (10 mL) was loaded on the photo reactor with a flow rate of 1 mL/min and irradiated with UV light (254 nm) at room temperature. The solvent fraction containing the photoproduct was collected and was analyzed by HPLC-HRMS. No mixed products were detected as shown in Figure S5.

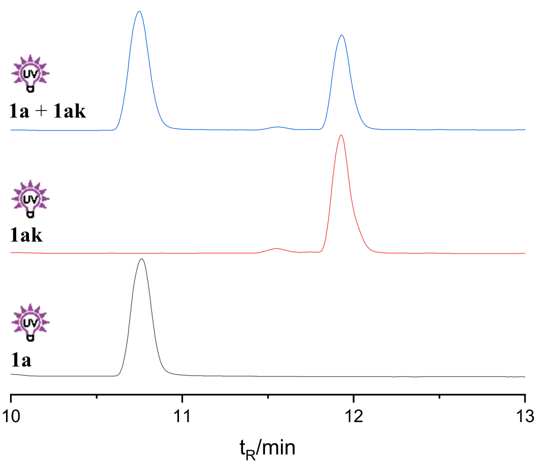

Figure S5. PDA traces from the irradiation of a mixture of **1a** and **1ak** in comparison with the crude products from the irradiation **1a** and **1ak**.

Modification of the linker

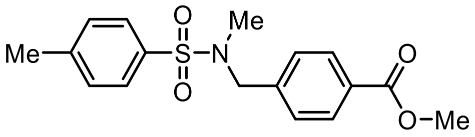

*Compound **1al**.* Methyl 4-(((*N*,4-dimethylphenyl)sulfonamido)methyl)benzoate. A solution of methyl 4-(((4-methylphenyl)sulfonamido)methyl)benzoate (**1a**) (68.8 mg, 0.22 mmol, 1 eq.) and K_2CO_3 (59.5 mg, 0.43 mmol, 1.5 eq.) was suspended in MeCN (3 mL) and dimethylsulfate (40.8 mg, 30.6 µL, 0.32 mmol, 1.5 eq.) was added. The solution was heated to 80 °C and stirred for 19 h. The solvent was removed under reduced pressure and the residue was dissolved in DCM (2 mL) and was washed with $NaHCO_3$ solution. The organic phase was dried over Na_2SO_4 and was removed under reduced pressure to yield the title product (72 mg, 0.22 mmol, 99%).
^{1}H NMR (300 MHz, $CDCl_3$) δ = 2.46 (s, 3H, -C-C**H₃**), 2.59 (s, 3H, -N(-C**H₃**)-), 3.91 (s, 3H, -O-C**H₃**), 4.18 (s, 2H, -N(-CH₃)-C**H₂**-), 7.37 (t, $^3J_{HH}$ = 7.8 Hz, 4H, H₃C-C-(C**H**)₂- & -CH₂-C-(C**H**)₂-), 7.77–7.70 (m, 2H, -SO₂-C-(C**H**)₂-), 8.04–7.97 (m, 2H, -C(=O)-C-(C**H**)₂-) ppm. ^{13}C NMR (75 MHz, $CDCl_3$) δ = 21.7 (1C, C-**C**H₃), 34.8 (1C, -N-**C**H₃), 52.3 (1C, -O-**C**H₃), 54.0 (1C, -N(-CH₃)-**C**H₂-C-), 127.6 (2C, -CH₂-C-(**C**H)₂-), 128.3 (2C, -SO₂-C-(**C**H)₂-), 129.9 (1C, -C(=O)-**C**-), 130.0 (2C, H₃C-C-(**C**H)₂-, 130.1 (2C, -C(=O)-C-(**C**H)₂-, 134.3 (1C, -SO₂-**C**), 141.1 (1C, H₃C-**C**-), 143.8 (1C, -CH₂-**C**-), 166.9 (1C, -**C**(=O)-O-) ppm. HRMS (ESI$^+$) calcd. for $C_{17}H_{20}NO_4S^+$: *m*/*z* = 334,1108, found: *m*/*z* = 334,1108.

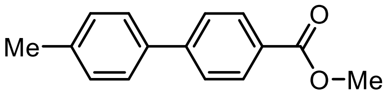

*Compound **2a** (=**2al**).* Methyl 4'-methyl-[1,1'-biphenyl]-4-carboxylate. A solution of methyl 4-(((*N*,4-dimethylphenyl)sulfonamido)methyl)benzoate (**1al**) (21.7 mg, 0.65 mmol) in MeCN (3 mL) was loaded on the photo reactor with a flow rate of 1 mL/min and irradiated with UV light (254 nm) at room temperature. The solvent fraction containing the photoproduct was collected and the solvent was removed under reduced pressure. The residue was purified by open column chromatography (silica, R_f=0.75, DCM) to yield the title compound (8 mg, 0.35 µmol, 54%).
NMR and HRMS spectra are equivalent to those of biphenyl **2a**.[3]

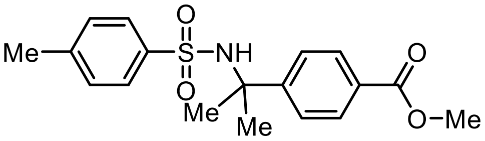

*Compound **1am**.* Methyl 4-(2-((4-methylphenyl)sulfonamido)propan-2-yl)benzoate. A solution of 4-methylbenzenesulfonyl chloride (42 mg, 0.22 mmol, 1 eq.), methyl 4-(2-aminopropan-2-yl)benzoate hydrochloride (50 mg, 0.22 mmol, 1 eq.) and *N*,*N*-diisopropylethylamine (62.6 mg, 82 µL, 0.48 mmol, 2.2 eq.) in DCM (2 mL) was stirred for 3 h at room temperature. The solution was washed with water (2 mL), aqueous hydrochloric acid (1 M, 2 mL) and brine (2 mL). The organic layer was dried with sodium sulfate, filtered and the solvent was evaporated. The residue was purified by open column chromatography (silica, R_f=0.22, DCM) to yield the title compound (36 mg, 0.10 mmol, 47%) as white crystals.
^{1}H NMR (500 MHz, $CDCl_3$) δ = 1.57–1.69 (s, 6H, (C**H₃**)₂C-N), 2.29–2.44 (s, 3H, C**H₃**C), 3.84–3.95 (s, 3H, C**H₃**-O-C), 5.30–5.45 (s, 1H, N**H**), 7.08–7.16 (d, $^3J_{HH}$ = 7.8 Hz, 2H, CH₃-C-(C**H**)₂), 7.31–7.38 (d, $^3J_{HH}$ = 8.7 Hz, 2H, C(CH₃)₂-C-(C**H**)₂), 7.47–7.56 (d, $^3J_{HH}$ = 8.2 Hz, 2H, SO₂-C-(C**H**)₂), 7.77–7.85 (d, $^3J_{HH}$ = 8.6 Hz, 2H, CO-C-(C**H**)₂) ppm. ^{13}C NMR (126 MHz, $CDCl_3$) δ = 21.5 (1C, **C**H₃-C-CH), 29.9 (2C, (**C**H₃)₂C), 52.2 (1C, **C**H₃-O), 58.5 (1C, (CH₃)₂**C**), 125.8 (2C, CH₃-C-(**C**H)₂), 127.0 (2C, C(CH₃)₂-C-(**C**H)₂), 128.8 (1C, **C**-CO), 129.4 (2C, SO₂-C-(**C**H)₂), 129.5 (2C, CO-C-(**C**H)₂), 139.6 (1C, CH₃-**C**-CH), 143.0 (1C, **C**-SO₂), 150.1 (1C, (CH₃)₂C-**C**), 166.9 (1C, **C**-O-CH₃) ppm. HRMS (ESI$^+$) calcd. for $C_{18}H_{22}NO_4S^+$: 348.1264; found: 348.1267. HRMS (ESI$^-$) calcd. for $C_{18}H_{20}NO_4S^-$: 346.1119; found: 346.1121.

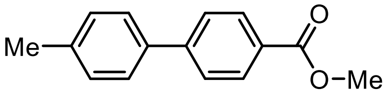

*Compound **2a** (=**2am**).* Methyl 4'-methyl-[1,1'-biphenyl]-4-carboxylate. A solution of methyl 4-(2-((4-methylphenyl)sulfonamido)propan-2-yl)benzoate (**1am**) (19.8 mg, 0.57 mmol) in MeCN (5 mL) was loaded on the photo reactor with a

flow rate of 1 mL/min and irradiated with UV light (254 nm) at room temperature. The solvent fraction containing the photoproduct was collected and the solvent was removed under reduced pressure. The residue was purified by open column chromatography (silica, R_f=0.75, DCM) to yield the title compound (8 mg, 0.35 mol, 62%).
NMR and HRMS spectra are equivalent to those of biphenyl **2a**.[3]

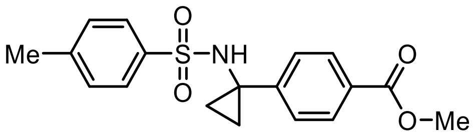

*Compound **1an.*** Methyl 4-(1-((4-methylphenyl)sulfonamido)cyclopropyl)benzoate. A solution of 4-methylbenzenesulfonyl chloride (30 mg, 0.16 mmol, 1 eq.), methyl-4-(1-aminocyclopropyl)benzoate (30 mg, 0.16 mmol, 1 eq.) and *N*,*N*-diisopropylethylamine (24.3 mg, 32.8 µL, 0.19 mmol, 1.2 eq.) in DCM (4 mL) was stirred for 12 h at room temperature and DMAP (20 mg, 0.16 mmol, 1 eq.) was added. After 1 h the solution was washed with water (3 mL), $NaHCO_3$ solution (3 mL) and aqueous hydrochloric acid (1 M, 3 mL). The organic layer was dried with sodium sulfate, filtered and the solvent was evaporated. The residue was purified by open column chromatography (silica, R_f=0.15, DCM) to yield the title compound (18.3 mg, 0.05 mmol, 34%) as white crystals.
^{1}H-NMR (300 MHz, $CDCl_3$) δ = 1.19 – 1.09 (m, 2H, -C**H**H-C**H**H-), 1.45 – 1.35 (m, 2H, -CH**H**-CH**H**-), 2.33 (s, 3H, -C-C**H$_3$**), 5.60 (s, 1H, -N**H**-), 3.89 (s, 3H, -O-C**H$_3$**), 7.19 – 7.04 (m, 4H, H_3C-C-(C**H**)$_2$- & -NH-C-C-(C**H**)$_2$-), 7.59–7.48 (m, 2H, -SO_2-C-(C**H**)$_2$-), 7.80–7.72 (m, 2H, -C(=O)-C-(C**H**)$_2$-) ppm. ^{13}C-NMR (75 MHz, $CDCl_3$) δ = 17.1 (2C, -NH-C(-**C**H_2)$_2$), 21.5 (1C, -C-**C**H_3), 37.8 (1C, -NH-**C**-), 52.2 (1C, -O-**C**H_3), 126.3 (2C, -NH-C-C-(**C**H)$_2$-), 127.2 (2C, -SO_2-C-(**C**H)$_2$-), 128.4 (1C, -C(=O)-**C**-), 129.5 (2C, -C(=O)-C-(**C**H)$_2$-), 129.6 (2C, H_3C-C-(**C**H)$_2$-), 138.4 (1C, H_3C-**C**-), 143.5 (1C, -NH-C-**C**-), 146.7 (1C, -SO_2-**C**-), 166.9 (1C, -**C**(=O)-O-) ppm. HRMS (ESI$^+$) calcd. for $C_{18}H_{20}NO_4S^+$: 346.1108; found: 346.1112. HRMS (ESI$^-$) calcd. for $C_{18}H_{18}NO_4S^-$: 344.0962; found: 344.0965.

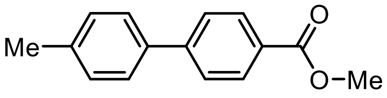

*Compound **2a** (=**2an**).* Methyl 4'-methyl-[1,1'-biphenyl]-4-carboxylate. A solution of methyl 4-(1-((4-methylphenyl)sulfonamido)cyclopropyl)benzoate (**1an**) (7.7 mg, 0.022 mmol) in MeCN (5 mL) was loaded on the photo reactor with a flow rate of 1 mL/min and irradiated with UV light (254 nm) at room temperature. The solvent fraction containing the photoproduct was collected and the solvent was removed under reduced pressure. The residue was purified by open column chromatography (silica, R_f=0.75, DCM) to yield the title compound (1.0 mg, 4.4 µmol, 20%).
NMR and HRMS spectra are equivalent to those of biphenyl **2a**.[3]

Chemical synthesis procedures for SAR studies

1a, **1b**, **1c**, **1h**, **1q**, **1s**, **1t**, **1u**, **2a**, **2b**, **2c**, **2h**, **2q**, **2s**, **2t** and **2u** are known compounds.[3]

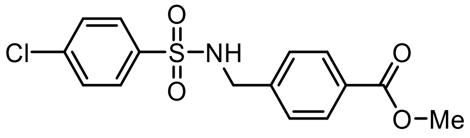

*Compound **1d**.* Methyl 4-(((4-chlorophenyl)sulfonamido)methyl)benzoate. A solution of 4-chlorobenzenesulfonyl chloride (211.1 mg, 1 mmol, 1 eq.), methyl 4-(aminomethyl)benzoate hydrochloride (201.7 mg, 1 mmol, 1 eq.) and *N*,*N*-diisopropylethylamine (284.4 mg, 383.2 µL, 2.2 mmol, 2.2 eq.) in DCM (4 mL) was stirred for 1 h at room temperature. After dilution with ethyl acetate (20 mL) the solution was washed with water (20 mL), aqueous hydrochloric acid (1 M, 20 mL) and brine (20 mL). The organic layer was dried with sodium sulfate, filtered and the solvent was evaporated. The crude product was obtained as white a solid (335.4 mg, 0.99 mmol, 99%) and used without further purification.
^{1}H NMR (600 MHz; $CDCl_3$): δ = 3.90 (s, 3H, -OC**H**$_3$), 4.20 (d, 2H, $^3J_{HH}$ = 5.7 Hz, -C**H**$_2$-), 5.00 (s, 1H, -N**H-**), 7.27 (d, 2H, $^3J_{HH}$ = 8.2 Hz, -CH$_2$-C-C**H**-), 7.46 (d, 2H, $^3J_{HH}$ = 8.6 Hz, -C(Cl)-C**H**-), 7.78 (d, 2H, $^3J_{HH}$ = 8.6 Hz, -C**H**-C-SO$_2$-), 7.93 (d, 2H, $^3J_{HH}$ = 8.2 Hz, -C**H**-C(COOMe)-) ppm. ^{13}C NMR (150.9 MHz; $CDCl_3$): δ = 47.0 (1C, -**C**H$_2$-), 52.4 (1C, -O**C**H$_3$), 127.8 (2C, -CH$_2$-C-**C**H-), 128.7 (2C, -**C**H-C-SO$_2$-), 129.6 (2C, -C(Cl)-**C**H-), 130.0 (1C, -**C**(COOMe)), 130.2 (2C, -**C**H-C(COOMe)-), 138.5 (1C, -**C**-SO$_2$-), 139.6 (1C, -**C**(Cl)-), 141.2 (1C, -CH$_2$-**C**-), 166.8 (1C, -**C**OOMe) ppm. HRMS (ESI$^+$) calcd. for $C_{15}H_{15}ClNO_4S^+$: 340.0405; found: 340.0408. HRMS (ESI$^-$) calcd. for $C_{15}H_{13}ClNO_4S^-$: 338.0259; found: 338.0255.

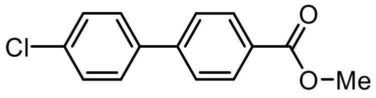

*Compound **2d**.* Methyl 4'-chloro-[1,1'-biphenyl]-4-carboxylate. A solution of methyl 4-(((4-chlorophenyl)sulfonamido)methyl)benzoate (**1d**) (21.5 mg, 63.3 µmol) in MeOH (10 mL) was loaded on the photo reactor with a flow rate of 1 mL/min (MeOH) and irradiated with UV light (254 nm) at room temperature. The solvent fraction containing the photoproduct was collected and the solvent was removed under reduced pressure. The residue was purified by open column chromatography (silica, R_f=0.68, DCM) to yield the title compound (13.5 mg, 54.7 µmol, 86%).
^{1}H NMR (300 MHz; $CDCl_3$): δ = 3.94 (s, 3H, -OC**H**$_3$), 7.42 – 7.45 (m, 2H, -C(Cl)-C**H**-), 7.53 – 7.56 (m, 2H, -C(Cl)-CH-C**H**-), 7.60 7.64 (m, 2H, -C**H**-CH-C(COOMe)-), 8.09 – 8.11 (m, 2H, -C**H**-C(COOMe)-) ppm. ^{13}C NMR (75.5 MHz; $CDCl_3$): δ = 52.3 (1C, -O**C**H$_3$), 127.0 (2C, -**C**H-CH-C(COOMe)-), 128.7 (2C, -C(Cl)-CH-**C**H-), 129.2 (2C, -C(Cl)-**C**H-), 129.3 (1C, -**C**(COOMe)-), 130.3 (2C, -**C**H-C(COOMe)-), 134.5 (1C, -**C**(Cl)-), 138.6 (1C, -**C**-C-CH-CH-C(COOMe)-), 144.5 (1C, -C-**C**-CH-CH-C(COOMe)-), 167.0 (1C, -**C**OOMe) ppm. HRMS (ESI$^+$) calcd. for $C_{14}H_{12}ClO_2^+$: 247.0520; found: 247.0520.

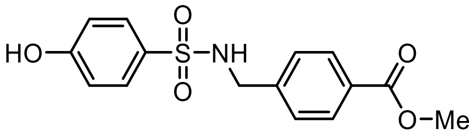

*Compound **1e**.* Methyl 4-(((4-hydroxyphenyl)sulfonamido)methyl)benzoate. A suspension of methyl 4-(((4-(benzyloxy)phenyl)sulfonamido)methyl)benzoate (**1w**) (100 mg, 0.243 mmol, 1 eq.) and palladium on carbon (26 mg, 5 mol%, 5% loading) in MeOH (2 mL) was hydrogenated for 1 h. The suspension was filtered off and the solvent was removed under reduced pressure. The crude product (75.5 mg, 0.235 mmol, 97%) was used without further purification.
^{1}H NMR (500 MHz; DMSO-D6): δ = 3.83 (s, 3H, -OC**H**$_3$), 4.00 (d, 2H, $^3J_{HH}$ = 6.4 Hz, -C**H**$_2$-), 6.88 (d, 2H, $^3J_{HH}$ = 8.7 Hz, -C(OH)-C**H**-), 7.39 (d, 2H, $^3J_{HH}$ = 8.3 Hz, -CH$_2$-C-C**H**-), 7.62 (d, 2H, $^3J_{HH}$ = 8.7 Hz, -C**H**-C(SO$_2$)-), 7.87 (d, 2H, $^3J_{HH}$ = 8.3 Hz, -C**H**-C(COOMe)-), 8.02 (d, 2H, $^3J_{HH}$ = 6.4 Hz, -N**H**-), 10.41 (s, 1H, -O**H**) ppm. ^{13}C NMR (125.8 MHz; DMSO-D6): δ = 45.7 (1C, -**C**H$_2$-), 52.1 (1C, -O**C**H$_3$), 115.5 (2C, -C(OH)-**C**H-), 127.7 (2C, -CH$_2$-C-**C**H-), 128.4 (1C, -**C**(COOMe)-), 128.9 (2C, -**C**H-C(SO$_2$)-), 129.1 (2C, -**C**H-C(COOMe)-), 130.5 (1C, -**C**(SO$_2$)-), 143.7 (1C, -CH$_2$-**C**-), 161.0 (1C, -**C**(OH)-), 166.1 (1C, -**C**OOMe) ppm. HRMS (ESI$^+$) calcd. for $C_{15}H_{16}NO_5S^+$: 322.0744; found: 322.0737. HRMS (ESI$^-$) calcd. for $C_{15}H_{14}NO_5S^-$: 320.0598; found: 320.0598.

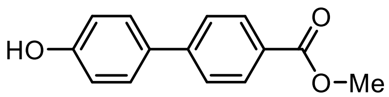

*Compound **2e.*** Methyl 4'-hydroxy-[1,1'-biphenyl]-4-carboxylate. A solution of methyl 4-(((4-hydroxyphenyl)sulfonamido)methyl)benzoate (**1e**) (22.5 mg, 70.1 µmol) in MeCN (10 mL) was loaded on the photo reactor with a flow rate of 1 mL/min (MeCN) and irradiated with UV light (254 nm) at room temperature. The solvent fraction containing the photoproduct was collected and the solvent was removed under reduced pressure. The residue was purified by open column chromatography (silica, R_f=0.18 DCM) to yield the title compound (12.1 mg, 53.1 µmol, 76%).
^{1}H NMR (500 MHz; $CDCl_3$): δ = 3.86 (s, 3H, -OC**H**$_3$), 6.88 (d, 2H, $^3J_{HH}$ = 8.7 Hz, -C(OH)-C**H**-), 7.58 (d, 2H, $^3J_{HH}$ = 8.7 Hz, -C(OH)-CH-C**H**-), 7.74 (d, 2H, $^3J_{HH}$ = 8.5 Hz, -C**H**-CH-C(COOMe)-), 7.98 (d, 2H, $^3J_{HH}$ = 8.5 Hz, -C**H**-C(COOMe)-), 9.72 (s, 1H, -O**H**) ppm. ^{13}C NMR (125.8 MHz; $CDCl_3$): δ = 52.0 (1C, -O**C**H$_3$), 115.9 (2C, -C(OH)-**C**H-), 126.0 (2C, -**C**H-CH-C(COOMe)-), 127.3 (1C, -**C**(COOMe)-), 128.2 (2C, -C(OH)-CH-**C**H-), 129.4 (1C, -C(OH)-CH-CH-**C**-), 129.7 (2C, -**C**H-C(COOMe)-), 144.7 (1C, -**C**-CH-CH-C(COOMe)-), 158.0 (1C, -**C**(OH)-), 166.1 (1C, -**C**OOMe) ppm. HRMS (ESI$^-$) calcd. for $C_{14}H_{11}O_3^-$: 227.0714; found: 227.0719.

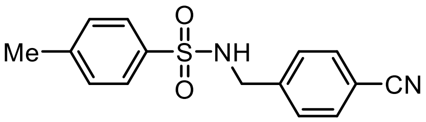

*Compound **1f.*** *N*-(4-Cyanobenzyl)-4-methylbenzenesulfonamide. A solution of 4-methylbenzenesulfonyl chloride (190.6 mg, 1 mmol, 1 eq.) and 4-(aminomethyl)benzonitrile hydrochloride (168.6 mg, 1 mmol, 1 eq.) in 1,4-dioxane (3 mL) was prepared and *N*,*N*-diisopropylethylamine (284.4 mg, 383.2 µL, 2.2 mmol, 2.2 eq.) was added. The solution was stirred for 24 h at room temperature. The mixture was diluted with aqueous hydrochloric acid (1 M, 30 mL) and ethyl acetate (50 mL). The separated organic phase was washed with brine (30 mL). The organic layer was dried with sodium sulfate, filtered and the solvent was removed under reduced pressure. This provided the title compound as a white powder (282 mg, 0.98 mmol, 98%).
^{1}H NMR (300 MHz; $CDCl_3$): δ = 2.43 (s, 3H, C-C**H**$_3$), 4.16 (m, 2H, -C**H**$_2$-), 5.38 (s, 1H, -N**H**-), 7.26–7.29 (m, 2H, -C(CH$_3$)-C**H**-), 7.31 - 7.34 (m, 2H, -CH$_2$-C-C**H**-), 7.51 – 7.54 (m, 2H, -C**H**-C(CN)-), 7.69 – 7.72 (m, 2H, -C**H**-C(SO$_2$)-) ppm. ^{13}C NMR (75.5 MHz; $CDCl_3$): δ = 21.5 (1C, -**C**H$_3$), 46.5 (1C, -**C**H$_2$-), 111.5 (1C, -**C**(CN)-), 118.5 (1C, -**C**N), 127.0 (2C, -**C**H-C(SO$_2$)-), 128.3 (2C, -CH$_2$-C-**C**H-), 129.8 (2C, -C(CH$_3$)-**C**H-), 132.3 (2C, -**C**H-C(CN)-), 136.5 (1C, -**C**(CH$_3$)-), 142.0 (1C, -CH$_2$-**C**-), 143.9 (1C, -**C**-SO$_2$-) ppm. HRMS (ESI$^+$) calcd. for $C_{15}H_{15}N_2O_2S^+$: 287.0849; found: 287.0840. HRMS (ESI$^-$) calcd. for $C_{15}H_{13}N_2O_2S^-$: 285.0703; found: 285.0703.

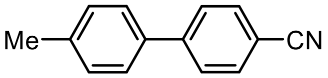

*Compound **2f.*** 4'-Methyl-[1,1'-biphenyl]-4-carbonitrile. A solution of *N*-(4-cyanobenzyl)-4-methylbenzenesulfonamide (**1f**) (27.6 mg, 96.4 µmol) in MeOH (30 mL) was loaded on the photo reactor with a flow rate of 1.0 mL/min (MeOH) and irradiated with UV light (254 nm) at 8 °C. The solvent fraction containing the photoproduct was collected and the solvent was removed under reduced pressure. The residue was purified by preparative HPLC to yield the title compound (13.7 mg, 70.9 µmol, 74%).
^{1}H NMR (500 MHz; $CDCl_3$): δ = 2.42 (s, 3H, -C**H**$_3$), 7.27 – 7.31 (m, 2H, -C**H**-C-CH$_3$-), 7.47 - 7.51 (m, 2H, -C**H**-CH-C-CH$_3$), 7.65 - 7.73 (m, 4H, -C**H**-C**H**-C-CN) ppm. ^{13}C NMR (125.8 MHz; $CDCl_3$): δ = 21.2 (1C, -**C**H$_3$), 110.5 (1C, -**C**-CN-), 119.0 (1C, **C**N), 127.0 (2C, -**C**H-CH-C-CH$_3$-), 127.4 (2C, -**C**H-CH-C-CN), 129.8 (2C, -**C**H-C-CH$_3$-), 132.5 (2C, -**C**H-C-CN), 136.3 (1C, -**C**-CH-CH-C-CH$_3$-), 138.7 (1C, **C**-CH$_3$), 145.6 (1C, -**C**-CH-CH-C-CN) ppm. HRMS (ESI$^+$) calcd. for $C_{14}H_{12}N^+$: 194.0964; found: 194.0965.

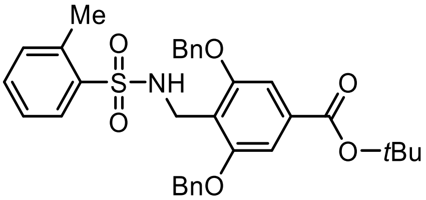

*Compound **1g.*** *tert*-Butyl 3,5-bis(benzyloxy)-4-(((2-methylphenyl)sulfonamido)methyl) benzoate. A solution of *tert*-butyl 3,5-bis(benzyloxy)4-(((*o*-tolylsulfonyl)-imino)methyl)benzoate (85 mg, 0.15 mmol, 1 eq.) in dried THF was prepared under argon. Freshly prepared lithium methoxide (0.23 mg, 6 µmol, 0.04 eq.) and trimethoxysilane (63.6 mg, 0.52 mmol, 47 µL, 3.5 eq.) were added at 0 °C and the mixture was allowed to warm up to room temperature within 3 hours. After dilution with ethyl acetate (20 mL) the solution was washed with water (10 mL), aqueous hydrochloric acid (1 M, 10 mL) and brine (10 mL). The organic layer was dried with sodium sulfate, filtered and the solvent was removed under reduced pressure. The crude product was purified by recrystallization from MeOH/water (*v*/*v* = 1:1) to provide the title compound as white crystals (44 mg, 76.7 µmol, 52%).
^{1}H NMR (300 MHz; CD_3OD): δ = 1.55 (s, 9H, -C(C**H**$_3$)$_3$), 2.41 (s, 3H, -C(C**H**$_3$)-), 4.28 (s, 2H, -C**H**$_2$-NH-), 5.07 (s, 4H, -O-C**H**$_2$-Ph), 7.08 – 7.14 (m, 2H, Me-C$_6$**H**$_4$-SO$_2$-), 7.12 (s, 2H, -C(OBn)-C**H**-), 7.33 – 7.44 (m, 12H, Me-C$_6$**H**$_4$-SO$_2$- and -O-CH$_2$-C$_6$**H**$_5$), 7.76 (dd, 1H,

$^3J_{HH}$ = 7.9 Hz, $^4J_{HH}$ = 1.3 Hz, -N**H**-) ppm. ^{13}C NMR (75.5 MHz; CD$_3$OD): δ = 20.2 (1C, Ar-**C**H$_3$), 28.4 (3C, -C(**C**H$_3$)$_3$), 36.6 (1C, -**C**H$_2$-), 71.5 (2C, -O-**C**H$_2$-Ph), 82.6 (1C, -**C**(CH$_3$)$_3$), 107.2 (2C, -C(OBn)-**C**H-), 119.1 (1C, -NH-CH$_2$-**C**-), 126.8 (1C, -C(SO$_2$)-CH-**C**H-), 128.5 (4C, -CH$_2$-C-**C**H-), 129.0 (2C, -CH$_2$-C-CH-CH-**C**H-), 129.7 (4C, -CH$_2$-C-CH-**C**H-), 130.3 (1C, -C(SO$_2$)-**C**H-), 133.3 (1C, -C(CH$_3$)-**C**H-), 133.4 (1C, -C(CH$_3$)-CH-**C**H-), 134.1 (1C, -**C**-CO-), 138.2 (2C, -O-CH$_2$-**C**-), 138.3 (1C, -C(SO$_2$)-**C**(CH$_3$)-), 139.6 (1C, -**C**(SO$_2$)-), 158.6 (2C, -**C**(OBn)-), 166.6 (1C, -**C**O-) ppm. HRMS (ESI$^-$) calcd. for $C_{33}H_{34}NO_6S^-$: 572.2112; found: 572.2111.

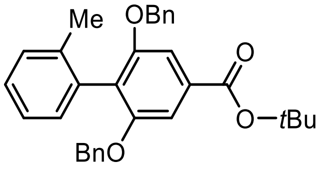

*Compound **2g.*** *tert*-Butyl 2,6-bis(benzyloxy)-2'-methyl-[1,1'-biphenyl]-4-carboxylate. A solution of *tert*-butyl 3,5-bis(benzyloxy)-4-(((2-methylphenyl)sulfonamido)methyl)benzoate (**1g**) (12.0 mg, 20.9 µmol) in MeOH (5 mL, containing 0.01 M NH$_3$) was loaded on the photo reactor with a flow rate of 4 mL/min (MeOH, containing 0.01 M NH$_3$) and irradiated with UV light (254 nm) at room temperature. The solvent fraction containing the photoproduct was collected and the solvent was removed under reduced pressure. The residue was purified by open column chromatography (silica, R$_f$=0.79, DCM) to yield the title compound (6.8 mg, 14.1 µmol, 67%).
^{1}H NMR (300 MHz; CD$_3$CN): δ = 1.58 (s, 9H, -C(C**H**$_3$)$_3$), 2.07 (s, 3H, -C(C**H**$_3$)-), 5.06 (s, 4H, -O-C**H**$_2$-Ph), 7.13 – 7.33 (m, 14H, Me-C$_6$**H**$_4$-SO$_2$- and -O-CH$_2$-C$_6$**H**$_5$), 7.36 (s, 2H, -C(OBn)-C**H**-) ppm. ^{13}C NMR (75.5 MHz; CD$_3$CN): δ = 19.9 (1C, Ar-**C**H$_3$), 28.3 (3C, -C(**C**H$_3$)$_3$), 71.1 (2C, -O-**C**H$_2$-Ph), 82.1 (1C, -**C**(CH$_3$)$_3$), 107.8 (2C, -C(OBn)-**C**H-), 125.3 (1C, -**C**-CO-), 126.2 (1C, -C(CH$_3$)-C-C**H**-), 128.2 (4C, -CH$_2$-C-**C**H-), 128.4 (1C, -C(CH$_3$)-CH-CH-**C**H-), 128.7 (2C, -CH$_2$-C-CH-CH-**C**H-), 129.3 (4C, -CH$_2$-C-CH-**C**H-), 130.4 (1C, -C(CH$_3$)-CH-**C**H-), 131.3 (1C, -C(CH$_3$)-**C**H-), 134.9 (1C, -C(OBn)-**C**-), 137.9 (1C, -C(CH$_3$)-**C**-), 138.1 (1C, -**C**(CH$_3$)-), 157.5 (2C, -**C**(OBn)-), 166.0 (1C, -**C**O-) ppm. HRMS (ESI$^+$) calcd. for $C_{32}H_{33}O_4^+$: 481.2373; found: 481.2363. HRMS (ESI$^-$) calcd. for $C_{32}H_{31}O_4^-$: 479.2228; found: 479.2232.

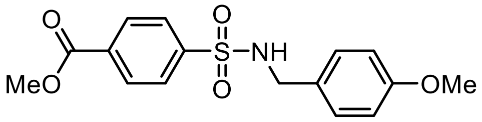

*Compound **1i.*** Methyl 4-(*N*-(4-methoxybenzyl)sulfamoyl)benzoate. A solution of methyl 4-(chlorosulfonyl)benzoate (234.7 mg, 1 mmol, 1 eq.), (4-methoxyphenyl)methanamine (137.2 mg, 1 mmol, 1 eq.) and *N*,*N*-diisopropylethylamine (142.1 mg, 191.6 µL, 1.1 mmol, 1.1 eq.) in DCM (4 mL) was stirred for 16 h at room temperature. After dilution with ethyl acetate (20 mL) the solution was washed with water (10 mL), aqueous hydrochloric acid (1 M, 10 mL) and brine (10 mL). The organic layer was dried with sodium sulfate, filtered and the solvent was removed under reduced pressure. The crude product was purified by recrystallization from ethanol/water (*v*/*v* = 1:1) which provided the title compound as a white powder (273 mg, 0.81 mmol, 81%).
^{1}H NMR (500 MHz; CDCl$_3$): δ = 3.76 (s, 3H, -C(OC**H**$_3$)), 3.97 (s, 3H, -C(O)-OC**H**$_3$), 4.10 (d, 2H, $^3J_{HH}$ = 6.0 Hz, -C**H**$_2$-), 4.82 (t, 1H, $^3J_{HH}$ = 6.0 Hz, -N**H**-), 6.78 (d, 2H, $^3J_{HH}$ = 8.6 Hz, -C(OMe)-C**H**-), 7.08 (d, 2H, $^3J_{HH}$ = 8.6 Hz, -C(OMe)-CH-C**H**-), 7.91 (d, 2H, $^3J_{HH}$ = 8.5 Hz, -C(COOMe)-CH-C**H**-), 8.15 (d, 2H, $^3J_{HH}$ = 8.5 Hz, -C(COOMe)-C**H**-) ppm. ^{13}C NMR (125.8 MHz; CDCl$_3$): δ = 47.0 (1C, -**C**H$_2$-), 52.8 (1C, -COO**C**H$_3$), 55.4 (1C, -C(O**C**H$_3$)), 114.3 (2C, -C(OMe)-**C**H-), 127.7 (2C, -C(COOMe)-CH-**C**H-), 128.0 (1C, -**C**(CH$_2$)-), 129.4 (2C, -C(OMe)-CH-**C**H-), 130.5 (2C, -C(COOMe)-**C**H-), 133.9 (1C, -**C**(COOMe)-), 144.2 (1C, -**C**(SO$_2$)-), 159.6 (1C, -**C**(OCH$_3$)), 165.8 (1C, -**C**OOMe) ppm. HRMS (ESI$^+$) calcd. for $C_{16}H_{18}NO_5S^+$: 336.0900; found: 336.0892. HRMS (ESI$^-$) calcd. for $C_{16}H_{16}NO_5S^-$: 334.0755; found: 334.0754.

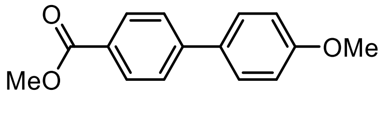

*Compound **2i.*** Methyl 4'-methoxy-[1,1'-biphenyl]-4-carboxylate. A solution of methyl 4-(*N*-(4-methoxybenzyl)sulfamoyl)benzoate (**1i**) (20.6 mg, 62.3 µmol) in MeOH (10 mL) was loaded on the photo reactor with a flow rate of 10 mL/min (MeOH) and irradiated with UV light (254 nm) at room temperature. The solvent fraction containing the photoproduct was collected and the solvent was removed under reduced pressure. The residue was purified by open column chromatography (silica, R$_f$=0.66, DCM) to yield the title compound (9.1 mg, 37.6 µmol, 61%).
NMR and HRMS spectra are equivalent to those of biphenyl **2i**.[3]

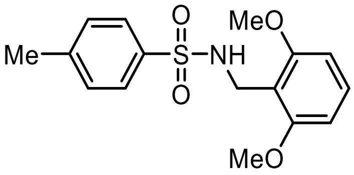

*Compound **1j.*** *N*-(2,6-Dimethoxybenzyl)-4-methylbenzenesulfonamide. A solution of 4-methylbenzenesulfonyl chloride (190.6 mg, 1 mmol, 1 eq.), (2,6-dimethoxyphenyl)methanamine (167.2 mg, 1 mmol, 1 eq.) and *N*,*N*-diisopropylethylamine (142.1 mg, 191.6 µL, 1.1 mmol, 1.1 eq.) in DCM (4 mL) was stirred for 1 h at room temperature. After dilution with ethyl acetate (20 mL) the solution was washed with water (20 mL), aqueous hydrochloric acid (1 M, 20 mL) and brine (20 mL). The organic layer was dried with sodium sulfate, filtered and the solvent was evaporated. The crude product was purified by recrystallization from ethanol/water (*v*/*v* = 3:2) to provide the title compound as white crystals (257 mg, 80.0 µmol, 80%).
^{1}H NMR (500 MHz; $CDCl_3$): δ = 2.36 (s, 3H, -C**H**$_3$), 3.71 (s, 6H, -OC**H**$_3$), 4.25 (d, 2H, $^3J_{HH}$ = 6.3 Hz, -C**H**$_2$-), 5.17 (t, 1H, $^3J_{HH}$ = 6.3 Hz, -N**H**-), 6.38 (d, 2H, $^3J_{HH}$ = 8.4 Hz, -C(OMe)-C**H**-), 7.10 (t, 1H, $^3J_{HH}$ = 8.4 Hz, -CH-C**H**-CH-), 7.15 (d, 2H, $^3J_{HH}$ = 8.4 Hz, -C(Me)-C**H**-), 7.64 (d, 2H, $^3J_{HH}$ = 8.4 Hz, -C(Me)-CH-C**H**-) ppm. ^{13}C NMR (125.8 MHz; $CDCl_3$): δ = 21.5 (1C, -**C**H$_3$), 36.5 (1C, -**C**H$_2$-), 55.7 (2C, -O**C**H$_3$), 103.6 (2C, -C(OMe)-**C**H-), 112.8 (1C, -CH$_2$-**C**-), 127.2 (2C, -C(Me)-CH-**C**H-), 129.1 (2C, -C(Me)-**C**H-), 129.3 (1C, -CH-**C**H-CH-), 137.4 (1C, -**C**(Me)-), 142.8 (1C, -**C**-SO$_2$-), 158.1 (2C, -**C**(OMe)-) ppm. HRMS (ESI$^+$) calcd. for $C_{16}H_{20}NO_4S^+$: 322.1108; found: 322.1108. HRMS (ESI$^-$) calcd. for $C_{16}H_{18}NO_4S^-$: 320.0951; found: 320.0966.

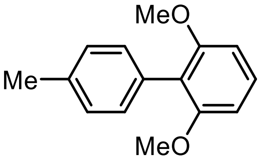

*Compound **2j.*** 2,6-Dimethoxy-4'-methyl-1,1'-biphenyl. A solution of *N*-(2,6-dimethoxybenzyl)-4-methylbenzenesulfonamide (**1j**) (26.0 mg, 80.9 µmol) in MeOH (10 mL) was loaded on the photo reactor with a flow rate of 1 mL/min (MeOH) and irradiated with UV light (254 nm) at room temperature. The solvent fraction containing the photoproduct was collected and the solvent was removed under reduced pressure. The residue was purified by open column chromatography (silica, R_f=0.88, DCM) to yield the title compound (10.6 mg, 46.5 µmol, 57%).
^{1}H NMR (500 MHz; $CDCl_3$): δ = 2.40 (s, 3H, -C**H**$_3$), 3.74 (s, 6H, -OC**H**$_3$), 6.66 (d, 2H, $^3J_{HH}$ = 8.4 Hz, -C(OMe)-C**H**-), 7.22–7.28 (m, 5H, -C(Me)-C**H**-C**H**- and –CH-C**H**-CH-) ppm. ^{13}C NMR (125.8 MHz; $CDCl_3$): δ = 21.5 (1C, -**C**H$_3$), 56.1 (2C, -O**C**H$_3$), 104.4 (2C, -C(OMe)-**C**H-), 119.7 (1C, -**C**-C(OMe)-), 128.6 (1C, –CH-**C**H-CH-), 128.7 (2C, -C(Me)-**C**H-CH-), 130.9 (2C, -C(Me)-CH-**C**H-), 131.2 (1C, -CH-**C**-C-C(OMe)-), 136.4 (1C, -**C**(Me)-), 157.9 (2C, -**C**(OMe)-) ppm. HRMS (ESI$^+$) calcd. for $C_{15}H_{17}O_2^+$: 229.1223; found: 229.1231.

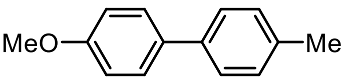

*Compound **2k.*** 4-Methoxy-4'-methyl-1,1'-biphenyl. A solution of 4-methoxy-*N*-(4-methylbenzyl)benzenesulfonamide (Alfa Aesar, H56245, **1k**) (20.2 mg, 69.3 µmol) in MeCN (5 mL) was loaded on the photo reactor with a flow rate of 1 mL/min (MeCN) and irradiated with UV light (254 nm) at room temperature. The solvent fraction containing the photoproduct was collected and the solvent was removed under reduced pressure. The residue was purified by open column chromatography (DCM) to yield the title compound (4.3 mg, 21.7 µmol, 36%).
^{1}H NMR (300 MHz; CD_2Cl_2): δ = 2.37 (s, 3H, C**H**$_3$-C), 3.83 (s, 3H, C**H**$_3$-O), 6.95 – 9.98 (m, 2H, (C**H**)$_2$-C-OCH$_3$), 7.23 (d, 2H, $^3J_{HH}$ = 8.0 Hz, CH$_3$-C-(C**H**)$_2$), 7.45 (d, 2H, $^3J_{HH}$ = 8.0 Hz, CH$_3$-C-(CH)$_2$-(C**H**)$_2$), 7.50 – 7.53 (m, 2H, (C**H**)$_2$-(CH)$_2$-C-OCH$_3$) ppm.
^{13}C NMR (75.5 MHz; CD_2Cl_2): δ = 21.3 (1C, C-**C**H$_3$), 55.8 (1C, O-**C**H$_3$), 114.7 (2C, (**C**H)$_2$-C-OCH$_3$), 126.9 (2C, CH$_3$-C-(CH)$_2$-(**C**H)$_2$), 128.3 (2C, (**C**H)$_2$-(CH)$_2$-C-OCH$_3$), 130.0 (2C, CH$_3$-C-(**C**H)$_2$), 134.0 (1C, **C**-(CH)$_2$-(CH)$_2$-C-OCH$_3$), 137.0 (1C, CH$_3$-**C**), 138.3 (1C, CH$_3$-C-(CH)$_2$-(CH)$_2$-**C**), 159.6 (1C, CH$_3$-O-**C**) ppm. HRMS (ESI$^+$) calcd. for $C_{14}H_{15}O^+$: 199.1117; found: 199.1117.

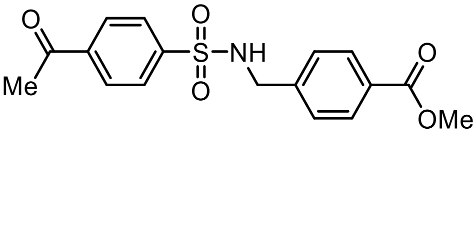

Compound ***1l.*** Methyl 4-(((4-acetylphenyl)sulfonamido)methyl)benzoate. A solution of 4-acetylbenzenesulfonyl chloride (218.7 mg, 1 mmol, 1 eq.), methyl 4-(aminomethyl)benzoate hydrochloride (201.7 mg, 1 mmol, 1 eq.) and *N*,*N*-diisopropylethylamine (284.4 mg, 383.2 µL, 2.2 mmol, 2.2 eq.) in DCM (4 mL) was stirred for 1 h at room temperature. After dilution with ethyl acetate (20 mL) the solution was washed with water (10 mL), aqueous hydrochloric acid (1 M, 10 mL) and brine (10 mL). The organic layer was dried with sodium sulfate, filtered and the solvent was evaporated. The crude product was obtained as white a solid (200.3 mg, 0.58 mmol, 58%) and used without further purification.
^{1}H NMR (300 MHz; CD_3CN): δ = 2.59 (s, 3H, -CO-C**H**$_3$), 3.84 (s, 3H, -OC**H**$_3$), 4.18 (d, 2H, $^3J_{HH}$ = 6.3 Hz, -C**H**$_2$-), 6.28 (t, 1H, $^3J_{HH}$ = 6.3 Hz, -N**H**-), 7.31 (d, 2H, $^3J_{HH}$ = 8.5 Hz, -CH$_2$-C-C**H**-), 7.84 – 7.89 (m, 4H, -C**H**-C-COOMe and -C**H**-C-SO$_2$-), 8.01 - 8.04 (m, 2H, Me-CO-C-C**H**-) ppm. ^{13}C NMR (75.5 MHz; CD_3CN): δ = 27.3 (1C, -CO-**C**H$_3$), 47.2 (1C, -**C**H$_2$-), 52.7 (1C, -O**C**H$_3$), 128.1 (2C, -**C**H-C-SO$_2$-), 128.9 (2C, -CH$_2$-C-**C**H-), 129.9 (2C, Me-CO-C-**C**H-), 130.4 (2C, -**C**H-C-COOMe), 130.4 (1C, -**C**(COOMe)), 141.0 (1C, Me-CO-**C**-), 143.4 (1C, -CH$_2$-**C**-), 145.2 (1C, -**C**-SO$_2$-), 167.4 (1C, -**C**OOMe), 198.2 (1C, -**C**O-Me) ppm. HRMS (ESI$^+$) calcd. for $C_{17}H_{18}NO_5S^+$: 348.0900; found: 348.0903. HRMS (ESI$^-$) calcd. for $C_{17}H_{16}NO_5S^-$: 346.0755; found: 346.0757.

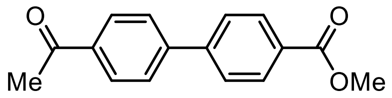

Compound ***2l.*** Methyl 4'-acetyl-[1,1'-biphenyl]-4-carboxylate. A solution of methyl 4-(((4-acetylphenyl)sulfonamido)methyl)benzoate (**1l**) (19.8 mg, 57.1 µmol) in MeCN (10 mL) was loaded on the photo reactor with a flow rate of 2 mL/min (MeCN) and irradiated with UV light (254 nm) at room temperature. The solvent fraction containing the photoproduct was collected and the solvent was removed under reduced pressure. The residue was purified by open column chromatography (silica, R_f=0.81, DCM) to yield the title compound (5.0 mg, 20.1 µmol, 35%).
^{1}H NMR (300 MHz; $CDCl_3$): δ = 2.65 (s, 3H, -CO-C**H**$_3$), 3.85 (s, 3H, -OC**H**$_3$), 7.68 – 7.73 (m, 4H, -C**H**-C-C-C**H**-), 8.05 (d, 2H, $^3J_{HH}$ = 8.3 Hz, Me-CO-C-C**H**-), 8.13 (d, 2H, $^3J_{HH}$ = 8.3 Hz, -C**H**-C-COOMe) ppm. ^{13}C NMR (75.5 MHz; $CDCl_3$): δ = 26.8 (1C, -CO-**C**H$_3$), 52.4 (1C, -**C**H$_2$-), 127.4 (2C, -**C**H-C-C-**C**H-), 127.6 (2C, -**C**H-C-C-**C**H-), 129.1 (2C, Me-CO-C-**C**H-), 129.9 (1C, -**C**-COOMe), 130.4 (2C, -**C**H-C-COOMe), 136.7 (1C, Me-CO-**C**-), 144.4 (1C, Me-CO-C-CH-CH-C-**C**-), 144.6 (1C, Me-CO-C-CH-CH-**C**-), 166.9 (1C, -**C**OOMe), 197.8 (1C, -**C**O-Me) ppm. HRMS (ESI$^+$) calcd. for $C_{16}H_{15}O_3^+$: 255.1016; found: 255.1015.

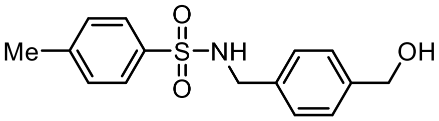

Compound ***1ao.*** *N*-(4-(Hydroxymethyl)benzyl)-4-methylbenzenesulfonamide. A solution of 4-methylbenzenesulfonyl chloride (190.6 mg; 1 mmol; 1 eq.), (4-(aminomethyl)phenyl)methanol (137,2 mg; 1 mmol; 1 eq.) and *N*,*N*-diisopropylethylamine (142,1 mg; 191,6 µL; 1,1 mmol; 1,1 eq.) in DCM (4 mL) was stirred for 1 h at room temperature. The solution was diluted with ethyl acetate (20 mL) washed with water (10 mL), aqueous hydrochloric acid (1 M, 10 mL) and brine (10 mL). The organic layer was dried with sodium sulfate, filtered and the solvent was evaporated. The crude product was purified by recrystallization from ethanol/water (*v*/*v* = 1:1) to provide the title compound as a white crystalline compound (245.7 mg; 0.84 mmol; 84%).
^{1}H NMR (500 MHz; CD_3CN): δ = 2.42 (s; 3H; -C**H**$_3$); 3.17 (t; 1H; $^3J_{HH}$ = 5.9 Hz; -O**H**); 4.00 (d; 2H; $^3J_{HH}$ = 6.4 Hz; -NH-C**H**$_2$-); 4.52 (d; 2H; $^3J_{HH}$ = 5.9 Hz; -C**H**$_2$OH); 5.93 (t; 1H; $^3J_{HH}$ = 6.4 Hz; -N**H**-); 7.18 (d; 2H; $^3J_{HH}$ = 8.0 Hz; -NH-CH$_2$-C-C**H**-); 7.24 (d; 2H; $^3J_{HH}$ = 8.0 Hz; -C**H**-C-CH$_2$OH); 7.36 (d; 2H; $^3J_{HH}$ = 8.2 Hz; -C(CH$_3$)-C**H**-); 7.71 (d; 2H; $^3J_{HH}$ = 8.2 Hz; -C(CH$_3$)-CH-C**H**-) ppm. ^{13}C NMR (125.8 MHz; CD_3CN): δ = 21.4 (1C; -**C**H$_3$); 47.4 (1C; -NH-**C**H$_2$-); 64.3 (1C; -**C**H$_2$OH); 127.7 (2C; -**C**H-C-CH$_2$OH); 127.8 (2C; -C(CH$_3$)-CH-**C**H-); 128.7 (2C; -NH-CH$_2$-C-**C**H-); 130.6 (2C; -C(CH$_3$)-**C**H-); 137.0 (1C; -CH$_2$-**C**-); 138.4 (1C; -**C**-SO$_2$-); 142.3 (1C; -**C**-CH$_2$OH); 144.5 (1C; -**C**-CH$_3$) ppm. HRMS (ESI$^+$) calcd. for $C_{15}H_{18}NO_3S^+$: 292.1024; found: 292.0994. HRMS (ESI$^-$) calcd. for $C_{15}H_{16}NO_3S^-$: 290.0856; found: 290.0852.

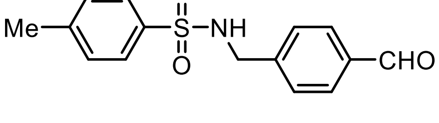

Compound ***1m.*** *N*-(4-Formylbenzyl)-4-methylbenzenesulfonamide. A solution of *N*-(4-(hydroxymethyl)benzyl)-4-methylbenzenesulfonamide (**1ao**) (100 mg, 0.34 mmol, 1 eq.) and pyridinium chlorochromate (109.9 mg, 0.51 mmol, 1.5 eq.) in MeCN (4 mL) was stirred for 1 h at room temperature. After dilution with ethyl acetate (20 mL) the solution was washed with water (10 mL) and brine (10 mL). The organic layer was dried with sodium sulfate, filtered and the solvent was evaporated. The residue

was dissolved, filtered and purified by open column chromatography (silica, R_f=0.49, chloroform/MeOH 95:5) to yield the title compound (96.8 mg, 0.33 mmol, 97%) as a white amorphous solid.
^{1}H NMR (500 MHz; $CDCl_3$): δ = 2.43 (s, 3H, -C**H**$_3$), 4.21 (d, 2H, $^3J_{HH}$ = 6.4 Hz, -C**H**$_2$-), 4.97 (t, 1H, $^3J_{HH}$ = 6.0 Hz, -N**H**-), 7.30 (d, 2H, $^3J_{HH}$ = 8.0 Hz, -C**H**-C(SO_2)-), 7.39 (d, 2H, $^3J_{HH}$ = 8.0 Hz, -CH$_2$-C-C**H**-), 7.75 (d, 2H, $^3J_{HH}$ = 8.2 Hz, -C(Me)-C**H**-), 7.79 (d, 2H, $^3J_{HH}$ = 8.1 Hz, -C**H**-C(CHO)-), 9.97 (s, 1H, -C**H**O) ppm. ^{13}C NMR (125.8 MHz; $CDCl_3$): δ = 21.7 (1C, -**C**H$_3$), 47.0 (1C, -**C**H$_2$-), 127.3 (2C, -**C**H-C(SO_2)-), 128.4 (2C, -CH$_2$-C-**C**H-), 130.0 (2C, -C(Me)-**C**H-), 130.2 (2C, -**C**H-C(CHO)-), 136.1 (1C, -**C**(CHO)-), 136.9 (1C, -**C**(Me)-), 143.5 (1C, -CH$_2$-**C**-), 144.0 (1C, -**C**(SO_2)-), 191.9 (1C, -**C**HO) ppm. HRMS (ESI$^+$) calcd. for $C_{16}H_{15}NO_3S^+$: 290.0845; found: 290.0840. HRMS (ESI$^-$) calcd. for $C_{16}H_{15}NO_3S^-$: 288.0700; found: 288.0697.

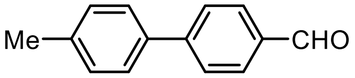

Compound ***2m.*** 4'-Methyl-[1,1'-biphenyl]-4-carbaldehyde. A solution of *N*-(4-formylbenzyl)-4-methylbenzenesulfonamide (**1m**) (17.5 mg, 60.5 µmol) in MeCN (10 mL) was loaded on the photo reactor with a flow rate of 1.5 mL/min (MeCN) and irradiated with UV light (254 nm) at room temperature. The solvent fraction containing the photoproduct was collected and the solvent was removed under reduced pressure. The residue was purified by open column chromatography (silica, R_f=0.76, DCM) to yield the title compound (3.7 mg, 18.9 µmol, 31%).
^{1}H NMR (500 MHz; $CDCl_3$): δ = 2.42 (s, 3H, -C**H**$_3$), 7.29 (d, 2H, $^3J_{HH}$ = 7.9 Hz, -C(Me)-C**H**-), 7.55 (d, 2H, $^3J_{HH}$ = 8.2 Hz, -C(Me)-CH-C**H**-), 7.74 (d, 2H, $^3J_{HH}$ = 8.2 Hz, -C**H**-CH-C(CHO)-), 7.94 (d, 2H, $^3J_{HH}$ = 8.4 Hz, -C**H**-C(CHO)-), 10.05 (s, 1H, -C**H**O) ppm. ^{13}C NMR (125.8 MHz; $CDCl_3$): δ = 21.3 (1C, -**C**H$_3$), 127.4 (2C, -C(Me)-CH-**C**H-), 127.6 (2C, -**C**H-CH-C(CHO)-), 129.9 (2C, -C(Me)-**C**H-), 130.4 (2C, -**C**H-C(CHO)-), 135.1 (1C, -**C**(CHO)-), 137.0 (1C, -C(Me)-CH-CH-**C**-), 138.7 (1C, -**C**(Me)-), 147.3 (1C, -**C**-CH-CH-C(CHO)-), 192.1 (1C, -**C**HO) ppm. HRMS (ESI$^+$) calcd. for $C_{14}H_{13}O^+$: 197.0961; found: 197.0969.

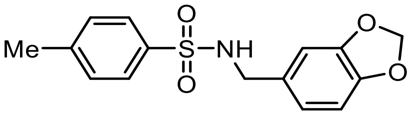

Compound ***1n.*** *N*-(benzo[*d*][1,3]dioxol-5-ylmethyl)-4-methylbenzenesulfonamide. A solution of benzo[*d*][1,3]dioxol-5-ylmethanamine (302.3 mg, 249 µL, 2 mmol, 1 eq.), *N*,*N* diisopropylamine (310.2 mg, 408 µL, 2.4 mmol, 1.2 eq.) and 4-methylbenzenesulfonyl chloride (381.3 mg, 2 mmol, 1 eq.) in DCM (4 mL) was stirred for 48 h at room temperature. The solution was washed with water (3 mL), aqueous hydrochloric acid (0.5 M, 3 mL) and water (3 mL). The organic solvent was dried over Na_2SO_4, removed under reduced pressure and purified by column chromatography (silica, R_f=0.43, DCM) to give the title compound (454.4 mg, 1.49 mmol, 74%) as slightly yellow crystals.
^{1}H NMR (500 MHz, $CDCl_3$): δ = 2.44 (s, 3H, -C**H**$_3$), 4.01 (d, 2H, $^3J_{HH}$ = 6.1 Hz, -N-C**H**$_2$-), 4.69 (t, 1H, $^3J_{HH}$ = 6.2 Hz, -N**H**-), 5.92 (s, 2H, -O-C**H**$_2$-O), 6.70-6,61 (m, 3H, -C**H**-C(-O-)-C(-O-)-C**H**-C**H**-), 7.31 (d, 2H, $^3J_{HH}$ = 8.0 Hz, -C**H**-C-CH$_3$), 7.74 (d, 2H, $^3J_{HH}$ = 8.0 Hz, -C**H**-C-SO$_2$-) ppm. ^{13}C NMR (126 MHz, $CDCl_3$): δ = 21.7 (1C, -**C**H$_3$), 47.3 (1C, -NH-**C**H$_2$-), 101.3 (1C, -O-**C**H$_2$-O-), 108.4 (1C, -C-CH-**C**H-C(-O-)-, 108.6 (1C, -C-**C**H-C(-O-)-, 121.5 (1C, -C-**C**H-CH-C(-O-)), 127.3 (2C, -**C**H-CH-C-CH$_3$), 129.9 (2C, -**C**H-C-CH$_3$), 130.2 (1C, -NH-CH$_2$-**C**-), 137.0 (1C, -**C**-CH$_3$), 143.7 (1C, -**C**-SO$_2$-), 147.4 (1C, -C-CH-**C**(-O-)-C(-O-)-), 148.0 (1C, -C-CH-C(-O-)-**C**(-O-)-) ppm. HRMS (ESI$^+$) calcd. for $C_{15}H_{16}NO_4S^+$: 306.0795, found: 306.0793.

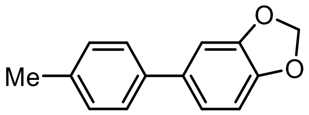

Compound ***2n.*** 5-(*p*-tolyl)benzo[*d*][1,3]dioxole. A solution of ethyl *N*-(benzo[*d*][1,3]dioxol-5-ylmethyl)-4-methylbenzenesulfonamide (**1n**) (19.98 mg, 65.4 µmol) in MeCN (3 mL) was loaded on the photo reactor with a flow rate of 0.5 mL/min and irradiated with UV light (254 nm) at room temperature. The solvent fraction containing the photoproduct was collected and the solvent was removed under reduced pressure. The residue was purified by open column chromatography (silica, R_f=0.87, DCM) to yield the title compound (4 mg, 18.9 µmol, 29%).
^{1}H NMR (500 MHz, $CDCl_3$) δ = 2.38 (s, 3H, -C**H$_3$**), 5.99 (s, 2H, -O-C**H**$_2$-O), 6.87 (d, 1H, $^3J_{HH}$ = 7.9 Hz, -C(-O-)-C**H**-CH-), 7.08 - 7.01 (m, 2H, -C**H**-C-C**H**-C(-O-)-), 7.22 (d, 2H, $^3J_{HH}$ = 8.3 Hz, Me-C-C**H**-CH-), 7.41 (d, 2H, $^3J_{HH}$ = 8.2 Hz, Me-C-CH-C**H**-) ppm. ^{13}C NMR (151 MHz, $CDCl_3$) δ = 21.2 (C1, -**C**H$_3$), 101.2 (C1, -O-CH$_2$-O-), 107.7 (C1, -C-CH-**C**H-C(-O-)-), 108.7 (C1, -C-**C**H-C(-O-)-), 120.5 (C1, -C-**C**H-CH-C(-O-)-), 126.9 (2C, -**C**H-CH-C-CH$_3$), 129.6 (2C, -**C**H-C-CH$_3$), 135.7 (C1, -**C**-CH$_3$), 136.8 (C1, -C(-O-)-CH-C-**C**-), 138.2 (C1, -C(-O-)-CH-**C**-C-),146.9 (C1, -C-CH-C(-O-)-**C**(-O)-), 148.2 (C1, -C-CH-**C**(-O-)-) ppm. HRMS (ESI$^+$) calcd. for $C_{14}H_{13}O_2^+$: 213.0910, found: 213.0910.

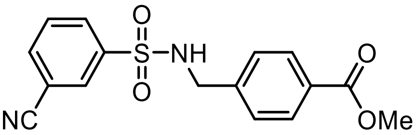

Compound ***1o****.* Methyl 4-(((3-cyanophenyl)sulfonamido)methyl)benzoate. A solution of 3-cyanobenzenesulfonyl chloride (201.6 mg, 1 mmol, 1 eq.), methyl 4-(aminomethyl)benzoate hydrochloride (201.7 mg, 1 mmol, 1 eq.) and triethylamine (253 mg, 347.5 µL, 2.5 mmol, 2.5 eq.) in 1,4-dioxane (12 mL) was stirred for 12 h at room temperature. The mixture was diluted with ethyl acetate (50 mL) and washed with aqueous hydrochloric acid (2 M, 25 mL) and water (50 mL). The organic layer was dried with sodium sulfate, filtered and the solvent was removed under reduced pressure. The crude product was purified by preparative HPLC to provide the title compound (100 mg, 0.3 mmol, 30%).
^{1}H NMR (300 MHz; $CDCl_3$): δ = 3.92 (s, 3H, -OC**H**$_3$), 4.27 (d, 2H, $^3J_{HH}$ = 6.1 Hz, -C**H**$_2$-), 5.44 (t, 1H, $^3J_{HH}$ = 6.1 Hz, -N**H**-), 7.27 (d, 2H, $^3J_{HH}$ = 8.3 Hz, -CH_2-C-C**H**-), 7.63 (t, 1H, $^3J_{HH}$ = 7.8 Hz, -CH-C**H**-CH-), 7.84 (d, 1H, $^3J_{HH}$ = 7.8 Hz, -C(CN)-C**H**-CH-CH-), 7.92 (d, 2H, $^3J_{HH}$ = 8.3 Hz, -C**H**-C(COOMe)-), 8.05 (d, 1H, $^3J_{HH}$ = 7.8 Hz, -C(CN)-CH-CH-C**H**-), 8.08 (m, 1H, -C(CN)-C**H**-C(SO_2)-) ppm. ^{13}C NMR (75.5 MHz; $CDCl_3$): δ = 47.0 (1C, -**C**H_2-), 52.4 (1C, -O**C**H_3), 113.8 (1C, -**C**(CN)-), 117.2 (1C, -**C**N), 127.9 (2C, -CH_2-C-**C**H-), 130.0 (1C, -**C**(COOMe)-), 130.1 (2C, -**C**H-C(COOMe)-), 130.3 (1C, -CH-**C**H-CH-), 130.8 (1C, -C(CN)-**C**H-C(SO_2)-), 131.0 (1C, -C(CN)-CH-CH-**C**H-), 135.9 (1C, -C(CN)-**C**H-CH-CH-), 140.9 (1C, -CH_2-**C**-), 142.0 (1C, -**C**(SO_2)-), 166.7 (1C, -**C**OOMe) ppm. HRMS (ESI$^+$) calcd. for $C_{16}H_{15}N_2O_4S^+$: 331.0747; found: 331.0738. HRMS (ESI$^-$) calcd. for $C_{16}H_{13}N_2O_4S^-$: 329.0602; found: 329.0600.

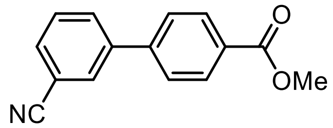

Compound ***2o****.* Methyl 3'-cyano-[1,1'-biphenyl]-4-carboxylate. A solution of methyl 4-(((3-cyano)sulfonamido)methyl)benzoate (**1o**) (20.3 mg, 61.4 µmol) in MeOH (20 mL) was loaded on the photo reactor with a flow rate of 1.0 mL/min (MeOH) and irradiated with UV light (254 nm) at room temperature. The solvent fraction containing the photoproduct was collected and the solvent was evaporated under reduced pressure. The residue was purified by column chromatography (silica, R_f=0.78, DCM) to yield the title compound (3.0 mg, 12.6 µmol, 21%).
^{1}H NMR (500 MHz; $CDCl_3$): δ = 3.96 (s, 3H, -OC**H**$_3$), 7.56–7.61 (m, 1H, C**H**-CH-C-C≡N), 7.62–7.66 (m, 2H, -C**H**-CH-C-(C=O)-), 7.66-7.70 (m, 1H, -CH-C**H**-C-C≡N), 7.82–7.87 (m, 1H, -C**H**-C-CH-C-C≡N), 7.90 (m, 1H, -C-C**H**-C-C≡N), 8.13-8.17 (m, 2H, -C**H**-C-(C=O)-) ppm. ^{13}C NMR (125.8 MHz; $CDCl_3$): δ = 52.3 (1C, -O**C**H_3), 113.3 (1C, -**C**-CN-), 118.5 (1C, -**C**N), 127.1 (2C, -**C**H-CH-C-(C=O)-), 129.8 (1C, -**C**H-CH-C-C≡N), 130.1 (1C, -**C**-(C=O)-), 130.4 (2C, -**C**H-C-(C=O)-), 130.8 (1C, -C-**C**H-C-C≡N), 131.5 (1C, -CH-**C**H-C-C≡N), 131.6 (1C, -**C**H-C-CH-C-C≡N), 141.3 (1C, -**C**-CH-C-C≡N), 143.1 (1C, -**C**-C-CH-C-C≡N), 166.6 (1C, -(**C**=O)-) ppm. HRMS (ESI$^+$) calcd. for $C_{15}H_{12}O_2N^+$: 238.0863; found: 238.0858.

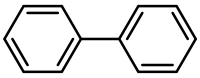

Compound ***2p.*** 1,1'-Biphenyl. A solution of *N*-benzylbenzenesulfonamide (Alfa Aesar, H55588, **1p**) (20.5 mg, 82.9 µmol) in MeOH (5 mL) was loaded on the photo reactor with a flow rate of 1.5 mL/min (MeOH) and irradiated with UV light (254 nm) at room temperature. The solvent fraction containing the photoproduct was collected and the solvent was removed under reduced pressure. The residue was purified by filtration over silica (DCM) to yield the title compound (traces).
GCMS (EI, 70 eV): 154 (100).

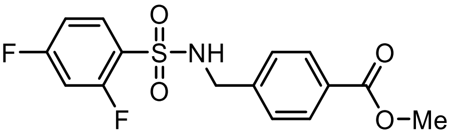

Compound ***1r.*** Methyl 4-(((2,4-difluorophenyl)sulfonamido)methyl)benzoate. A solution of 2,4-difluorobenzenesulfonyl chloride (212.2 mg, 134 µL, 1 mmol, 1 eq.), methyl 4-(aminomethyl)benzoate hydrochloride (201.7 mg, 1 mmol, 1 eq.) and *N*,*N*-diisopropylethylamine (284.4 mg, 383.2 µL, 2.2 mmol, 2.2 eq.) in DCM (4 mL) was stirred for 1 h at room temperature. After dilution with ethyl acetate (20 mL) the solution was washed with water (10 mL), aqueous hydrochloric acid (1 M, 10 mL) and brine (10 mL). The organic layer was dried with sodium sulfate, filtered and the solvent was evaporated. The crude product was purified by

recrystallization from ethanol/water (*v*/*v* = 1:1) to provide the title compound as a white crystalline compound (316.5 mg, 0.93 mmol, 93%).

^{1}H NMR (500 MHz; $CDCl_3$): δ = 3.90 (s, 3H, -OC**H**$_3$), 4.27 (d, 2H, $^{3}J_{HH}$ = 6.4 Hz, -C**H**$_2$-), 5.21 (t, 1H, $^{3}J_{HH}$ = 5.9 Hz, -N**H**-), 6.89–6.93 (m, 1H, -CF-C**H**-CF-), 6.95 - 6.99 (m, 1H, -CF-C**H**-CH-), 7.29 (d, 2H, $^{3}J_{HH}$ = 8.1 Hz, -CH$_2$-C-C**H**-), 7.86 - 7.90 (m, 1H, -CF-CH-C**H**-), 7.93 (d, 2H, $^{3}J_{HH}$ = 8.1 Hz, -C**H**-C(COOMe)) ppm. ^{13}C NMR (125.8 MHz; $CDCl_3$): δ = 47.1 (1C, -**C**H$_2$-), 52.4 (1C, -O**C**H$_3$), 105.7 (t, 1C, $^{2}J_{CF}$ = 25.5 Hz, -CF-**C**H-CF-), 112.1 (dd, 1C, $^{2}J_{CF}$ = 21.9 Hz, $^{4}J_{CF}$ = 3.3 Hz, -CF-**C**H-CH-), 124.7 (dd, 1C, $^{2}J_{CF}$ = 14.0 Hz, $^{4}J_{CF}$ = 4.0 Hz, -**C**-SO$_2$-), 127.8 (2C, -CH$_2$-C-**C**H-), 130.0 (1C, -**C**(COOMe)), 130.1 (2C, -**C**H-C(COOMe)), 132.1 (d, 1C, $^{3}J_{CF}$ = 10.4 Hz, -CF-CH-**C**H-), 141.1 (1C, -CH$_2$-**C**-), 159.6 (dd, 1C, $^{1}J_{CF}$ = 257.2 Hz, $^{3}J_{CF}$ = 13.3 Hz, -**C**F-C-SO$_2$-), 166.0 (dd, 1C, $^{1}J_{CF}$ = 258.0 Hz, $^{3}J_{CF}$ = 12.0 Hz, -**C**F-CH-CH-), 166.7 (1C, -**C**OOMe) ppm. HRMS (ESI$^+$) calcd. for $C_{15}H_{14}NO_4F_2S^+$: 342.0606; found: 342.0590. HRMS (ESI$^-$) calcd. for $C_{15}H_{12}NO_4F_2S^-$: 340.0461; found: 340.0455.

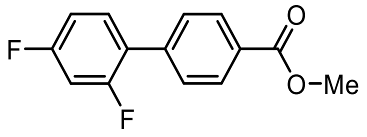

Compound ***2r.*** Methyl 2',4'-difluoro-[1,1'-biphenyl]-4-carboxylate. A solution of methyl 4-(((2,4-difluorophenyl)sulfonamido)methyl)benzoate (**1r**) (19.5 mg, 57.2 µmol) in MeCN (10 mL) was loaded on the photo reactor with a flow rate of 2 mL/min (MeCN) and irradiated with UV light (254 nm) at room temperature. The solvent fraction containing the photoproduct was collected and the solvent was removed under reduced pressure. The residue was purified by open column chromatography (silica, R_f=0.75, DCM) to yield the title compound (11.2 mg, 45.2 µmol, 79%).

^{1}H NMR (500 MHz; $CDCl_3$): δ = 3.94 (s, 3H, -OC**H**$_3$), 6.91 - 7.00 (m, 2H, -CF-C**H**-CF- and -CF-C**H**-CH-), 7.41 - 7.45 (m, 1H, -CF-CH-C**H**-), 7.57 (m, 2H, -C**H**-CH-C(COOMe)), 8.10 (d, 2H, $^{3}J_{HH}$ = 8.3 Hz, -C**H**-C(COOMe)) ppm. ^{13}C NMR (125.8 MHz; $CDCl_3$): δ = 52.3 (1C, -O**C**H$_3$), 104.7 (t, 1C, $^{2}J_{CF}$ = 25.9 Hz, -CF-**C**H-CF-), 111.9 (dd, 1C, $^{2}J_{CF}$ = 21.3 Hz, $^{4}J_{CF}$ = 3.7 Hz, -CF-**C**H-CH-), 124.4 (m, 1C, -CF-**C**-C-), 129.0 (m, 2C, -**C**H-CH-C(COOMe)), 129.5 (1C, -**C**(COOMe)), 129.9 (2C, -**C**H-C(COOMe)), 131.6 (m, 1C, -CF-CH-**C**H-), 139.7 (1C, -CF-C-**C**-), 159.95 (dd, 1C, $^{1}J_{CF}$ = 250.1 Hz, $^{3}J_{CF}$ = 12.3 Hz, -**C**F-C-), 162.9 (dd, 1C, $^{1}J_{CF}$ = 251.5 Hz, $^{3}J_{CF}$ = 12.2 Hz, -**C**F-CH-CH-), 167.0 (1C, -**C**OOMe) ppm. HRMS (ESI$^+$) calcd. for $C_{14}H_{11}O_2F_2^+$: 249.0722; found: 249.0725.

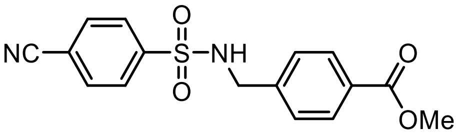

Compound ***1v.*** Methyl 4-(((4-cyanophenyl)sulfonamido)methyl)benzoate. A solution of 4-cyanobenzenesulfonyl chloride (201.6 mg, 1 mmol, 1 eq.), methyl 4-(aminomethyl)benzoate hydrochloride (201.7 mg, 1 mmol, 1 eq.) and triethylamine (253 mg, 347.5 µL, 2.5 mmol, 2.5 eq.) in 1,4-dioxane (12 mL) was stirred for 12 h at room temperature. The mixture was diluted with ethyl acetate (50 mL) and washed with aqueous hydrochloric acid (2 M, 25 mL) and water (50 mL). The organic layer was dried with sodium sulfate, filtered and the solvent was removed under reduced pressure. The crude product was purified by preparative HPLC to provide the title compound (252 mg, 0.77 mmol, 77%).

^{1}H NMR (300 MHz; $CDCl_3$): δ = 3.90 (s, 3H, -OC**H**$_3$), 4.24 (d, 2H, $^{3}J_{HH}$ = 5.7 Hz, -C**H**$_2$-), 5.34 (s, 1H, -N**H**-), 7.25 (d, 2H, $^{3}J_{HH}$ = 7.9 Hz, -CH$_2$-C-C**H**-), 7.76 (d, 2H, $^{3}J_{HH}$ = 8.1 Hz, -C(CN)-C**H**-), 7.89 - 7.94 (m, 4H, -C**H**-C(SO$_2$)- and -C**H**-C(COOMe)-) ppm. ^{13}C NMR (75.5 MHz; $CDCl_3$): δ = 47.0 (1C, -**C**H$_2$-), 52.4 (1C, -O**C**H$_3$), 116.6 (1C, -**C**(CN)-), 117.3 (1C, -**C**N), 127.8 (4C, -CH$_2$-C-**C**H- and -**C**H-C(SO$_2$)-); 130.1 (1C, -**C**(COOMe)), 130.1 (2C, -**C**H-C(COOMe)-), 133.1 (2C, -C(CN)-**C**H-), 140.9 (1C, -CH$_2$-**C**-), 144.4 (1C, **C**-SO$_2$-), 166.9 (1C, -**C**OOMe) ppm. HRMS (ESI$^+$) calcd. for $C_{16}H_{15}N_2O_4S^+$: 331.0747; found: 331.0739. HRMS (ESI$^-$) calcd. for $C_{16}H_{13}N_2O_4S^-$: 329.0602; found: 329.0600.

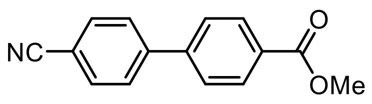

Compound ***2v.*** Methyl 4'-cyano-[1,1'-biphenyl]-4-carboxylate. A solution of methyl 4-(((4-cyano)sulfonamido)methyl)benzoate (**1v**) (21.9 mg, 66.3 µmol) in MeOH (20 mL) was loaded on the photo reactor with a flow rate of 1.0 mL/min (MeOH) and irradiated with UV light (254 nm) at room temperature. The solvent fraction containing the photoproduct was collected and the solvent was removed under reduced pressure. The residue was purified by column chromatography (silica, R_f=0.68, chloroform/MeOH 49:1) to yield the title compound (10.1 mg, 42.6 µmol, 64%).

^{1}H NMR (500 MHz; CD_2Cl_2): δ = 3.92 (s, 3H, -OC**H**$_3$), 7.68 - 7.72 (m, 2H, -C**H**-CH-C-(C=O)-), 7.73 - 7.79 (m, 4H, -C**H**-C**H**-C-C≡N), 8.11 - 8.15 (m, 2H, -C**H**-C-(C=O)-) ppm. ^{13}C NMR (125.8 MHz; CD_2Cl_2): δ = 52.7 (1C, -O**C**H$_3$), 112.4 (1C, -**C**-CN-), 119.2 (1C, **C**N), 127.8 (2C, -**C**H-CH-C-(C=O)-), 128.5 (2C, -**C**H-CH-C-C≡N), 130.7 (2C, -**C**H-C-(C=O)-), 130.9 (1C, -**C**-(C=O)-), 133.3 (2C, -**C**H-C-C

≡N), 144.0 (1C, -**C**-CH-CH-C-(C=O)-), 144.9 (1C, -**C**-CH-CH-C-C≡N), 167.0 (1C, -(**C**=O)-) ppm. HRMS (ESI$^+$) calcd. for $C_{15}H_{12}O_2N^+$: 238.0863; found: 238.0858.

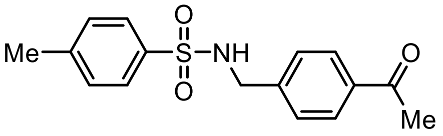

*Compound **1w.*** *N*-(4-Acetylbenzyl)-4-methylbenzenesulfonamide. A solution of 4-methylbenzenesulfonyl chloride (190.7 mg, 1 mmol, 1 eq.), 1-(4-(aminomethyl)phenyl)ethan-1-one hydrochloride (185.7 mg, 1 mmol, 1 eq.) and *N*,*N*-diisopropylethylamine (284.4 mg, 383.2 µL, 2.2 mmol, 2.2 eq.) in DCM (4 mL) was stirred for 2 h at room temperature. After dilution with ethyl acetate (20 mL) the solution was washed with water (10 mL), aqueous hydrochloric acid (1 M, 10 mL) and brine (10 mL). The organic layer was dried with sodium sulfate, filtered and the solvent was evaporated. The crude product was purified by recrystallization from ethanol/water (*v*/*v* = 1:1) to provide the title compound as a white crystalline compound (214.4 mg, 0.71 mmol, 71%).
^{1}H NMR (500 MHz; $CDCl_3$): δ = 2.43 (s, 3H, -C**H**$_3$), 2.56 (s, 3H, -C(O)-C**H**$_3$), 4.17 (d, 2H, $^3J_{HH}$ = 6.4 Hz, -C**H**$_2$-), 5.07 (t, 1H, $^3J_{HH}$ = 6.3 Hz, -N**H**-), 7.29 - 7.31 (m, 4H, -CH$_2$-C-C**H**- and -C(Me)-C**H**-), 7.74 (d, 2H, $^3J_{HH}$ = 8.2 Hz, -C(Me)-CH-C**H**-), 7.84 (d, 2H, $^3J_{HH}$ = 8.2 Hz, -CH$_2$-C-CH-C**H**-) ppm. ^{13}C NMR (125.8 MHz; $CDCl_3$): δ = 21.7 (1C, -**C**H$_3$), 26.8 (1C, -C(O)-**C**H$_3$), 46.9 (1C, -**C**H$_2$-), 127.3 (2C, -C(Me)-CH-**C**H-), 128.0 (2C, -CH$_2$-C-**C**H-), 128.8 (2C, -CH$_2$-C-CH-**C**H-), 129.9 (2C, -C(Me)-**C**H-), 136.7 (1C, -**C**(COMe)-), 136.9 (1C, -**C**(SO$_2$)-), 141.9 (1C, -CH$_2$-**C**-), 143.9 (1C, -**C**(Me)-), 197.8 (1C, -**C**(O)-) ppm. HRMS (ESI$^+$) calcd. for $C_{16}H_{18}NO_3S^+$: 304.1002; found: 304.0997. HRMS (ESI$^-$) calcd. for $C_{16}H_{16}NO_3S^-$: 302.0856; found: 302.0857.

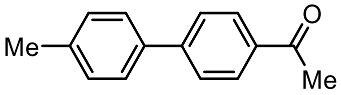

*Compound **2w.*** 1-(4'-Methyl-[1,1'-biphenyl]-4-yl)ethan-1-one. A solution of *N*-(4-acetylbenzyl)-4-methylbenzenesulfonamide (**1w**) (18.6 mg, 64.3 µmol) in MeOH (10 mL) was loaded on the photo reactor with a flow rate of 1 mL/min (MeOH) and irradiated with UV light (254 nm) at room temperature. The solvent fraction containing the photoproduct was collected and the solvent was removed under reduced pressure. The residue was purified by open column chromatography (silica, R_f=0.56, DCM) to yield the title compound (7.6 mg, 38.7 µmol, 60%).
^{1}H NMR (500 MHz; $CDCl_3$): δ = 2.41 (s, 3H, -C**H**$_3$), 2.63 (s, 3H, -C(O)-C**H**$_3$), 7.28 (d, 2H, $^3J_{HH}$ = 8.0 Hz, -C(Me)-C**H**-), 7.53 (d, 2H, $^3J_{HH}$ = 8.0 Hz, -C(Me)-CH-C**H**-), 7.67 (d, 2H, $^3J_{HH}$ = 8.4 Hz, -C**H**-CH-C(COMe)-), 8.02 (d, 2H, $^3J_{HH}$ = 8.4 Hz, -C**H**-C(COMe)-) ppm. ^{13}C NMR (125.8 MHz; $CDCl_3$): δ = 21.3 (1C, -**C**H$_3$), 26.8 (1C, -C(O)-**C**H$_3$), 127.1 (2C, -**C**H-CH-C(COMe)-), 127.3 (2C, -C(Me)-CH-**C**H-), 129.1 (2C, -**C**H-C(COMe)-), 129.8 (2C, -C(Me)-**C**H-), 135.8 (1C, -**C**(COMe)-), 137.1 (1C, -**C**(Me)-), 138.4 (1C, -**C**-C-CH-CH-C(COMe)-), 145.9 (1C, -**C**-CH-CH-C(COMe)-), 197.9 (1C, -**C**(O)-) ppm. HRMS (ESI$^+$) calcd. for $C_{15}H_{15}O^+$: 211.1117; found: 211.1115.

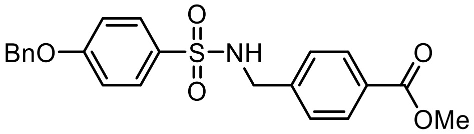

*Compound **1x.*** Methyl 4-(((4-(benzyloxy)phenyl)sulfonamido)methyl)benzoate. A solution of 4-(benzyloxy)benzenesulfonyl chloride (282.8 mg, 1 mmol, 1 eq.), methyl 4-(aminomethyl)benzoate hydrochloride (201.7 mg, 1 mmol, 1 eq.) and *N*,*N*-diisopropylethylamine (284.4 mg, 383.2 µL, 2.2 mmol, 2.2 eq.) in DCM (4 mL) was stirred for 2 h at room temperature. After dilution with ethyl acetate (20 mL) the solution was washed with water (10 mL), aqueous hydrochloric acid (1 M, 10 mL) and brine (10 mL). The organic layer was dried with sodium sulfate, filtered and the solvent was evaporated. The crude product (395.8 mg, 0.96 mmol, 96%) was used without further purification.
^{1}H NMR (500 MHz; $CDCl_3$): δ = 3.89 (s, 3H, -OC**H**$_3$), 4.17 (d, 2H, $^3J_{HH}$ = 6.3 Hz, -NH-C**H**$_2$-), 4.95 (t, 1H, $^3J_{HH}$ = 6.3 Hz, -N**H**-CH$_2$-), 5.12 (s, 2H, -O-C**H**$_2$-), 7.03 (d, 2H, $^3J_{HH}$ = 8.9 Hz, -C(OBn)-C**H**-), 7.27 (d, 2H, $^3J_{HH}$ = 8.2 Hz, -CH$_2$-C-C**H**-), 7.35 - 7.44 (m, 5H, -O-CH$_2$-C$_6$**H**$_5$), 7.78 (d, 2H, $^3J_{HH}$ = 8.9 Hz, -C**H**-C(SO$_2$)-), 7.93 (d, 2H, $^3J_{HH}$ = 8.2 Hz, -C**H**-C(COOMe)-) ppm. ^{13}C NMR (125.8 MHz; $CDCl_3$): δ = 47.0 (1C, -NH-**C**H$_2$-), 52.3 (1C, -O**C**H$_3$), 70.5 (1C, -O-**C**H$_2$-), 115.3 (2C, -C(OBn)-**C**H-), 127.6 (2C, -O-CH$_2$-**C**$_6$H$_5$), 127.8 (2C, -CH$_2$-C-**C**H-), 128.5 (1C, -O-CH$_2$-**C**$_6$H$_5$), 128.9 (2C, -O-CH$_2$-**C**$_6$H$_5$), 129.4 (2C, -**C**H-C(SO$_2$)-), 129.8 (1C, -**C**(COOMe)-), 130.1 (2C, -**C**H-C(COOMe)-), 131.7 (1C, -**C**(SO$_2$)-), 135.9 (1C, -O-CH$_2$-**C**$_6$H$_5$), 141.7 (1C, -CH$_2$-**C**-), 162.3 (1C, -**C**(OBn)-), 166.8 (1C, -**C**OOMe) ppm. HRMS (ESI$^+$) calcd. for $C_{22}H_{22}NO_5S^+$: 412.1206; found: 412.1208. HRMS (ESI$^-$) calcd. for $C_{22}H_{20}NO_5S^-$: 410.1068; found: 410.1068.

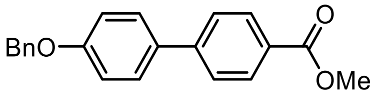

*Compound **2x.*** Methyl 4'-(benzyloxy)-[1,1'-biphenyl]-4-carboxylate. A solution of methyl 4-(((4-(benzyloxy)phenyl)sulfonamido)methyl)benzoate (**1x**) (22.4 mg, 54.4 µmol) in MeOH (10 mL) was loaded on the photo reactor with a flow rate of 1 mL/min (MeOH) and irradiated with UV light (254 nm) at room temperature. The solvent fraction containing the photoproduct was collected and the solvent was removed under reduced pressure. The residue was purified by open column chromatography (silica, R_f=0.76, DCM) to yield the title compound (9.5 mg, 29.8 µmol, 55%).

^{1}H NMR (500 MHz; $CDCl_3$): δ = 3.94 (s, 3H, -OC**H**$_3$), 5.13 (s, 2H, -O-C**H**$_2$-), 7.07 (d, 2H, $^3J_{HH}$ = 8.7 Hz, -C(OBn)-C**H**-), 7.26 - 7.47 (m, 5H, -O-CH$_2$-C$_6$**H**$_5$), 7.57 (d, 2H, $^3J_{HH}$ = 8.7 Hz, -C(OBn)-CH-C**H**-), 7.62 (d, 2H, $^3J_{HH}$ = 8.4 Hz, -C**H**-CH-C(COOMe)-), 8.08 (d, 2H, $^3J_{HH}$ = 8.4 Hz, -C**H**-C(COOMe)-) ppm. ^{13}C NMR (125.8 MHz; $CDCl_3$): δ = 52.2 (1C, -O**C**H$_3$), 70.2 (1C, -O-**C**H$_2$-), 115.4 (2C, -C(OBn)-**C**H-), 126.6 (2C, -**C**H-CH-C(COOMe)-), 127.6 (2C, -O-CH$_2$-**C**$_6$H$_5$), 128.2 (1C, -O-CH$_2$-**C**$_6$H$_5$), 128.4 (1C, -**C**(COOMe)-), 128.5 (2C, -C(OBn)-CH-**C**H-), 128.8 (2C, -O-CH$_2$-**C**$_6$H$_5$), 130.2 (2C, -**C**H-C(COOMe)-), 132.8 (1C, -C(OBn)-CH-CH-**C**-), 136.9 (1C, -O-CH$_2$-**C**$_6$H$_5$), 145.3 (1C, -**C**-CH-CH-C(COOMe)-), 159.2 (1C, -**C**(OBn)-), 167.2 (1C, -**C**OOMe) ppm. HRMS (ESI$^+$) calcd. for $C_{21}H_{19}O_3^+$: 319.1329; found: 319.1325.

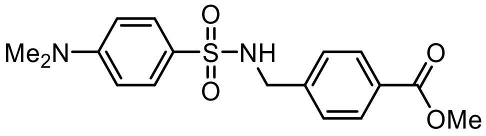

*Compound **1y.*** Methyl 4-(((4-(dimethylamino)phenyl)sulfonamido)methyl)benzoate. A solution of 4-(dimethylamino)benzenesulfonyl chloride (219.7 mg, 1 mmol, 1 eq.), methyl 4-(aminomethyl)benzoate hydrochloride (201.7 mg, 1 mmol, 1 eq.) and *N*,*N*-diisopropylethylamine (284.4 mg, 383.2 µL, 2.2 mmol, 2.2 eq.) in DCM (4 mL) was stirred for 2 h at room temperature. After dilution with ethyl acetate (20 mL) the solution was washed with water (10 mL), aqueous hydrochloric acid (1 M, 10 mL) and brine (10 mL). The organic layer was dried with sodium sulfate, filtered and the solvent was evaporated. The crude product was purified by recrystallization from ethanol/water (*v*/*v* = 1:1) to provide the title compound as a white crystalline compound (260 mg, 0.75 mmol, 75%).

^{1}H NMR (300 MHz; $CDCl_3$): δ = 3.05 (s, 6H, -N(C**H**$_3$)$_2$), 3.89 (s, 3H, -OC**H**$_3$), 4.14 (d, 2H, $^3J_{HH}$ = 6.4 Hz, -NH-C**H**$_2$-), 4.79 (t, 1H, $^3J_{HH}$ = 6.4 Hz, -N**H**-CH$_2$-), 6.66 - 6.69 (m, 2H, -C(N(CH$_3$)$_2$)-C**H**-), 7.27 - 7.30 (m, 2H, -CH$_2$-C-C**H**-), 7.67 - 7.70 (m, 2H, -C(N(CH$_3$)$_2$)-CH-C**H**-), 7.91 - 7.94 (m, 2H, -C**H**-C(COOMe)-) ppm. ^{13}C NMR (75.5 MHz; $CDCl_3$): δ = 40.3 (2C, -C(N(**C**H$_3$)$_2$)-), 46.9 (1C, -**C**H$_2$-), 52.3 (1C, -O**C**H$_3$), 111.3 (2C, -C(N(CH$_3$)$_2$)-**C**H-), 125.0 (1C, -**C**(SO$_2$)-), 127.8 (2C, -CH$_2$-C-**C**H-), 129.1 (2C, -C(N(CH$_3$)$_2$)-CH-**C**H-), 129.6 (1C, -**C**(COOMe)-), 130.0 (2C, -**C**H-C(COOMe)-), 142.1 (1C, -CH$_2$-**C**-), 152.9 (1C, -C(N(CH$_3$)$_2$)-), 166.9 (1C, -**C**OOMe) ppm. HRMS (ESI$^+$) calcd. for $C_{17}H_{21}N_2O_4S^+$: 349.1217; found: 349.1211. HRMS (ESI$^-$) calcd. for $C_{17}H_{19}N_2O_4S^-$: 347.1071; found: 347.1067.

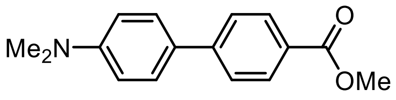

*Compound **2y.*** Methyl 4'-(dimethylamino)-[1,1'-biphenyl]-4-carboxylate. A solution of methyl 4´-(dimethylamino)phenyl)sulfonamido)methyl)benzoate (**1y**) (21.1 mg, 60.6 µmol) in MeCN (10 mL) was loaded on the photo reactor with a flow rate of 0.5 mL/min (MeCN) and irradiated with UV light (254 nm) at room temperature. The solvent fraction containing the photoproduct was collected and the solvent was removed under reduced pressure. The residue was purified by open column chromatography (silica, R_f=0.75, DCM) to yield the title compound (5.5 mg, 21.6 µmol, 36%).

^{1}H NMR (300 MHz; $CDCl_3$): δ = 3.04 (s, 6H, -N(C**H**$_3$)$_2$), 3.95 (s, 3H, -OC**H**$_3$), 6.86 - 6.88 (m, 2H, -C(N(CH$_3$)$_2$)-C**H**-), 7.57 - 7.66 (m, 4H, -C(N(CH$_3$)$_2$)-CH-C**H**- and -C**H**-CH-C(COOMe)-), 8.06 - 8.09 (m, 2H, -C**H**-C(COOMe)-) ppm. ^{13}C NMR (75.5 MHz; $CDCl_3$): δ = 40.8 (2C, -C(N(**C**H$_3$)$_2$)-), 52.1 (1C, -O**C**H$_3$), 113.0 (2C, -C(N(CH$_3$)$_2$)-**C**H-), 126.0 (2C, -**C**H-CH-C(COOMe)-), 127.6 (1C, -**C**(COOMe)-), 128.1 (2C, -C(N(CH$_3$)$_2$)-CH-**C**H-), 130.2 (2C, -**C**H-C(COOMe)-), 130.4 (1C, -C(N(CH$_3$)$_2$)-CH-CH-**C**-), 145.6 (1C, -**C**-CH-CH-C(COOMe)-), 150.5 (1C, -**C**(N(CH$_3$)$_2$)-), 167.3 (1C, -**C**OOMe) ppm. HRMS (ESI$^+$) calcd. for $C_{16}H_{18}O_2N^+$: 256.1332; found: 256.1332.

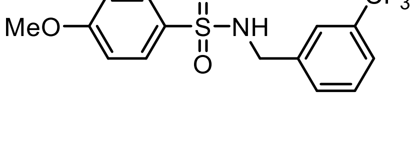

Compound ***1z.*** 4-Methoxy-*N*-(3-(trifluoromethyl)benzyl)benzenesulfonamide. A solution of 4-methoxybenzenesulfonyl chloride (200 mg, 0.97 mmol, 1 eq.), (3-(trifluoromethyl)phenyl)methanamine (170 mg, 138.7 µL, 0.97 mmol, 1 eq.) and *N*,*N*-diisopropylethylamine (150.1 mg, 202.3 µL, 1.16 mmol, 1.2 eq.) in DCM (4 mL) was stirred for 1 h at room temperature. The solution was washed with water (4 mL), aqueous hydrochloric acid (1 M, 4 mL) and brine (4 mL). The organic layer was dried with sodium sulfate, filtered and the solvent was evaporated. The crude product was obtained as white a solid (254.5 mg, 0.74 mmol, 76%) and used without further purification.

^{1}H NMR (500 MHz, $CDCl_3$) δ = 3.81 - 3.91 (s, 3H, O-C$\mathbf{H_3}$), 4.14 - 4.25 (d, $^3J_{HH}$ = 4.2 Hz, 2H, NH-C$\mathbf{H_2}$), 4.91 - 5.07 (s, 1H, N**H**), 6.89 - 7.00 (m, 2H, O-C-(C**H**)$_2$), 7.34 - 7.45 (m, 3H, C**H**-C-CH-C**H**-C**H**), 7.45 - 7.53 (d, $^3J_{HH}$ = 7.4 Hz, 1H, CF_3-C-C**H**-CH), 7.72 - 7.81 (m, 2H, O-C-(CH-C**H**)$_2$) ppm. ^{13}C NMR (126 MHz, $CDCl_3$) δ = 46.8 (1C, **C**H_2-NH), 55.8 (1C, O-**C**H_3), 114.5 (2C, O-C-(CH-**C**H)$_2$), 120.31 - 127.82 (1C, q, $^1J_{CF}$ = 272.4 Hz, **C**F_3), 124.58 - 124.70 (1C, q, $^3J_{CF}$ = 3.7 Hz, CH_2-C-**C**H-C), 124.74-124.86 (1C, q, $^3J_{CF}$ = 3.7 Hz, CF_3-C-**C**H-CH), 129.3 (1C, CF_3-C-CH-**C**H), 129.4 (2C, O-C-(**C**H)$_2$), 130.66-131.55 (1C, q, $^2J_{CF}$ = 32.0 Hz, C-CF_3), 131.3 (1C, CH_2-C-**C**H-CH), 131.4 (1C, SO_2-C), 137.6 (1C, CH_2-**C**), 163.2 (1C, H_3C-O-**C**) ppm. ^{19}F NMR (471 MHz, $CDCl_3$): -62.6 (s, 3F) ppm. HRMS (ESI$^+$) calcd. for $C_{15}H_{15}F_3NO_3S^+$: 346.0719; found: 346.0721. HRMS (ESI$^-$) calcd. for $C_{15}H_{13}F_3NO_3S^-$: 344.0574; found: 344.0576.

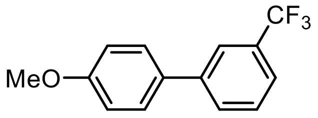

Compound ***2z.*** 4'-Methoxy-3-(trifluoromethyl)-1,1'-biphenyl. A solution of 4-methoxy-*N*-(3-(trifluoromethyl)benzyl)benzenesulfonamide (**1z**) (20 mg, 57.9 µmol) in MeCN (10 mL) was loaded on the photo reactor with a flow rate of 2 mL/min and irradiated with UV light (254 nm) at room temperature. The solvent fraction containing the photoproduct was collected and the solvent was removed under reduced pressure. The residue was purified by open column chromatography (silica, R_f=0.80, DCM) to yield the title compound (4.7 mg, 18.5 µmol, 32%).

^{1}H NMR (500 MHz, $CDCl_3$) δ = 7.76-7.82 (s, 1H, C-C**H**-C-CF_3), 7.68-7.75 (d, $^3J_{HH}$ = 7.1 Hz, 1H, C**H**-CH-C-CF_3), 7.48-7.60 (m, 4H, C**H**-C**H**-CH-C-CF_3 & C-C-(C**H**)$_2$), 6.93-7.05 (m, 2H, O-C-(C**H**)$_2$), 3.80-3.92 (s, 3H, O-C$\mathbf{H_3}$) ppm. ^{13}C NMR (126 MHz, $CDCl_3$) δ = 55.5 (1C, O-**C**H_3), 114.6 (2C, O-C-(**C**H)$_2$), 123.38–123.50 (1C, q, $^3J_{CF}$ = 3.9 Hz, CF_3-C-**C**H-CH), 123.52–123.66 (1C, q, $^3J_{CF}$ = 3.9 Hz, CF_3-C-**C**H-C), 120.99–127.85 (1C, q, $^1J_{CF}$ = 272.6 Hz, **C**F_3), 128.4 (2C, C-C-(**C**H)$_2$), 129.3 (1C, CF_3-C-CH-**C**H-CH), 130.1 (1C, CF_3-C-CH-CH-**C**H), 130.78-131.72 (1C, q, $^2J_{CF}$ = 31.6 Hz, **C**-CF_3), 132.4 (1C, F_3C-C-CH-C-**C**), 141.7 (1C, F_3C-C-CH-**C**-C), 159.9 (1C, **C**-O-CH_3) ppm. ^{19}F NMR (471 MHz, $CDCl_3$): -62.5 (s, 3F) ppm. HRMS (ESI$^+$) calcd. for $C_{14}H_{12}F_3O^+$: 253,0835; found: 253.0835. HRMS (ESI$^-$) calcd. for $C_{14}H_{10}F_3O^-$: 251,0689; found: 251.0689.

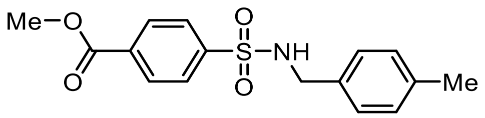

Compound ***1aa.*** Methyl 4-(*N*-(4-methylbenzyl)sulfamoyl)benzoate. A solution of 4-tolylmethanamine (50 mg, 53 µL, 0.41 mmol, 1.1 eq.), methyl 4-(chlorosulfonyl)benzoate (88 mg, 0.38 mmol, 1 eq.) and *N*,*N*-diisopropylethylamine (73 mg, 98 µL, 0.57 mmol, 1.5 eq.) in DCM (2 mL) was stirred for 1 h at room temperature. The solution was washed with water (2 mL), $NaHCO_3$ solution (2 mL) and aqueous hydrochloric acid (1 M, 2 mL). The organic layer was dried with sodium sulfate, filtered and the solvent was evaporated. The residue was purified by open column chromatography (silica, R_f=0.20, DCM) to yield the title compound (104 mg, 0.33 mmol, 87%) as a white solid.

^{1}H NMR (500 MHz, $CDCl_3$) δ = 2.27 - 2.32 (s, 3H, C-C$\mathbf{H_3}$), 3.95 - 3.99 (s, 3H, -O-C$\mathbf{H_3}$), 4.10 - 4.15 (d, $^3J_{HH}$ = 5.8 Hz, 2H, -C$\mathbf{H_2}$-), 4.77 - 4.83 (d, $^3J_{HH}$ = 6.2 Hz, 1H, N**H**), 7.02 - 7.10 (m, 4H, C**H**-C**H**-C-CH_3), 7.88 - 7.94 (d, $^3J_{HH}$ = 8.5 Hz, 2H, C**H**-C-SO_2), 8.11–8.17 (d, $^3J_{HH}$ = 8.5 Hz, 2H, C**H**-C-C(=O)) ppm. ^{13}C NMR (126 MHz, $CDCl_3$) δ = 21.2 (1C, C-**C**H_3), 47.3 (1C, HN-**C**H_2), 52.8 (1C, O-**C**H_3), 127.3 (2C, **C**H-C-SO_2), 128.0 (2C, **C**H-CH-C-CH_3), 129.6 (2C, **C**H-C-CH_3), 130.4 (2C, **C**H-C-C(=O)), 132.9 (1C, CH_2-**C**), 133.9 (1C, CH-**C**-SO_2), 138.1 (1C, **C**-CH_3), 144.2 (1C, CH-**C**-C(=O)), 165.8 (1C, O-**C**(=O)-C) ppm. HRMS (ESI$^+$) calcd. for $C_{16}H_{18}NO_4S^+$: 320.0951; found: 320.0949. HRMS (ESI$^-$) calcd. for $C_{16}H_{16}NO_4S^-$: 318.0806; found: 318.0805.

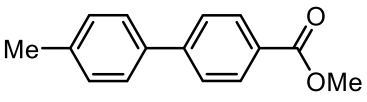

Compound ***2a (=2aa).*** Methyl 4'-methyl-[1,1'-biphenyl]-4-carboxylate. A solution of methyl 4-(*N*-(4-methylbenzyl)sulfamoyl)benzoate (**1aa**) (20.2 mg, 0.063 mmol) in MeCN (10 mL) was loaded on the photo reactor with a flow rate of 5 mL/min and irradiated with UV light (254 nm) at room temperature. The solvent fraction containing the photoproduct was collected and the solvent was removed

under reduced pressure. The residue was purified by open column chromatography (silica, R_f=0.75, DCM) to yield the title compound (4.7 mg, 0.021 µmol, 33%) as a white solid.
NMR and HRMS spectra are equivalent to those of biphenyl **2a**.[3]

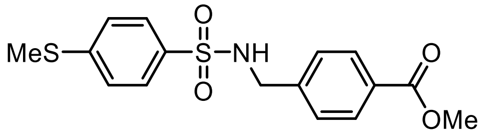

*Compound **1ab**.* Methyl 4-(((4-(methylthio)phenyl)sulfonamido)methyl)benzoate. A solution of 4-(methylthio)benzenesulfonyl chloride (222.7 mg, 1 mmol, 1 eq.), methyl 4-(aminomethyl)benzoate hydrochloride (201.7 mg, 1 mmol, 1 eq.) and *N*,*N*-diisopropylethylamine (284.4 mg, 383.2 µL, 2.2 mmol, 2.2 eq.) in DCM (4 mL) was stirred for 2 h at room temperature. After dilution with ethyl acetate (20 mL) the solution was washed with water (10 mL), aqueous hydrochloric acid (1 M, 10 mL) and brine (10 mL). The organic layer was dried with sodium sulfate, filtered and the solvent was evaporated. The crude product was purified by recrystallization from ethanol/water (*v*/*v* = 1:1) to provide the title compound as a white crystalline compound (307 mg, 0.88 mmol, 88%).
^{1}H NMR (300 MHz; $CDCl_3$): δ = 2.51 (s, 3H, -SC**H**$_3$), 3.89 (s, 3H, -OC**H**$_3$), 4.16 (d, 2H, $^3J_{HH}$ = 5.1 Hz, -NH-C**H**$_2$-), 5.19 (t, 1H, -N**H**-CH$_2$-), 7.24 - 7.27 (m, 4H, -C**H**-CH-C(COOMe)- and -C(SMe)-C**H**-), 7.69 - 7.72 (m, 2H, -C(SMe)-CH-C**H**-), 7.89 - 7.92 (m, 2H, -C**H**-C(COOMe)-) ppm. ^{13}C NMR (75.5 MHz; $CDCl_3$): δ = 14.9 (1C, -S**C**H$_3$), 46.9 (1C, -**C**H$_2$-), 52.3 (1C, -O**C**H$_3$), 125.5 (2C, -C(SMe)-**C**H-), 127.5 (2C, -C(SMe)-CH-**C**H-), 127.8 (2C, -**C**H-CH-C(COOMe)-), 129.7 (1C, -**C**(COOMe)-), 130.0 (2C, -**C**H-C(COOMe)-), 135.4 (1C, -**C**-SO$_2$-), 141.6 (1C, -CH$_2$-**C**-), 146.1 (1C, -**C**(SMe)-), 166.8 (1C, -**C**OOMe) ppm. HRMS (ESI$^+$) calcd. for $C_{16}H_{18}NO_4S_2^+$: 352.0672; found: 352.0662. HRMS (ESI$^-$) calcd. for $C_{16}H_{16}NO_4S_2^-$: 350.0526; found: 350.0520.

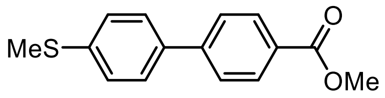

*Compound **2ab**.* Methyl 4'-(methylthio)-[1,1'-biphenyl]-4-carboxylate. A solution of methyl 4-(((4-(methylthio)phenyl)sulfonamido)methyl)benzoate (**1ab**) (25.0 mg, 71.2 µmol) in MeOH (10 mL) was loaded on the photo reactor with a flow rate of 1 mL/min (MeOH) and irradiated with UV light (254 nm) at room temperature. The solvent fraction containing the photoproduct was collected and the solvent was removed under reduced pressure. The residue was purified by open column chromatography (silica, R_f=0.40, DCM) and recrystallization from diethyl ether/petrol ether (*v*/*v* = 1:1) to yield the title compound (4.8 mg, 18.6 µmol, 26%).
^{1}H NMR (300 MHz; $CDCl_3$): δ = 2.53 (s, 3H, -SC**H**$_3$), 3.94 (s, 3H, -OC**H**$_3$), 7.32 - 7.35 (m, 2H, -C(SMe)-C**H**-), 7.57 - 7.54 (m, 2H, -C(SMe)-CH-C**H**-), 7.62 - 7.65 (m, 2H, -C**H**-CH-C(COOMe)-), 8.08 - 8.11 (m, 2H, -C**H**-C(COOMe)-) ppm. ^{13}C NMR (75.5 MHz; $CDCl_3$): δ = 15.8 (1C, -S**C**H$_3$), 52.3 (1C, -O**C**H$_3$), 126.8 (2C, -**C**H-CH-C(COOMe)-), 126.9 (2C, -C(SMe)-**C**H-), 127.7 (2C, -C(SMe)-CH-**C**H-), 128.9 (1C, -**C**(COOMe)-), 130.3 (2C, -**C**H-C(COOMe)-), 136.7 (1C, -C(SMe)-CH-CH-**C**-), 139.1 (1C, -**C**(SMe)-), 145.1 (1C, -**C**-CH-CH-C(COOMe)-), 167.1 (1C, -**C**OOMe) ppm. HRMS (ESI$^+$) calcd. for $C_{15}H_{15}O_2S^+$: 259.0787; found: 259.0789. HRMS (ESI$^-$) calcd. for $C_{15}H_{13}O_2S^-$: 257.0642; found: 257.0643.

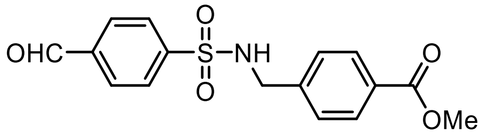

*Compound **1ac**.* Methyl 4-(((4-formylphenyl)sulfonamido)methyl)benzoate. A solution of 4-formylbenzenesulfonyl chloride (204.6 mg, 1 mmol, 1 eq.), methyl 4-(aminomethyl)benzoate hydrochloride (201.7 mg, 1 mmol, 1 eq.) and *N*,*N*-diisopropylethylamine (284.4 mg, 383.2 µL, 2.2 mmol, 2.2 eq.) in DCM (4 mL) was stirred for 1 h at room temperature. After dilution with ethyl acetate (20 mL) the solution was washed with water (10 mL), aqueous hydrochloric acid (1 M, 10 mL) and brine (10 mL). The organic layer was dried with sodium sulfate, filtered and the solvent was evaporated. The crude product was purified by recrystallization from ethanol/water (*v*/*v* = 1:1) to provide the title compound as a white crystalline compound (291.7 mg, 0.88 mmol, 88%).
^{1}H NMR (300 MHz; CD_3CN): δ = 3.84 (s, 3H, -OC**H**$_3$), 4.19 (d, 2H, $^3J_{HH}$ = 6.3 Hz, -C**H**$_2$-), 6.31 (t, 1H, $^3J_{HH}$ = 6.3 Hz, -N**H**-), 7.31 (d, 2H, $^3J_{HH}$ = 8.3 Hz, -CH$_2$-C-C**H**-), 7.86 (d, 2H, $^3J_{HH}$ = 8.3 Hz, -C**H**-C-COOMe), 7.94–8.01 (m, 4H, OHC-C-C**H**- and -C**H**-C-SO$_2$-), 10.06 (s, 1H, -C**H**O) ppm. ^{13}C NMR (75.5 MHz; CD_3CN): δ = 47.2 (1C, -**C**H$_2$-), 52.6 (1C, -O**C**H$_3$), 128.5 (2C, -**C**H-C-SO$_2$-), 128.8 (2C, -CH$_2$-C-**C**H-), 130.3 (2C, -**C**H-C-COOMe), 130.4 (1C, -**C**-COOMe), 131.0 (2C, OCH-C-C**H**-), 139.9 (1C, OHC-**C**-), 143.4 (1C, -CH$_2$-**C**-), 146.3 (1C, -**C**-SO$_2$-), 167.3 (1C, -**C**OOMe), 192.8 (1C, -**C**HO) ppm. HRMS (ESI$^+$) calcd. for $C_{16}H_{16}NO_5S^+$: 332.0744; found: 332.0746. HRMS (ESI$^-$) calcd. for $C_{16}H_{14}NO_5S^-$: 332.0598; found: 332.0602.

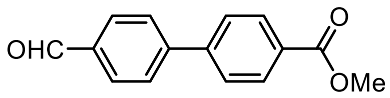

Compound ***2ac.*** Methyl 4'-formyl-[1,1'-biphenyl]-4-carboxylate. A solution of methyl 4-(((4-formylphenyl)sulfonamido)methyl)benzoate (**1ac**) (18.0 mg, 54.1 µmol) in MeOH (10 mL) was loaded on the photo reactor with a flow rate of 2 mL/min (MeOH) and irradiated with UV light (254 nm) at room temperature. The solvent fraction containing the photoproduct was collected and the solvent was removed under reduced pressure. The residue was purified by open column chromatography (silica, R_f=0.65, DCM) to yield the title compound (2.4 mg, 10 µmol, 18%).
^{1}H NMR (300 MHz; $CDCl_3$): δ = 3.96 (s, 3H, -OC**H**$_3$), 7.69 - 7.72 (m, 2H, -C**H**-C-C-C**H**-), 7.77 - 7.80 (m, 2H, -C**H**-C-C-C**H**-), 7.97 - 8.00 (m, 2H, OHC-C-C**H**-), 8.10 - 8.16 (m, 2H, -C**H**-C-COOMe), 10.08 (s, 1H, -C**H**O) ppm. ^{13}C NMR (75.5 MHz; $CDCl_3$): δ = 52.4 (1C, -O**C**H$_3$), 127.5 (2C, -**C**H-C-C-**C**H-), 128.1 (2C, -**C**H-C-C-**C**H-), 130.2 (1C, -**C**-COOMe), 130.4 (2C, -**C**H-C-COOMe), 130.5 (2C, OHC-C-**C**H-), 136.0 (1C, OHC-**C**-), 144.2 (1C, OHC-C-CH-CH-C-**C**-), 146.0 (1C, OHC-C-CH-CH -**C**-C-), 166.9 (1C, -**C**OOMe), 191.2 (1C, -**C**HO) ppm. HRMS (ESI$^+$) calcd. for $C_{15}H_{13}O_3^+$: 241.0859; found: 241.0857.

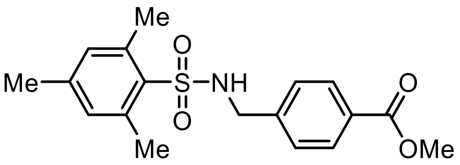

Compound ***1ad.*** Methyl 4-(((2,4,6-trimethylphenyl)sulfonamido)methyl)benzoate. A solution of 2,4,6-trimethylbenzenesulfonyl chloride (218.7 mg, 1 mmol, 1 eq.), methyl 4-(aminomethyl)benzoate hydrochloride (201.7 mg, 1 mmol, 1 eq.) and *N*,*N*-diisopropylethylamine (284.4 mg, 383.2 µL, 2.2 mmol, 2.2 eq.) in DCM (4 mL) was stirred for 1 h at room temperature. After dilution with ethyl acetate (20 mL) the solution was washed with water (10 mL), aqueous hydrochloric acid (1 M, 10 mL) and brine (10 mL). The organic layer was dried with sodium sulfate, filtered and the solvent was evaporated. The crude product was purified by recrystallization from ethanol/water (*v*/*v* = 1:1) to provide the title compound as a white crystalline compound (287.5 mg, 0.83 mmol, 83%).
^{1}H NMR (300 MHz, $CDCl_3$) δ = 2.27–2.33 (s, 3H, **H$_3$**C-C-CH-C-C), 2.59–2.66 (s, 6H, **H$_3$**C-C-C), 3.87–3.93 (s, 3H, O-C**H$_3$**), 4.10–4.19 (d, $^3J_{HH}$ = 5.3, 2H, C**H$_2$**), 4.73–4.90 (s, 1H, N**H**), 6.91–6.98 (s, 2H, H$_3$C-C-C**H**-C-CH$_3$), 7.19–7.26 (d, $^3J_{HH}$ = 8.7, 2H, C**H**-CH-C-CO), 7.87–7.97 (d, $^3J_{HH}$ = 8.3, 2H, C**H**-C-CO) ppm. ^{13}C NMR (75 MHz, $CDCl_3$) δ = 21.1 (1C, H$_3$**C**-C-CH-C-C), 23.1 (2C, H$_3$**C**-C-C), 46.6 (1C, **C**H$_2$), 52.3 (1C, O-**C**H$_3$), 127.8 (2C, CH$_2$-C-**C**H), 129.8 (1C, **C**-SO$_2$), 130.0 (2C, H$_3$C-C-**C**H-C-C), 132.2 (2C, CH$_2$-C-CH-**C**H), 133.6 (1C, H$_3$C-**C**-CH-C-C), 139.2 (2C, H$_3$C-**C**-C-SO$_2$), 141.7 (1C, CH$_2$-**C**), 142.7 (1C, **C**-CO), 166.8 (1C, C-**C**O) ppm. HRMS (ESI$^+$) calcd. for $C_{18}H_{22}NO_4S^+$: 384.1264; found: 348.1265.

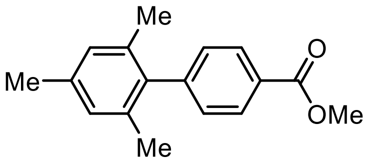

Compound ***2ad.*** Methyl 2',4',6'-trimethyl-[1,1'-biphenyl]-4-carboxylate. A solution of methyl 4-(((2,4,6-trimethylphenyl)sulfonamido)methyl)benzoate (**1ad**) (20.5 mg, 82.9 µmol) in MeOH (10 mL) was loaded on the photo reactor with a flow rate of 1 mL/min (MeOH) and irradiated with UV light (254 nm) at room temperature. The solvent fraction containing the photoproducts was collected and the solvent was removed under reduced pressure. The desired photoproduct was observed by HPLC-HRMS and GCMS in traces.
HRMS (ESI$^+$) calcd. for $C_{17}H_{19}O_2^+$: 255.1380; found: 255.1382; GCMS (EI, 70 eV) m/z (%): 254 (100), 239 (9), 223 (29), 195 (52), 180 (30), 165 (35), 152 (11).

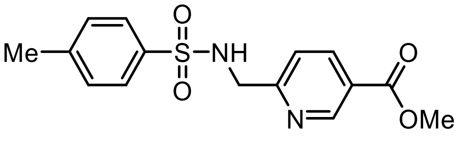

Compound ***1ae.*** Methyl 6-(((4-methylphenyl)sulfonamido)methyl)nicotinate. A solution of methyl 6-(aminomethyl)nicotinate hydrochloride (95,3 mg; 0,47 mmol; 1 eq.) in 1,4-dioxan (500 µL) was added to a suspension of 4-methylbenzenesulfonyl chloride (101,3 mg; 0,53 mmol; 1,1 eq.) and *N*,*N*-diisopropylethylamine (153 mg; 207 µL; 1,19 mmol; 2,5 eq.) 1,4-dioxan (500 µL). The

mixture was sonicated for 5 h at room temperature. After dilution with ethyl acetate (30 mL) the solution was washed with NH_4Cl solution (30 mL, saturated) and brine (30 mL). The organic layer was dried with Na_2SO_4, filtered and the solvent was evaporated under reduced pressure. The crude product was crystallized from ethanol/water (*v*/*v* = 3/2) to provide the title compound as a white crystalline solid (75.4 mg; 0.235 mmol; 50%).
^{1}H NMR (500 MHz; $CDCl_3$): δ = 2.38 (s; 3H; C-C**H**$_3$); 3.95 (s; 3H; -OC**H**$_3$); 4.34 (d; 2H; $^3J_{HH}$ = 5.7 Hz; -C**H**$_2$-); 5.94 (t; 1H; $^3J_{HH}$ = 5.5 Hz; -N**H-**); 7.22–7.27 (m; 2H; -C**H**-C-CH$_3$); 7.31–7.35 (m; 2H; -CH$_2$-C-C**H**); 7.70–7.76 (m; 2H; -C**H**-C-SO$_2$-); 8.21–8.27 (m; 1H; CH-C**H**-C-(C=O)-); 9.01–9.06 (m; 1H; -C**H**=N-) ppm. ^{13}C NMR (125.8 MHz; $CDCl_3$): δ = 21.5 (1C; C-**C**H$_3$); 47.2 (1C; -**C**H$_2$-); 52.5 (1C; -O**C**H$_3$); 121.8 (1C; -CH$_2$-C-**C**H-); 125.3 (1C; -**C**-(C=O)-); 127.2 (2C; -SO$_2$-C-**C**H-); 129.7 (2C; -**C**H-C-CH$_3$); 136.4 (1C; **C**-SO$_2$); 138.3 (1C; -**C**H-C-CH$_2$-); 143.6 (1C; -**C**-CH$_3$-); 149.7 (1C; -**C**H=N-); 159.1 (1C; -**C**-CH$_2$-); 165.1 (1C; -**C**=O-) ppm. HRMS (ESI$^+$) calcd. for $C_{15}H_{17}O_4N_2S^+$: 321.0904; found: 321.0901.

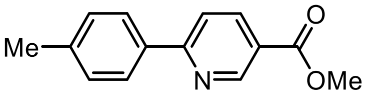

*Compound **2ae.*** Methyl-6-(*p*-tolyl)nicotinate. A solution of methyl 6-(((4-methylphenyl)sulfonamido)methyl)nicotinate (**1ae**) (20,1 mg; 62,7 µmol) in MeCN (50 mL) was loaded on the photo reactor with a flow rate of 1 mL/min and irradiated with UV light (254 nm) at room temperature. The solvent fraction containing the photoproduct was collected and the solvent was removed under reduced pressure. The residue was purified by open column chromatography (silica, R_f=0.22, DCM with 1% *N*,*N*-diisopropylethylamine) to yield the title compound (5,2 mg; 22,9 µmol; 37%) as a white solid.
^{1}H NMR (500 MHz; $CDCl_3$): δ = 2.42 (s; 3H; C-C**H**$_3$); 3.97 (s; 3H; -OC**H**$_3$); 7.29–7.33 (m; 2H; -C**H**-C-CH$_3$); 7.78–7.82 (m; 1H; -C**H**-CH-C-(C=O)-); 7.95–8.00 (m; 2H; -C**H**-CH-C-CH$_3$); 8.32–8.37 (m; 1H; -C**H**-C-(C=O)-); 9.25–9.29 (m; 1H; -C**H**=N-) ppm. ^{13}C NMR (125.8 MHz; $CDCl_3$): δ = 21.4 (1C; C-**C**H$_3$); 52.3 (1C; -O**C**H$_3$); 119.7 (1C; -**C**H-CH-C-(C=O)-); 124.0 (1C; -**C**-(C=O)-); 127.3 (2C; -**C**H-CH-C-CH$_3$); 129.7 (2C; -**C**H-C-CH$_3$); 135.0 (1C; -**C**-C=N-); 138.1 (1C; -CH-**C**H-C-(C=O)-); 140.5 (1C; -**C**-CH$_3$); 150.6 (1C; -**C**H=N-); 160.7 (1C; -C-**C**=N-); 165.7 (1C; -**C**=O-) ppm. HRMS (ESI$^+$) calcd. for $C_{14}H_{14}O_2N^+$: 228.1019; found: 228.1016.

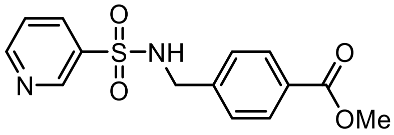

*Compound **1af.*** Methyl -4-((pyridin-3-sulfonamido)methyl)benzoate. A solution of pyridine-3-sulfonyl chloride (194,6 mg; 1 mmol; 1 eq.), methyl 4-(aminomethyl)benzoate hydrochloride (201.7 mg, 1 mmol, 1 eq.) and *N*,*N*-diisopropylethylamine (284.4 mg, 383.2 µL, 2.2 mmol, 2.2 eq.) in DCM (4 mL) was stirred for 1 h at room temperature. After dilution with ethyl acetate (20 mL) the solution was washed with water (20 mL), aqueous hydrochloric acid (1 M, 20 mL) and brine (20 mL). The organic layer was dried with sodium sulfate, filtered and the solvent was evaporated. The crude product was used without further purification (135,6 mg; 0,44 mmol; 44%).
^{1}H NMR (300 MHz; $CDCl_3$): δ = 3.90 (s; 3H; -OC**H**$_3$); 4.27 (d; 2H; $^3J_{HH}$ = 6.1 Hz; -C**H**$_2$-); 5.42 (t; 1H; $^3J_{HH}$ = 6.1 Hz; -N**H-**); 7.13–7.19 (m; 2H; -SO$_2$-C-C**H**-); 7.27 (d; 2H; $^3J_{HH}$ = 8.3 Hz; -CH$_2$-C-C**H**-); 7.43 (dd; 1H; $^3J_{HH}$ = 7.9 Hz; $^4J_{HH}$ = 4.8 Hz; -C-CH-C**H**-); 7.93 (d; 2H; $^3J_{HH}$ = 8.3 Hz; -C**H**-C-COOMe); 8.09 (dt; 1H; $^3J_{HH}$ = 8.0 Hz; $^4J_{HH}$ = 1.9 Hz; -C-C**H**-CH-); 8.77 (dd; 1H; $^3J_{HH}$ = 4.8 Hz; $^4J_{HH}$ = 1.4 Hz; -C-CH-N-C**H**-); 9.03 (d; 1H; $^3J_{HH}$ = 2.0 Hz; -C-C**H**-N-) ppm. ^{13}C NMR (75.5 MHz; $CDCl_3$): δ = 47.0 (1C; -**C**H$_2$-); 52.4 (1C; -O**C**H$_3$); 123.9 (1C; -C-CH-**C**H-); 127.9 (2C; -CH$_2$-C-**C**H-); 130.1 (1C; -**C**-COOMe); 130.2 (2C; -**C**H-C-COOMe); 134.9 (1C; -C-**C**H-CH-); 137.0 (1C; -**C**-SO$_2$-); 141.0 (1C; -CH$_2$-**C**-); 148.0 (1C; -C-**C**H-N-); 153.3 (1C; -C-CH-N-**C**H-); 166.7 (1C; -C-**C**OOMe) ppm. HRMS (ESI$^+$) calcd. for $C_{14}H_{15}N_2O_4S^+$: 307.0747; found: 307.0743. HRMS (ESI$^-$) calcd. for $C_{14}H_{13}N_2O_4S^-$: 305.0602; found: 305.0598.

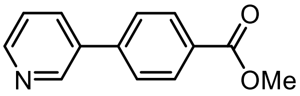

*Compound **2af.*** Methyl-4-(pyridin-3-yl)benzoat. A solution of methyl methyl-4-((pyridin-3-sulfonamido)methyl)benzoate (**1af**) (17,0 mg; 56,0 µmol) in MeCN (10 mL) was loaded on the photo reactor with a flow rate of 1 mL/min and irradiated with UV light (254 nm) at room temperature. The solvent fraction containing the photoproduct was collected and the solvent was removed under reduced pressure. The residue was purified by open column chromatography (silica, R_f=0.45, chloroform/MeOH 95:5) to yield the title compound (10 mg; 46,9 µmol; 85%) as a white solid.
^{1}H NMR (300 MHz; $CDCl_3$): δ = 3.95 (s; 3H; -OC**H**$_3$); 7.40 (dd; 1H; $^3J_{HH}$ = 7.9 Hz; $^3J_{HH}$ = 4.9 Hz; -CH-C**H**-CH-N-); 7.64–7.68 (d; 2H; $^3J_{HH}$ = 8.4 Hz; -C**H**-CH-C(COOMe)-); 7.89–7.93 (m; 1H; -C**H**-CH-CH-N-); 8.12–8.16 (d; 2H; $^3J_{HH}$ = 8.4 Hz; -C**H**-C(COOMe)-); 8.63 (d; 1H; $^3J_{HH}$ = 3.8 Hz; -C**H**-N-CH-C-); 8.88 (s; 1H; -N-C**H**-C-) ppm. ^{13}C NMR (75.5 MHz; $CDCl_3$): δ = 52.4 (1C; -O**C**H$_3$); 123.8 (1C; -CH-**C**H-CH-N-); 127.2 (2C; -**C**H-CH-C(COOMe)-); 129.9 (1C; -**C**(COOMe)-); 130.5 (2C; -**C**H-C(COOMe)-); 134.7 (1C; -**C**H-CH-CH-N-); 135.7 (1C; -N-CH-**C**-); 142.3 (1C; -**C**-CH-CH-C(COOMe)-); 148.4 (1C; -N-**C**H-C-); 149.3 (1C; -**C**H-N-CH-C-); 166.9 (1C; -**C**OOMe) ppm. HRMS (ESI$^+$) calcd. for $C_{13}H_{12}NO_2^+$: 214.0863; found: 214.0861.

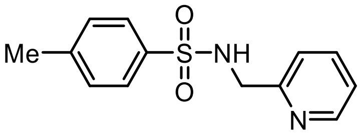

Compound ***1ag.*** 4-Methyl-*N*-(pyridin-2-yl-methyl)benzensulfonamide. A solution of 4-methylbenzenesulfonyl chloride (190,6 mg; 1 mmol; 1 eq.), pyridin-2-ylmethanamine (108,1 mg; 103 µL; 1 mmol; 1 eq.) and *N*,*N*-diisopropylethylamine (142,1 mg; 191,6 µL; 1,1 mmol; 1,1 eq.) in 1,4-dioxan (2 mL) was prepared and stirred for 12 h at room temperature. After dilution with ethyl acetate (20 mL) the solution was washed with NH_4Cl solution (30 mL, saturated) and brine (30 mL). The organic layer was dried with Na_2SO_4, filtered and the solvent was evaporated under reduced pressure. The crude product was used without further purification (247 mg; 0,94 mmol; 94%).

^{1}H NMR (300 MHz; CD_2Cl_2): *δ* = 2,38 (s; 3H; C**H**$_3$); 4,22 (d; 2H; $^3J_{HH}$ = 5,4 Hz; C**H**$_2$); 6,03 (s; 1H; N**H**); 7,14–7,18 (m; 2H; N-C-C**H**; N-CH-C**H**); 7,26 (d; 2H; $^3J_{HH}$ = 8,2 Hz; CH_3-C-(C**H**)$_2$); 7,61 (dt; $^3J_{HH}$ = 7,7 Hz; $^4J_{HH}$ = 1,7 Hz; N-CH-CH-C**H**); 7,70 (d; 2H; $^3J_{HH}$ = 8,2 Hz; SO_2-C-(C**H**)$_2$); 8,43 (m; 2H; N-C**H**) ppm. ^{13}C NMR (75,5 MHz; CD_2Cl_2): *δ* = 21,6 (1C; **C**H$_3$); 47,7 (1C; **C**H$_2$); 122,3 (1C; N-CH-**C**H); 123,0 (1C; N-C-**C**H); 127,5 (2C; SO_2-C-(**C**H)$_2$); 130,0 (2C; CH_3-C-(**C**H)$_2$); 137,1 (1C; N-CH-CH-**C**H); 137,2 (1C; SO_2-**C**); 143,9 (1C; CH_3-**C**); 149,2 (1C; N-**C**H); 155,3 (1C; N-**C**) ppm. HRMS (ESI$^+$) calcd. for $C_{13}H_{15}N_2O_2S^+$: 263,0849; found: 263,0847.

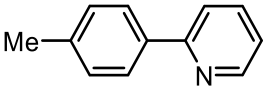

Compound ***2ag.*** 2-(*p*-Tolyl)pyridine. A solution of 4-methyl-*N*-(pyridin-2-yl-methyl)benzensulfonamide (**1ag**) (23,3 mg; 88,8 µmol) in MeCN (50 mL) was loaded on the photo reactor with a flow rate of 2 mL/min and irradiated with UV light (254 nm) at room temperature. The solvent fraction containing the photoproduct was collected and the solvent was removed under reduced pressure. The residue was purified by open column chromatography (silica, R_f=0.20, DCM with 1% *N*,*N*-diisopropylethylamine) to yield the title compound (2,4 mg; 14,2 µmol; 16%) as a white solid.

^{1}H NMR (500 MHz; $CDCl_3$): *δ* = 2.41 (s; 3H; C-C**H**$_3$); 7.22–7.28 (m; 1H; -N=CH-C**H**-); 7.28–7.32 (m; 2H; -C**H**-C-CH_3); 7.72–7.75 (m; 1H; -C**H**-C=N-); 7.76–7.82 (m; 1H; -C**H**-CH-C=N-); 7.89–7.93 (m; 2H; -C**H**-C-C=N-); 8.68–8.73 (m; 1H; -C**H**=N-C-) ppm. ^{13}C NMR (125.8 MHz; $CDCl_3$): *δ* = 21.3 (1C; C-**C**H$_3$); 120.6 (1C; -**C**H-C=N-); 121.9 (1C; -N=CH-**C**H-); 126.9 (2C; -**C**H-CH-C-CH_3); 129.6 (2C; -**C**H-C-CH_3); 135.7 (1C; -**C**-C=N-); 137.5 (1C; -**C**H-CH-C=N-); 139.4 (1C; -**C**-CH_3); 148.9 (1C; -**C**H=N-); 157.1 (1C; -C-**C**=N-) ppm. HRMS (ESI$^+$) calcd. for $C_{12}H_{12}N^+$: 170.0964; found: 170.0964.

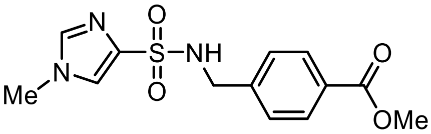

Compound ***1ah.*** Methyl 4-(((1-methyl-1*H*-imidazole)-4-sulfonamido)methyl)benzoate. A solution of 1-methyl-1*H*-imidazole-4-sulfonyl chloride (100 mg, 0.55 mmol, 1 eq.), methyl 4 (aminomethyl)benzoate hydrochloride (123 mg, 0.61 mmol, 1,1 eq.) and *N*,*N*-diisopropylethylamine (157.4 mg, 212.2 µL, 1.22 mmol, 2.2 eq.) in DCM (3 mL) was stirred for 2 h at room temperature. The solution was washed with water (3 mL), aqueous hydrochloric acid (0.5 M, 3 mL) and water (3 mL). The organic layer was dried with sodium sulfate, filtered and the solvent was evaporated. The crude product was obtained as white a solid (70,5 mg, 0.23 mmol, 41%) and used without further purification.

^{1}H NMR (300 MHz, $CDCl_3$) *δ* = 3.66 – 3.75 (s, 3H, N-C**H$_3$**), 3.87 – 3.94 (s, 3H, O-C**H$_3$**), 4.16 – 4.35 (d, $^3J_{HH}$ = 4.2, 2H, N-C**H$_2$**), 5.89 – 6.21 (s, 1H, N**H**), 7.34 – 7.41 (m, 3H, CH_2-C-C**H** & NMe-C**H**-C), 7.45 – 7.51 (s, 1H, N-C**H**-N), 7.91 – 7.98 (d, $^3J_{HH}$ = 8.3, 2H, C**H**-C-COOMe) ppm. ^{13}C NMR (75 MHz, $CDCl_3$) *δ* = 34.2 (1C, N-**C**H$_3$), 47.1 (1C, N-**C**H$_2$), 52.3 (1C, O-**C**H$_3$), 124.2 (1C, N-**C**H-C), 128.0 (2C, CH_2-C-**C**H), 129.6 (1C, COOMe-**C**), 129.9 (2C, **C**H-C-COOMe), 139.2 (1C, N-**C**H-N), 140.2(1C, SO_2-**C**), 142.1 (1C, CH_2-**C**), 166.9 (1C, **C**OOMe) ppm. HRMS (ESI$^+$) calcd. for $C_{13}H_{16}N_3O_4S^+$: 310,0856; found: 310,0845.

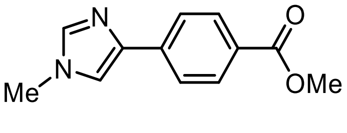

Compound ***2ah.*** Methyl 4-(1-methyl-1*H*-imidazol-4-yl)benzoate. A solution of methyl 4-(((1-methyl-1*H*-imidazole)-4-sulfonamido)methyl)benzoate (**1ah**) (14,5 mg; 46,9 µmol) in MeCN (15 mL) was loaded on the photo reactor with a flow rate of 0,7 mL/min and irradiated with UV light (254 nm) at room temperature. The solvent fraction containing the photoproduct was collected and the solvent was removed under reduced pressure. The residue was purified by open column chromatography (silica, R_f=0.20, ethyl acetate) to yield the title compound (5,9 mg; 27,3 µmol; 58%) as a white solid.

^{1}H NMR (300 MHz, $CDCl_3$) *δ* = 3.73 – 3.78 (s, 3H, N-C**H$_3$**), 3.89 – 3.93 (s, 3H, O-CH_3), 7.27 – 7.29 (d, $^4J_{HH}$ = 1.3, 1H, NMe-C**H**-C), 7.58 – 7.63 (m, 1H, N-C**H**-N), 7.79 – 7.86 (m, 2H, C**H**-C-COOMe), 8.01 – 8.08 (m, 2H, C**H**-CH-C-COOMe) ppm. ^{13}C NMR (75 MHz, $CDCl_3$) *δ* = 33.9 (N-**C**H$_3$), 52.1 (O-**C**H$_3$), 117.5 (1C, N-**C**H-C), 124.6 (2C, **C**H-C-COOMe), 128.4 (1C, **C**-COOMe), 130.2 (2C,

CH-CH-C-COOMe), 138.2 (1C, C-**C**-CH-CH), 138.5 (1C, N-**C**H-N), 141.1 (1C, N-**C**H-C), 167.2 (1C, **C**OOMe) ppm. HRMS (ESI$^+$) calcd. for $C_{12}H_{13}N_2O_2^+$: 217,0972; found: 217,0963.

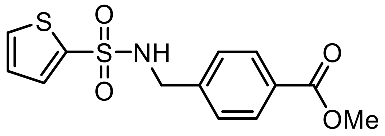

*Compound **1ai.*** Methyl 4-((thiophene-2-sulfonamido)methyl)benzoate. A solution of thiophene-2-sulfonyl chloride (100 mg, 0,55 mmol, 1 eq.), methyl 4-(aminomethyl)benzoate hydrochloride (116 mg, 0,58 mmol, 1 eq.) and *N*,*N*-diisopropylethylamine (155 mg, 210 µL, 1.21 mmol, 2.2 eq.) in DCM (2 mL) was stirred for 2 h at room temperature. After dilution with DCM (8 mL) the solution was washed with water (4 mL), aqueous hydrochloric acid (1 M, 4 mL) and water (4 mL). The organic layer was dried with sodium sulfate, filtered and the solvent was evaporated under reduced pressure. The residue was purified by open column chromatography (silica, R_f=0.13, DCM) to yield the title compound (162 mg; 0,52 mmol; 95%) as a crystalline solid.
^{1}H NMR (300 MHz; $CDCl_3$): δ = 3.84-3.98 (s, 3H, -OC**H**$_3$), 4.22- 4.36 (d, 2H, $^3J_{HH}$ = 6.3 Hz, -C**H**$_2$-), 4.87- 5.02 (t, 1H, $^3J_{HH}$ = 6.3 Hz, -N**H**-), 7.04-7.12 (dd, 1H, $^3J_{HH}$ = 5.0, 3.8, S-CH-C**H**-CH), 7.27-7.37 (d, 2H, $^3J_{HH}$ = 8.2 Hz, -C**H**-CH-C-COOMe), 7.56-7.65 (m, 2H, S-C**H**-CH-C**H**), 7.89- 8.01 (d, 2H, $^3J_{HH}$ = 8.2 Hz, C**H**-C-COOMe) ppm. ^{13}C NMR (75.5 MHz; $CDCl_3$): δ = 47.3 (1C, -**C**H$_2$-), 52.3 (1C, -O**C**H$_3$), 127.6 (1C, S-CH-**C**H-CH), 127.8 (2C, CH$_2$-C-**C**H), 130.0 (1C, -**C**-COOMe), 130.2 (2C, CH$_2$-C-CH-**C**H), 132.4 & 132.6 (2C, S-**C**H-CH-**C**H, 140.9 (1C, CH$_2$-**C)**, 141.3 (1C, -**C**-SO$_2$-), 166.8 (1C, -**C**OOMe) ppm. HRMS (ESI$^+$) calcd. for $C_{13}H_{14}NO_4S_2^+$: 312.0359; found: 312.0357. HRMS (ESI$^+$) calcd. for $C_{13}H_{12}NO_4S_2^-$: 310.0213; found: 310.0212.

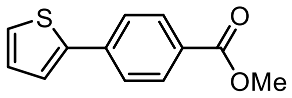

*Compound **2ai.*** Methyl 4-(thiophen-2-yl)benzoate. A solution of methyl 4-((thiophene-2-sulfonamido)methyl)benzoate (**1ai**) (20,5 mg; 65,8 µmol) in MeCN (10 mL) was loaded on the photo reactor with a flow rate of 4 mL/min and irradiated with UV light (254 nm) at room temperature. The solvent fraction containing the photoproduct was collected and the solvent was removed under reduced pressure. The residue was purified by open column chromatography (silica, R_f=0.80, DCM) to yield the title compound (4,5 mg; 20,6 µmol; 31%) as a white solid.
^{1}H NMR (300 MHz; $CDCl_3$): δ = 3.93 (s, 3H, -OC**H**$_3$), 7.10-7.13 (dd, 1H, $^3J_{HH}$ = 5.1, 3.6, S-CH-C**H**-CH), 7.33-7.45 (m, 2H, S-C**H**-CH-C**H**), 7.64-7.73 (d, 2H, $^3J_{HH}$ = 8.5 Hz, C**H**-CH-C-COOMe), 7.97-8.08 (d, 2H, $^3J_{HH}$ = 8.5 Hz, C**H**-C-COOMe) ppm. ^{13}C NMR (75.5 MHz; $CDCl_3$): δ = 52.3 (1C, -O**C**H$_3$), 124.6 & 126.4 (2C, S-**C**H-CH-**C**H), 125.7 (2C, **C**H-CH-C-COOMe), 128.5 (1C, S-CH-**C**H), 128.9 (1C, **C**-COOMe), 130.4 (2C, **C**H-C-COOMe), 138.8 (1C, S-C-**C**), 143.2 (1C, S-**C**), 166.9 (1C, -**C**OOMe) ppm. HRMS (ESI$^+$) calcd. for $C_{12}H_{11}O_2S^+$: 219,0474; found: 219.0477.

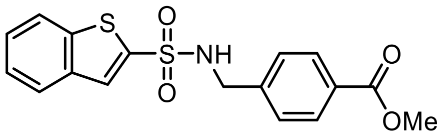

*Compound **1aj.*** Methyl 4-((benzo[*b*]thiophene-2-sulfonamido)methyl)benzoate. A solution of benzo[*b*]thiophene-2-sulfonyl chloride (50 mg, 0.21 mmol, 1 eq.), methyl 4-(aminomethyl)benzoate hydrochloride (47.7 mg, 0.24 mmol, 1.1 eq.) and *N*,*N*-diisopropylethylamine (61.1 mg, 82.3 µL, 0.47 µmol, 2,2 eq.) in DCM (3 mL) was stirred for 3 h at room temperature. The mixture was washed with water (3 mL) and aqueous hydrochloric acid (0.5 M, 3 mL). The organic layer was dried with sodium sulfate, filtered and the solvent was evaporated under reduced pressure. The residue was purified by open column chromatography (silica, R_f=0.26, chloroform/ethyl acetate 19:1) to yield the title compound (72.1 mg, 0.21 mmol, 93%) as a white solid.
^{1}H-NMR (300 MHz, $CDCl_3$) δ = 3.89 (s, 3H, -O-C**H$_3$**), 4.32 (d, 2H, $^3J_{HH}$ = 6,2 Hz. -NH-C**H$_2$**-), 5.11 (t, 1H, $^3J_{HH}$ = 6.3 Hz, -N**H**-), 7.36 – 7.29 (m, 2H, -C**H**-CH-C-C(=O)-O-), 7.54 – 7.42 (m, 2H, -CH-C**H**-C**H**-CH-C-CH-), 7.89 – 7.83 (m, 3H, -C**H**-CH-CH-C**H**-C-C**H**-), 7.95 – 7.89 (m, 2H, -C**H**-C-C(=O)-O-) ppm. ^{13}C-NMR (75 MHz, $CDCl_3$):δ = 47.3 (1C, -NH-**C**H$_2$-), 52.3 (1C, -O-**C**H$_3$), 122.8 (1C, -S-C-**C**H-CH-), 125.7 (1C, -S-C-C-**C**H-CH-), 125.8 (1C, -S-C-CH-**C**H-), 127.5 (1C, -S-C-C-CH-**C**H-), 127.8 (2C, -C(=O)-C-(CH-**C**H)$_2$-), 129.8 (1C, -C(=O)-**C**-),129.8 (1C, -S-C-**C**H-C-C-), 130.0 (2C, -C(=O)-C-(**C**H)$_2$-), 137.6 (-S-**C**-SO$_2$-), 140.6 (1C, -S-C-**C**-), 141.2 (1C, -S-**C**-C-), 141.8 (1C, -NH-CH$_2$-**C**-), 166.8 (1C, -**C**(=O)-O-) ppm. HRMS (ESI$^+$) calcd. for $C_{17}H_{16}NO_4S_2^+$: 362.0515, found: 362.0520.

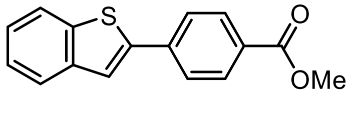

Compound ***2aj.*** Methyl 4-(benzo[*b*]thiophen-2-yl)benzoate. A solution of methyl 4-((benzo[*b*]thiophene-2-sulfonamido)methyl)benzoate (**1aj**) (21,1 mg, 58,4 µmol) in MeCN (3 mL) was loaded on the photo reactor with a flow rate of 1 mL/min and irradiated with UV light (254 nm) at room temperature. The solvent fraction containing the photoproduct was collected and the solvent was removed under reduced pressure. The residue was purified by open column chromatography (silica, R_f=0.75, DCM) to yield the title compound (2,8 mg, 10,4 µmol, 18%) as a faintly red solid.
^{1}H-NMR (300 MHz, $CDCl_3$) δ = 8.12 – 8.06 (m, 2H, -C**H**-C-C(=O)-O-)), 7.88 – 7.75 (m, 4H, -C**H**-CH-CH-CH-C-CH-C-C-C**H**-), 7.66 (d, 1H, $^3J_{HH}$ = 0.6 Hz, -S-C-C**H**-C-), 7.42 – 7.31 (m, 2H, -CH-C**H**-C**H**-CH-), 3.95 (s, 3H, -O-C**H₃**) ppm. ^{13}C-NMR (75 MHz, $CDCl_3$) δ = 166.8 (1C, -**C**(=O)-O-), 142.9 (1C, -CH-CH-CH-C-S-**C**-), 140.6 (1C, -CH-CH-CH-**C**-C-S-), 140.0 (1C, -CH-CH-CH-**C**-S-), 138.7 (1C, -S-C-**C**-CH-CH-C-), 130.4 (2C, -C(=O)-C-**C**H-), 129.7 (1C, -C(=O)-**C**-), 126.3 (2C, -C(=O)-C-CH-**C**H), 125.1 (1C, -S-C-CH-**C**H-), 124.9 (1C, -S-C-CH-CH-**C**H), 124.1 (1C, -S-C-CH-CH-CH-**C**H-), 122.5 (1C, -S-C-CH-**C**H-CH-), 121.2 (1C, -S-C-**C**H-C-), 52.4 (1C, -O-**C**H$_3$) ppm. HRMS (ESI$^+$) calcd. for $C_{16}H_{13}O_2S^+$: 269.0631, found: 269.0634.

Computational details

The ground state potential energy surface of **1a, 1p** and **1aa** were sampled by means of relaxed scans and conformer analysis. All ground state density functional theory (DFT) calculations were carried out using the range-separated exchange correlation functional CAM-B3LYP[4] and the all-electron def2-TZVP triple-ζ basis set[5]. Dispersion interactions were taken into account by Grimme's D3-model with Becke-Johnson damping[6-7].

Relaxed scans were performed with Gaussian 16, Revision B.01[8] at the DFT level (CAM-B3LYP/def2-TZVP) by varying the central (C-N-S-C)-dihedral angle in the sulfonamide-linker from -180° to 180° in 37 steps while equilibrating the remaining degrees of freedom at each step.

Possible conformers of the biaryl substrates were generated by the simulated annealing procedure as implemented in Grimme's extended tight binding code GFN-xTB 5.8 using the GFN2-parametrization. Effects of solvation (acetonitrile) on the conformer geometries were taken into account by the generalized Born solvent area (GBSA) continuum solvation model[9]. Improved energies[10] for the conformers (generated by xTB) were calculated with the domain-based local pair natural orbital coupled cluster approach with triples corrections (DLPNO–CCSD(T)) as implemented in ORCA 4.0.1.2[11-12]. The def2-QZVPP and the corresponding auxiliary basis sets were utilized[13-15]. Solvent effects (acetonitrile, $\varepsilon = 36.6$, $n = 1.344$) on the coupled cluster single point energies were taken into account by the conductor-like polarizable continuum model (CPCM)[16-17]. Tight criteria were used for the self-consistent-field convergence and the truncation threshold in the DLPNO procedure (TightPNO, $T_{\mathrm{CutPairs}} = 10^{-5}$, $T_{\mathrm{CutPNO}} = 10^{-7}$, $T_{\mathrm{cutMKN}} = 10^{-4}$)[18]. To visualize the correlation between the conformer bonding parameters and their coupled cluster energies, a principal component analysis was conducted using a set of eight internal coordinates as features.

Transition state (TS) optimizations, subsequent vibrational analysis and reaction path calculations were carried out at the DFT level, while the nature of the first-order saddle points was confirmed by vibrational analysis. Minimum energy paths were obtained by calculating the intrinsic reaction coordinate (IRC) using the Hessian predictor-corrector method as implemented in Gaussian[19-20] to verify that the optimized TS connects the presumed educt and product of the biaryl coupling reaction. The exact Hessian was recalculated every seventh IRC step. Equally spaced geometries were sampled from both sides of the IRC every sixth step, yielding reaction paths for the subsequent excited state calculations.

All excited-state calculations were carried out in Gaussian 16 using time-dependent DFT (TDDFT) and along the sampled IRC-images. The same basis set and dispersion correction model as for the preliminary ground state DFT calculations was applied. This computational setup allows a balanced description of local as well as of charge transfer excitations among the π-systems of educt and product states.[21] Vertical excitation energies and oscillator strengths for the six lowest singlet excited states were calculated for the sampled geometries along the IRC. Solvent effects (acetonitrile) on the vertical excitation energies and oscillator strengths were taken into account by CPCM. Excited state characters were interpreted in terms of natural transition orbitals (NTOs)[22] as calculated by Multiwfn 3.5[23].

Ground state calculations

Conformational analysis

Definition of internal coordinates for the PCA

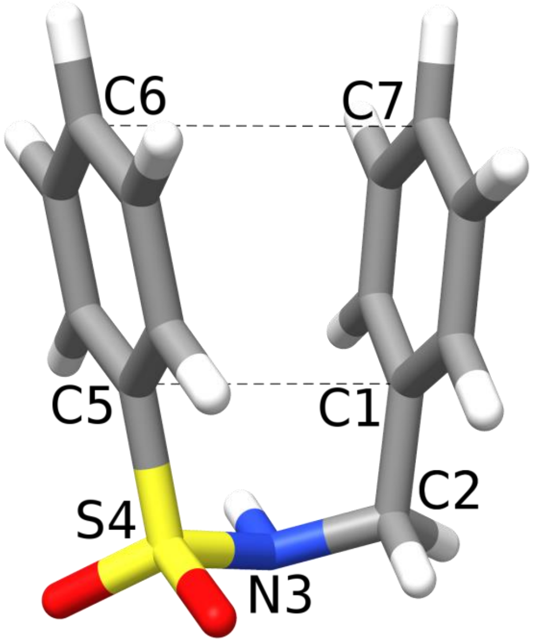

Figure S6: Atom labels used in defining the internal coordinates for the principal component analysis (PCA) of **1a**, **1p** and **1aa**.

Table S2: Definition of the eight internal coordinates used as features in the PCA.

Internal coordinate type	Indices
Distance	C6-C7
Distance	C5-C1
Angle	C5-S4-N3
Angle	S4-N3-C2
Angle	N3-C2-C1
Angle	C5-N3-C2
Dihedral	C5-S4-N3-C2
Dihedral	S4-N3-C2-C1

*Conformers of **1p***

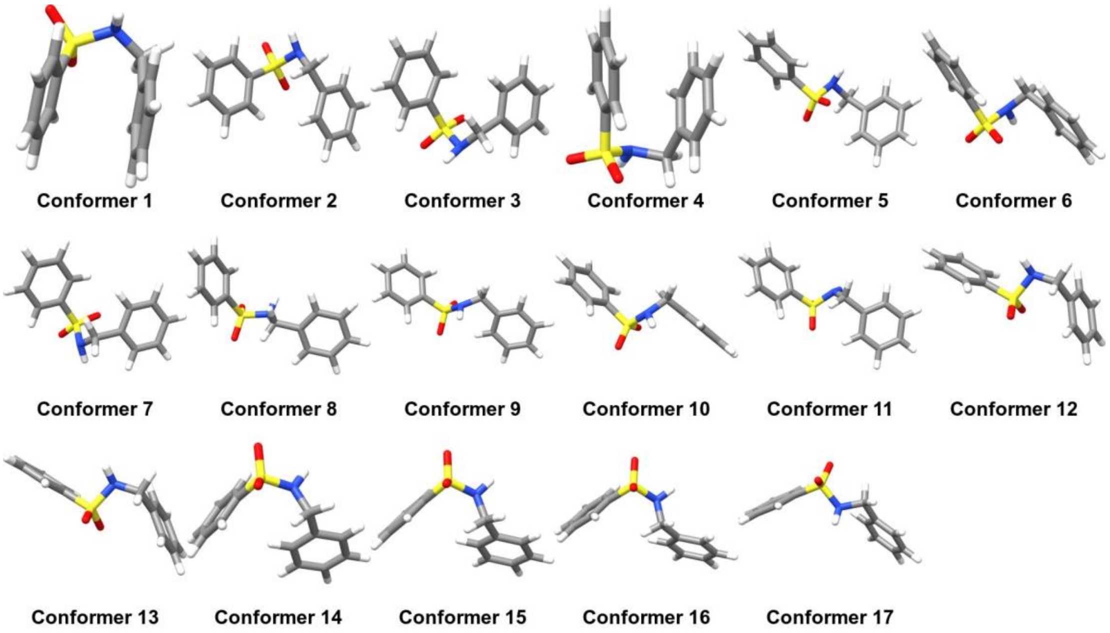

Figure S7: Conformers of **1p** obtained at the GFN2-xTB/GBSA (acetonitrile) level of theory.

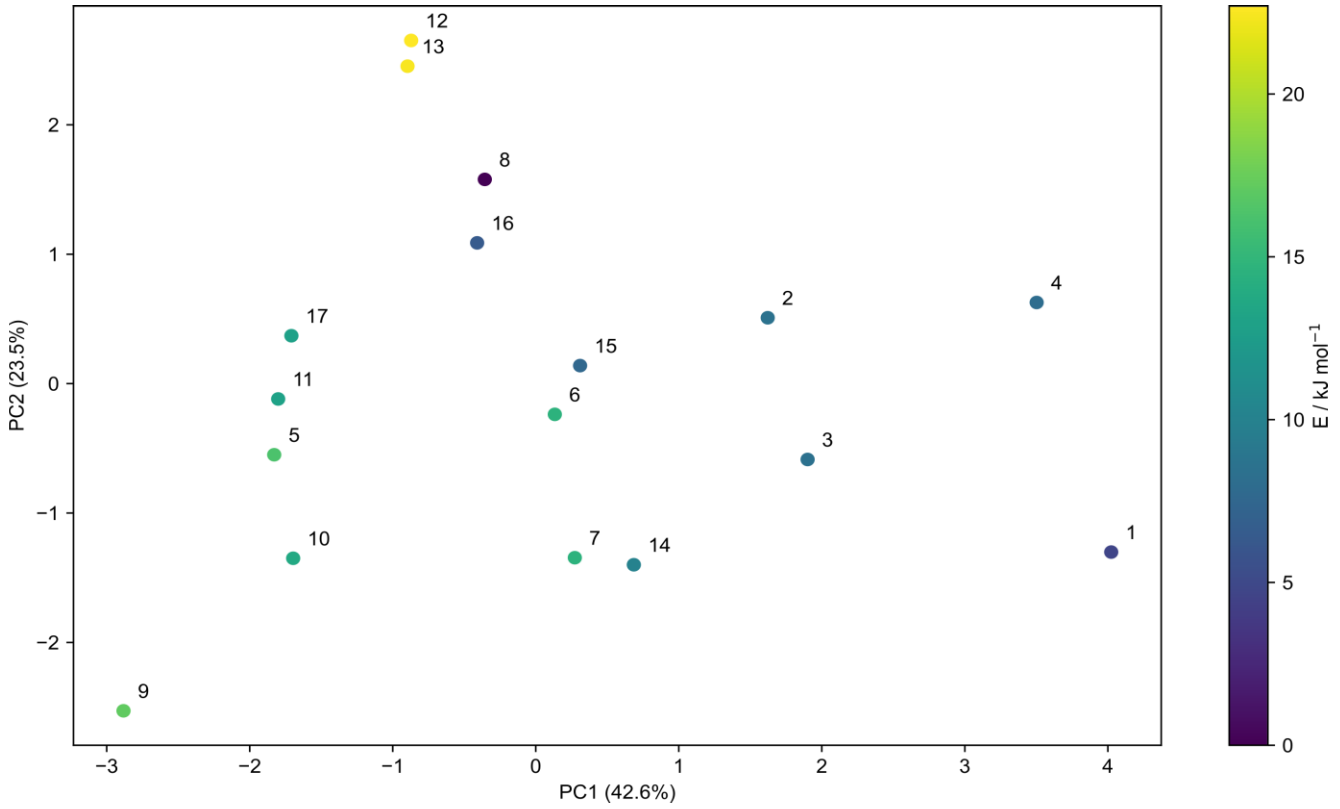

Figure S8: Scatter plot of the DLPNO-CCSD(T)/def2-QZVPP/CPCM (acetonitrile) energies for the first two principal components of the PCA for **1p**. All energies are given in relation to the minimum energy (conformer 8).

*Conformers of **1a***

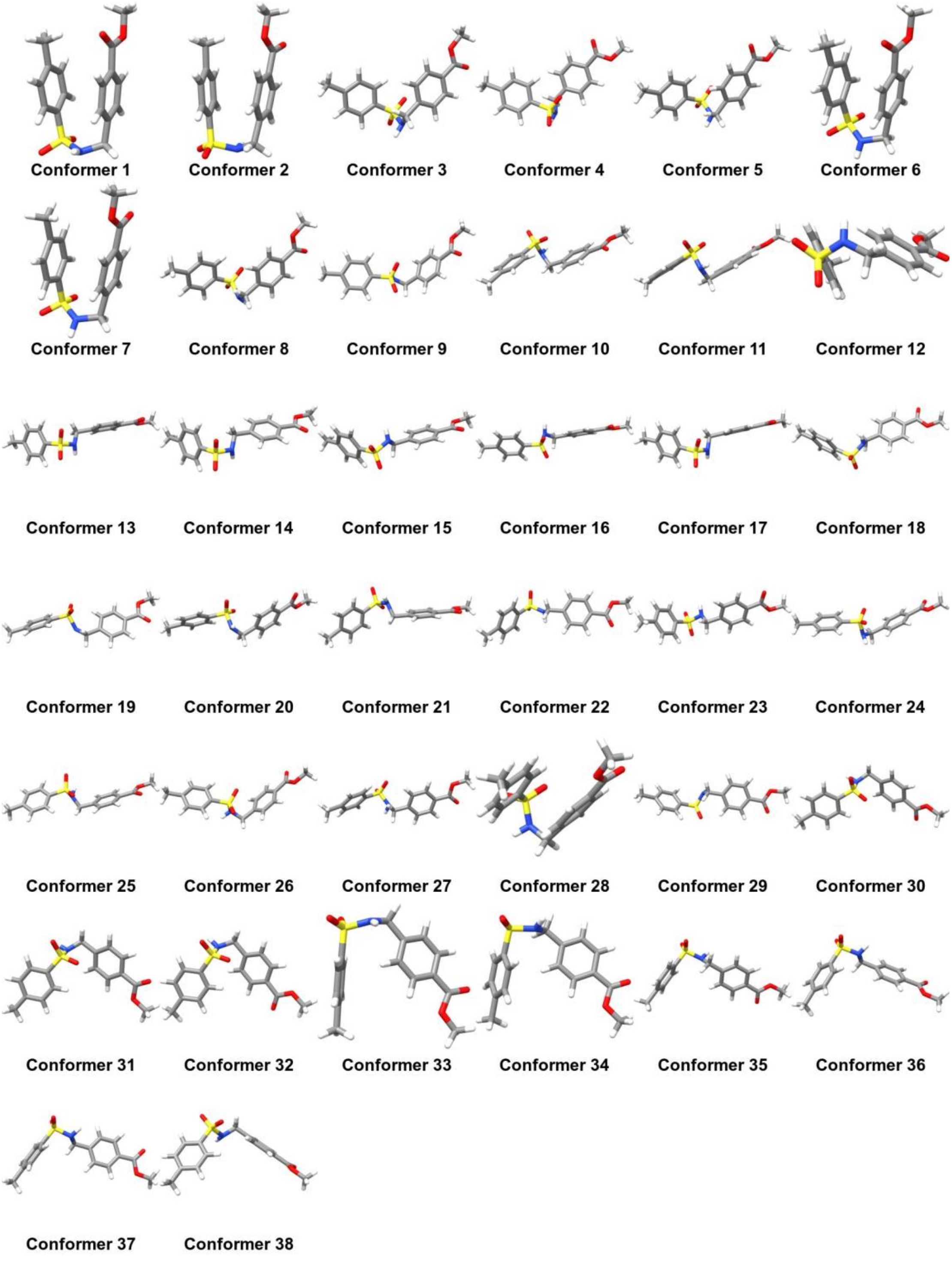

Figure S9: Conformers of **1a** obtained at the GFN2-xTB/GBSA (acetonitrile) level of theory.

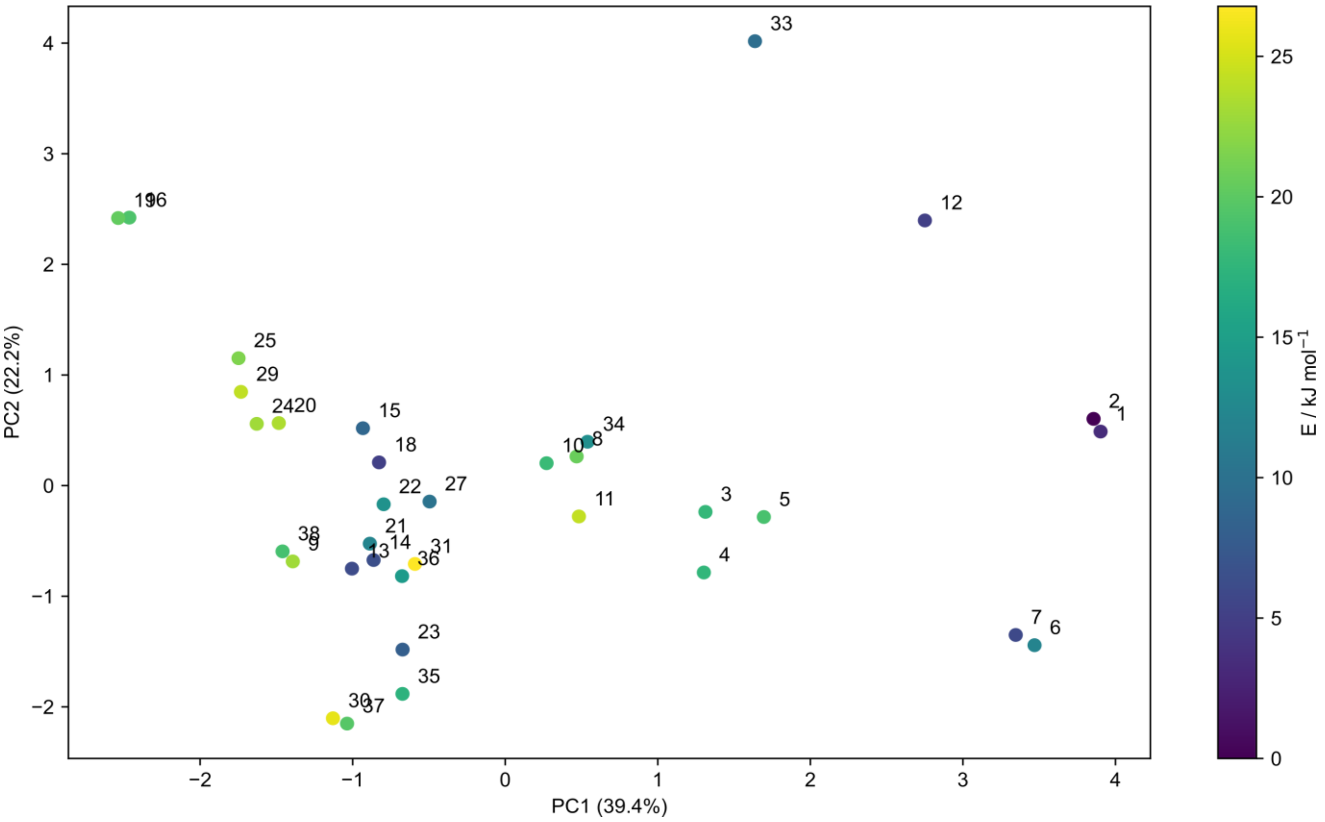

Figure S10: Scatter plot of the DLPNO-CCSD(T)/def2-QZVPP/CPCM (acetonitrile) energies for the first two principal components of the PCA for **1a**. All energies are given in relation to the minimum energy (conformer 2). Conformers with energies above 30 kJ/mol (conformers 17, 26, 28 and 32) were excluded.

Conformers of **1aa**

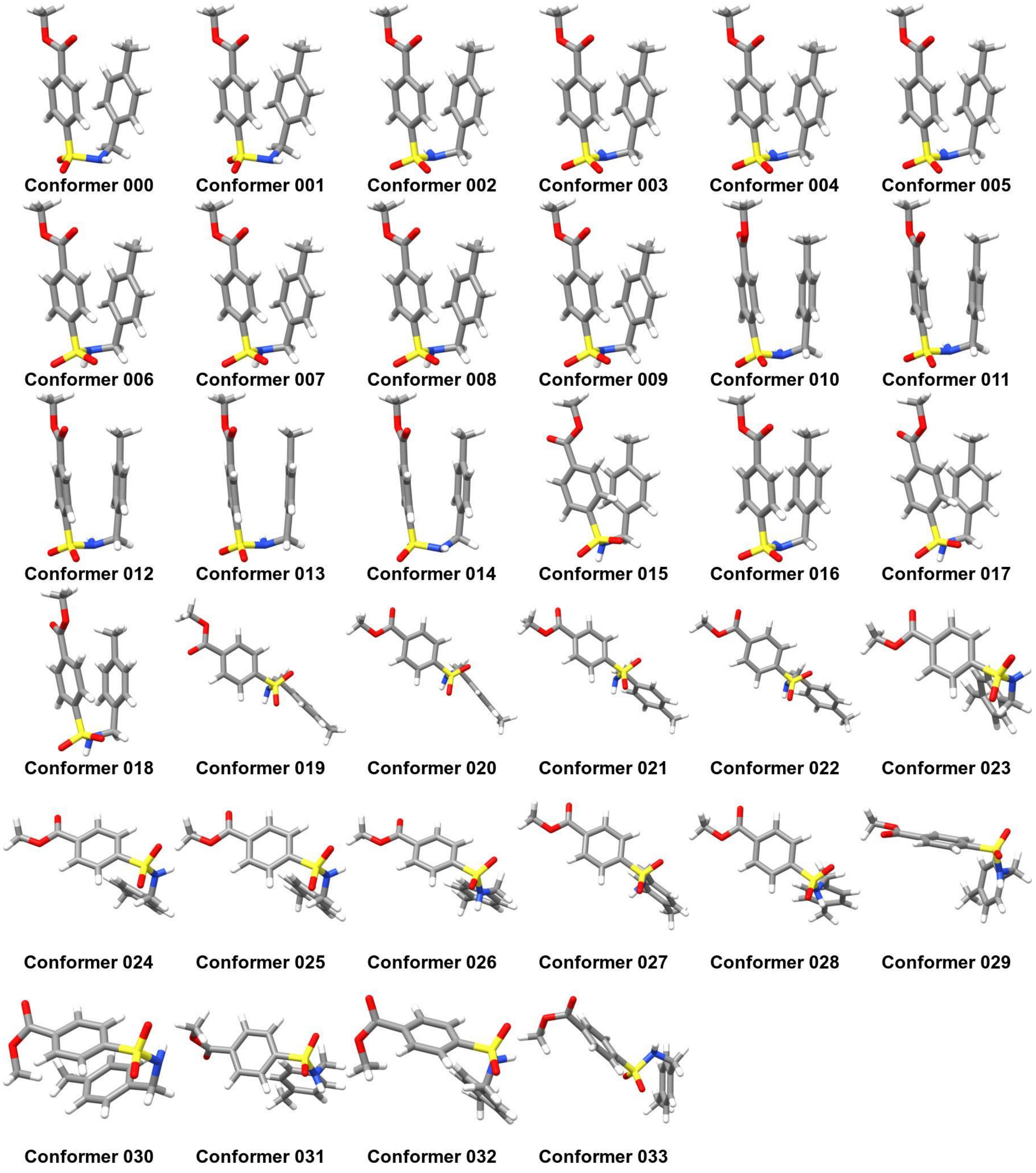

Figure S11: Conformers of **1aa** obtained at the GFN2-xTB/GBSA (acetonitrile) level of theory.

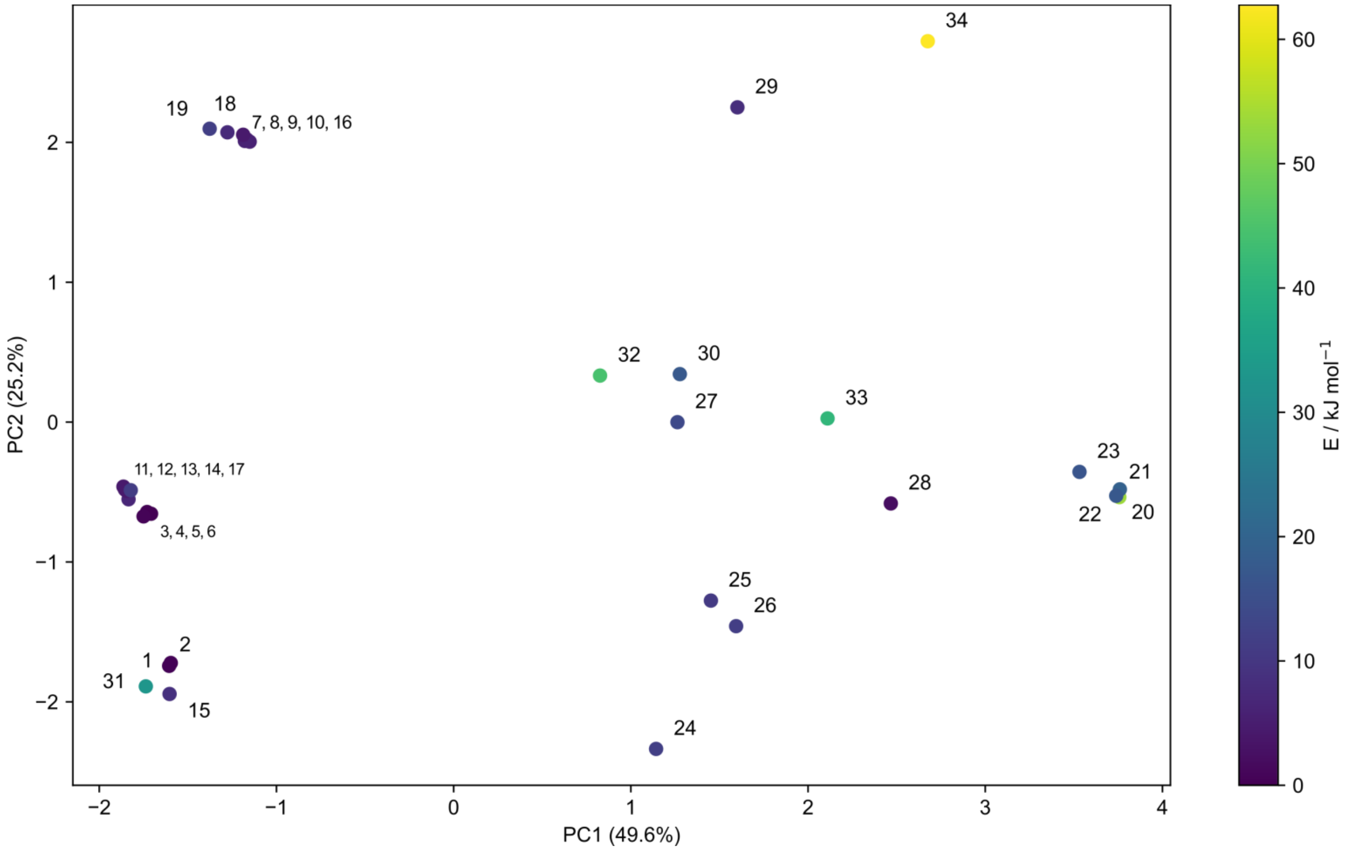

Figure S12: Scatter plot of the DLPNO-CCSD(T)/def2-QZVPP/CPCM (acetonitrile) energies for the first two principal components of the PCA for **1aa**. All energies are given in relation to the minimum energy (conformer 6). Conformers with energies above 30 kJ/mol (conformers 28-36) were excluded.

Frontier molecular orbitals

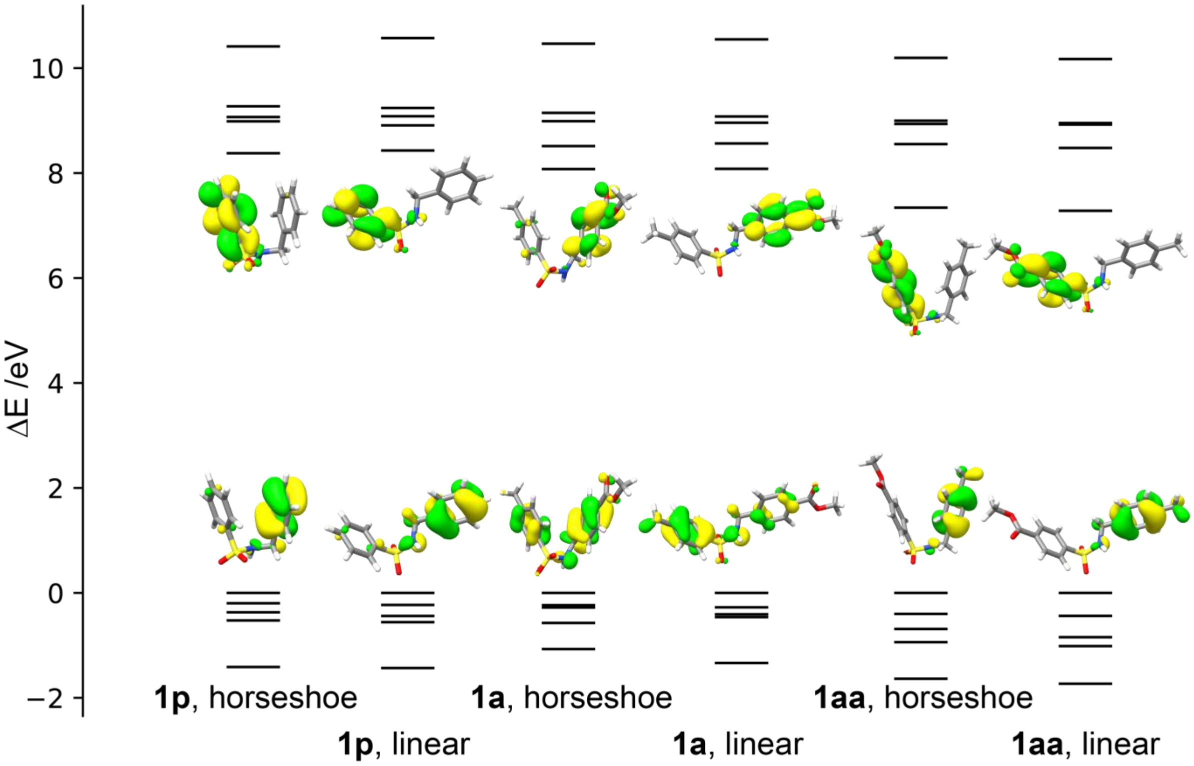

Figure S13: Isocontour plots and energies of the highest occupied molecular orbital (HOMO) and the lowest unoccupied molecular orbital (LUMO) for linear and horseshoe conformations of **1a, 1p** and **1aa** as obtained at the CAM-B3LYP/def2-TZVP/CPCM (acetonitrile) level of theory.

*Intrinsic Reaction Coordinate of **1p***

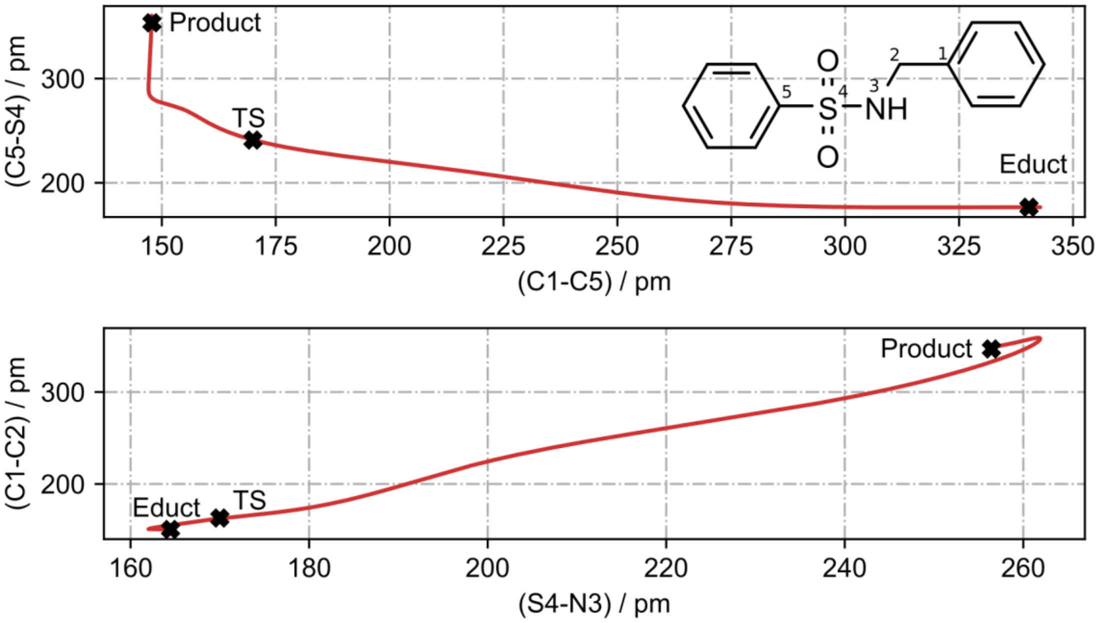

Figure S14: Selected bond lengths over the course of the IRC for **1p** as obtained at the CAM-B3LYP/def2-TZVP level of theory.

*Intrinsic Reaction Coordinate of **1a***

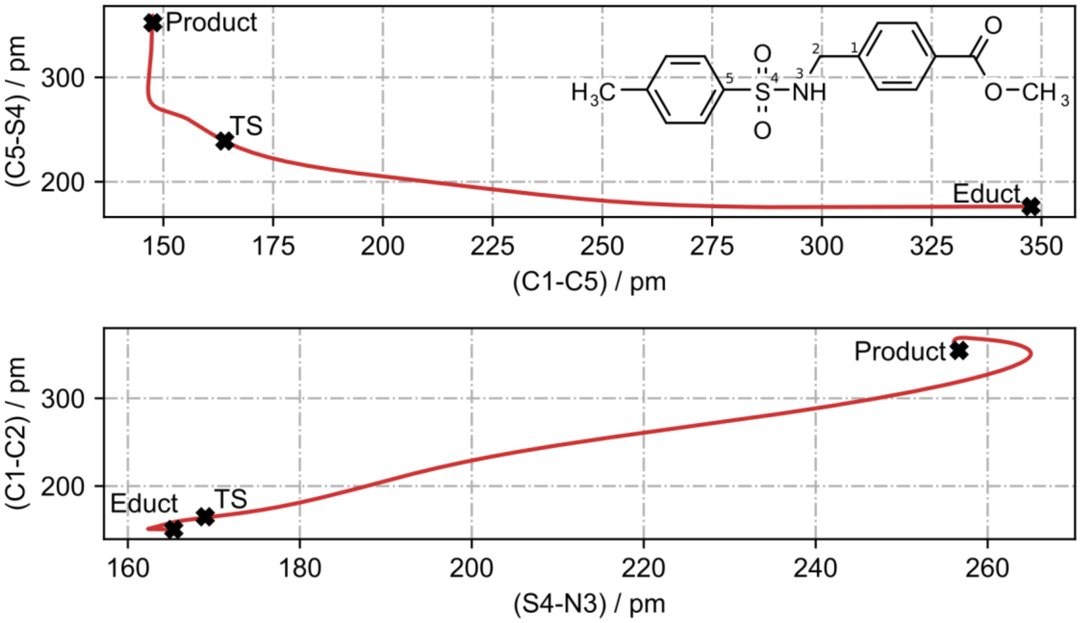

Figure S15: Selected bond lengths over the course of the IRC for **1a** as obtained at the CAM-B3LYP/def2-TZVP level of theory.

Intrinsic Reaction Coordinate of ***1aa***

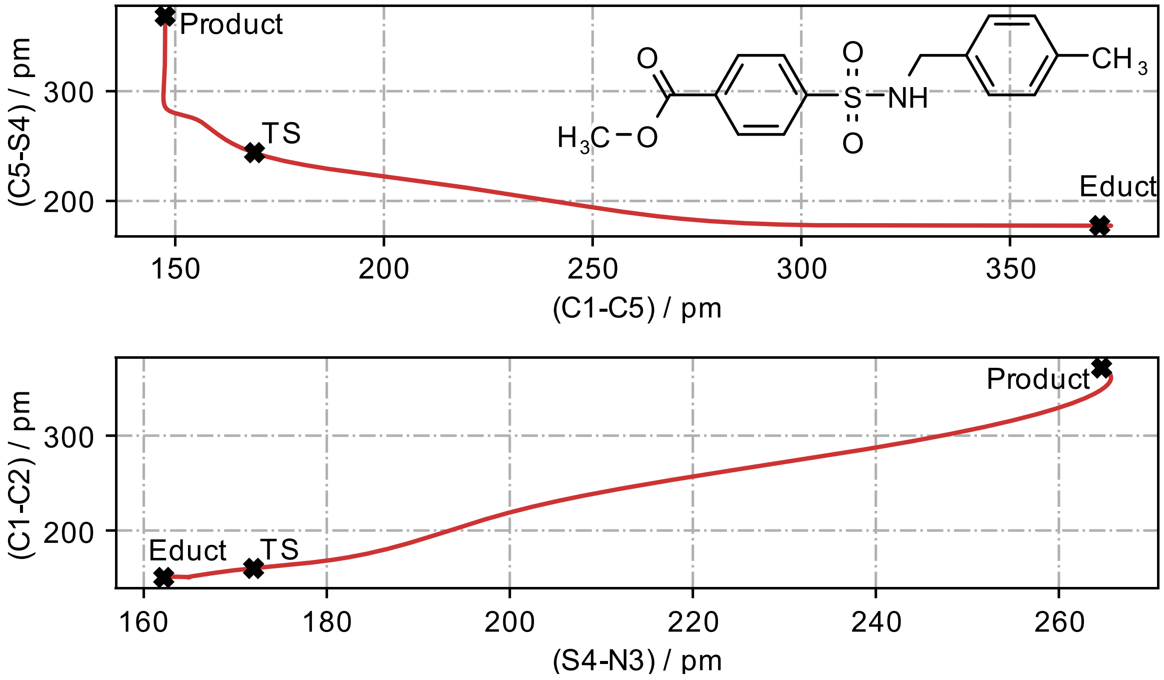

Figure S16: Selected bond lengths over the course of the IRC for **1aa** as obtained at the CAM-B3LYP/def2-TZVP level of theory.

Excited state calculations

Comparison between experimental and calculated spectrum

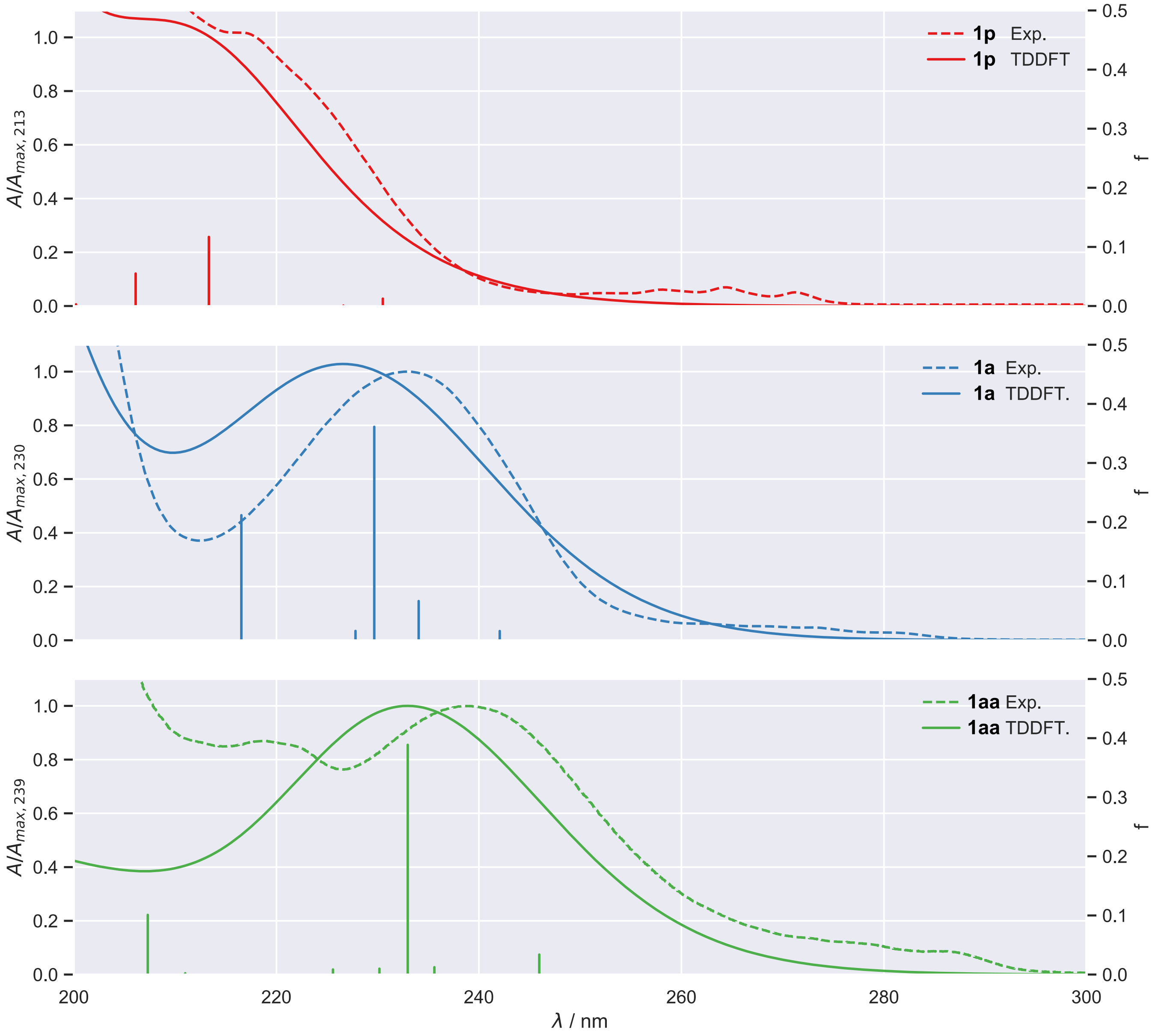

Figure S17: Comparison between experimental (dashed line) and calculated spectrum of **1p, 1a** and **1aa** as obtained at the CAM-B3LYP/def2-TZVP/CPCM (acetonitrile) level of theory.

Nomenclature

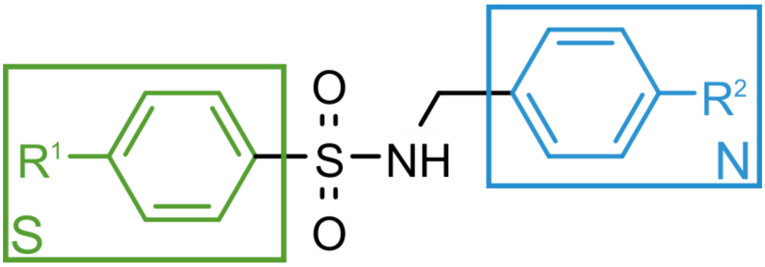

Figure S18: π-orbitals located on the S-linked phenyl residue (green) are denoted by a S-subscript (π_S), whereas π-orbitals located on the CH_2-NH-linked phenyl residue (blue) are given a N-subscript (π_N).

*TDDFT spectrum of **1p**, horseshoe*

Table S3: Calculated low-lying excited singlet states, excitation wavelengths (λ/nm), excitation energies (ΔE/eV), oscillator strengths and leading NTO pairs of **1p** for a horseshoe conformation at the educt side of the IRC obtained at the CAM-B3LYP/def2-TZVP/CPCM (acetonitrile) level of theory.

State	*λ*/nm	Δ*E*/eV	*f*	Transition	Weight/%
S_1	230.5	5.38	0.0125	$\pi_S \rightarrow \pi^*_S$<br>$\pi_S \rightarrow \pi^*_S$	67<br>33
S_2	226.6	5.47	0.0007	$\pi_N \rightarrow \pi^*_N$<br>$\pi_N \rightarrow \pi^*_N$	52<br>48
S_3	213.3	5.81	0.1169	$\pi_S \rightarrow \pi^*_S$	86
S_4	206.1	6.02	0.0550	$\pi_N \rightarrow \pi^*_N, \pi^*_S, \sigma_{CC}$<br>$\pi_N \rightarrow \pi^*_N$	74<br>19
S_5	200.2	6.19	0.0028	$\pi_N \rightarrow \pi^*_S$	87
S_6	194.6	6.37	0.0172	$\pi_N \rightarrow \pi^*_S$	94

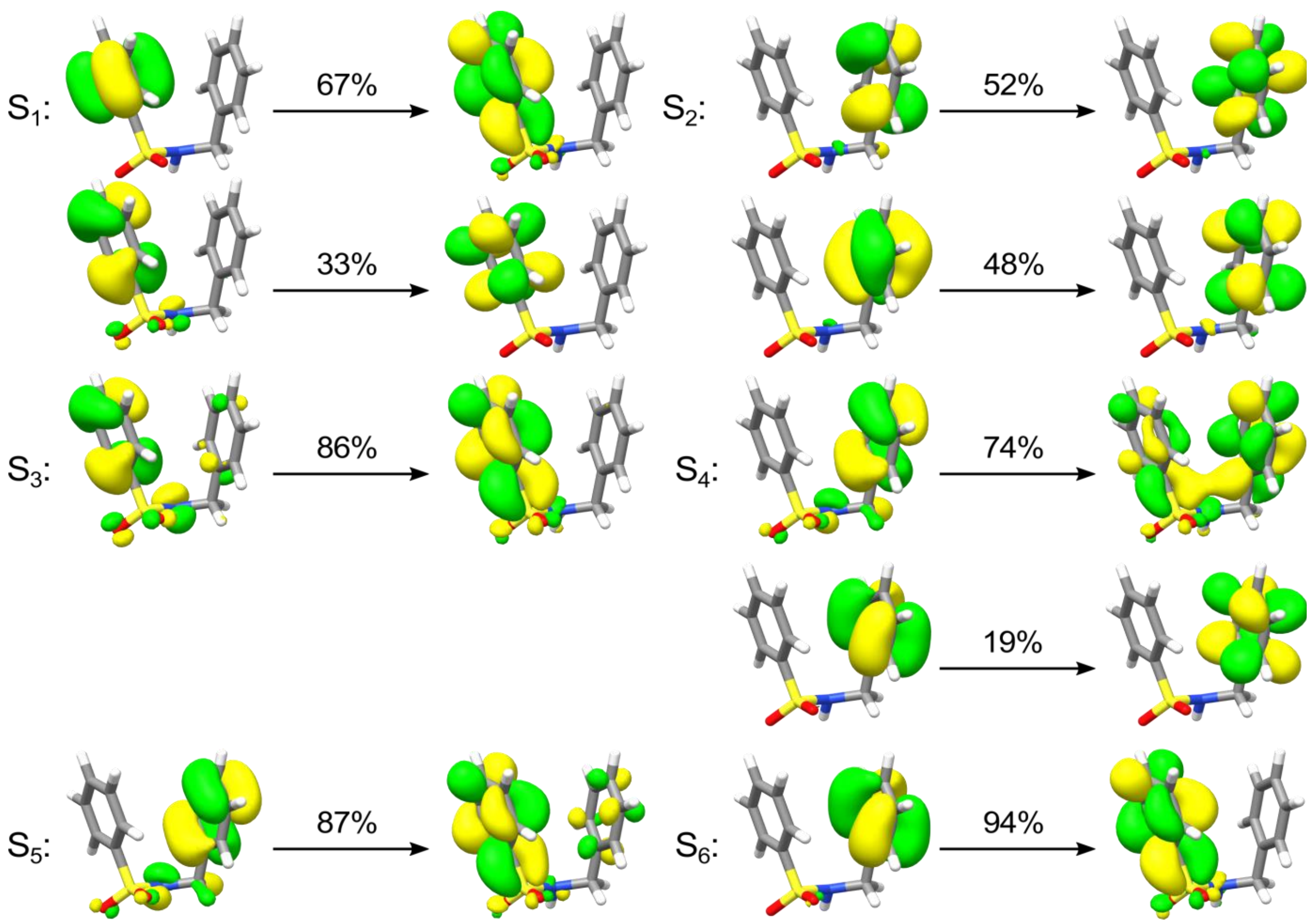

Figure S19: Leading NTO pairs for the horseshoe conformation of **1p**.

TDDFT spectrum of ***1p****, linear*

Table S4: Calculated low-lying excited singlet states, excitation wavelengths (λ/nm), excitation energies (ΔE/eV), oscillator strengths and leading NTO pairs for a linear form of **1p** (linear minimum of the relaxed scan) obtained at the CAM-B3LYP/def2-TZVP/CPCM (acetonitrile) level of theory.

State	*λ*/nm	Δ*E*/eV	*f*	Transition	Weight/%
S_1	229.3	5.41	0.0122	$\pi_S \rightarrow \pi^*_S$<br>$\pi_S \rightarrow \pi^*_S$	63<br>37
S_2	226.0	5.49	0.0001	$\pi_N \rightarrow \pi^*_N$<br>$\pi_N \rightarrow \pi^*_N$	54<br>45
S_3	210.5	5.89	0.2619	$\pi_S \rightarrow \pi^*_S$	83
S_4	204.1	6.08	0.0350	$\pi_N \rightarrow \pi^*_N$<br>$\pi_N \rightarrow \pi^*_N$	68<br>30
S_5	189.0	6.56	0.5052	$\pi_N \rightarrow \pi^*_S$<br>$\pi_S \rightarrow \pi^*_S$	57<br>35
S_6	183.6	6.75	0.4564	$\pi_S \rightarrow \pi^*_S$<br>$\pi_S \rightarrow \pi^*_S$	63<br>36

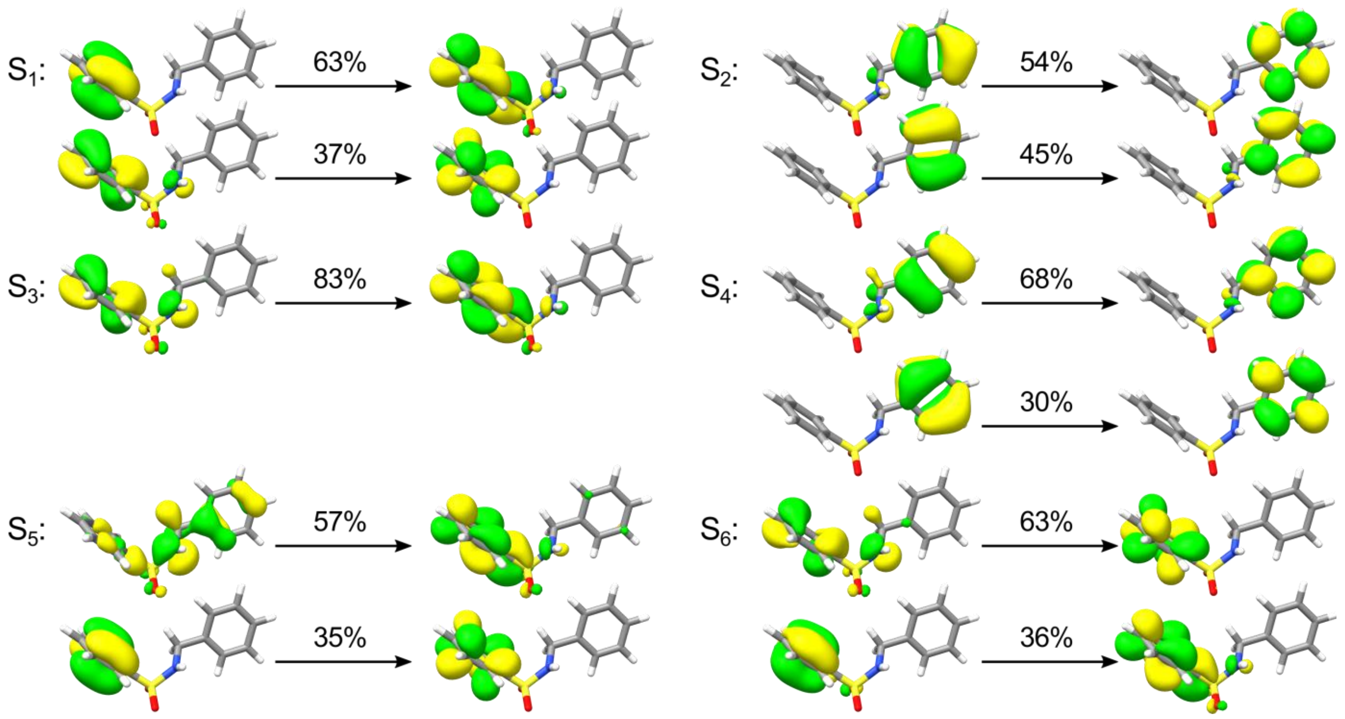

Figure S20: Leading NTO pairs for the linear form of **1p**.

*TDDFT spectrum of **1a**, horseshoe*

Table S5: Calculated low-lying excited singlet states, excitation wavelengths (λ/nm), excitation energies (ΔE/eV), oscillator strengths and leading NTO pairs of **1a** for a horseshoe conformation at the educt side of the IRC obtained at the CAM-B3LYP/def2-TZVP/CPCM (acetonitrile) level of theory.

State	***λ*/nm**	**Δ*E*/eV**	***f***	Transition	Weight/%
S_1	242.1	5.12	0.0157	$\pi_N \rightarrow \pi^*_N$<br>$\pi_N \rightarrow \pi^*_N$	74<br>25
S_2	234.1	5.30	0.0664	$\pi_S \rightarrow \pi^*_S, \sigma_{CC}$<br>$\pi_S \rightarrow \pi^*_S$	57<br>28
S_3	229.7	5.40	0.3611	$\pi_N \rightarrow \pi^*_N$<br>$\pi_S \rightarrow \pi^*_S$	70<br>16
S_4	227.8	5.44	0.0158	$n_O \rightarrow \pi^*_N$	98
S_5	216.5	5.73	0.2116	$\pi_S \rightarrow \pi^*_S$	82
S_6	195.9	6.33	0.0444	$\pi_S \rightarrow \pi^*_N, \sigma_{CC}$<br>$\pi_S, \pi_N \rightarrow \pi^*_S$	58<br>24

.

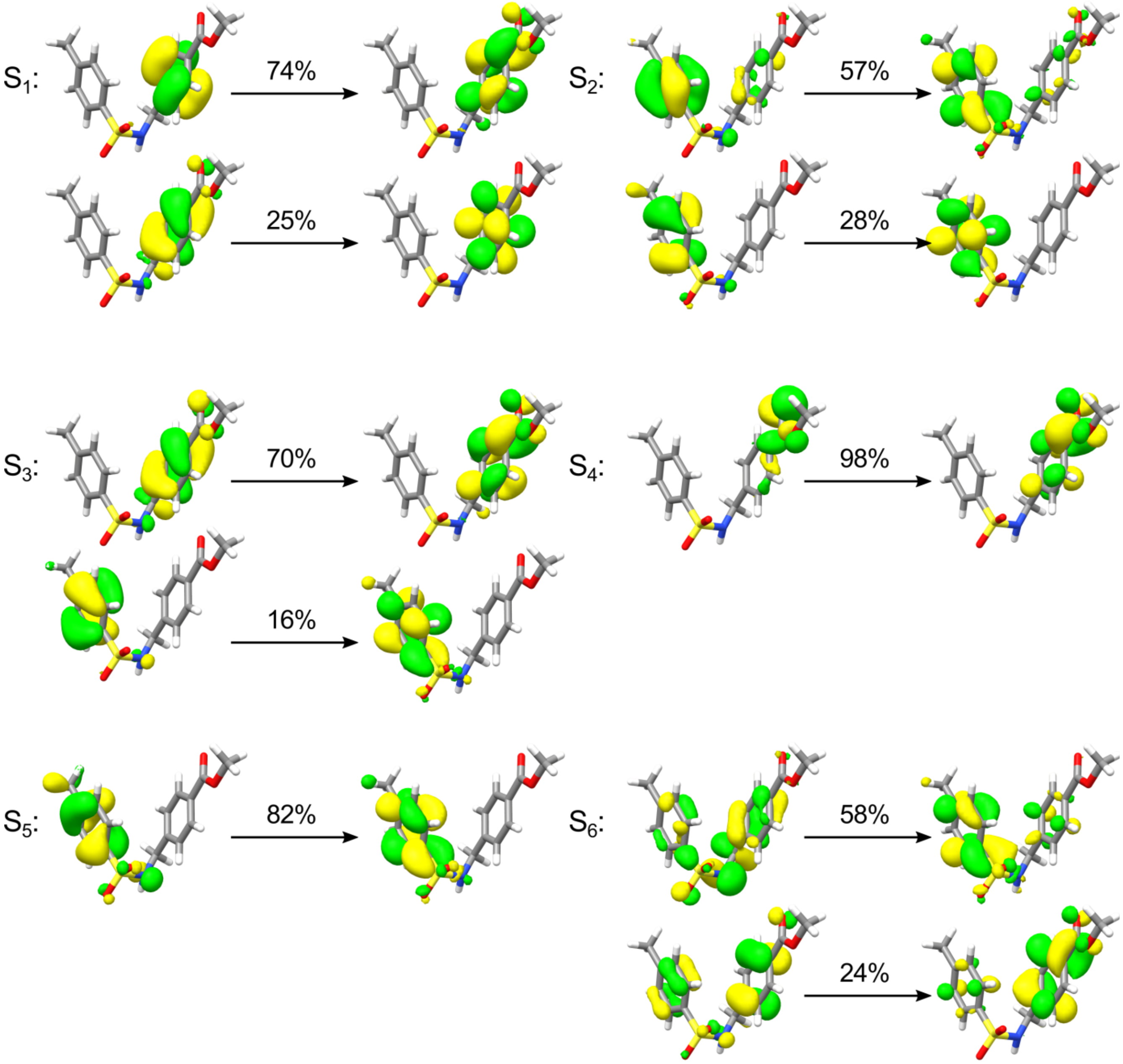

Figure S21: Leading NTO pairs for the horseshoe conformation of **1a**.

*TDDFT spectrum of **1a**, linear*

Table S6: Calculated low-lying excited singlet states, excitation wavelengths (λ/nm), excitation energies (ΔE/eV), oscillator strengths and leading NTO pairs for a linear form of **1a** (linear minimum of the relaxed scan) obtained at the CAM-B3LYP/def2-TZVP/CPCM (acetonitrile) level of theory.

State	*λ*/nm	Δ*E*/eV	*f*	Transition	Weight/%
S_1	240.4	5.16	0.0212	$\pi_N \rightarrow \pi^*_N$<br>$\pi_N \rightarrow \pi^*_N$	74<br>26
S_2	231.9	5.35	0.0063	$\pi_S \rightarrow \pi^*_S$<br>$\pi_S \rightarrow \pi^*_S$	53<br>47
S_3	228.2	5.43	0.0767	$n_O \rightarrow \pi^*_N$	97
S_4	227.7	5.44	0.5335	$\pi_N \rightarrow \pi^*_N$	90
S_5	215.6	5.75	0.2402	$\pi_S \rightarrow \pi^*_S$	83
S_6	193.5	6.41	0.0721	$p_N \rightarrow \pi^*_N$<br>$\pi_N \rightarrow \pi^*_N$	79<br>20

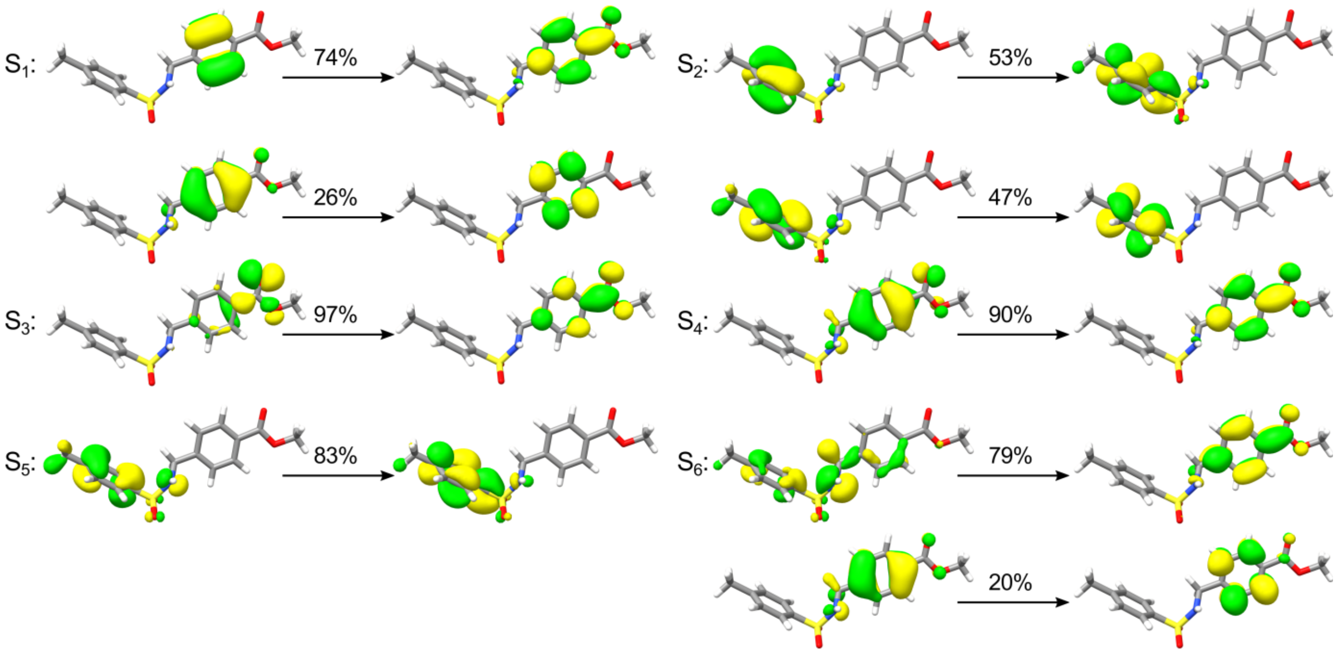

Figure S22: Leading NTO pairs for the linear form of **1a**.

*Comparison of excited states along the IRC for **1a** and **1p***

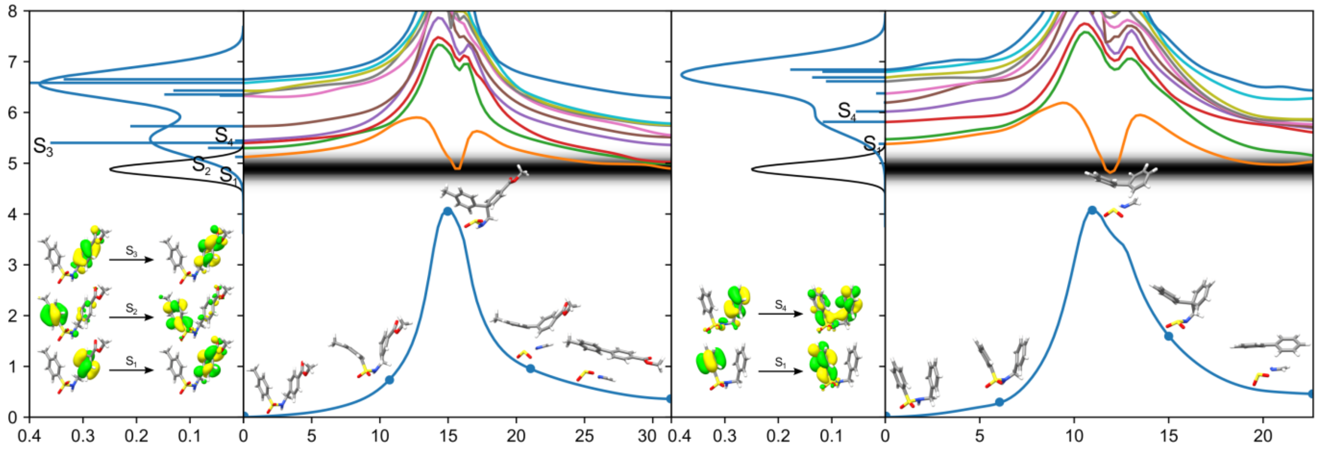

Figure S23: Comparison between excited states of **1a** and **1p** along their respective IRCs as obtained at the TDDFT level of theory (CAM-B3LYP/def2-TZVP/CPCM (acetonitrile)).

*TDDFT spectrum of **1aa**, horseshoe*

Table S7: Calculated low-lying excited singlet states, excitation wavelengths (λ/nm), excitation energies (ΔE/eV), oscillator strengths and leading NTO pairs of **1aa** for a horseshoe conformation at the educt side of the IRC obtained at the CAM-B3LYP/def2-TZVP/CPCM (acetonitrile) level of theory.

State	***λ*/nm**	**Δ*E*/eV**	***f***	Transition	Weight/%
S_1	246.0	5.04	0.0338	$\pi_S \rightarrow \pi^*_S$	81
S_2	235.6	5.26	0.0124	$n_O \rightarrow \pi^*_S$	99
S_3	233.0	5.32	0.3886	$\pi_S \rightarrow \pi^*_S$	93
S_4	230.2	5.39	0.0100	$\pi_N \rightarrow \pi^*_N$<br>$\pi_N \rightarrow \pi^*_N$	64<br>36
S_5	225.6	5.50	0.0086	$\pi_N \rightarrow \pi^*_S$	98
S_6	211.0	5.88	0.0022	$\pi_N \rightarrow \pi^*_S$	98
S_7	207.3	5.98	0.1010	$\pi_N \rightarrow \pi^*_N$<br>$\pi_N \rightarrow \pi^*_N$	68<br>30
S_8	195.9	6.33	0.0293	p_N, $\sigma_{CS} \rightarrow \pi^*_S$	90

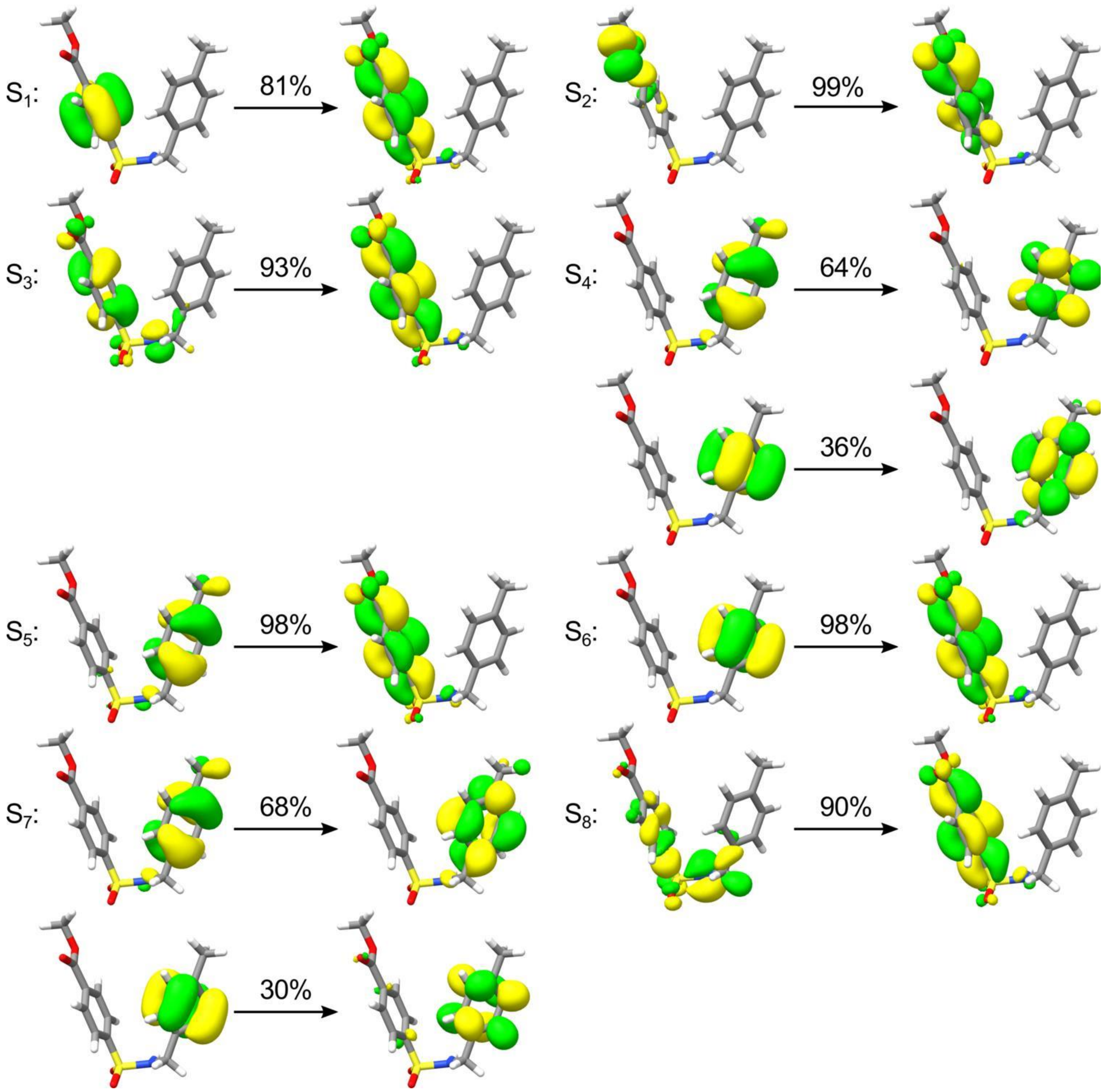

Figure S24: Leading NTO pairs for the horseshoe conformation of **1aa**.

*TDDFT spectrum of **1aa**, linear*

Table S8: Calculated low-lying excited singlet states, excitation wavelengths (λ/nm), excitation energies (ΔE/eV), oscillator strengths and leading NTO pairs of **1aa** for a linear conformation (linear minimum of the relaxed scan) obtained at the CAM-B3LYP/def2-TZVP/CPCM (acetonitrile) level of theory.

State	*λ*/nm	Δ*E*/eV	*f*	Transition	Weight/%
S_1	245.4	5.05	0.0405	$\pi_S \rightarrow \pi^*_S$	81
S_2	235.3	5.27	0.0049	$n_O \rightarrow \pi^*_S$	99
S_3	232.2	5.34	0.4409	$\pi_S \rightarrow \pi^*_S$	76
S_4	230.0	5.39	0.1631	$\pi_N \rightarrow \pi^*_N$<br>$\pi_N \rightarrow \pi^*_N$	50<br>28
S_5	211.0	5.88	0.0347	$\pi_N \rightarrow \pi^*_S$	95
S_6	207.8	5.97	0.0959	$\pi_N \rightarrow \pi^*_N$<br>$\pi_N \rightarrow \pi^*_N$	72<br>26

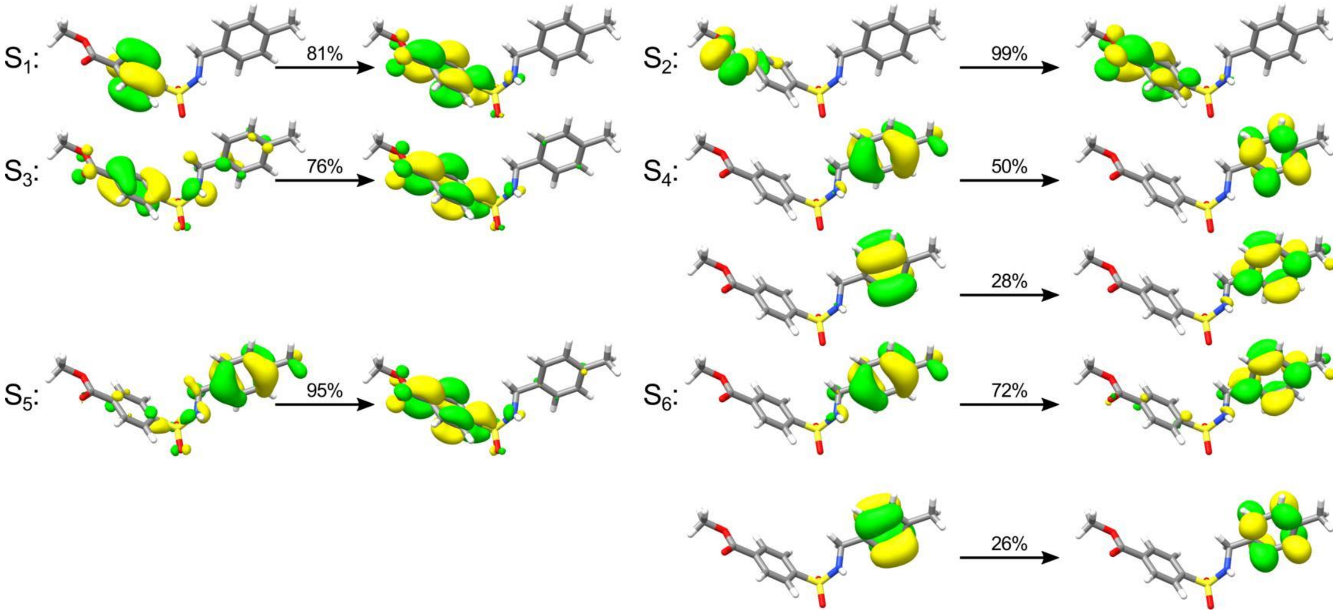

Figure S25: Leading NTO pairs for the linear form of **1aa**.

Video of the mechanism

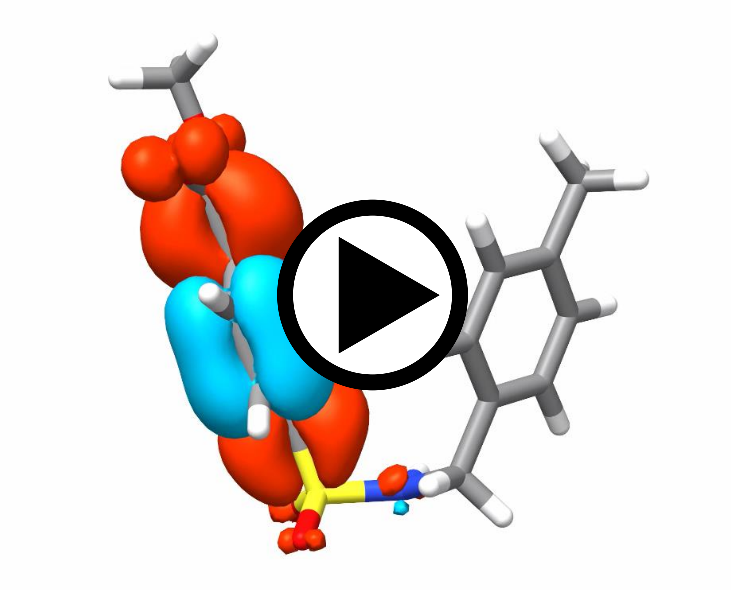

Figure S26: Video of charge density difference in the S_1 along the calculated IRC of **1aa**. Blue isosurfaces denote regions of charge density decrease, whereas red isosurfaces denote regions of charge density increase.

References

1. Fulmer, G. R.; Miller, A. J.; Sherden, N. H.; Gottlieb, H. E.; Nudelman, A.; Stoltz, B. M.; Bercaw, J. E.; Goldberg, K. I., NMR chemical shifts of trace impurities: common laboratory solvents, organics, and gases in deuterated solvents relevant to the organometallic chemist. *Organometallics* **2010,** *29* (9), 2176-2179.
2. Rosenau, C. P.; Jelier, B. J.; Gossert, A. D.; Togni, A., Exposing the origins of irreproducibility in fluorine NMR spectroscopy. *Angew. Chem., Int. Ed.* **2018,** *57* (30), 9528-9533.
3. Kloss, F.; Neuwirth, T.; Haensch, V. G.; Hertweck, C., Metal-Free Synthesis of Pharmaceutically Important Biaryls by Photosplicing. *Angew. Chem., Int. Ed.* **2018,** *57* (44), 14476-14481.
4. Yanai, T.; Tew, D. P.; Handy, N. C., A new hybrid exchange–correlation functional using the Coulomb-attenuating method (CAM-B3LYP). *Chem. Phys. Lett.* **2004,** *393* (1-3), 51-57.
5. Weigend, F.; Ahlrichs, R., Balanced basis sets of split valence, triple zeta valence and quadruple zeta valence quality for H to Rn: Design and assessment of accuracy. *Phys. Chem. Chem. Phys.* **2005,** *7* (18), 3297-3305.
6. Grimme, S.; Antony, J.; Ehrlich, S.; Krieg, H., A consistent and accurate ab initio parametrization of density functional dispersion correction (DFT-D) for the 94 elements H-Pu. *J. Chem. Phys.* **2010,** *132* (15), 154104.
7. Grimme, S.; Ehrlich, S.; Goerigk, L., Effect of the damping function in dispersion corrected density functional theory. *J. Comput. Chem.* **2011,** *32* (7), 1456-1465.
8. Frisch, M. J.; Trucks, G. W.; Schlegel, H. B.; Scuseria, G. E.; Robb, M. A.; Cheeseman, J. R.; Scalmani, G.; Barone, V.; Petersson, G. A.; Nakatsuji, H.; Li, X.; Caricato, M.; Marenich, A. V.; Bloino, J.; Janesko, B. G.; Gomperts, R.; Mennucci, B.; Hratchian, H. P.; Ortiz, J. V.; Izmaylov, A. F.; Sonnenberg, J. L.; Williams; Ding, F.; Lipparini, F.; Egidi, F.; Goings, J.; Peng, B.; Petrone, A.; Henderson, T.; Ranasinghe, D.; Zakrzewski, V. G.; Gao, J.; Rega, N.; Zheng, G.; Liang, W.; Hada, M.; Ehara, M.; Toyota, K.; Fukuda, R.; Hasegawa, J.; Ishida, M.; Nakajima, T.; Honda, Y.; Kitao, O.; Nakai, H.; Vreven, T.; Throssell, K.; Montgomery Jr., J. A.; Peralta, J. E.; Ogliaro, F.; Bearpark, M. J.; Heyd, J. J.; Brothers, E. N.; Kudin, K. N.; Staroverov, V. N.; Keith, T. A.; Kobayashi, R.; Normand, J.; Raghavachari, K.; Rendell, A. P.; Burant, J. C.; Iyengar, S. S.; Tomasi, J.; Cossi, M.; Millam, J. M.; Klene, M.; Adamo, C.; Cammi, R.; Ochterski, J. W.; Martin, R. L.; Morokuma, K.; Farkas, O.; Foresman, J. B.; Fox, D. J. *Gaussian 16 Rev. B.01*, Wallingford, CT, 2016.
9. Still, W. C.; Tempczyk, A.; Hawley, R. C.; Hendrickson, T., Semianalytical treatment of solvation for molecular mechanics and dynamics. *J. Am. Chem. Soc.* **1990,** *112* (16), 6127-6129.
10. Paulechka, E.; Kazakov, A., Efficient DLPNO–CCSD (T)-based estimation of formation enthalpies for C-, H-, O-, and N-containing closed-shell compounds validated against critically evaluated experimental data. *J. Phys. Chem. A* **2017,** *121* (22), 4379-4387.
11. Riplinger, C.; Neese, F., An efficient and near linear scaling pair natural orbital based local coupled cluster method. *J. Chem. Phys.* **2013,** *138* (3), 034106.
12. Riplinger, C.; Sandhoefer, B.; Hansen, A.; Neese, F., Natural triple excitations in local coupled cluster calculations with pair natural orbitals. *J. Chem. Phys.* **2013,** *139* (13), 134101.
13. Weigend, F.; Ahlrichs, R. J. P. C. C. P., Balanced basis sets of split valence, triple zeta valence and quadruple zeta valence quality for H to Rn: Design and assessment of accuracy. **2005,** *7* (18), 3297-3305.
14. Weigend, F., Accurate Coulomb-fitting basis sets for H to Rn. *Phys. Chem. Chem. Phys.* **2005,** *8*, 1057-1065.
15. Hellweg, A.; Hättig, C.; Höfener, S.; Klopper, W., Optimized accurate auxiliary basis sets for RI-MP2 and RI-CC2 calculations for the atoms Rb to Rn. *Theor. Chem. Acc.* **2007,** *117* (4), 587-597.
16. Barone, V.; Cossi, M., Quantum calculation of molecular energies and energy gradients in solution by a conductor solvent model. *J. Phys. Chem. A* **1998,** *102* (11), 1995-2001.
17. Cossi, M.; Rega, N.; Scalmani, G.; Barone, V., Energies, structures, and electronic properties of molecules in solution with the C‐PCM solvation model. *J. Comput. Chem.* **2003,** *24* (6), 669-681.
18. Liakos, D. G.; Sparta, M.; Kesharwani, M. K.; Martin, J. M.; Neese, F., Exploring the accuracy limits of local pair natural orbital coupled-cluster theory. *J. Chem. Theory Comput.* **2015,** *11* (4), 1525-1539.
19. Fukui, K., Formulation of the reaction coordinate. *J. Chem. Phys.* **1970,** *74* (23), 4161-4163.
20. Hratchian, H. P.; Schlegel, H. B., Accurate reaction paths using a Hessian based predictor–corrector integrator. *J. Chem. Phys.* **2004,** *120* (21), 9918-9924.
21. Peach, M. J.; Benfield, P.; Helgaker, T.; Tozer, D. J., Excitation energies in density functional theory: An evaluation and a diagnostic test. *J. Chem. Phys.* **2008,** *128* (4), 044118.
22. Martin, R. L., Natural transition orbitals. *J. Chem. Phys.* **2003,** *118* (11), 4775-4777.
23. Lu, T.; Chen, F., Multiwfn: a multifunctional wavefunction analyzer. *J. Comput. Chem.* **2012,** *33* (5), 580-592.

^{1}H and ^{13}C NMR Spectra

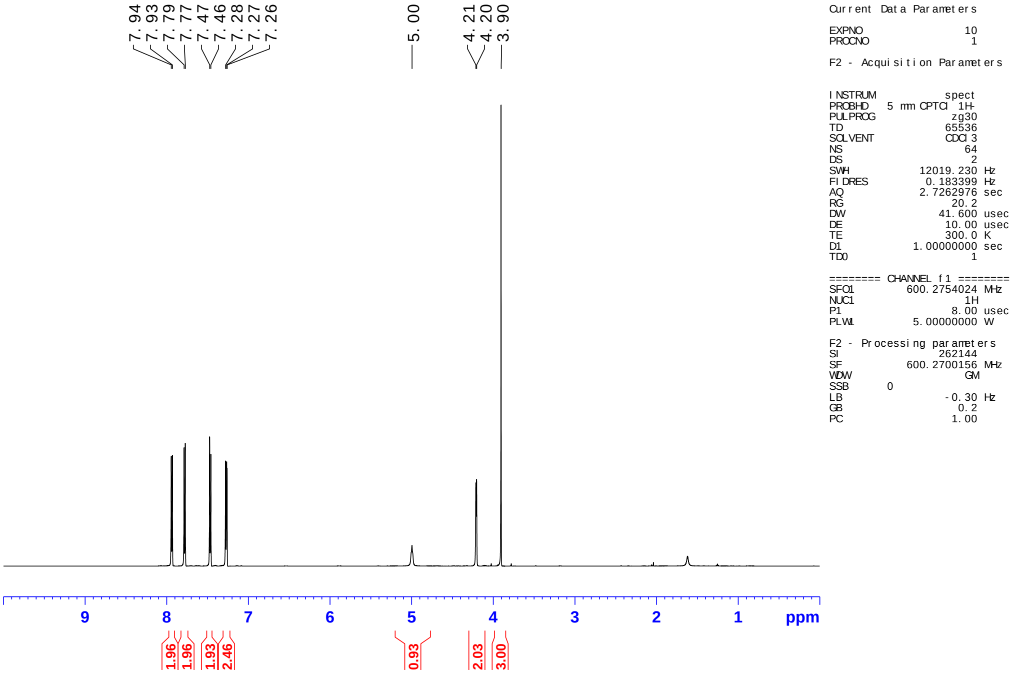

Figure S27: ^{1}H NMR spectrum of **1d**.

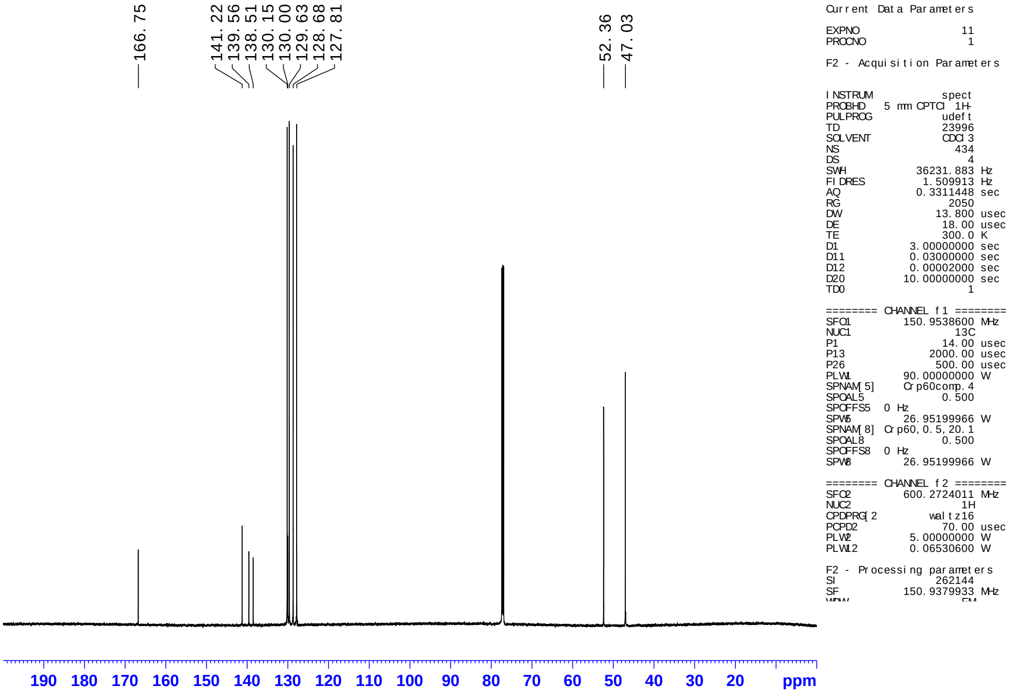

Figure S28: ^{13}C NMR spectrum of **1d**.

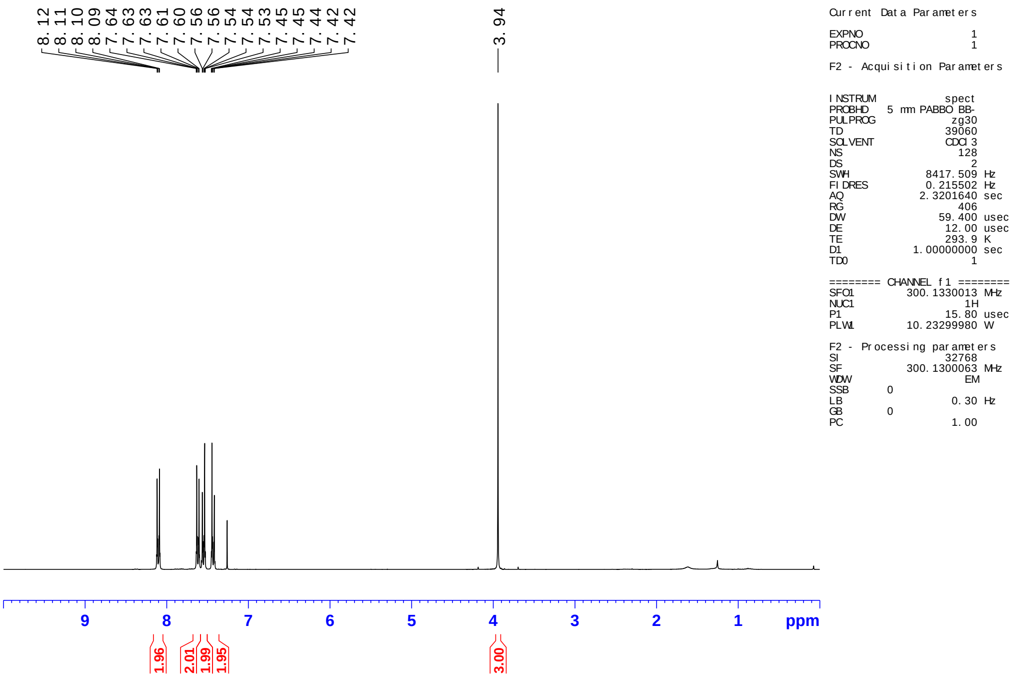

Figure S29: ^{1}H NMR spectrum of **2d**.

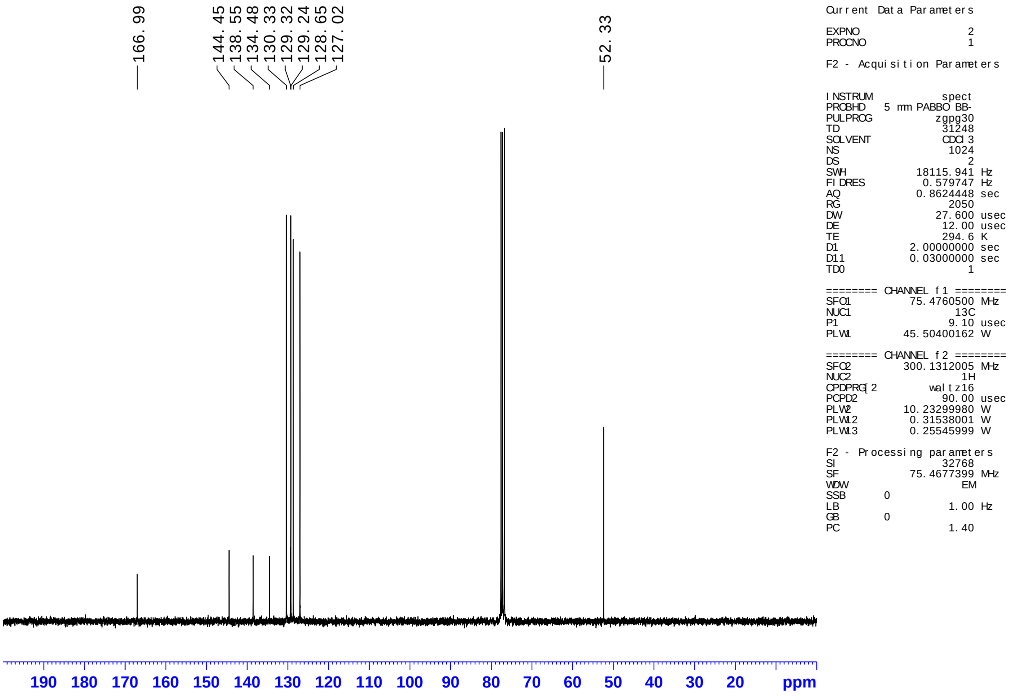

Figure S30: ^{13}C NMR spectrum of **2d**.

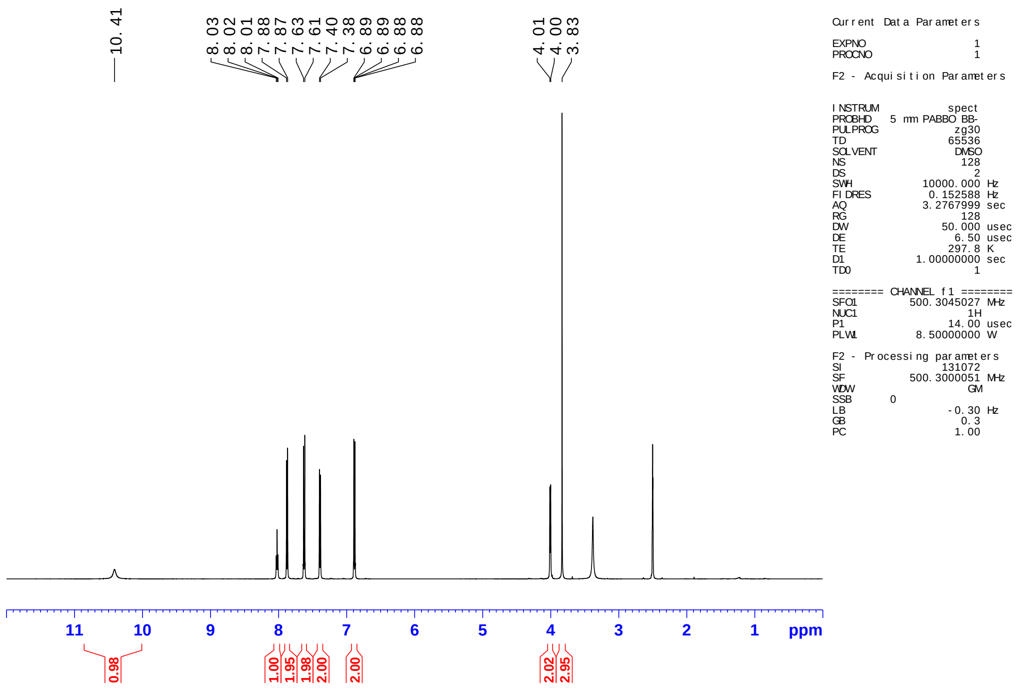

Figure S31: ^{1}H NMR spectrum of **1e**.

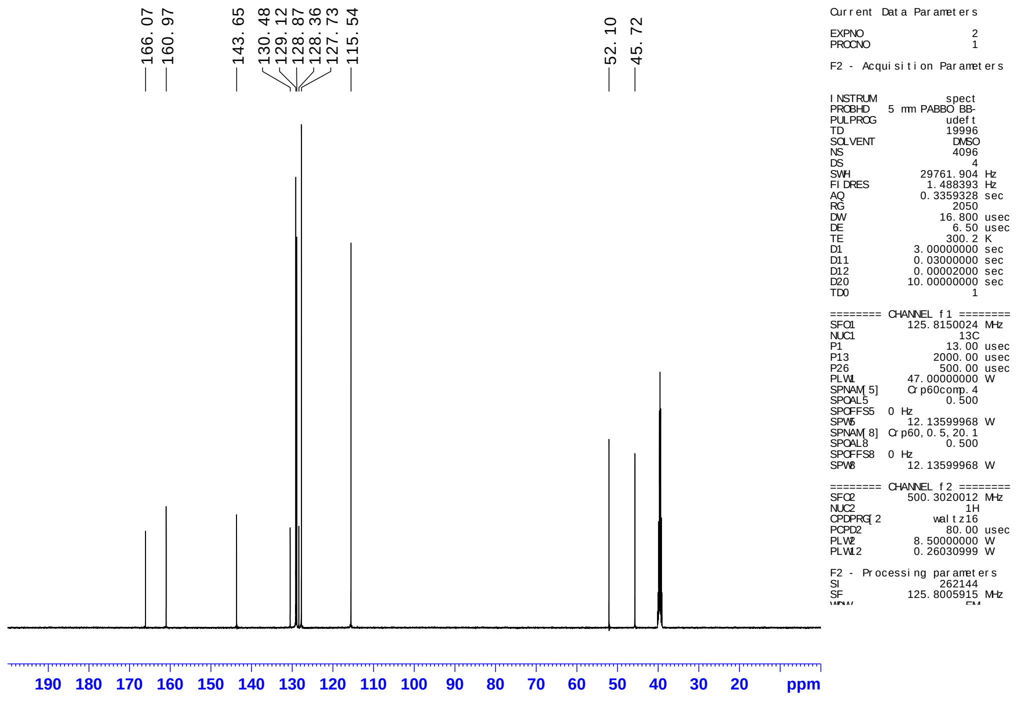

Figure S32: ^{13}C NMR spectrum of **1e**.

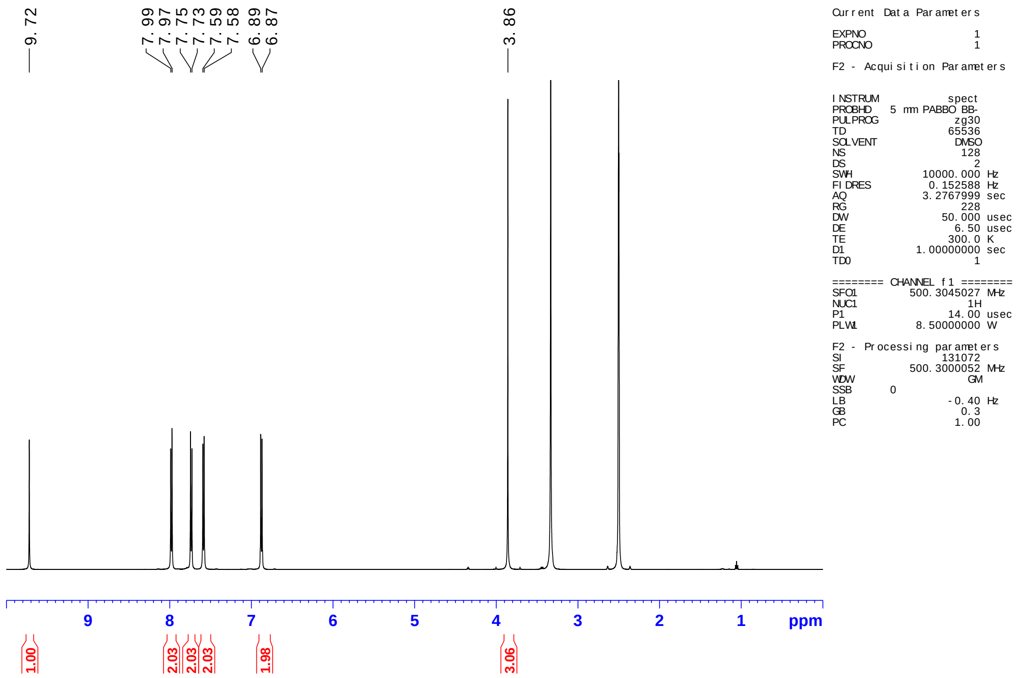

Figure S33: ^{1}H NMR spectrum of **2e**.

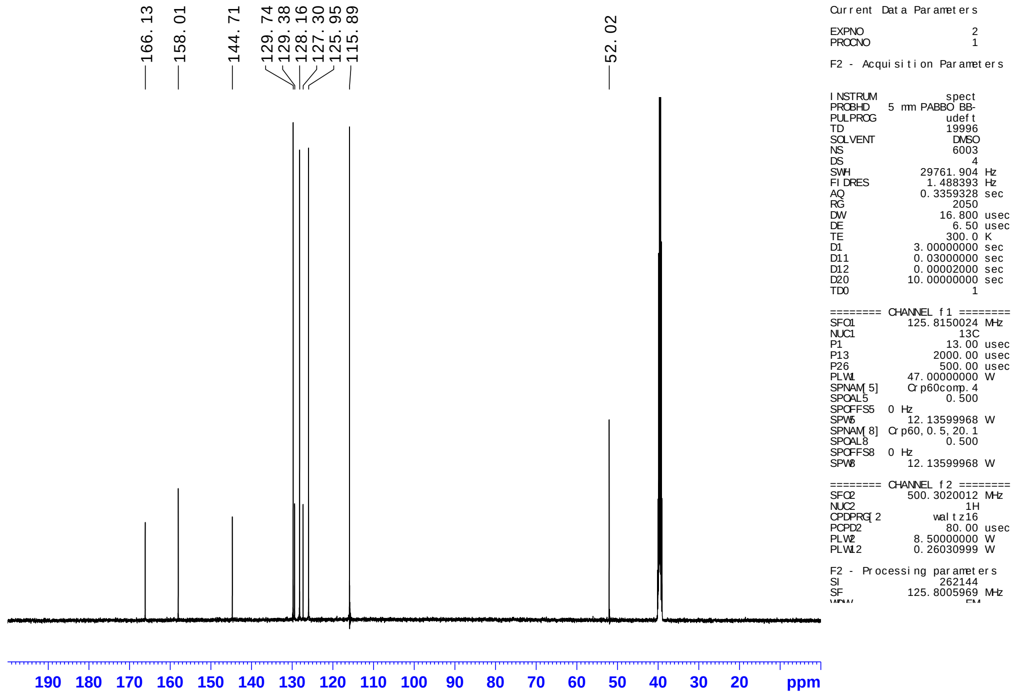

Figure S34: ^{13}C NMR spectrum of **2e**.

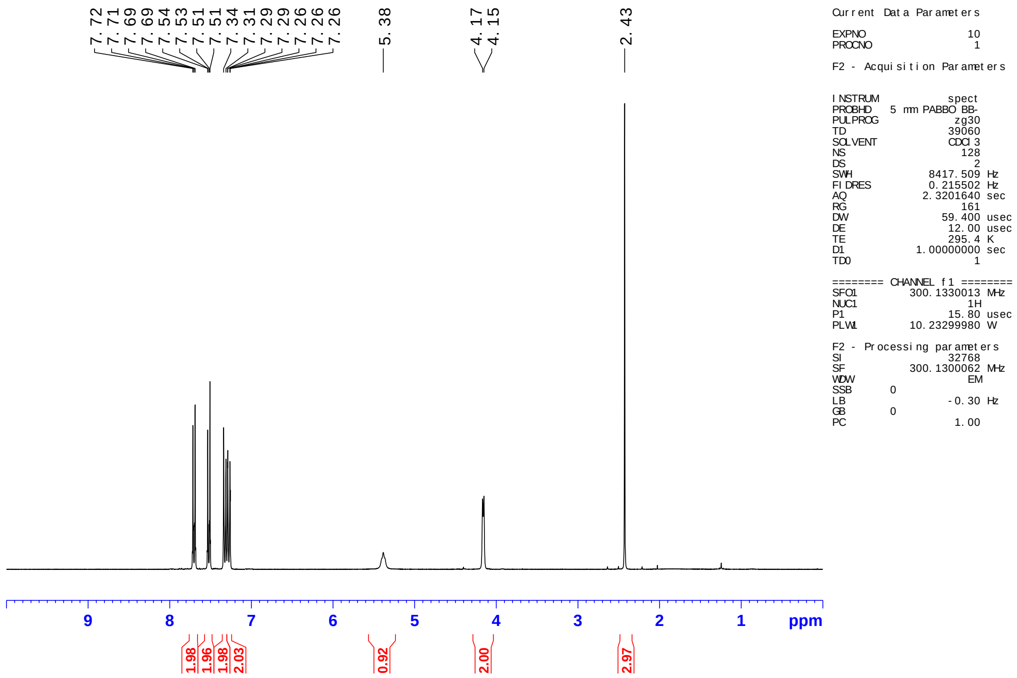

Figure S35: ^{1}H NMR spectrum of **1f**.

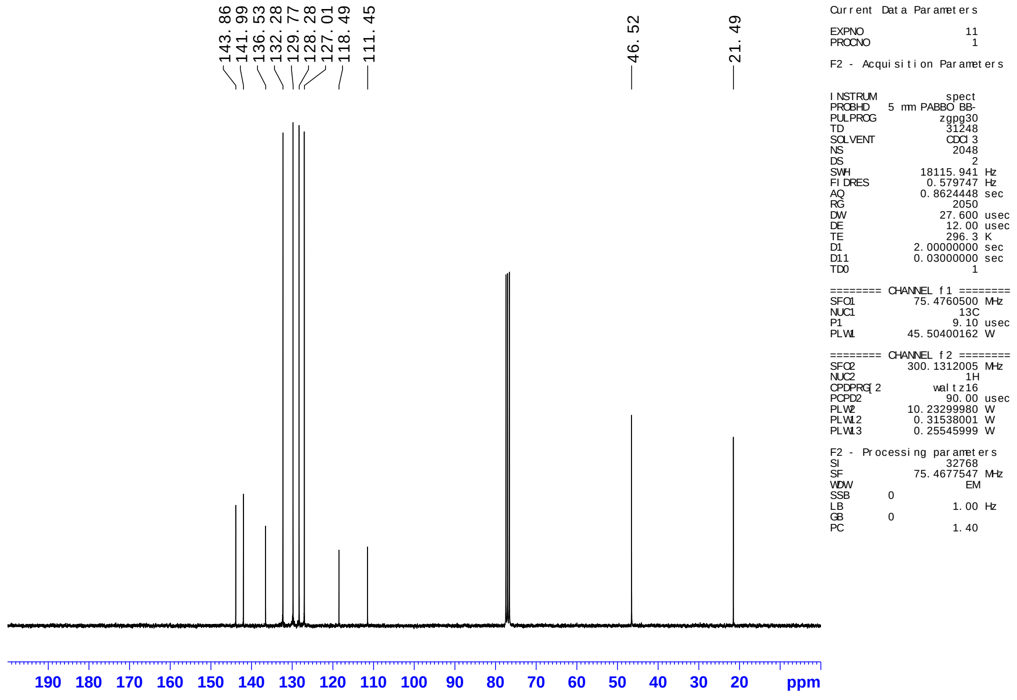

Figure S36: ^{13}C NMR spectrum of **1f**.

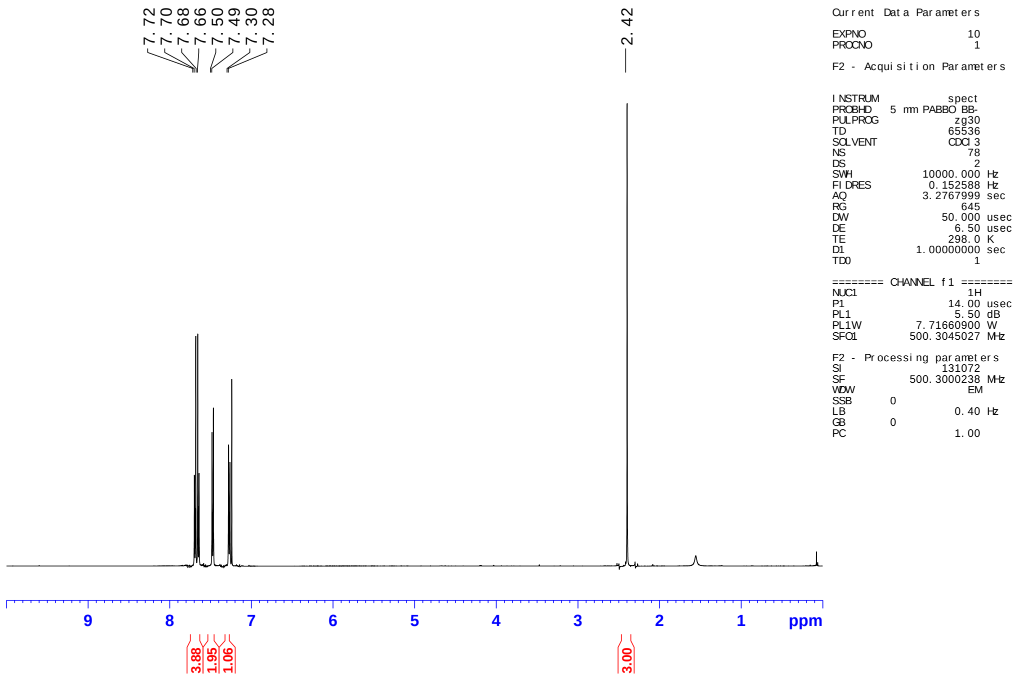

Figure S37: ^{1}H NMR spectrum of **2f**.

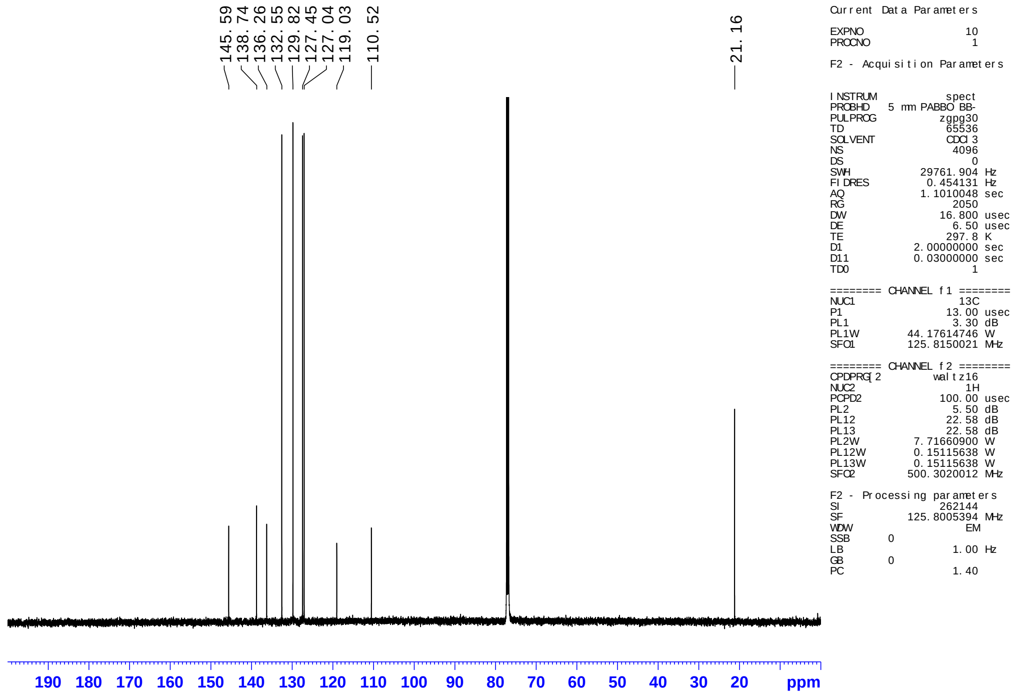

Figure S38: ^{13}C NMR spectrum of **2f**.

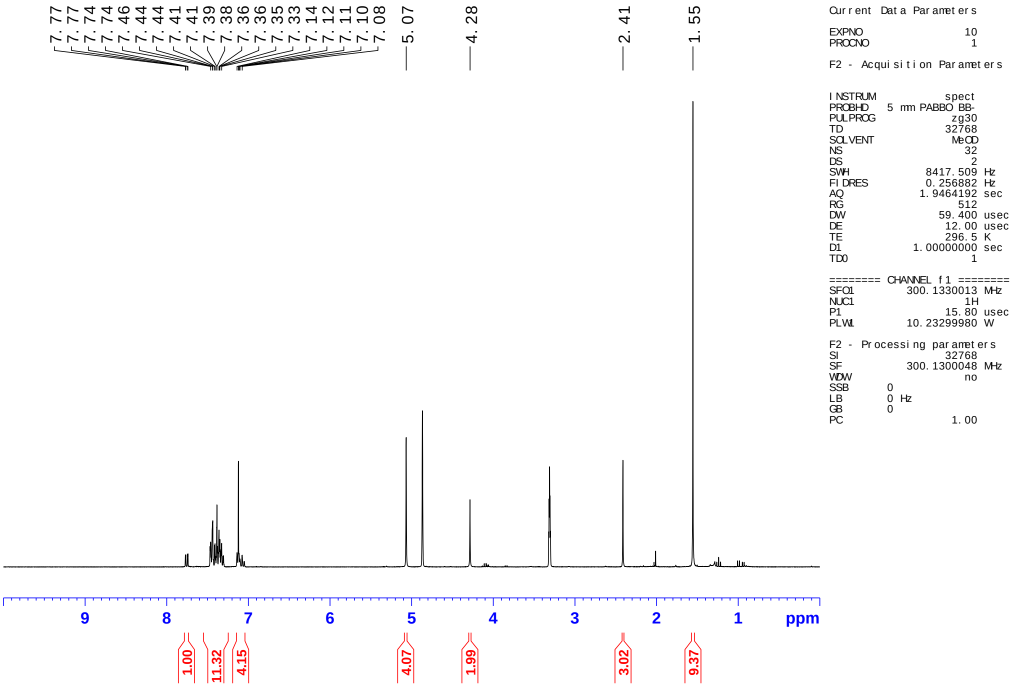

Figure S39: ^{1}H NMR spectrum of **1g**.

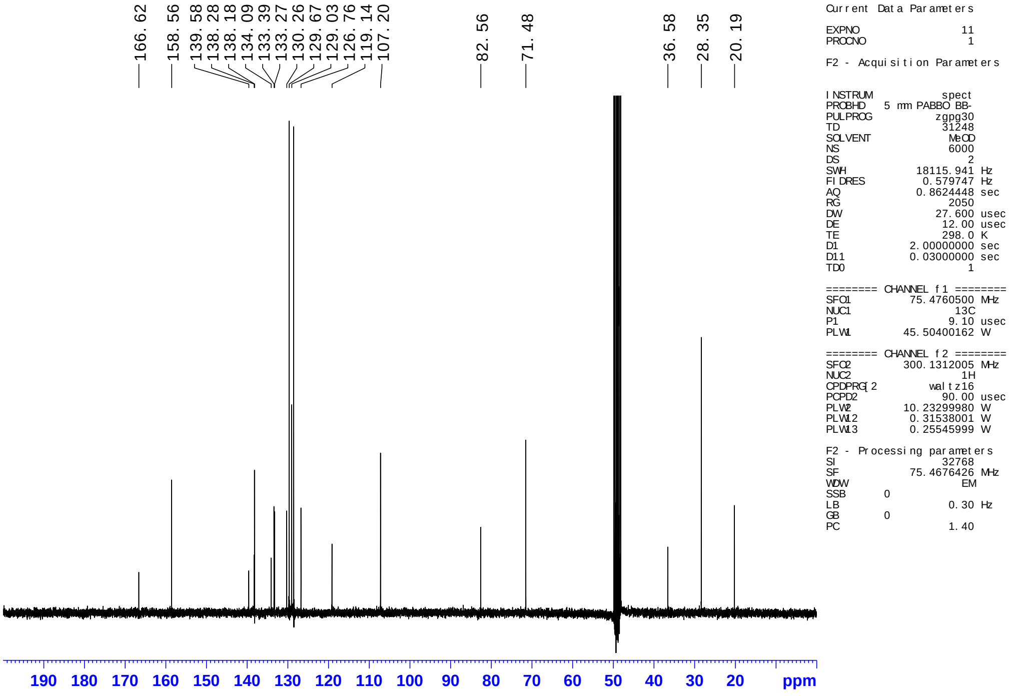

Figure S40: ^{13}C NMR spectrum of **1g**.

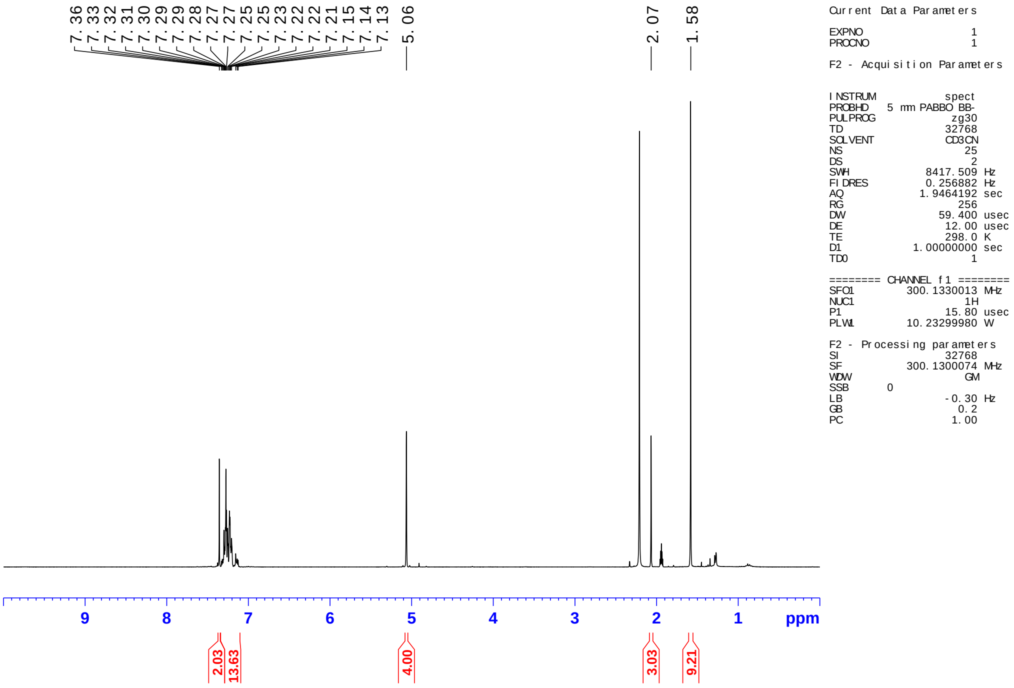

Figure S41: ^{1}H NMR spectrum of **2g**.

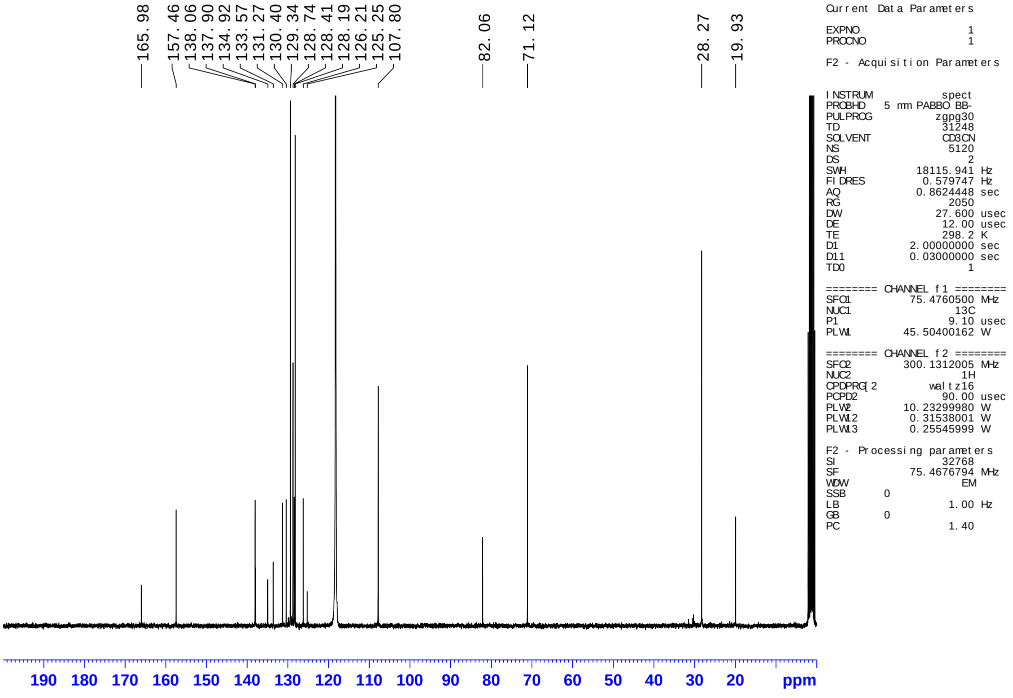

Figure S42: ^{13}C NMR spectrum of **2g**.

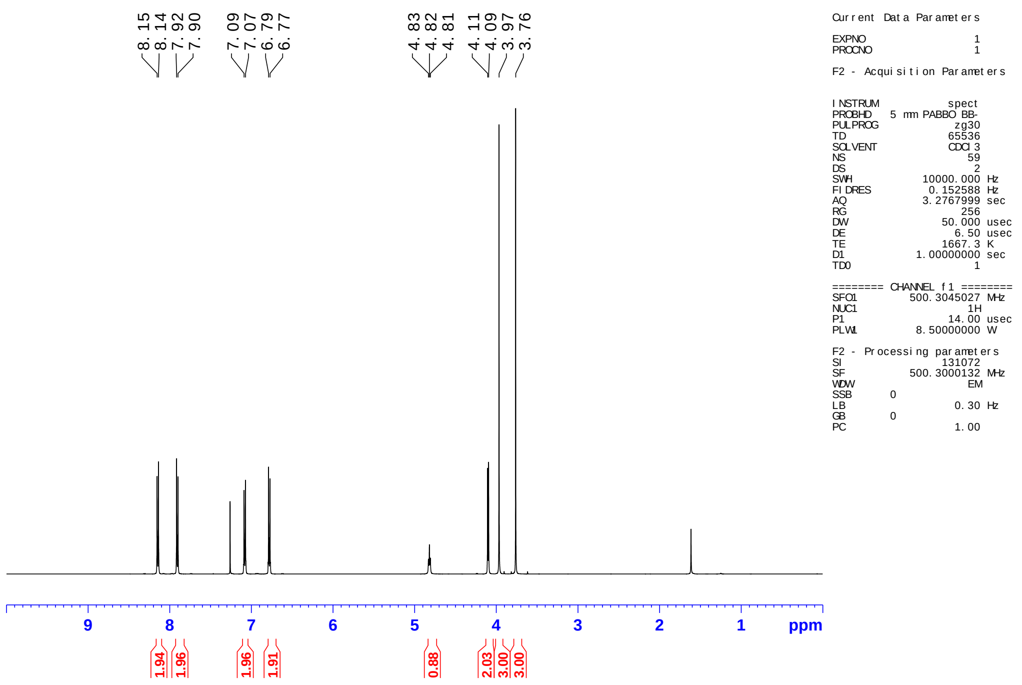

Figure S43: ^{1}H NMR spectrum of **1i**.

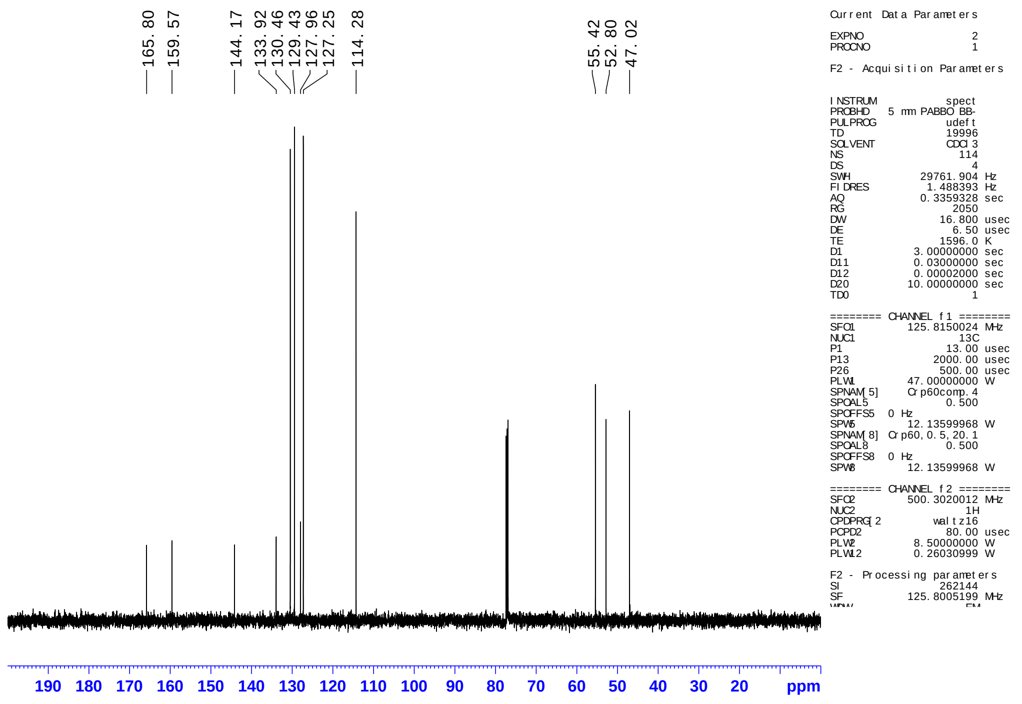

Figure S44: ^{13}C NMR spectrum of **1i**.

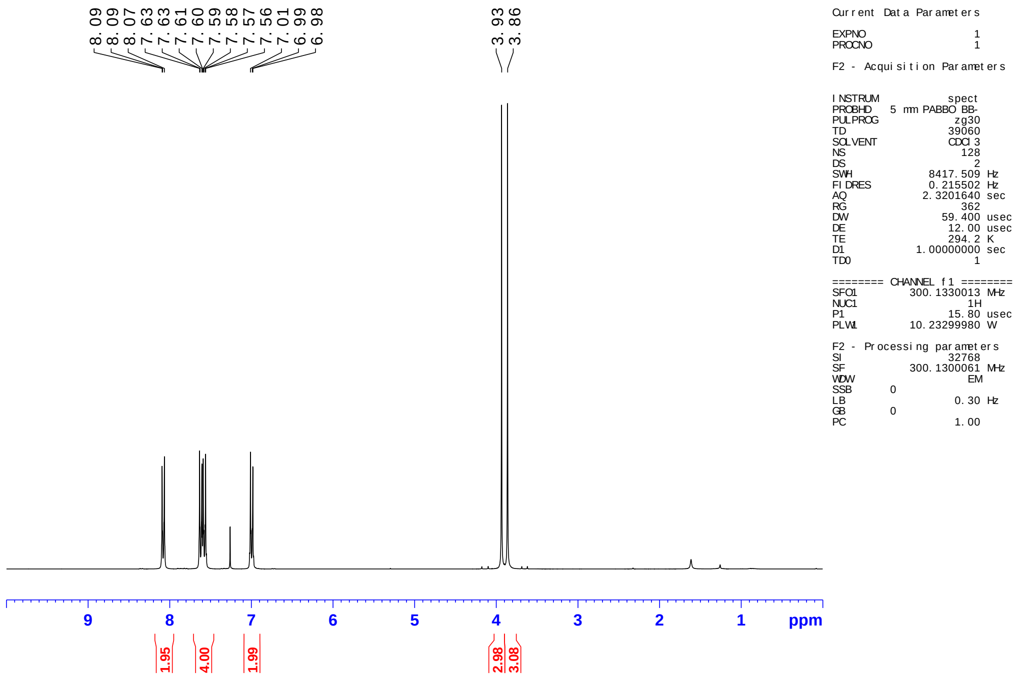

Figure S45: ^{1}H NMR spectrum of **2i**.

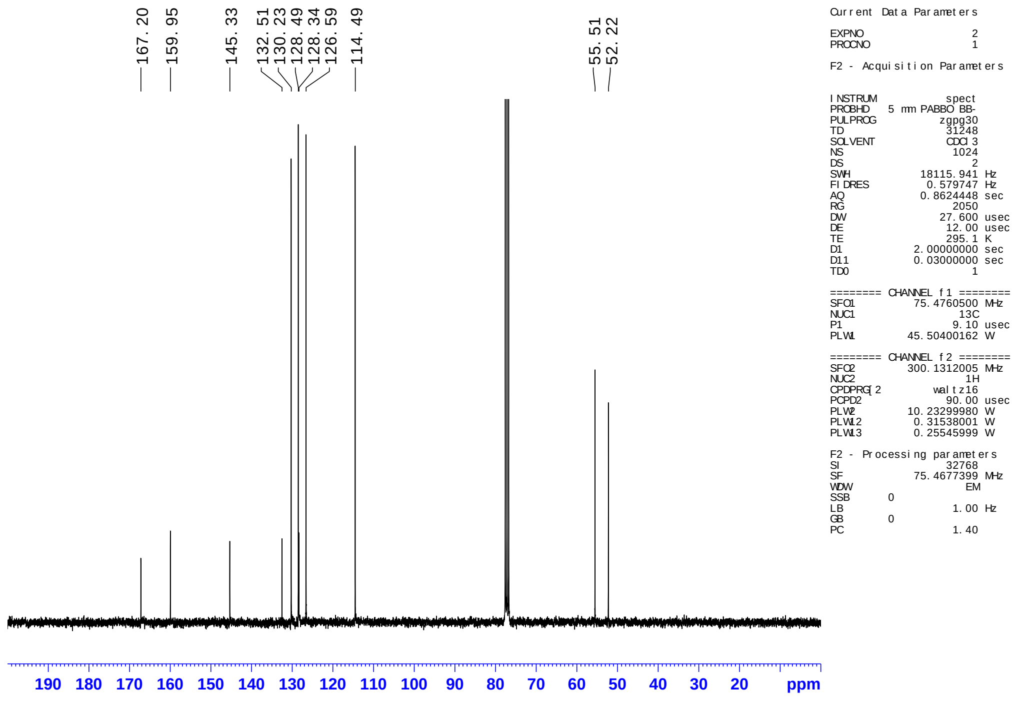

Figure S46: ^{13}C NMR spectrum of **2i**.

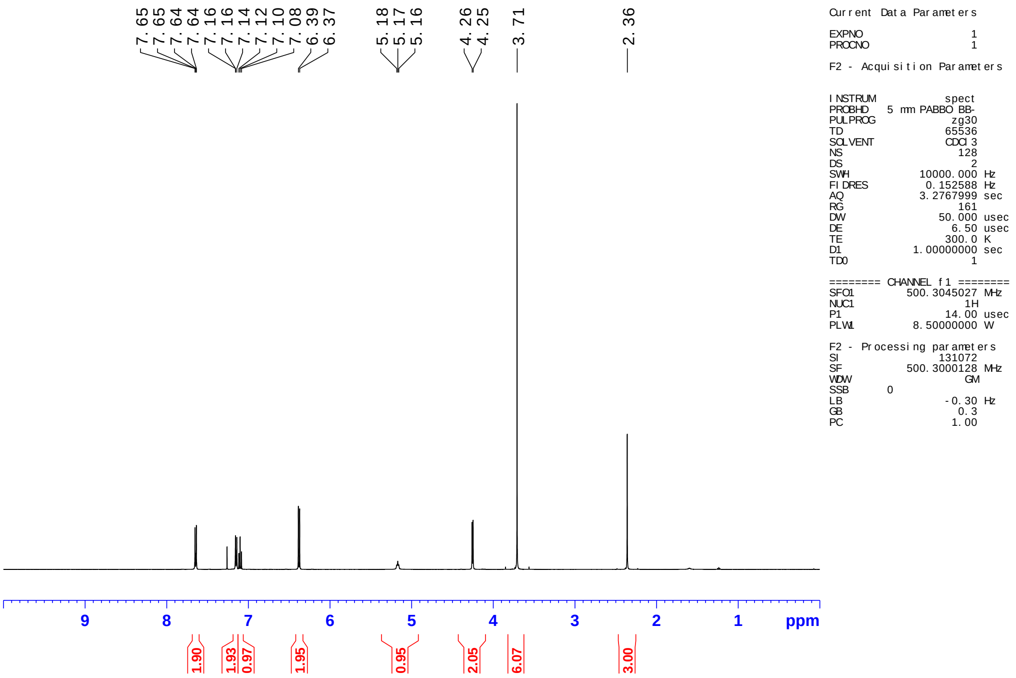

Figure S47: ^{1}H NMR spectrum of **1j**.

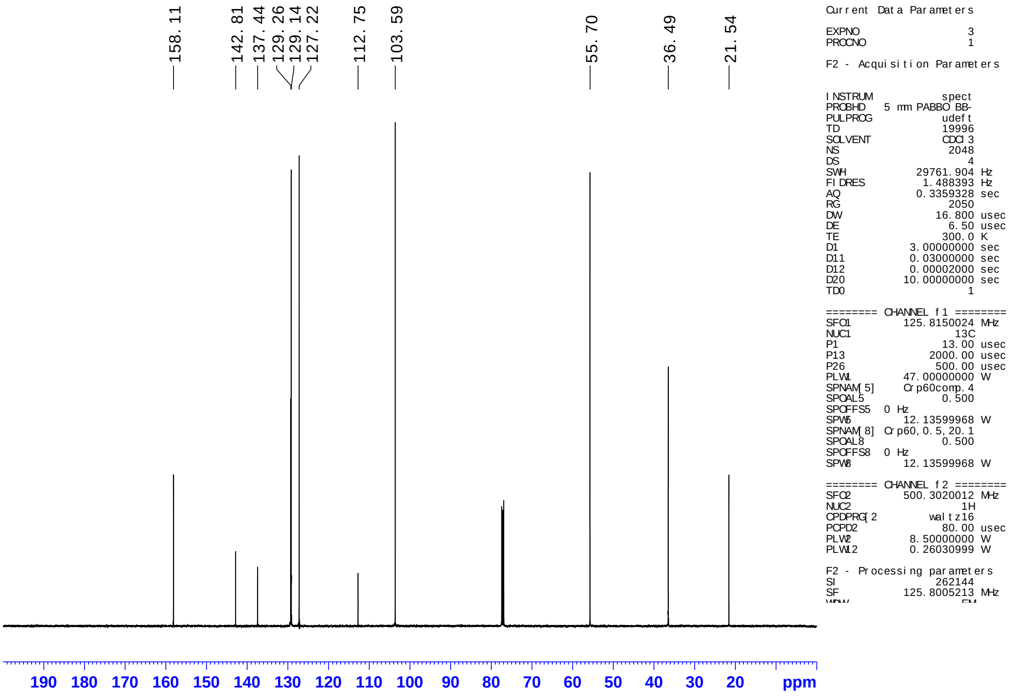

Figure S48: ^{13}C NMR spectrum of **1j**.

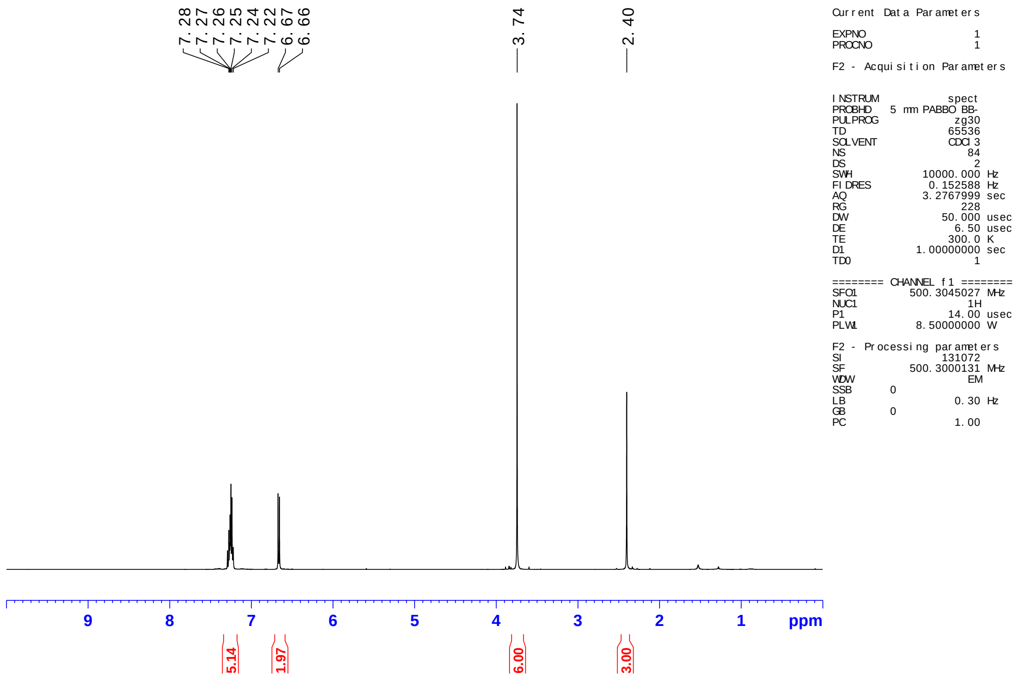

Figure S49: ^{1}H NMR spectrum of **2j**.

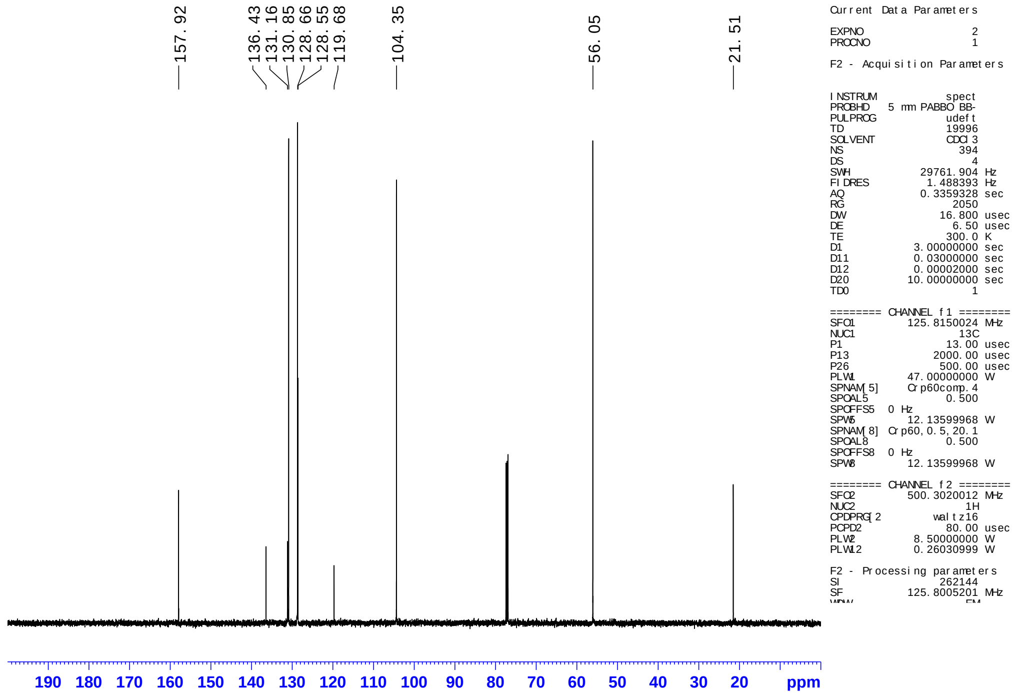

Figure S50: ^{13}C NMR spectrum of **2j**.

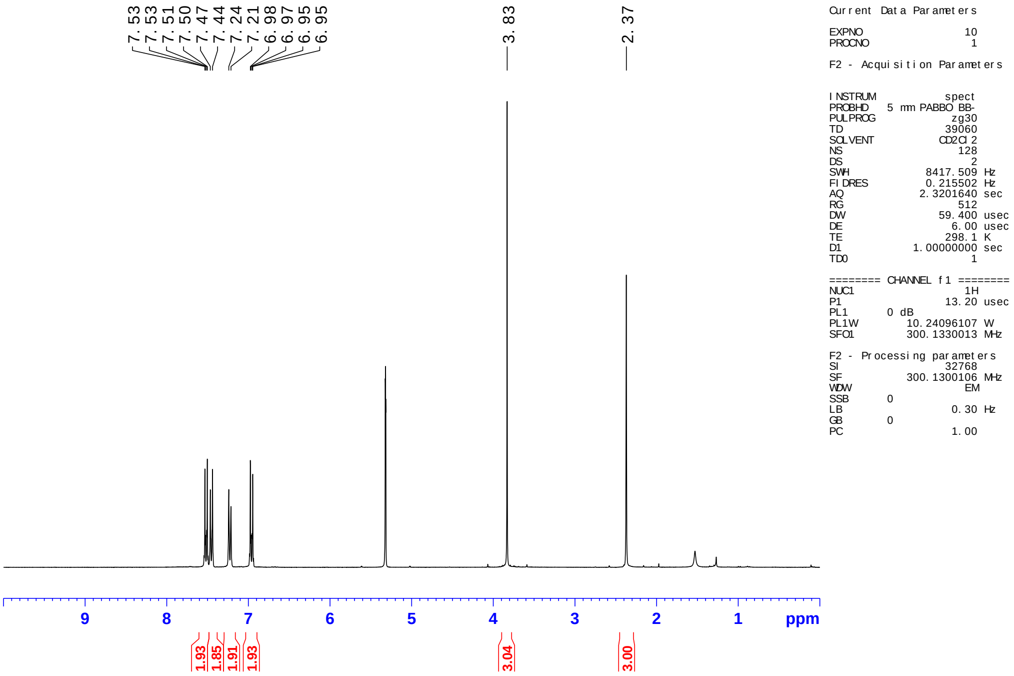

Figure S51: ^{1}H NMR spectrum of **2k**.

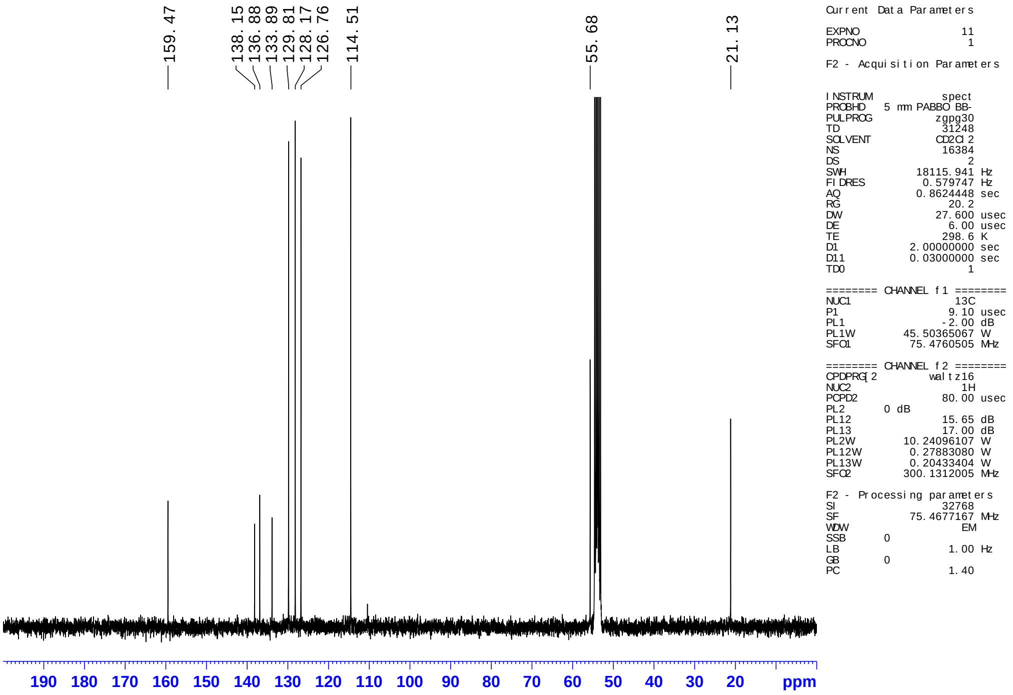

Figure S52: ^{13}C NMR spectrum of **2k**.

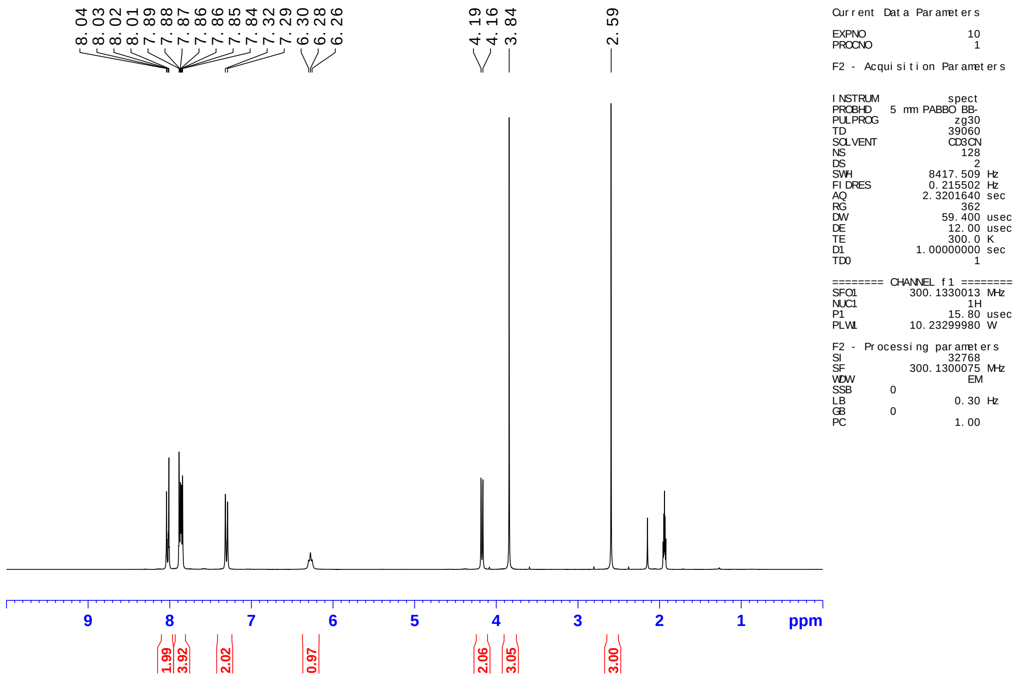

Figure S53: ^{1}H NMR spectrum of **1l**.

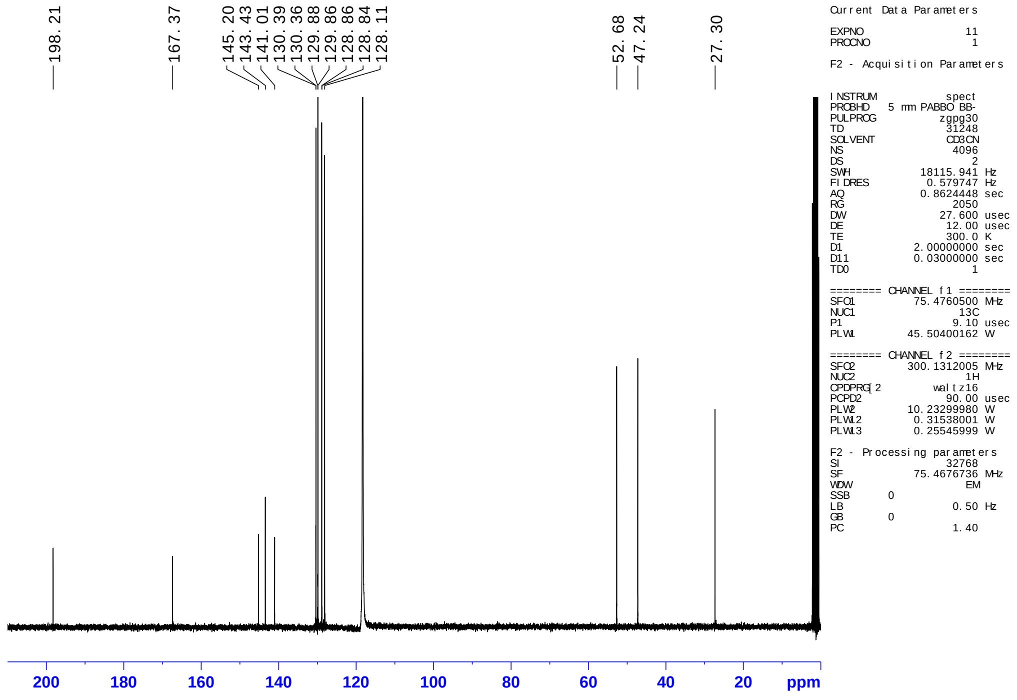

Figure S54: ^{13}C NMR spectrum of **1l**.

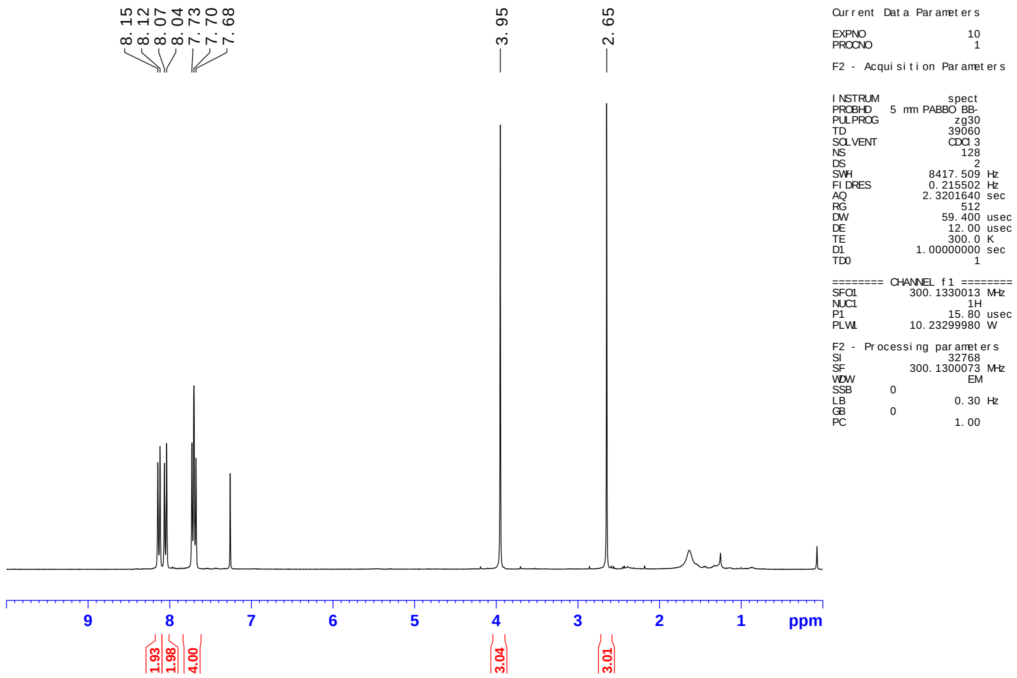

Figure S55: ^{1}H NMR spectrum of **2l**.

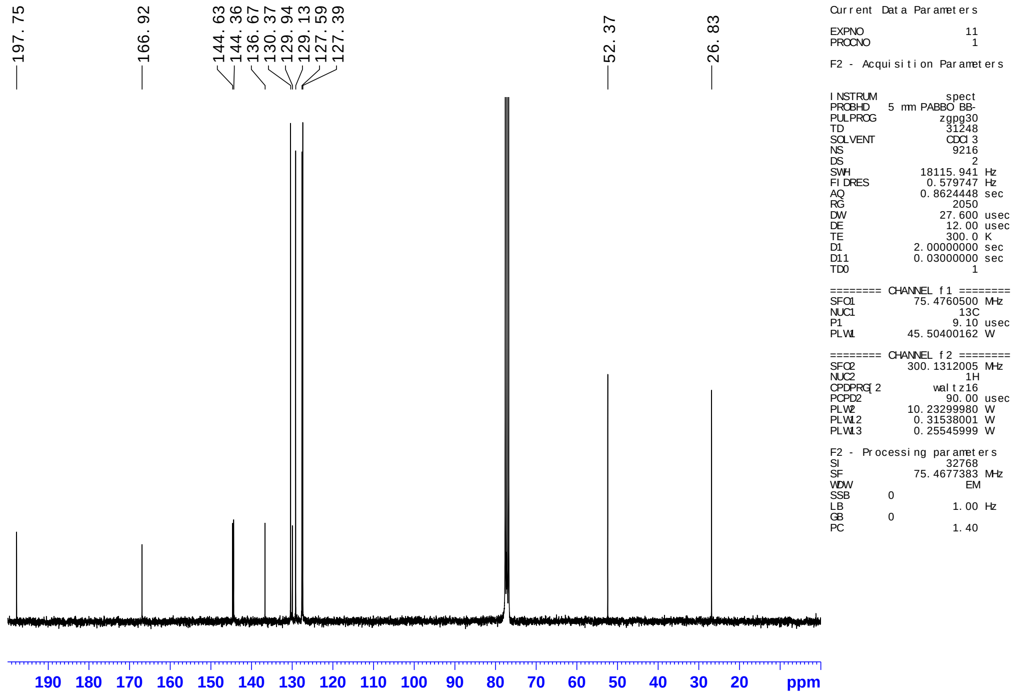

Figure S56: ^{13}C NMR spectrum of **2l**.

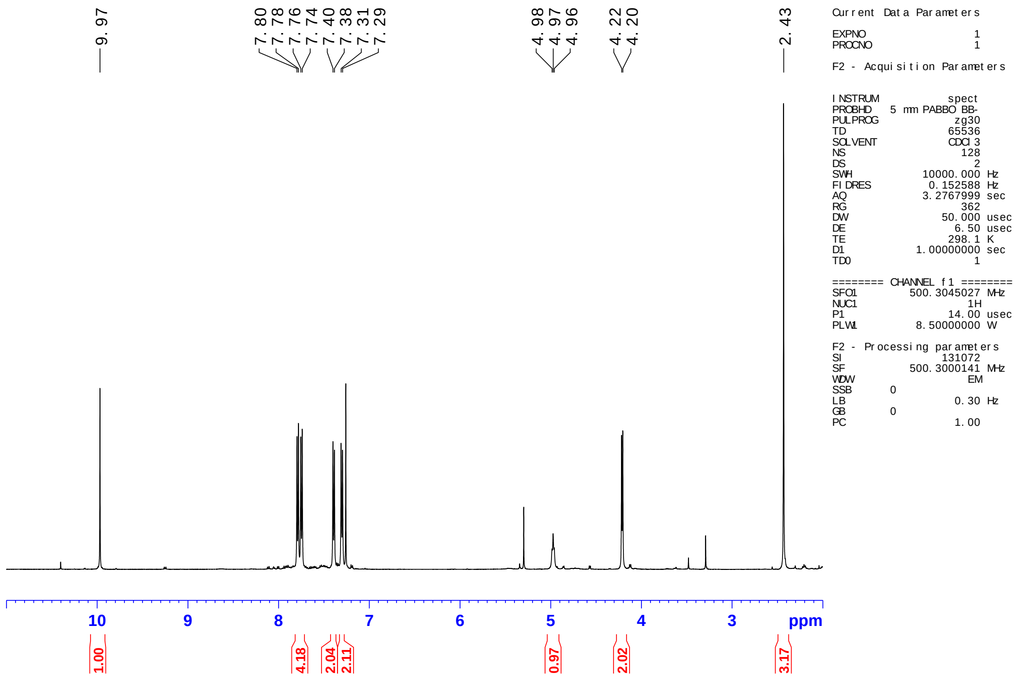

Figure S57: ^{1}H NMR spectrum of **1m**.

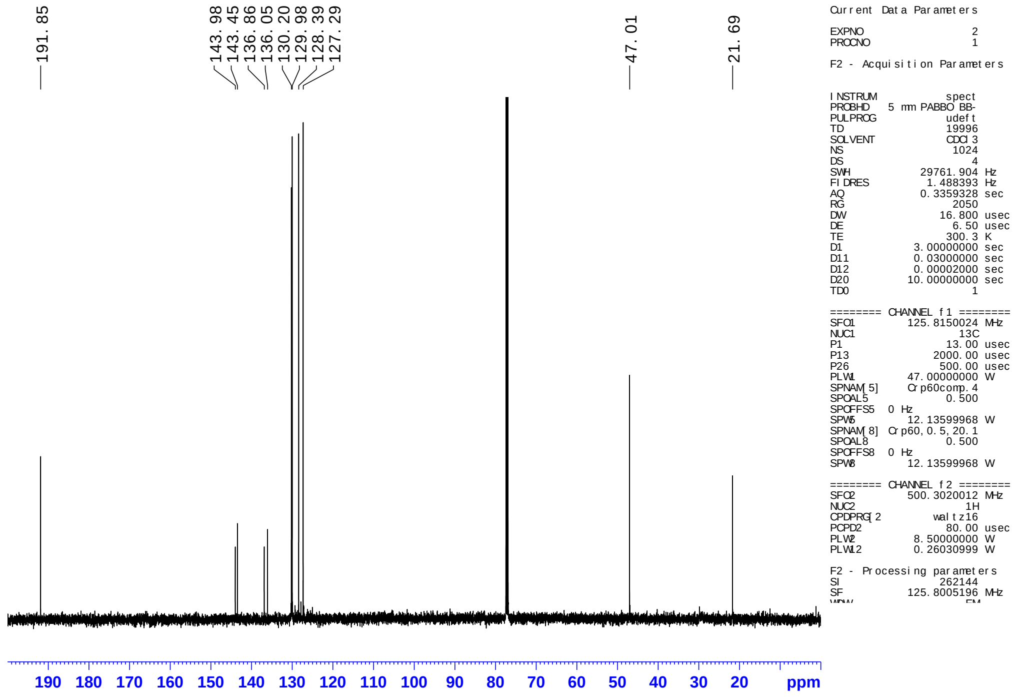

Figure S58: ^{13}C NMR spectrum of **1m**.

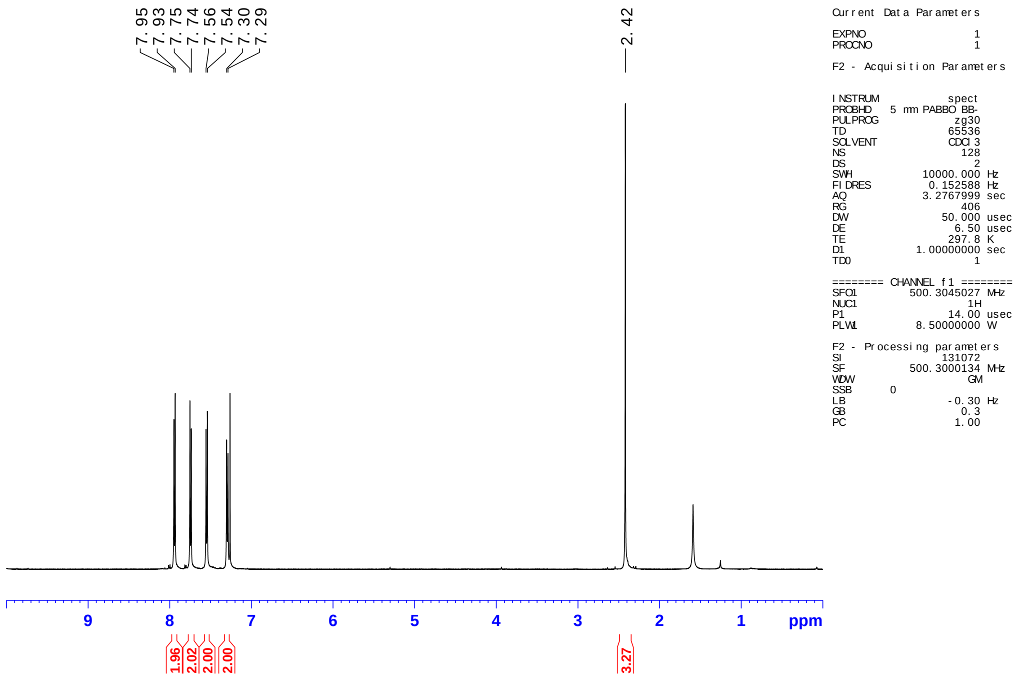

Figure S59: ^{1}H NMR spectrum of **2m**.

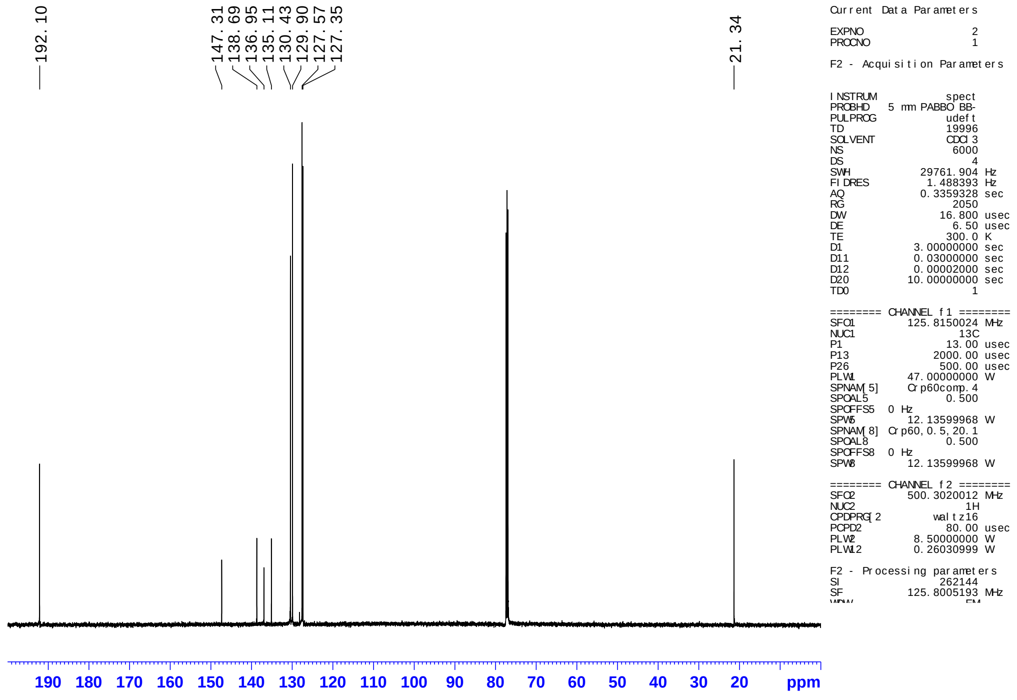

Figure S60: ^{13}C NMR spectrum of **2m**.

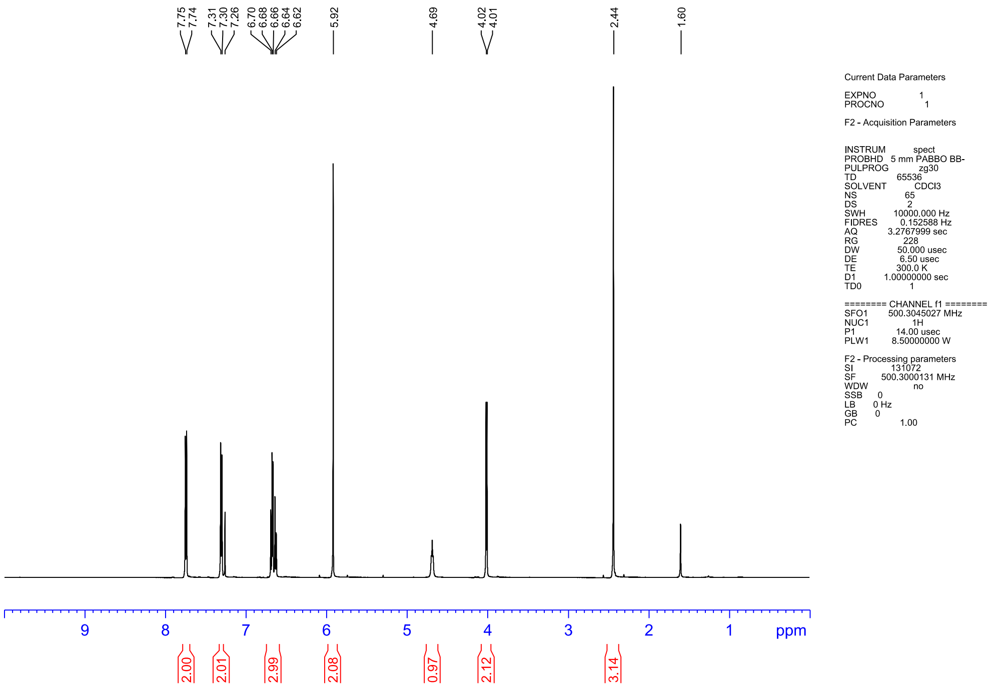

Figure S61: ^{1}H NMR spectrum of **1n**.

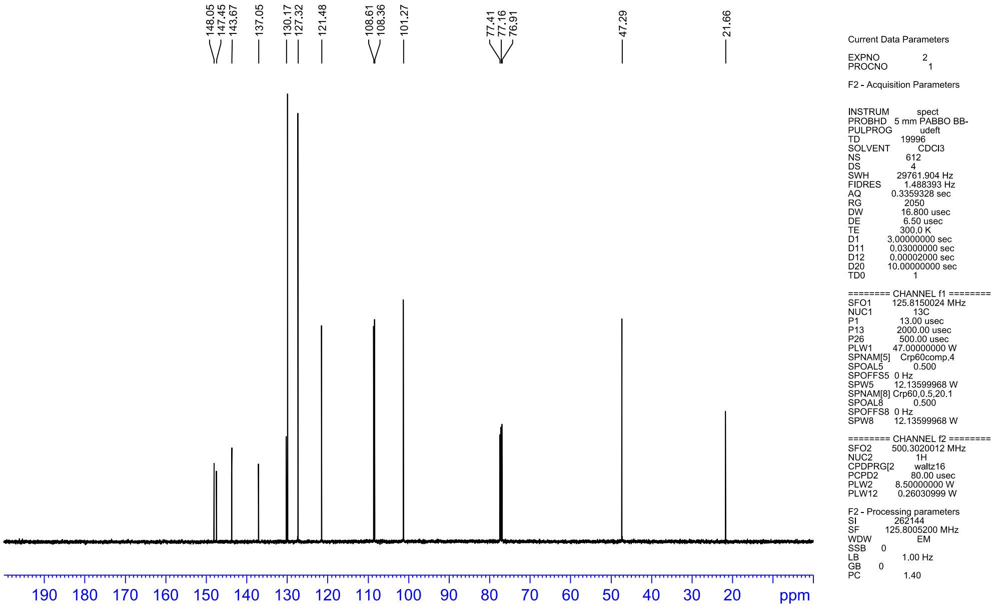

Figure S62: ^{13}C NMR spectrum of **1n**.

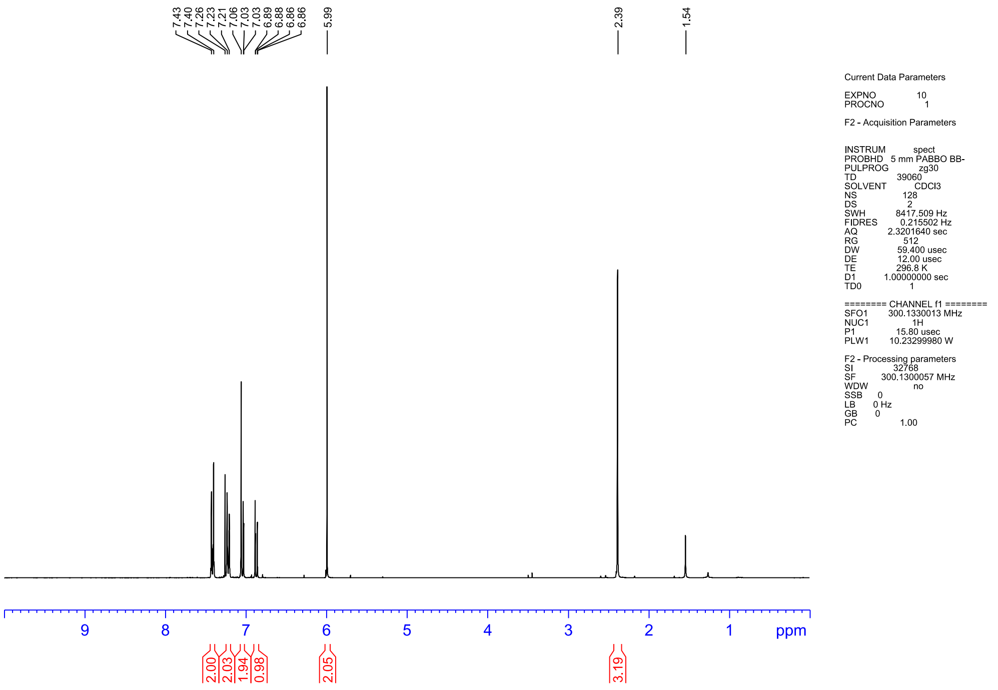

Figure S63: ^{1}H NMR spectrum of **2n**.

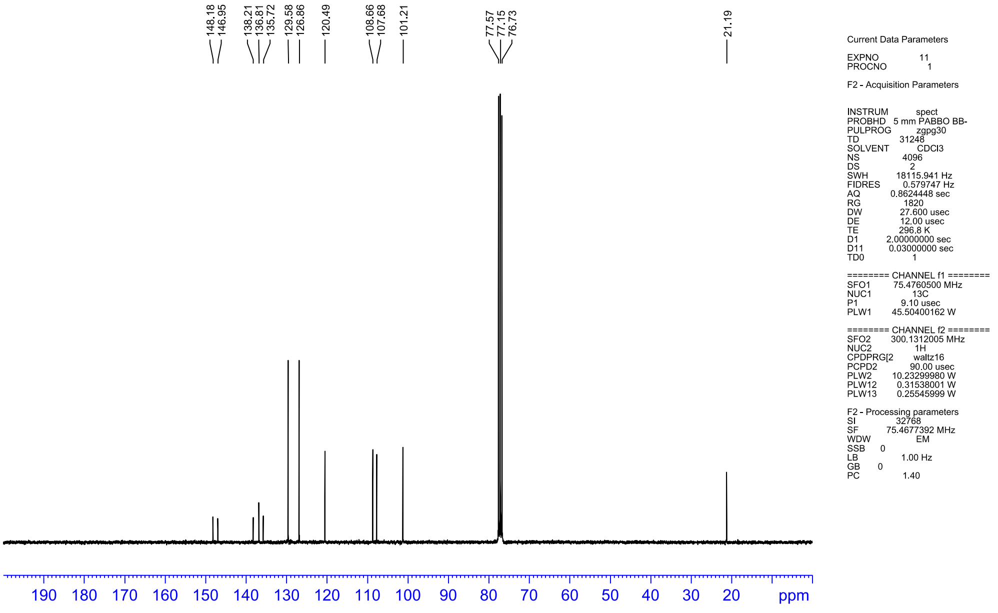

Figure S64: ^{13}C NMR spectrum of **2n**.

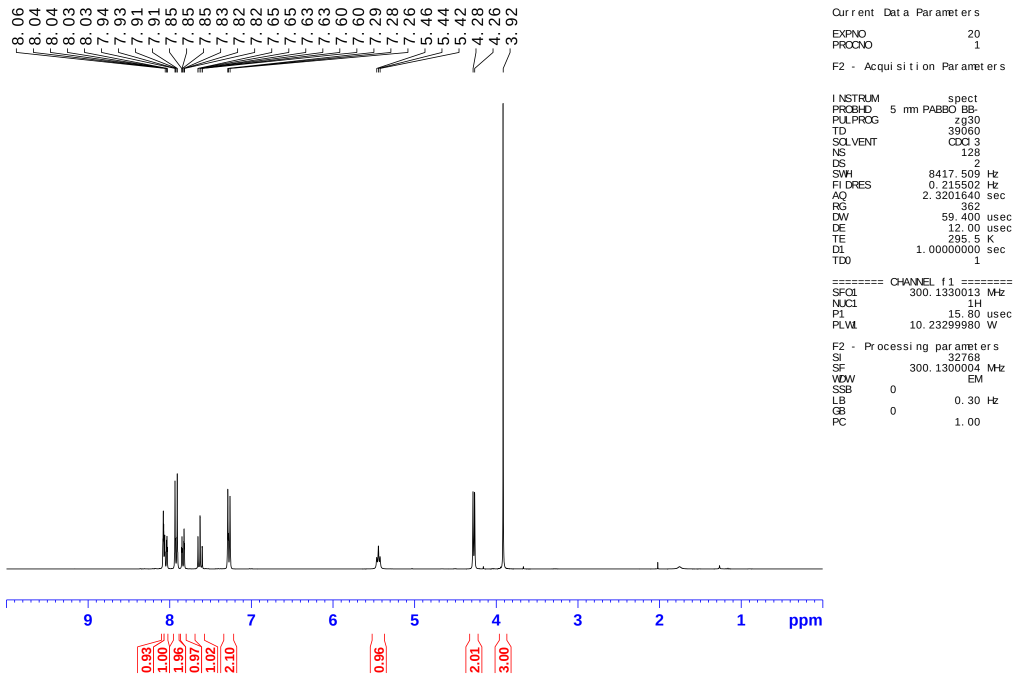

Figure S65: ^{1}H NMR spectrum of **1o**.

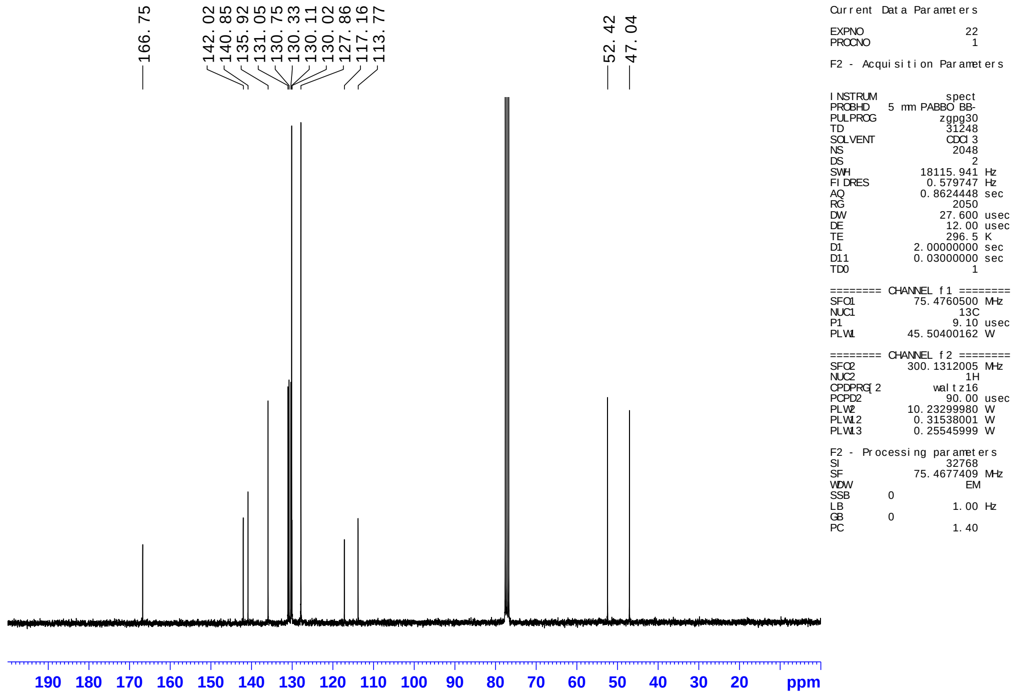

Figure S66: ^{13}C NMR spectrum of **1o**.

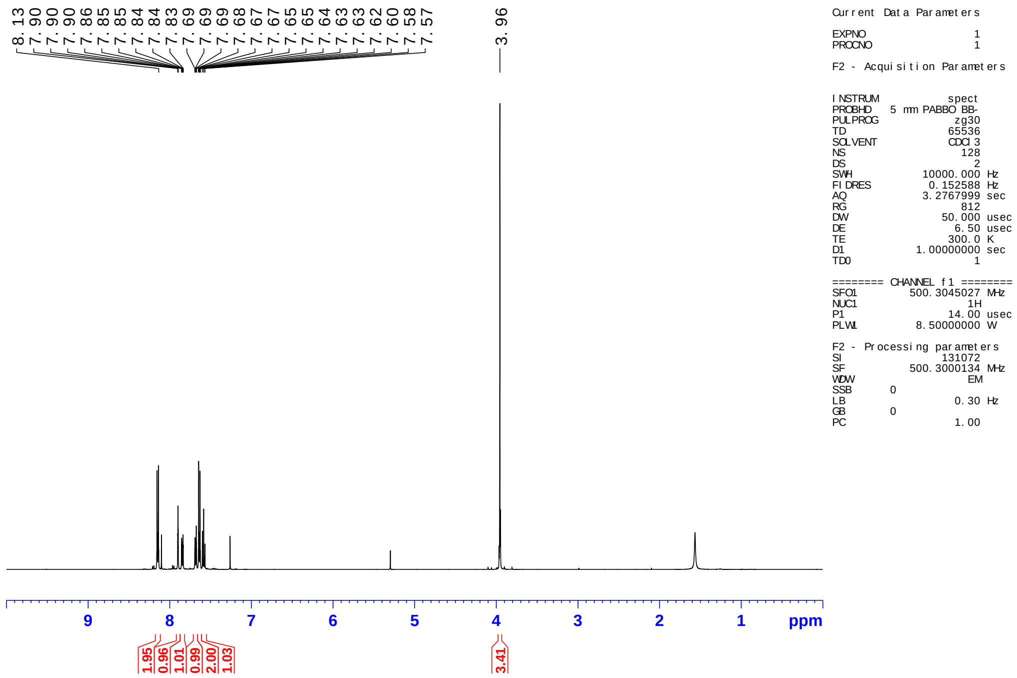

Figure S67: ^{1}H NMR spectrum of **2o**.

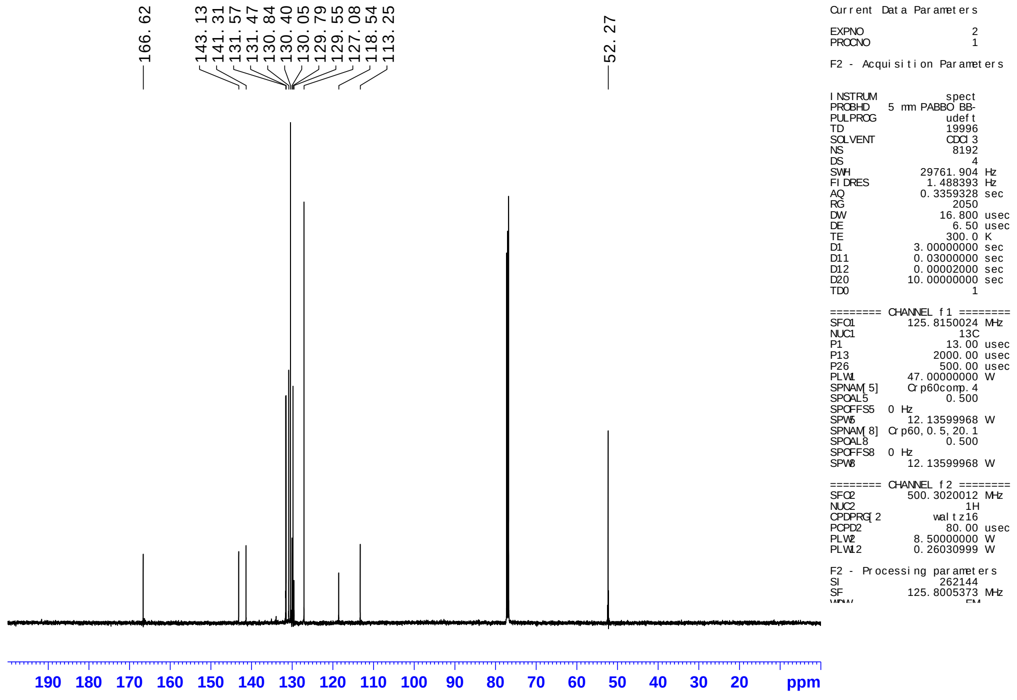

Figure S68: ^{13}C NMR spectrum of **2o**.

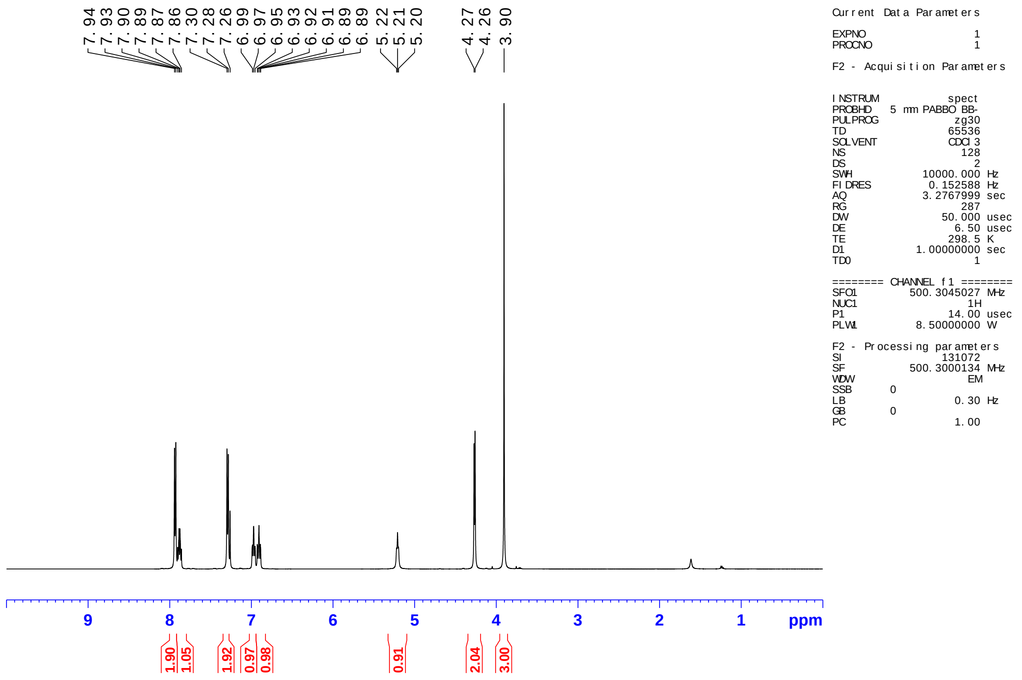

Figure S69: ^{1}H NMR spectrum of **1r**.

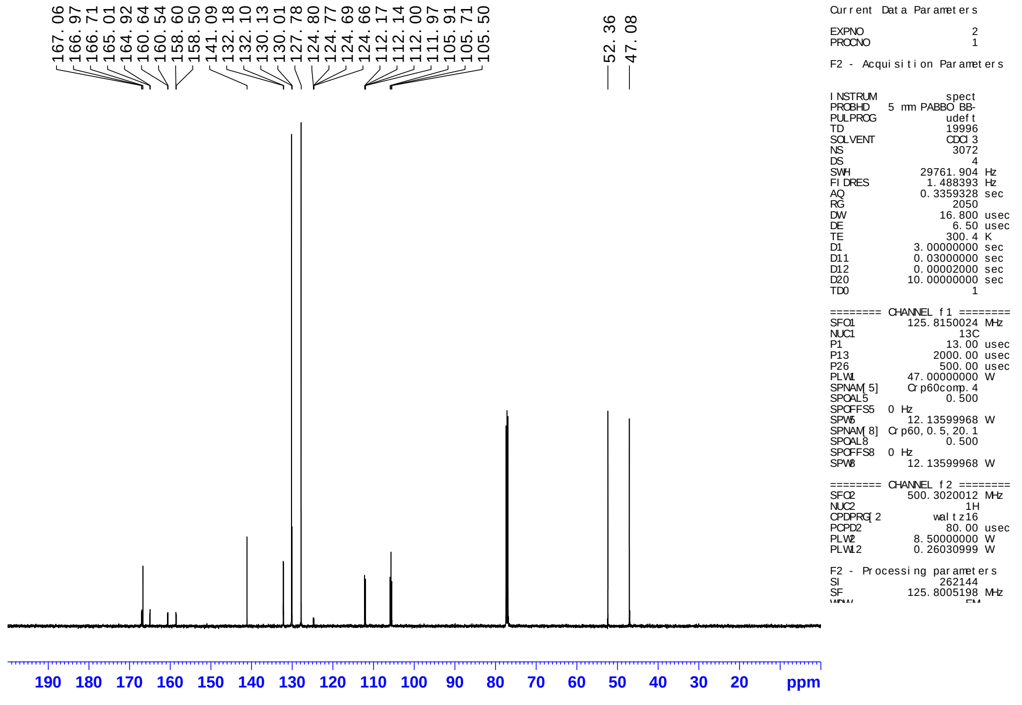

Figure S70: ^{13}C NMR spectrum of **1r**.

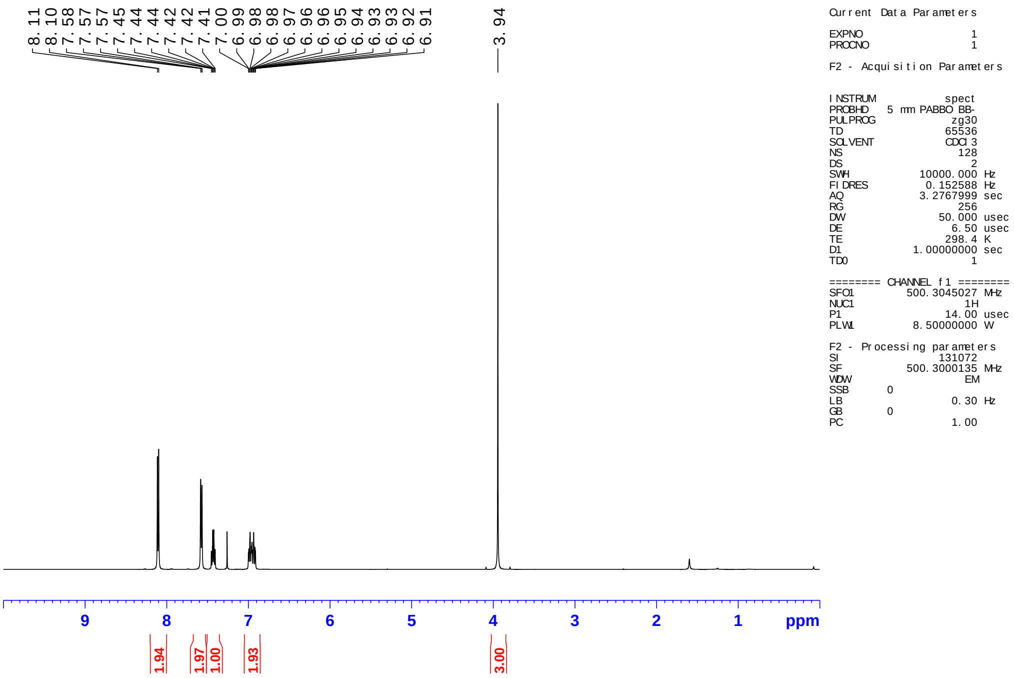

Figure S71: ^{1}H NMR spectrum of **2r**.

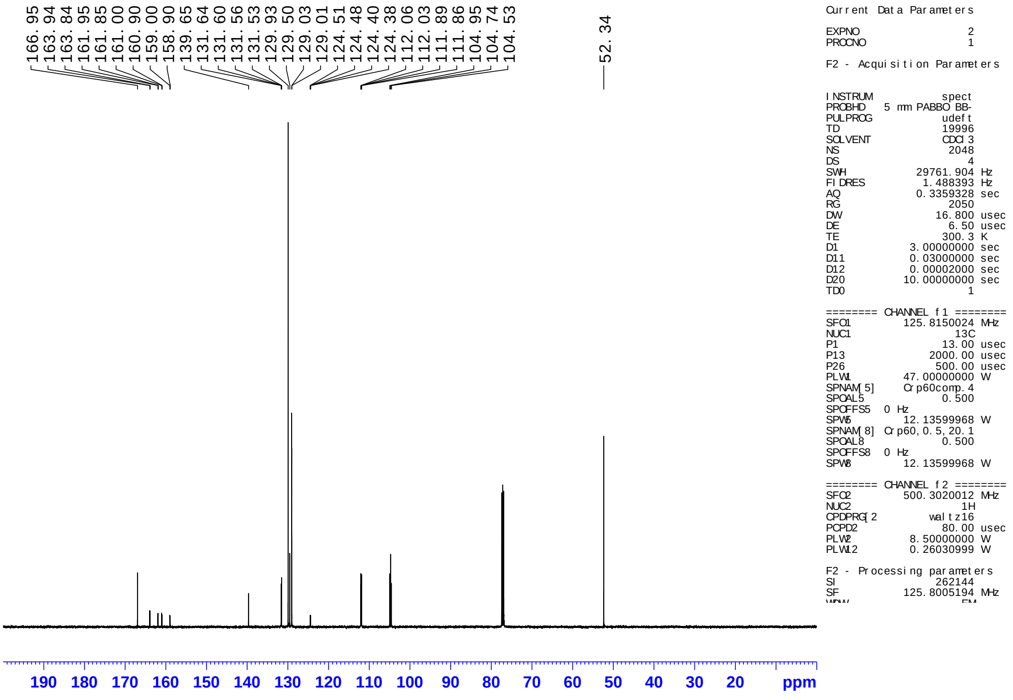

Figure S72: ^{13}C NMR spectrum of **2r**.

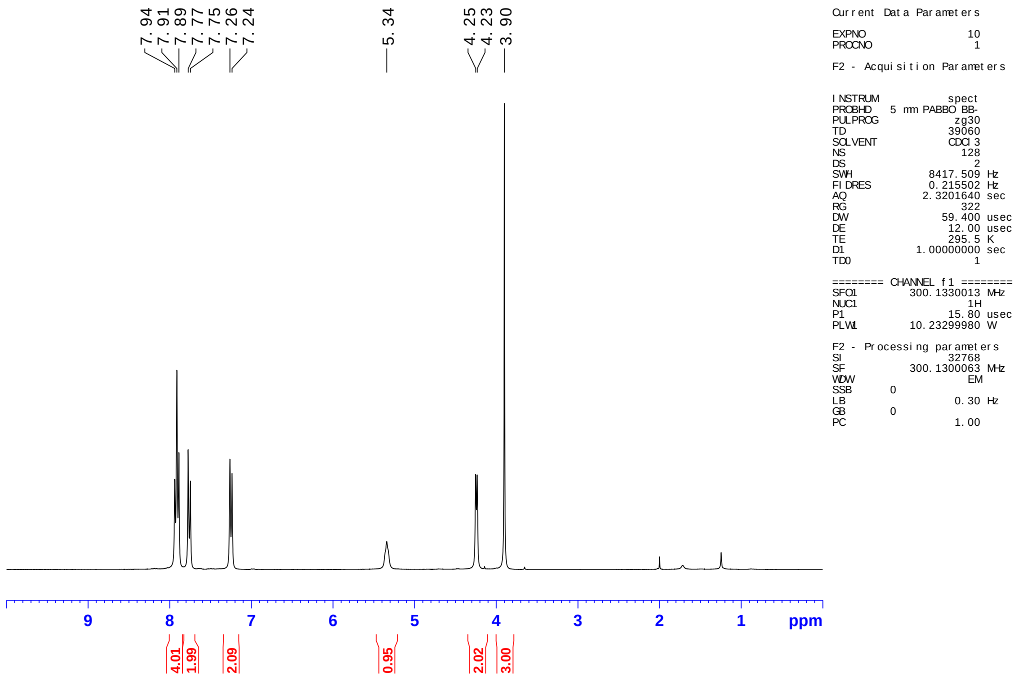

Figure S73: ^{1}H NMR spectrum of **1v**.

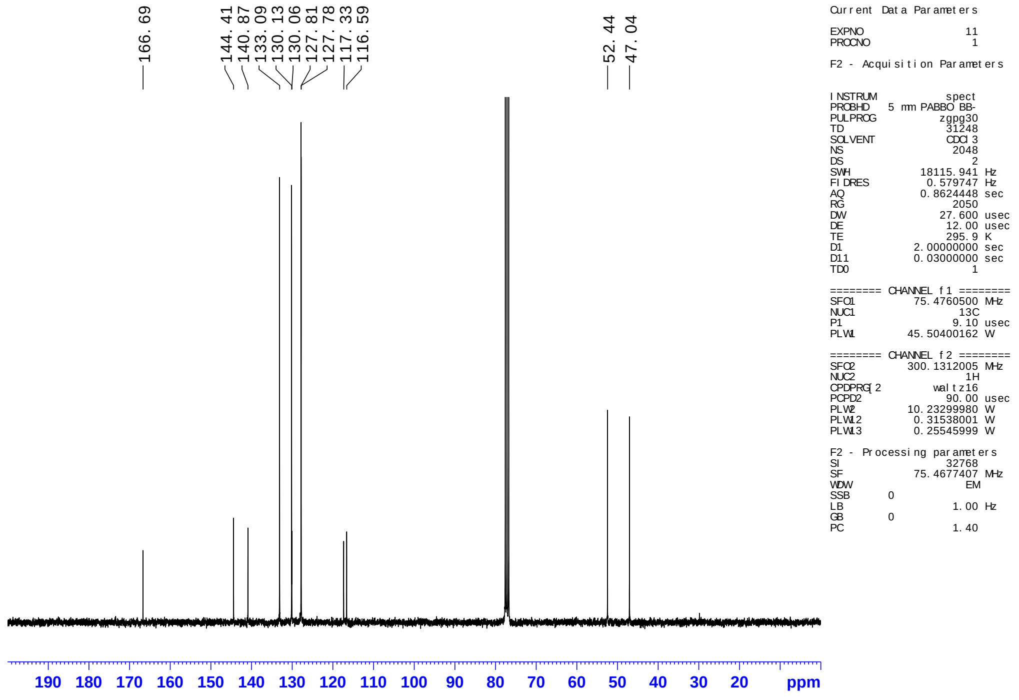

Figure S74: ^{13}C NMR spectrum of **1v**.

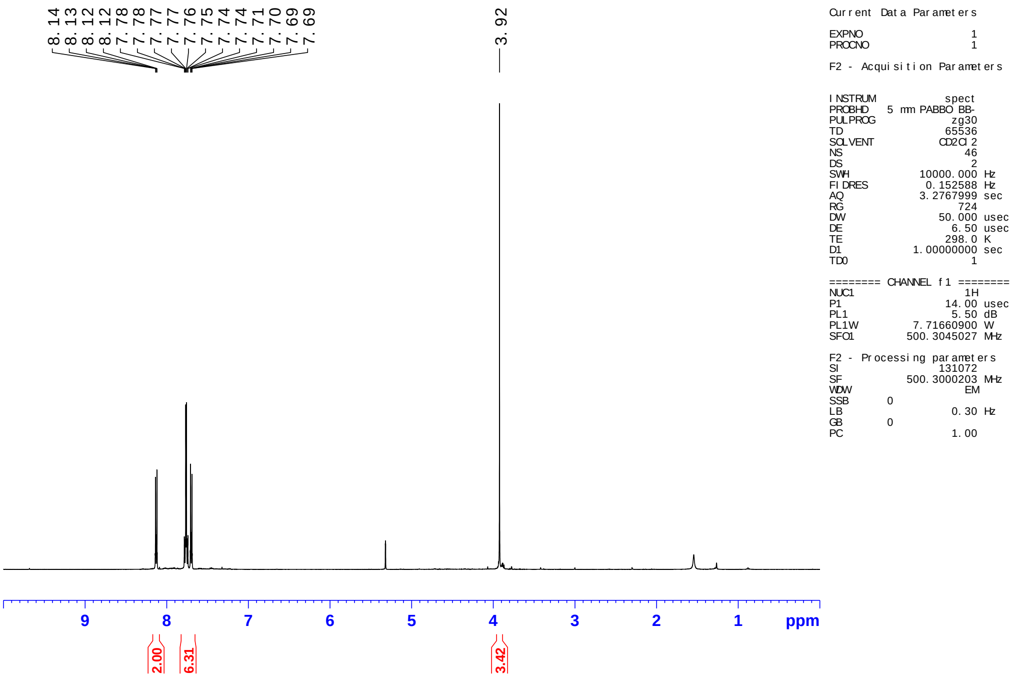

Figure S75: ^{1}H NMR spectrum of **2v**.

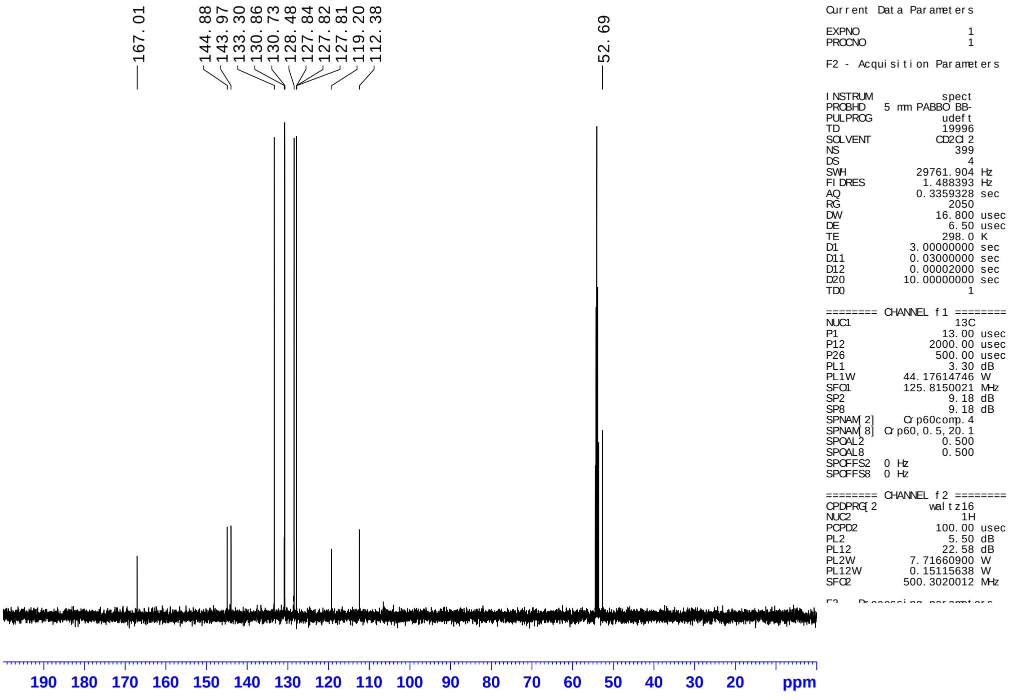

Figure S76: ^{13}C NMR spectrum of **2v**.

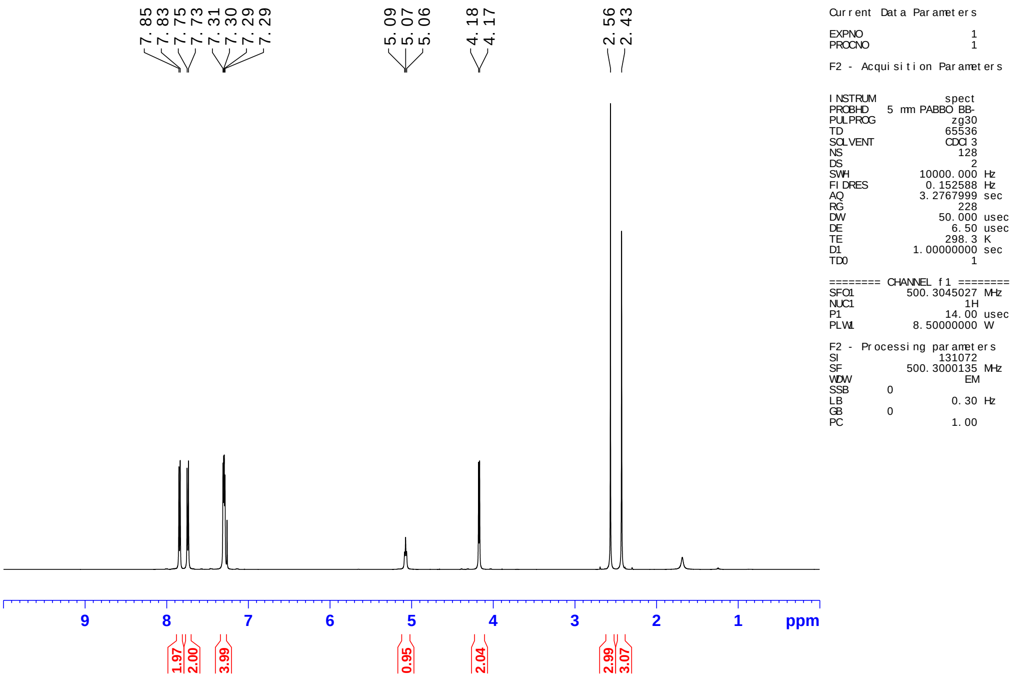

Figure S77: ^{1}H NMR spectrum of **1w**.

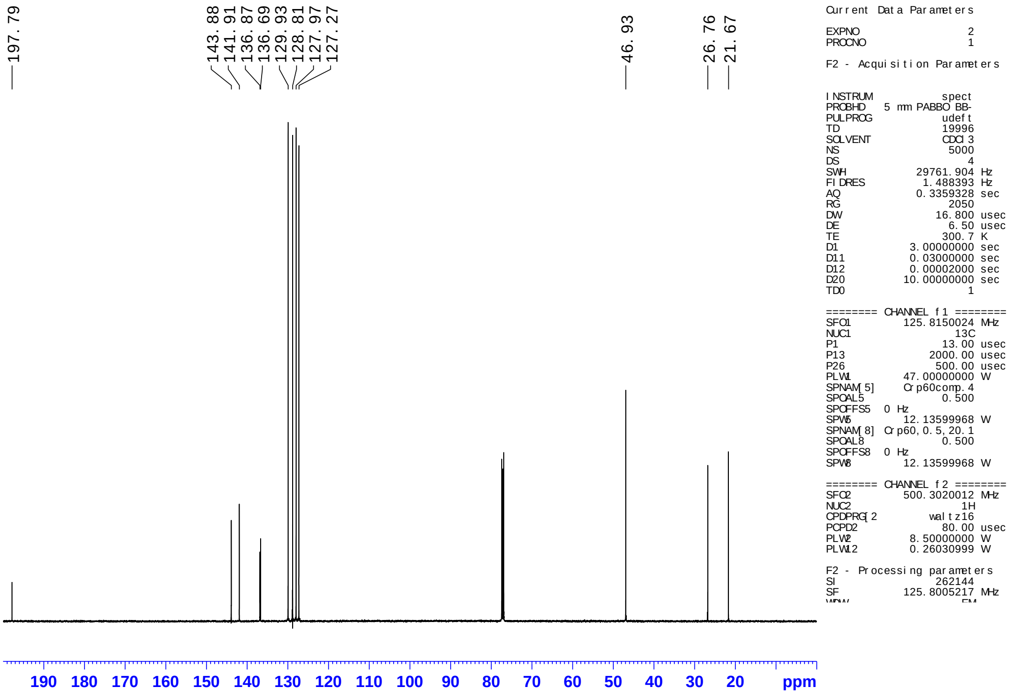

Figure S78: ^{13}C NMR spectrum of **1w**.

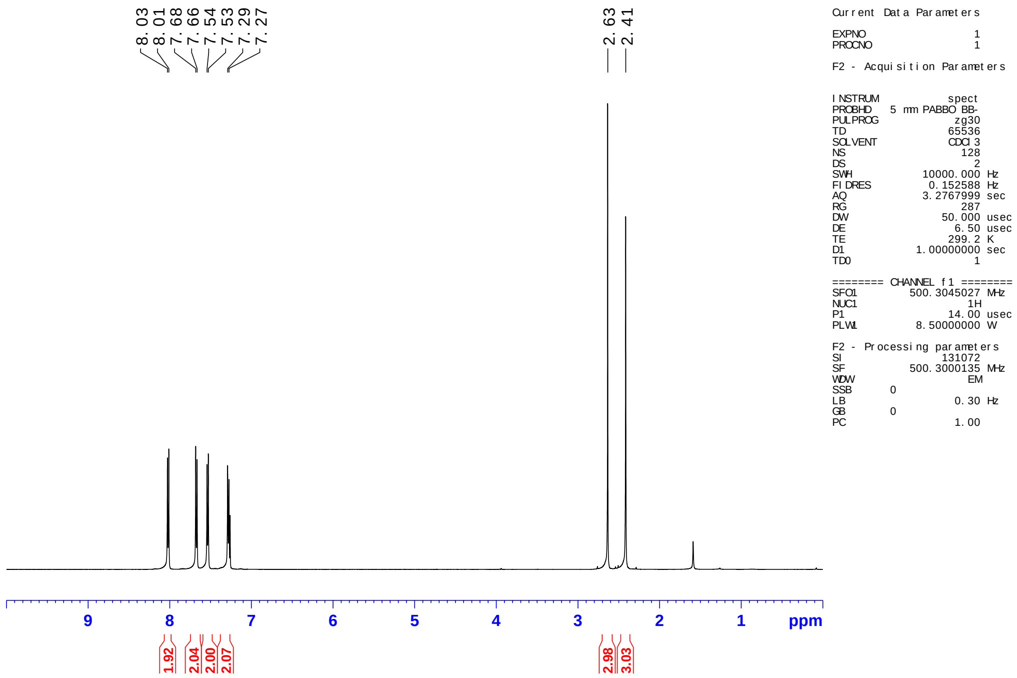

Figure S79: ^{1}H NMR spectrum of **2w**.

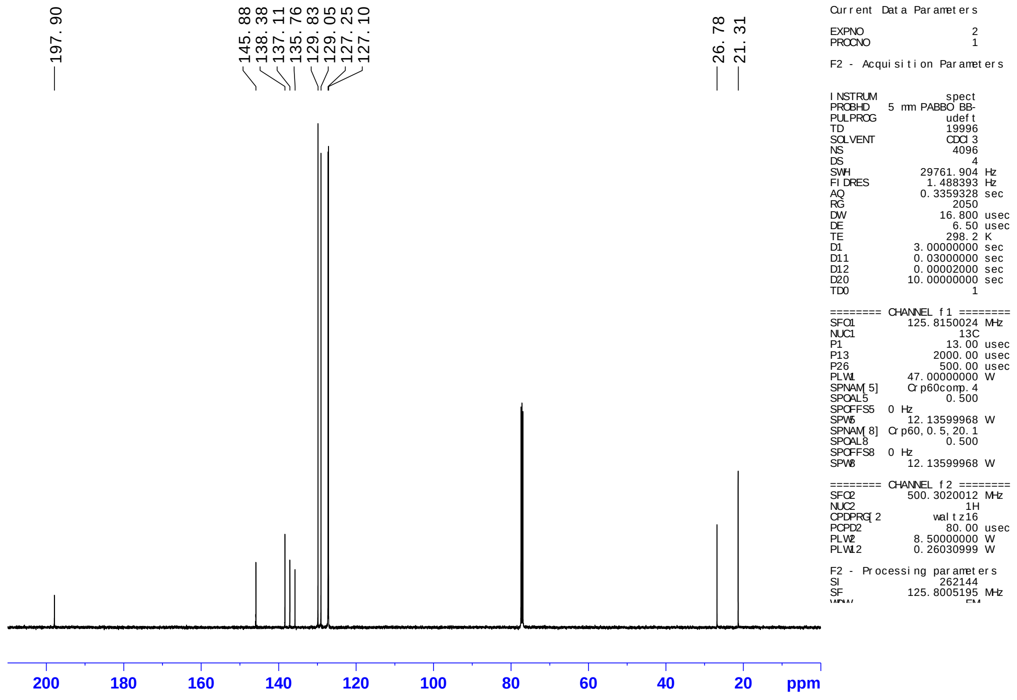

Figure S80: ^{13}C NMR spectrum of **2w**.

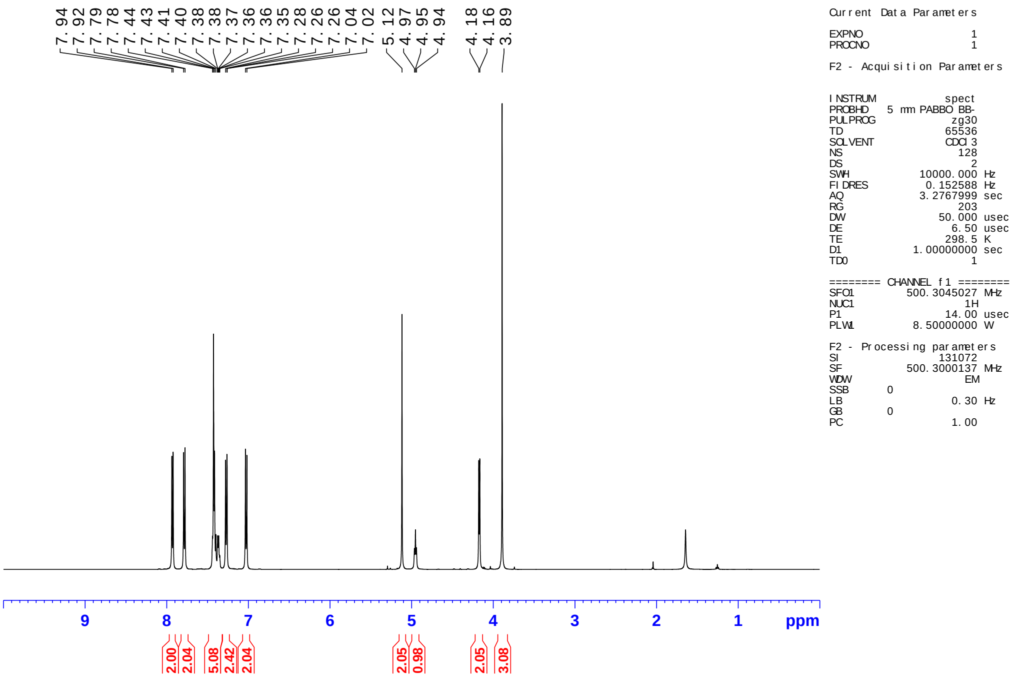

Figure S81: ^{1}H NMR spectrum of **1x**.

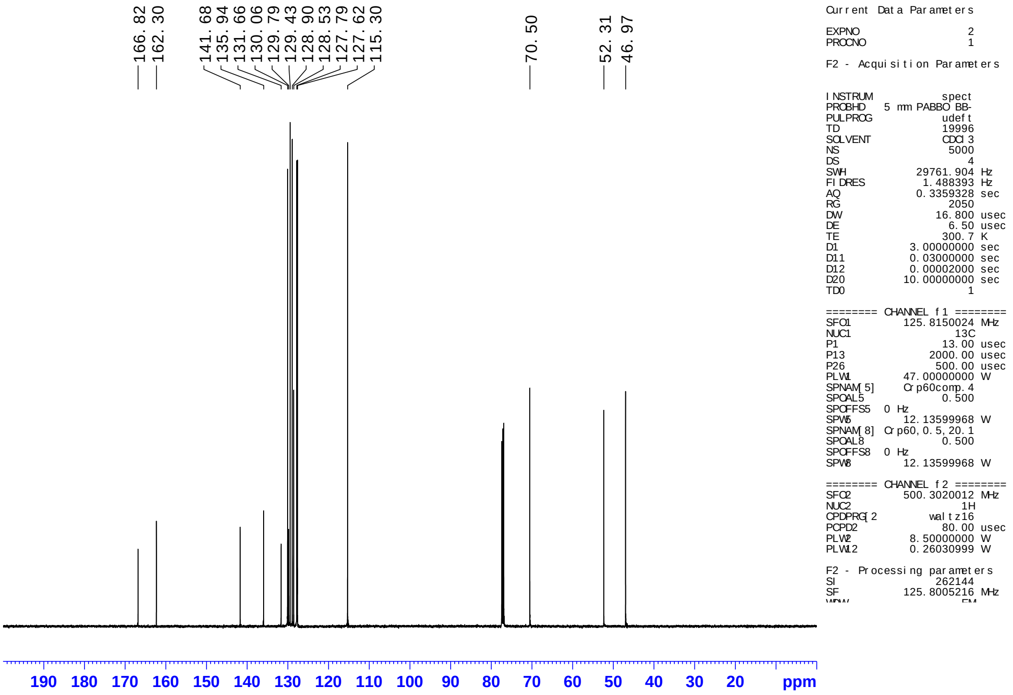

Figure S82: ^{13}C NMR spectrum of **1x**.

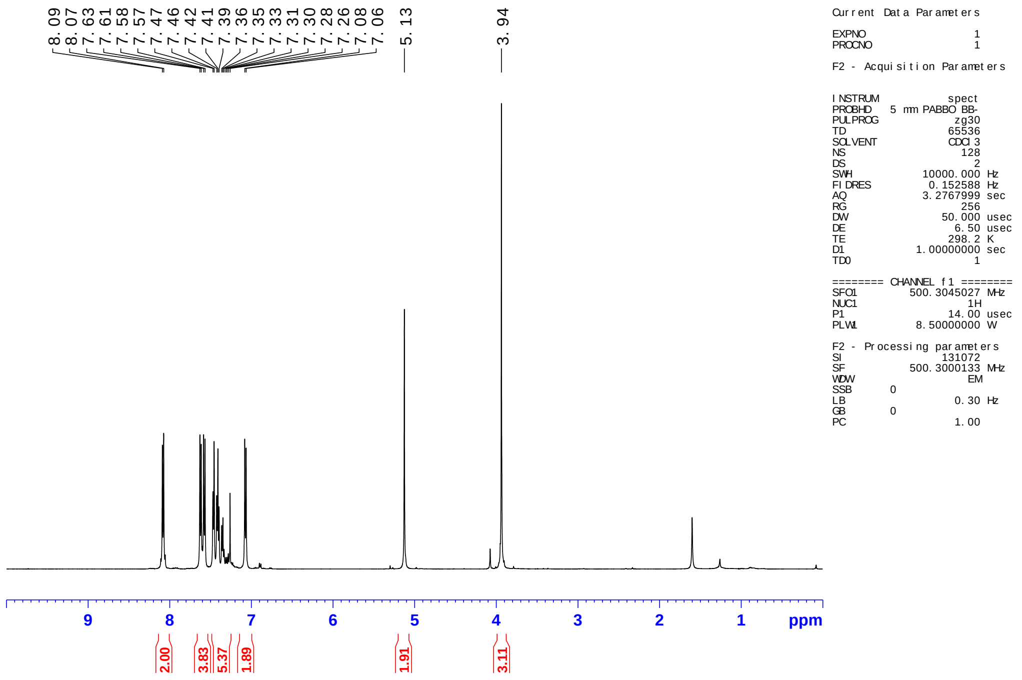

Figure S83: ^{1}H NMR spectrum of **2x**.

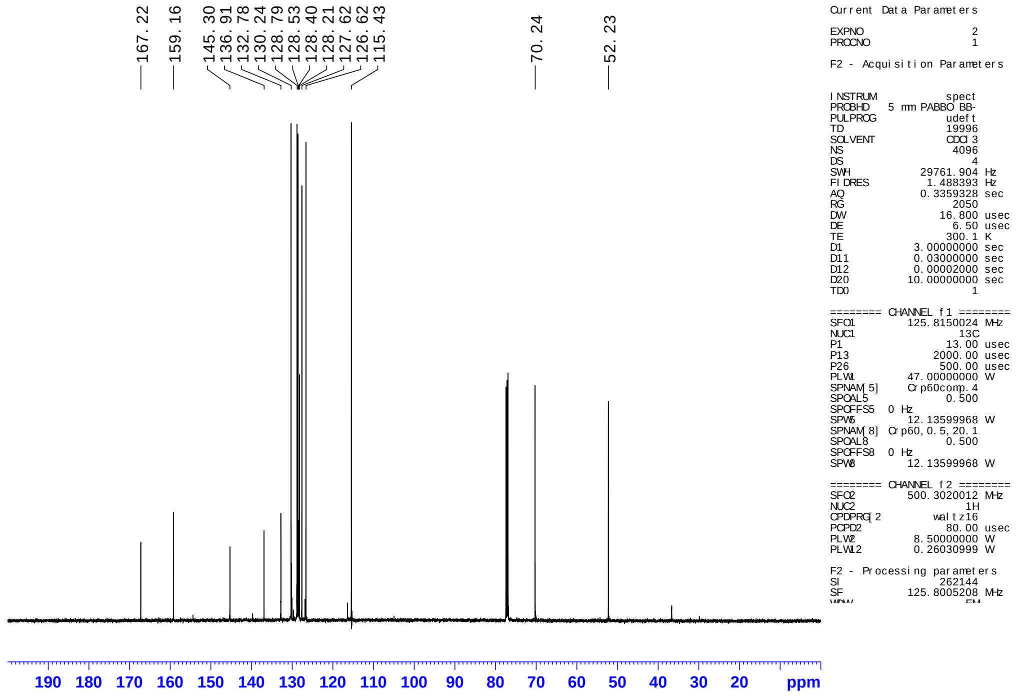

Figure S84: ^{13}C NMR spectrum of **2x**.

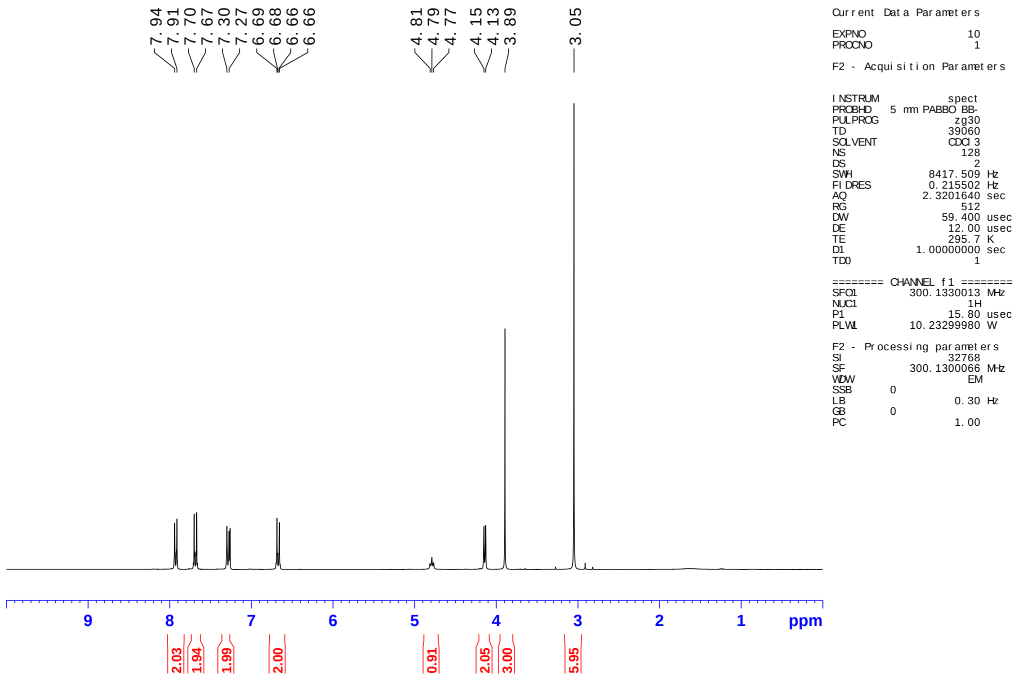

Figure S85: ^{1}H NMR spectrum of **1y**.

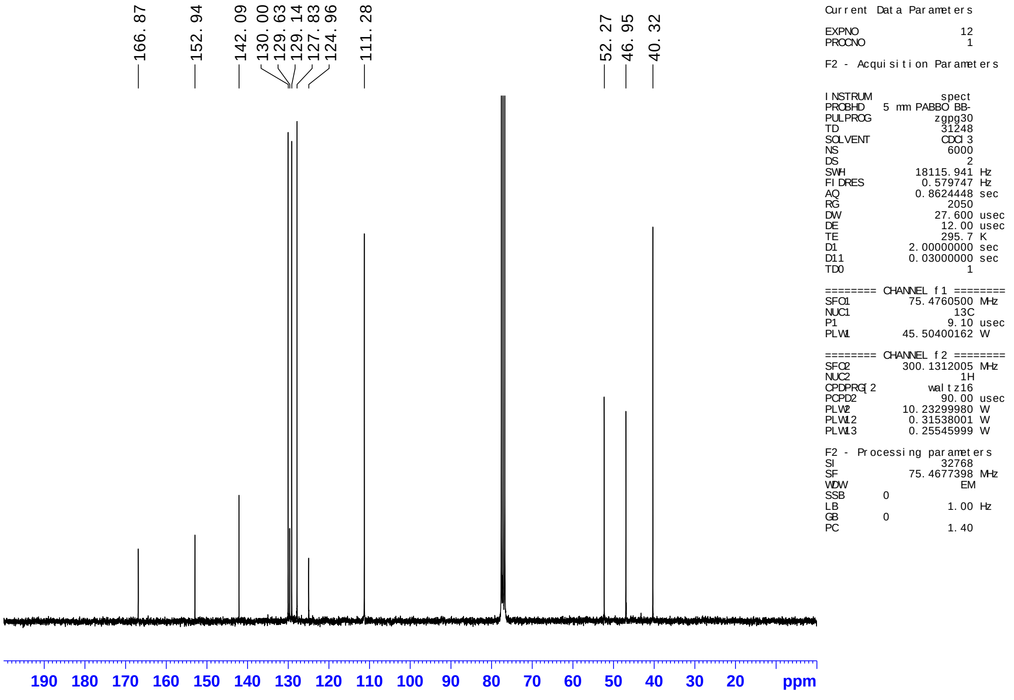

Figure S86: ^{13}C NMR spectrum of **1y**.

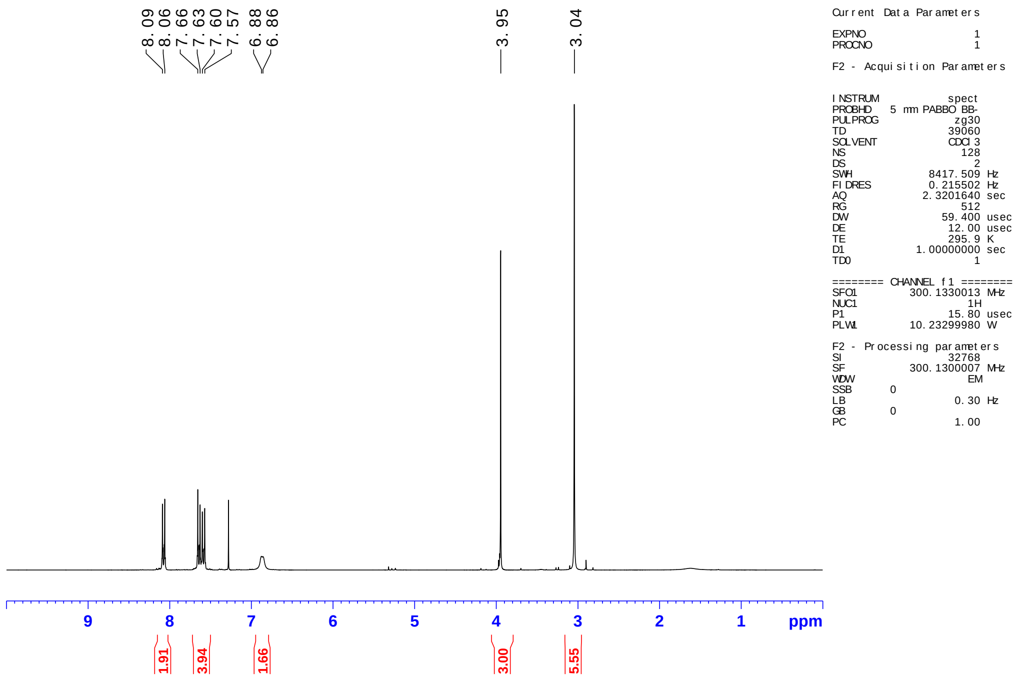

Figure S87: ^{1}H NMR spectrum of **2y**.

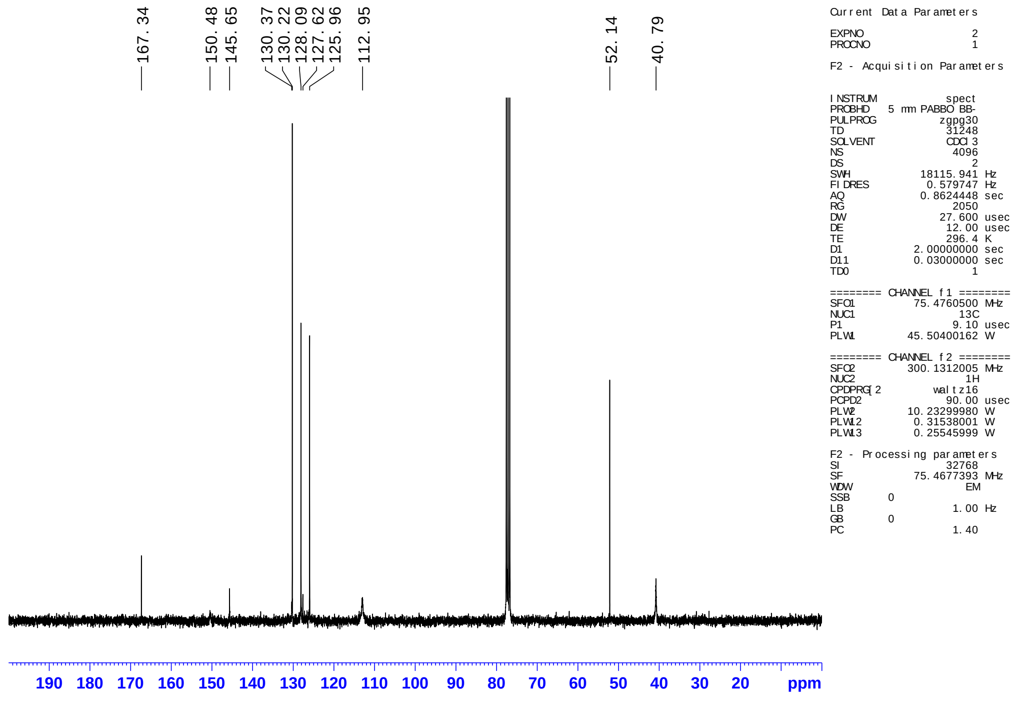

Figure S88: ^{13}C NMR spectrum of **2y**.

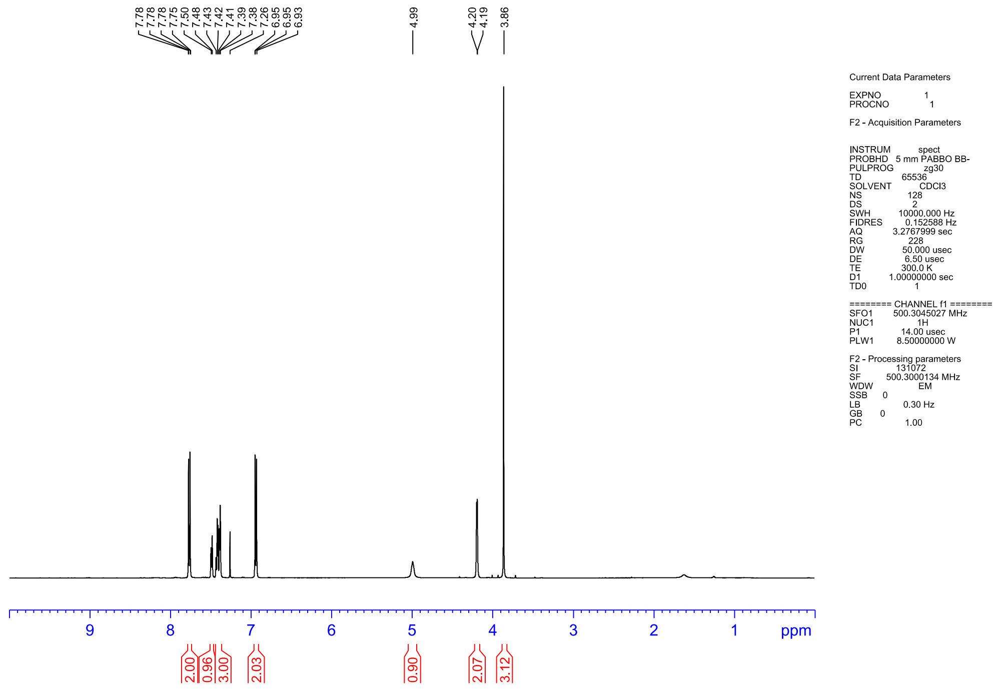

Figure S89: ^{1}H NMR spectrum of **1z**.

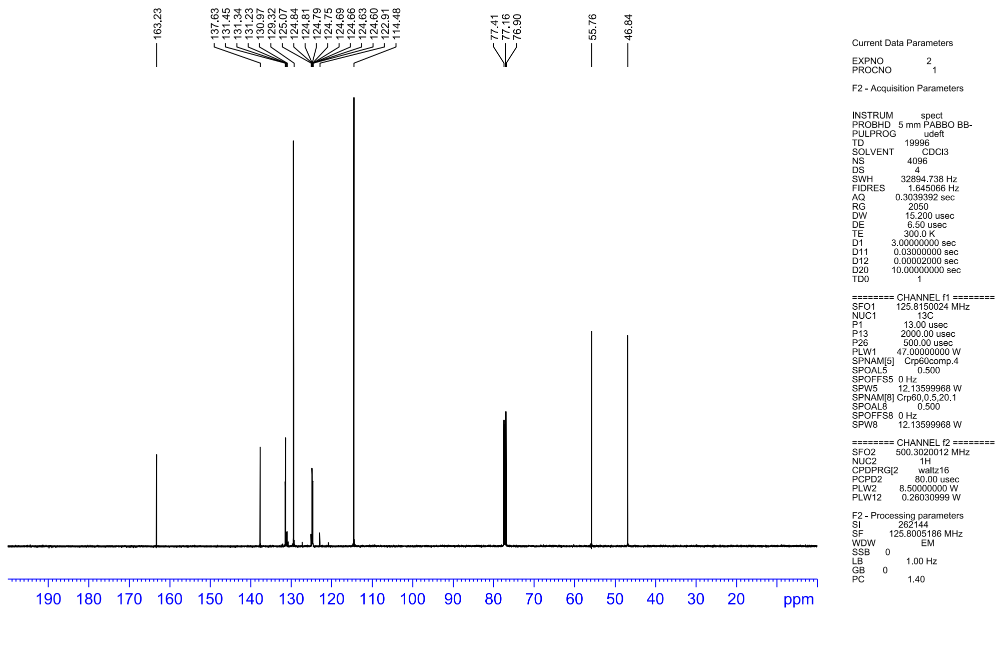

Figure S90: ^{13}C NMR spectrum of **1z**.

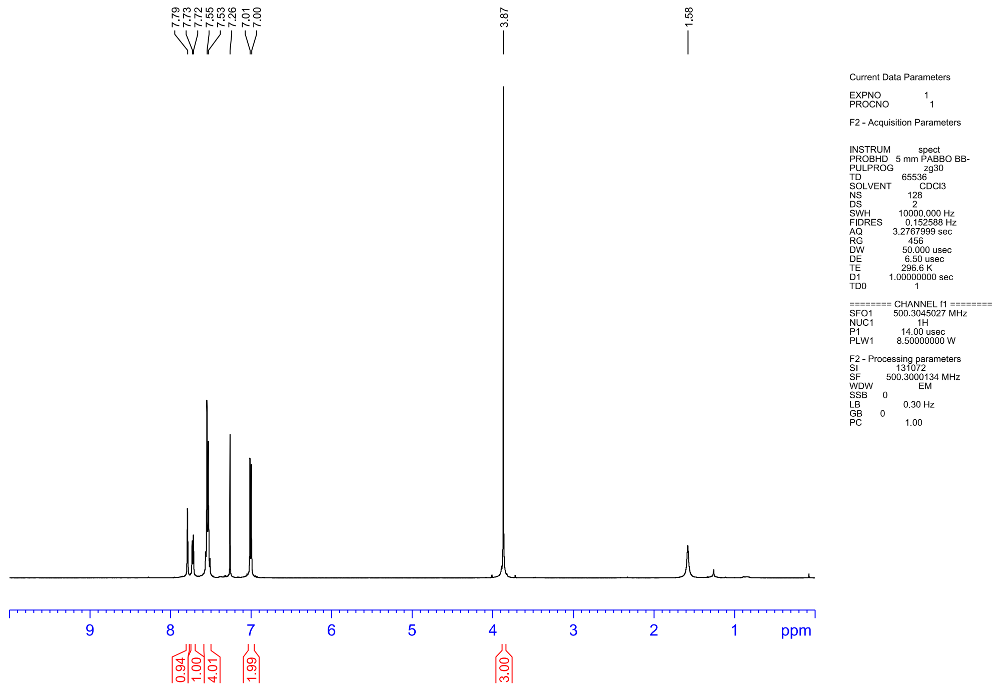

Figure S91: ^{1}H NMR spectrum of **2z**.

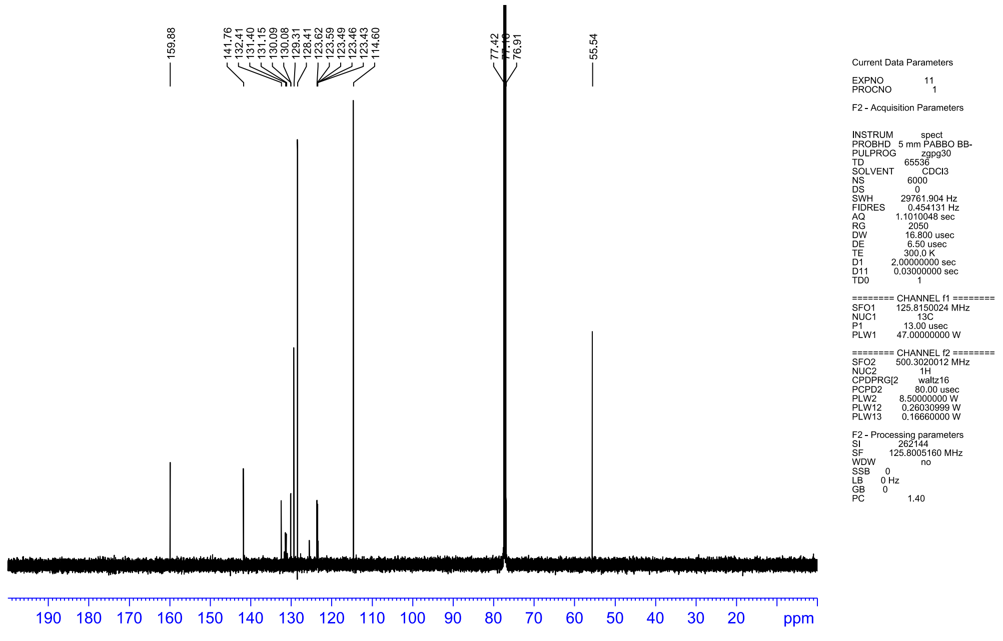

Figure S92: ^{13}C NMR spectrum of **2z**.

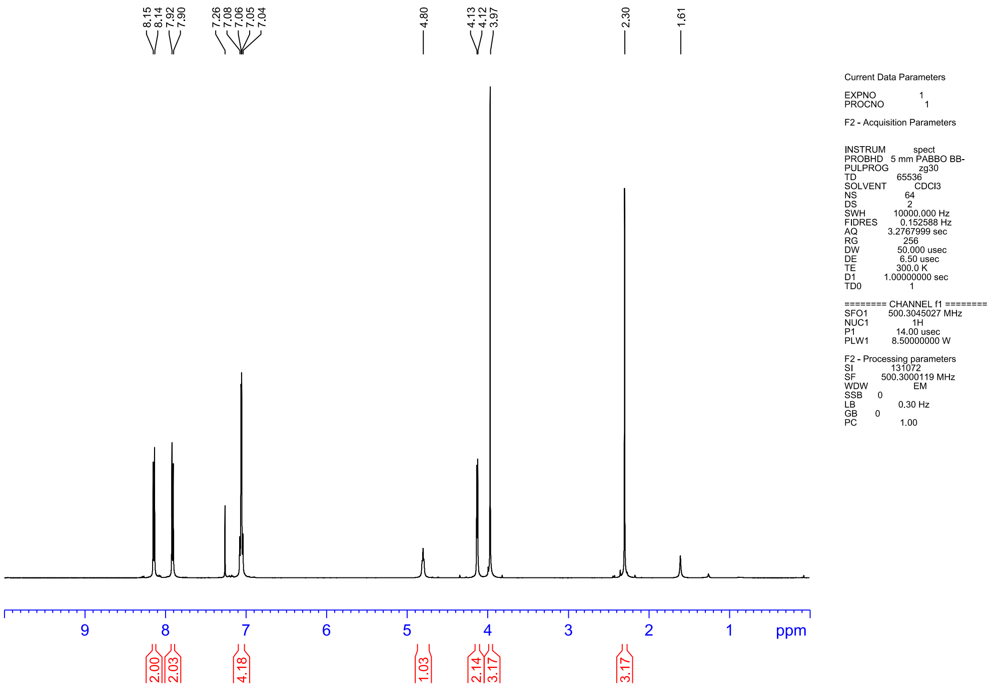

Figure S93: ^{1}H NMR spectrum of **1aa**.

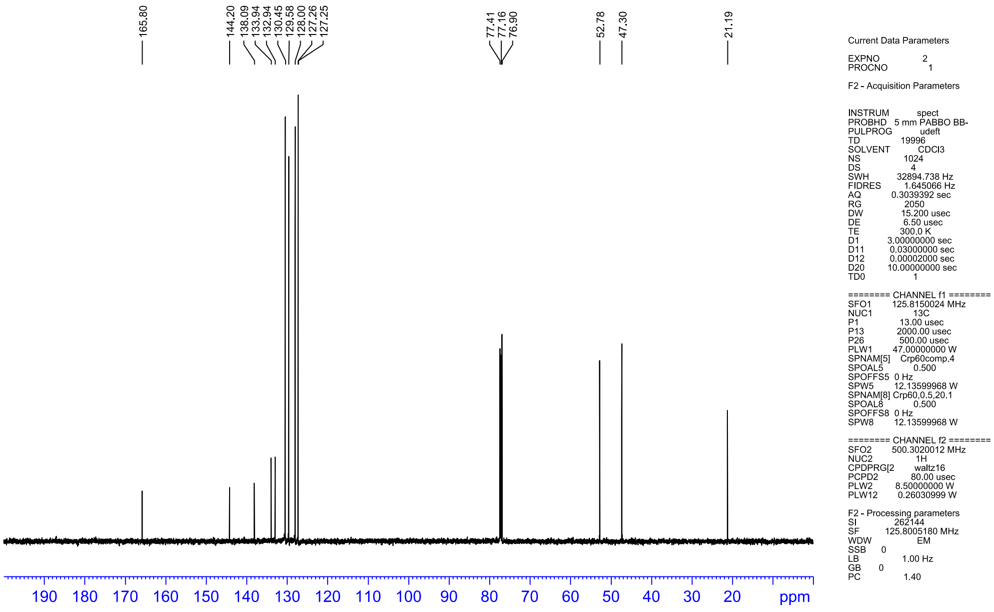

Figure S94: ^{13}C NMR spectrum of **1aa**.

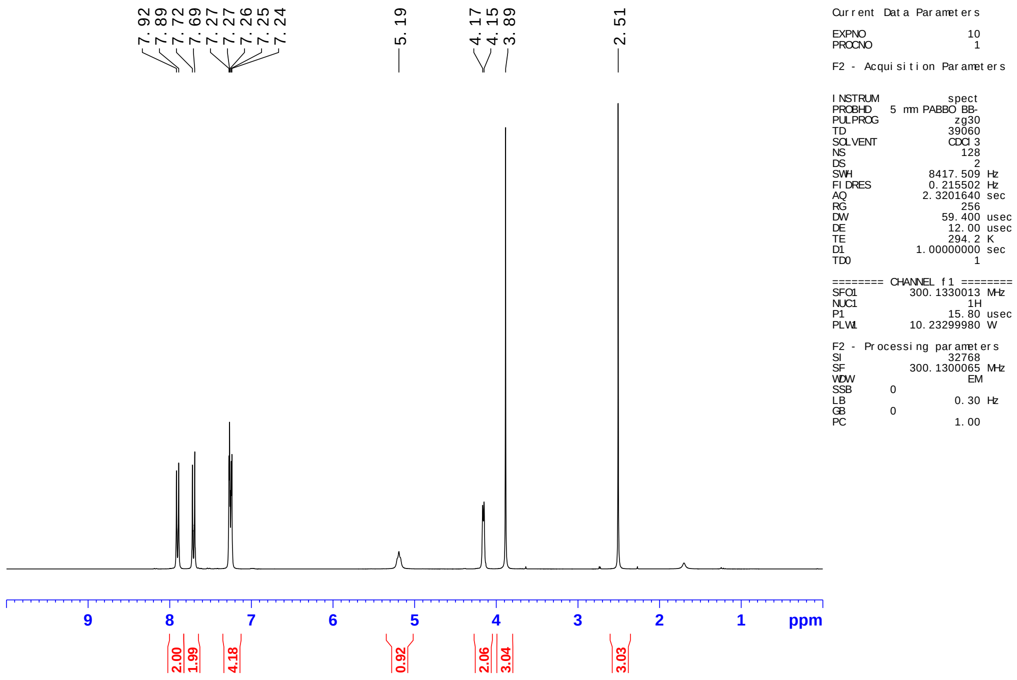

Figure S95: ^{1}H NMR spectrum of **1ab**.

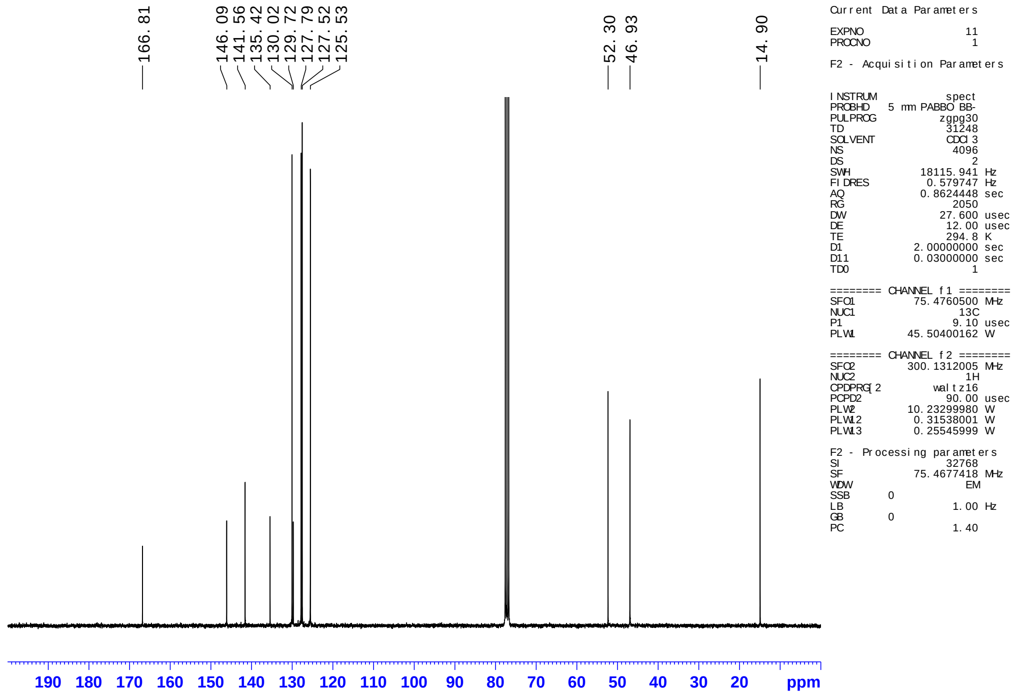

Figure S96: ^{13}C NMR spectrum of **1ab**.

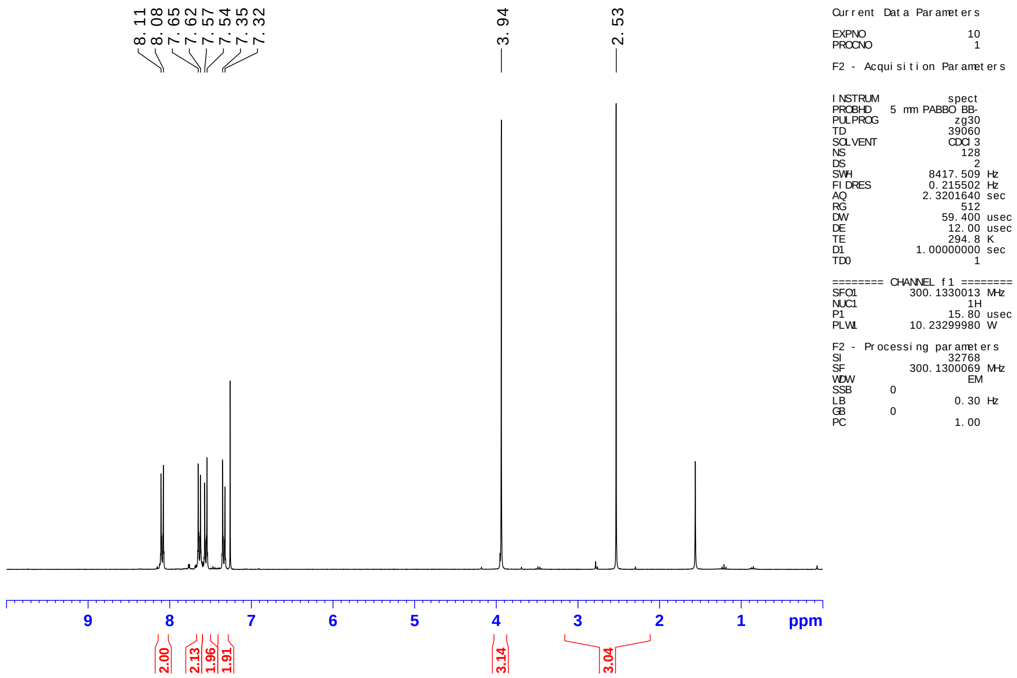

Figure S97: ^{1}H NMR spectrum of **2ab**.

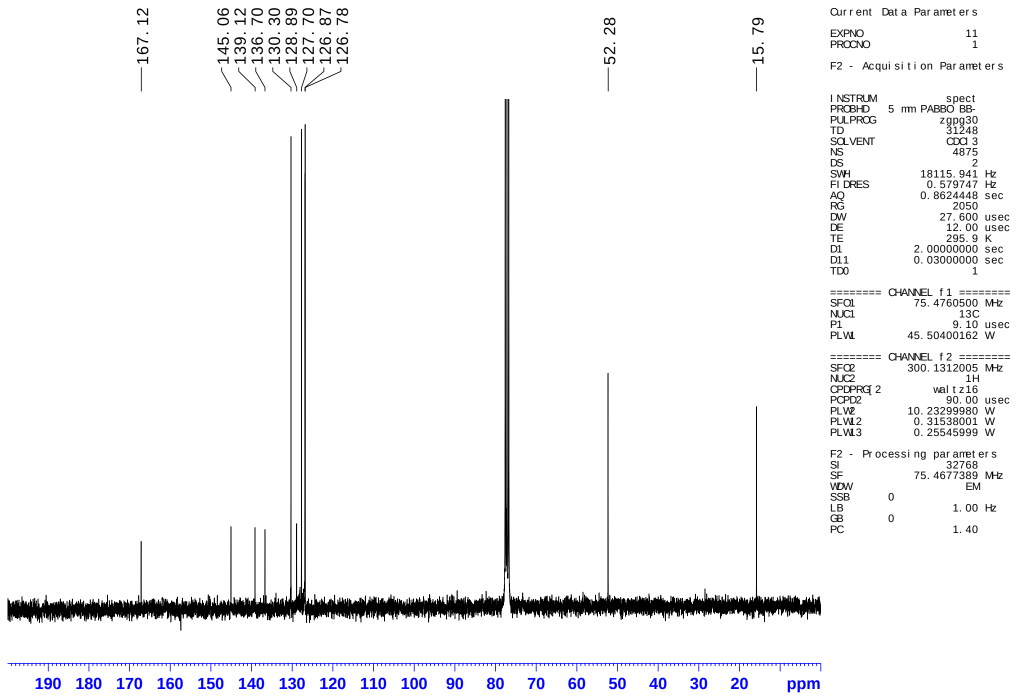

Figure S98: ^{13}C NMR spectrum of **2ab**.

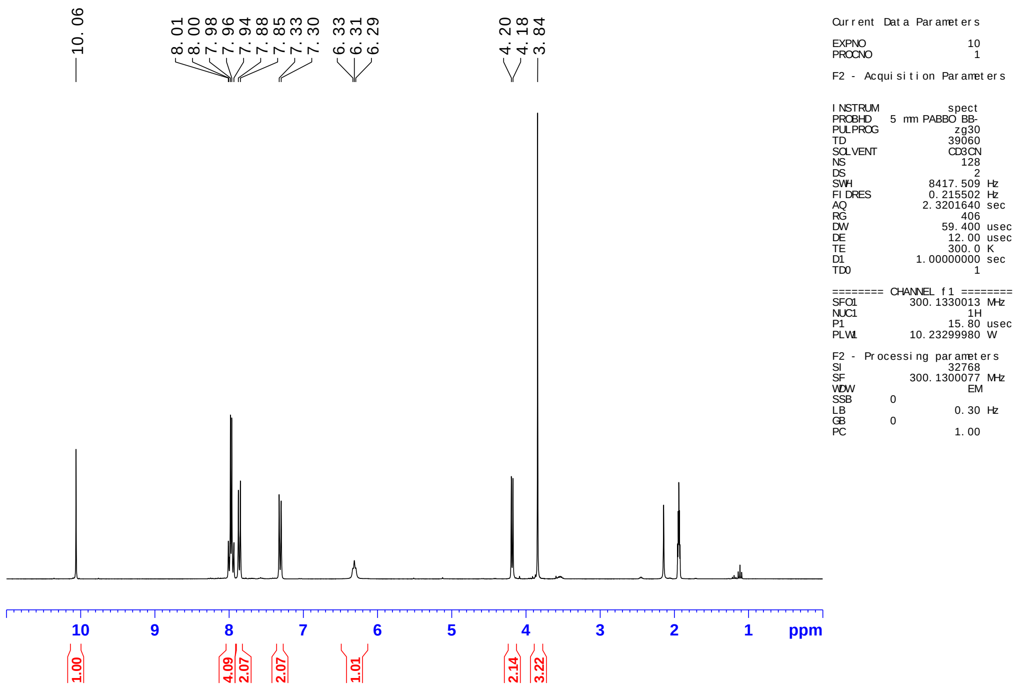

Figure S99: ^{1}H NMR spectrum of **1ac**.

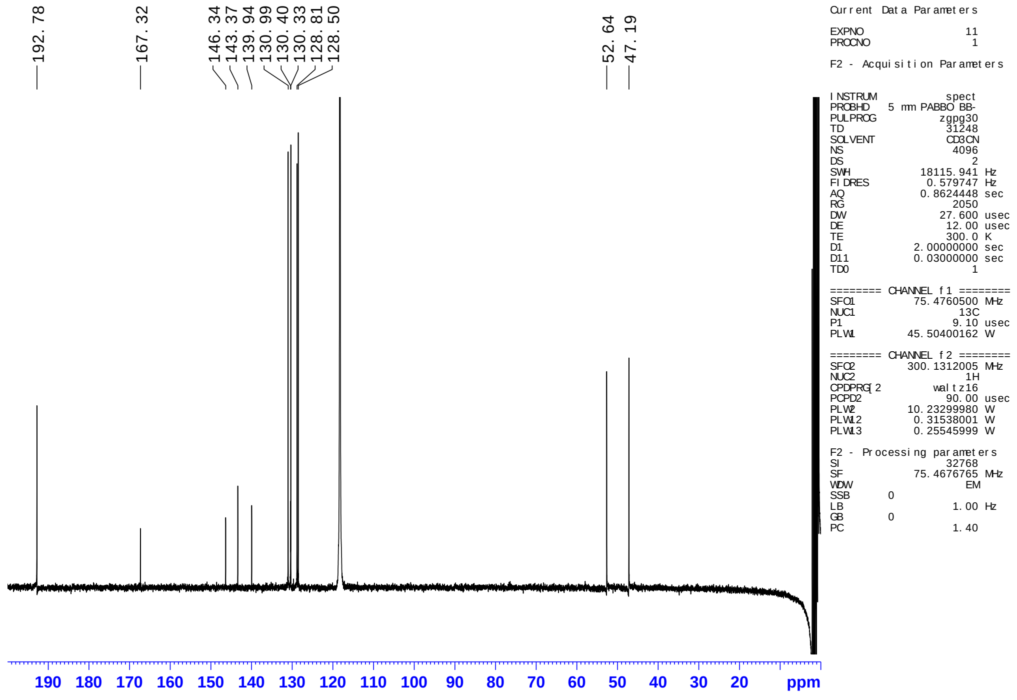

Figure S100: ^{13}C NMR spectrum of **1ac**.

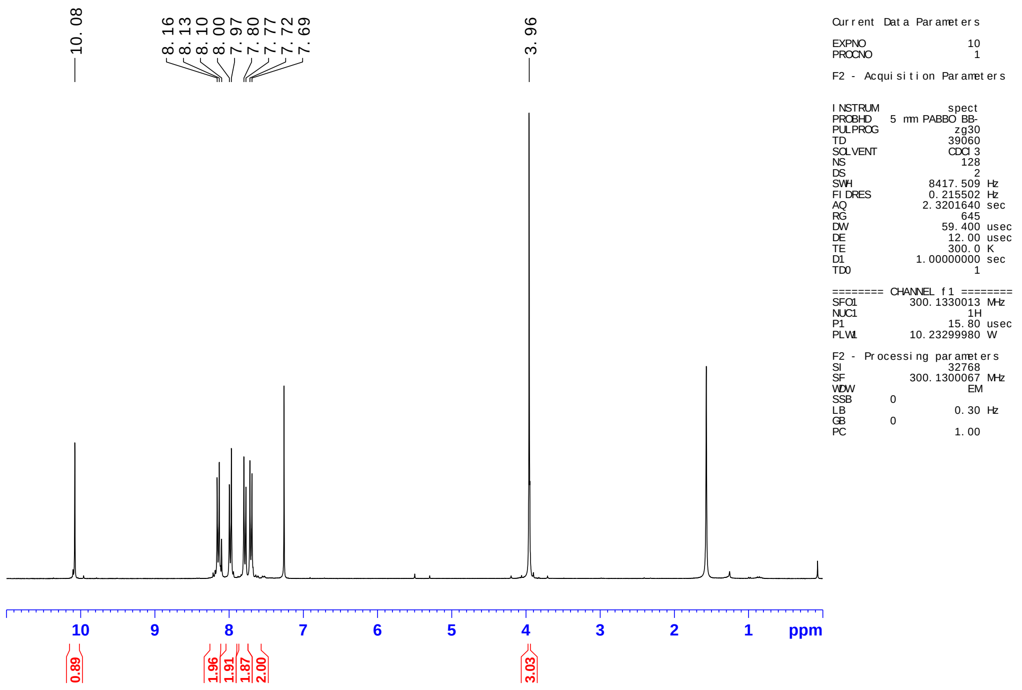

Figure S101: ^{1}H NMR spectrum of **2ac**.

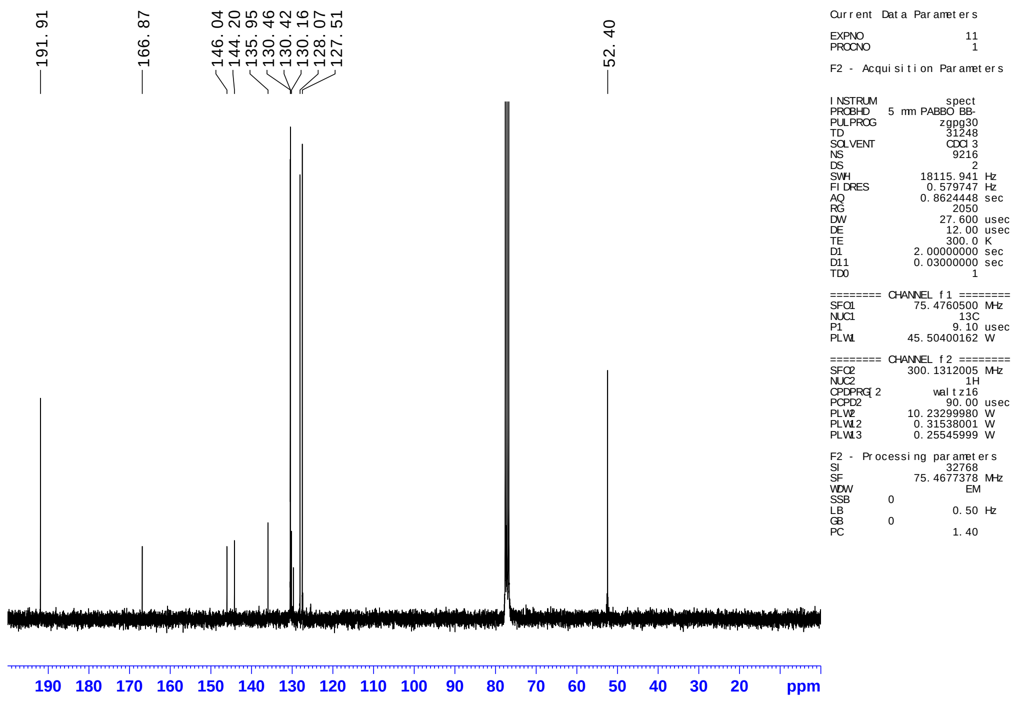

Figure S102: ^{13}C NMR spectrum of **2ac**.

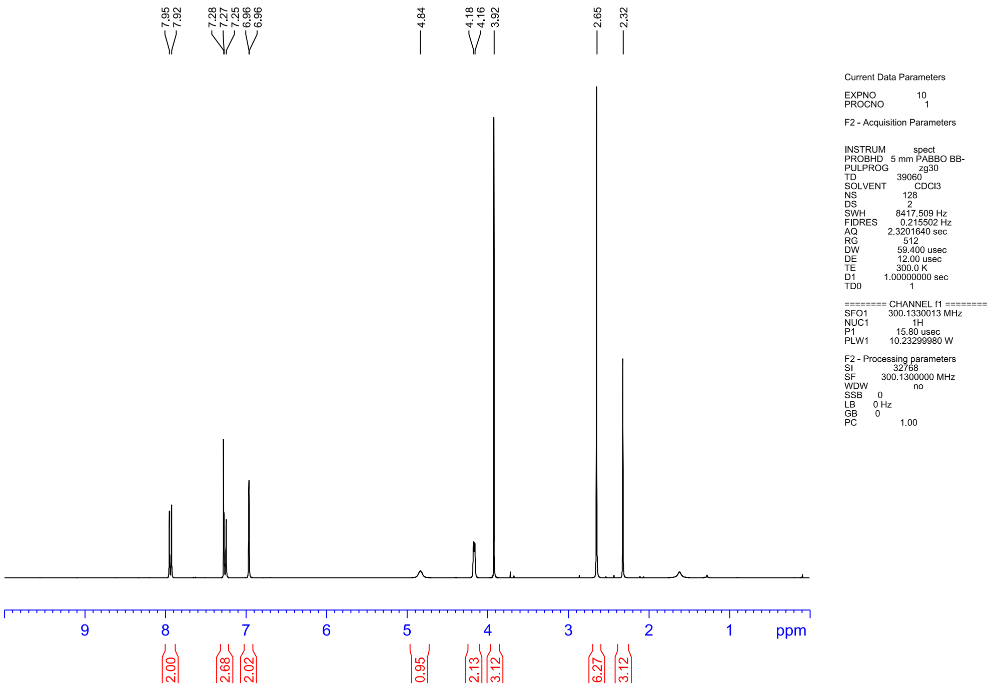

Figure S103: ^{1}H NMR spectrum of **1ad**.

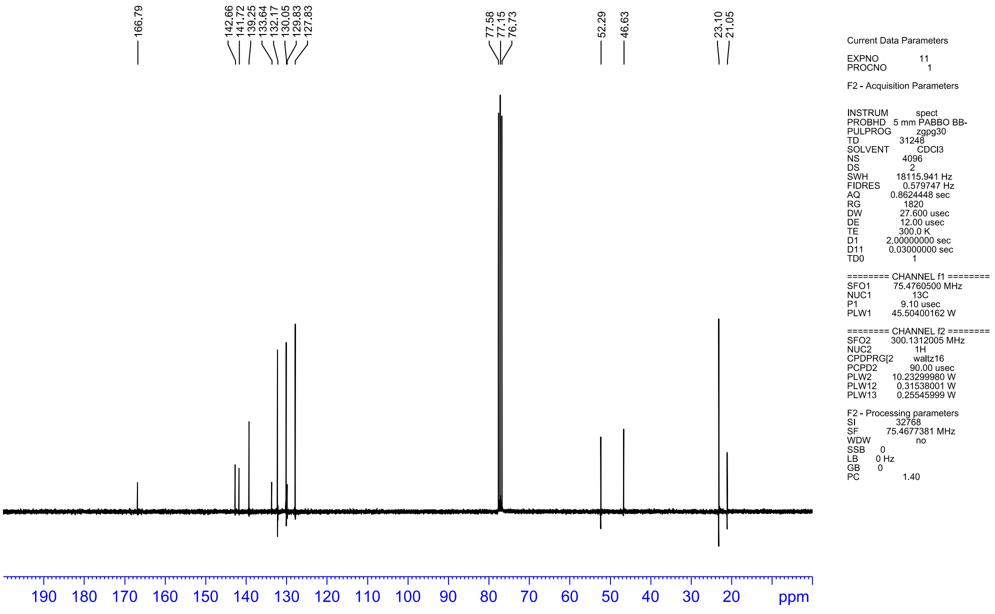

Figure S104: ^{13}C NMR spectrum of **1ad**.

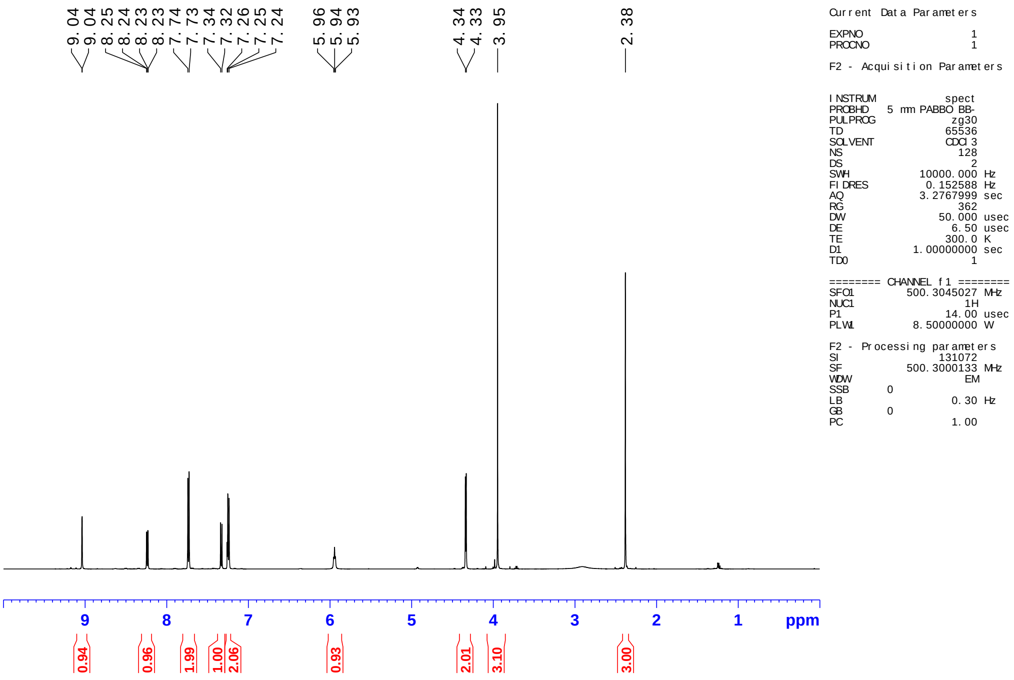

Figure S105: ^{1}H NMR spectrum of **1ae**.

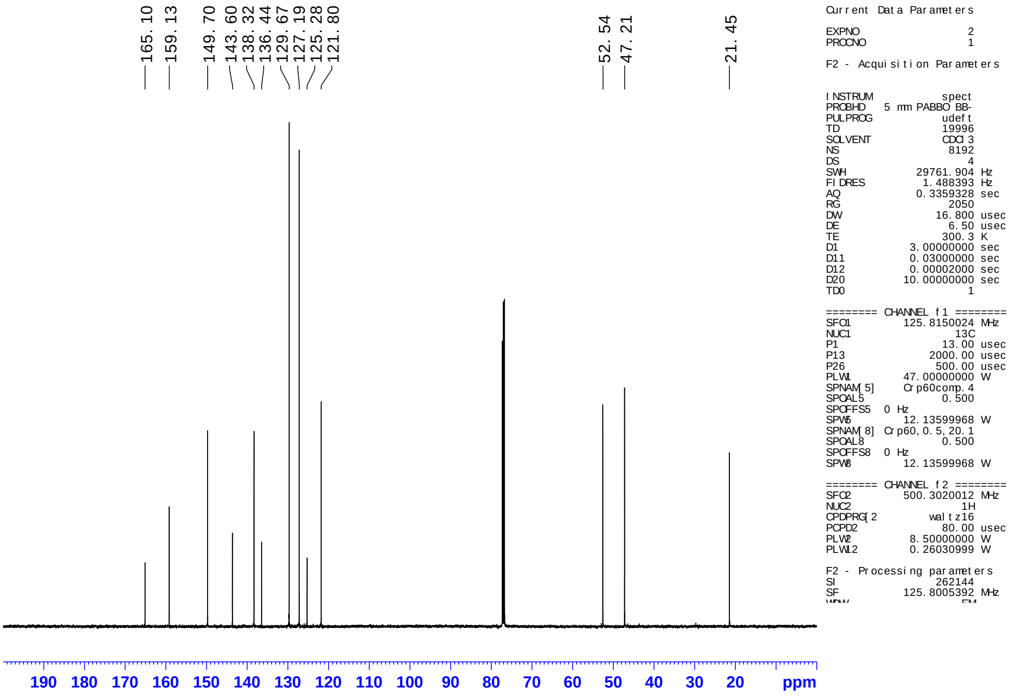

Figure S106: ^{13}C NMR spectrum of **1ae**.

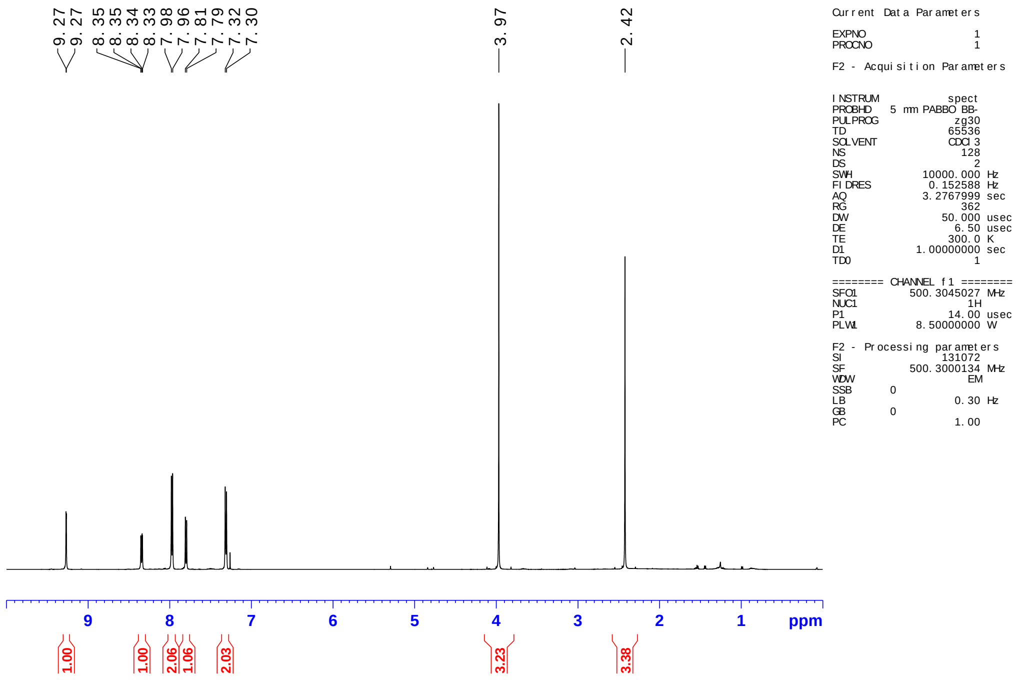

Figure S107: ^{1}H NMR spectrum of **2ae**.

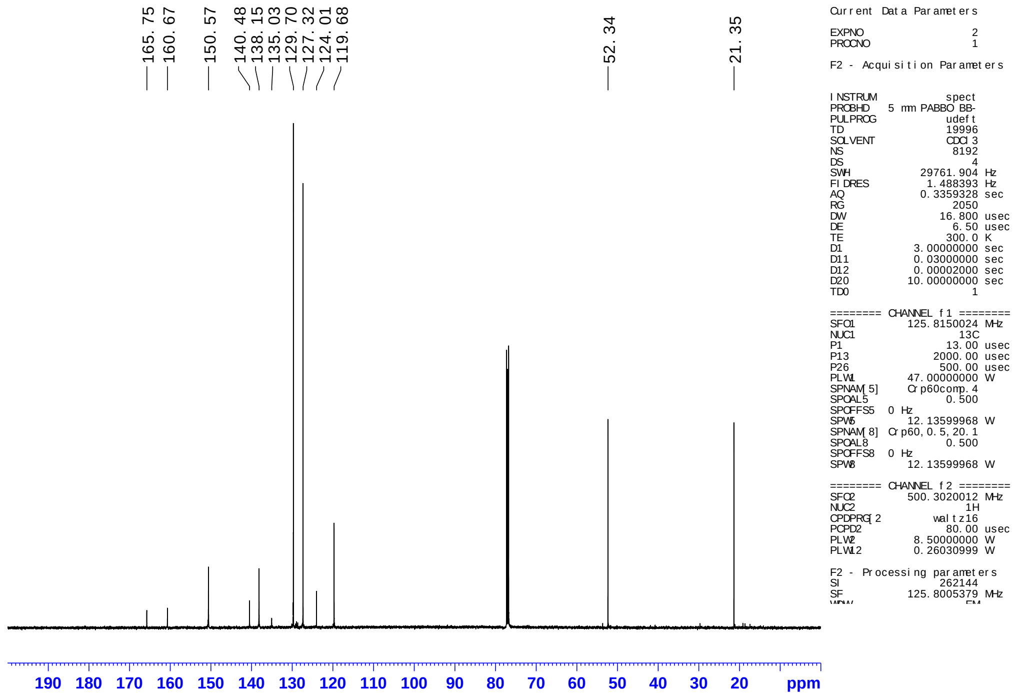

Figure S108: ^{13}C NMR spectrum of **2ae**.

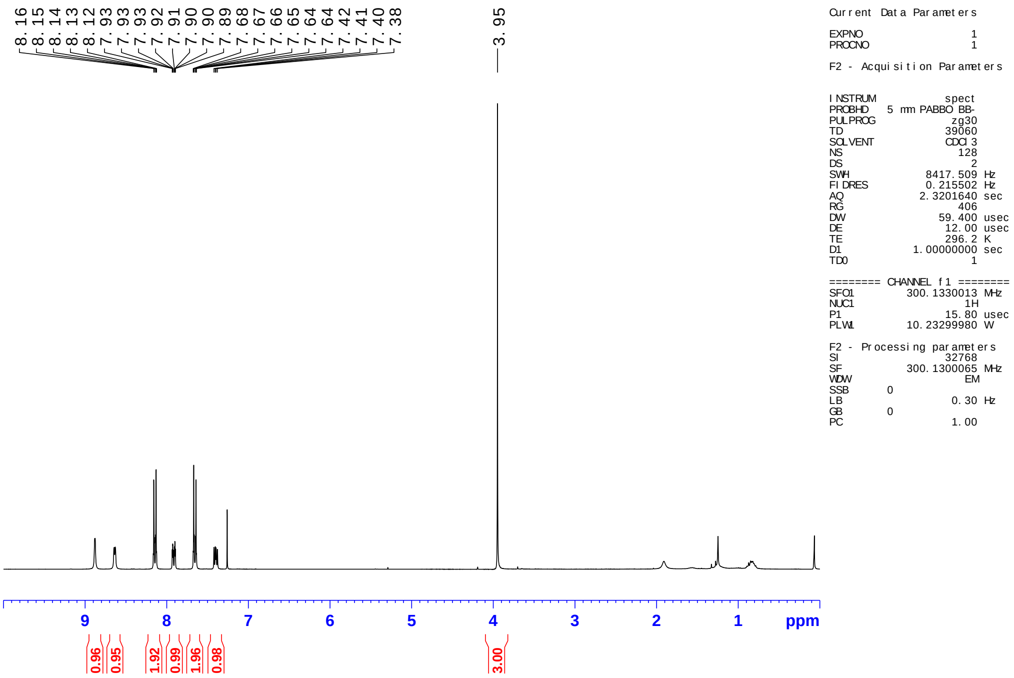

Figure S109: ^{1}H NMR spectrum of **1af**.

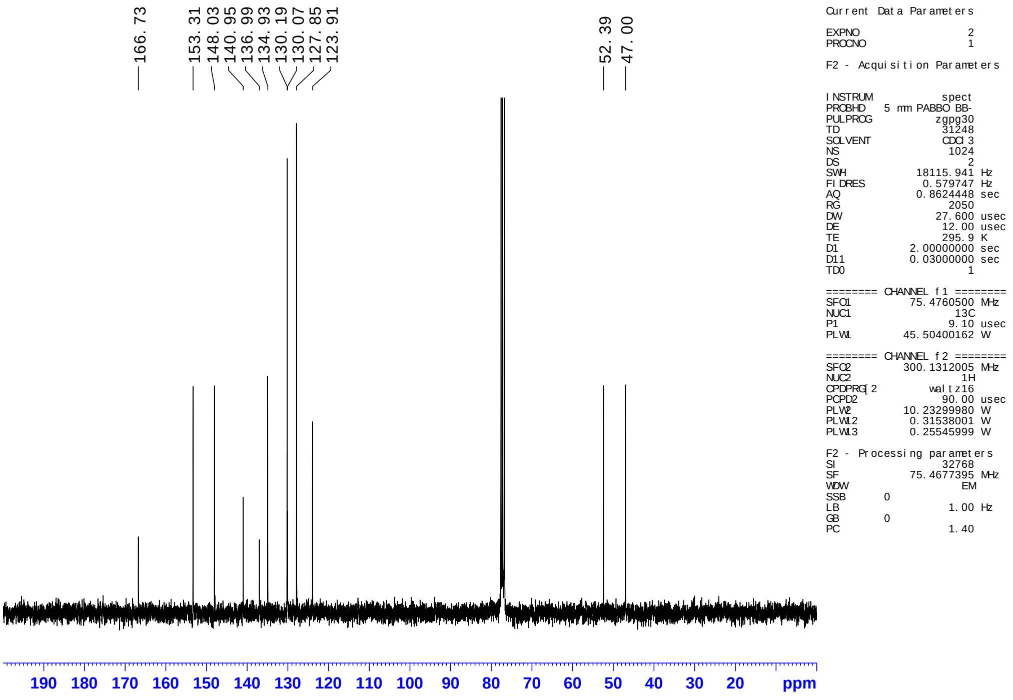

Figure S110: ^{13}C NMR spectrum of **1af**.

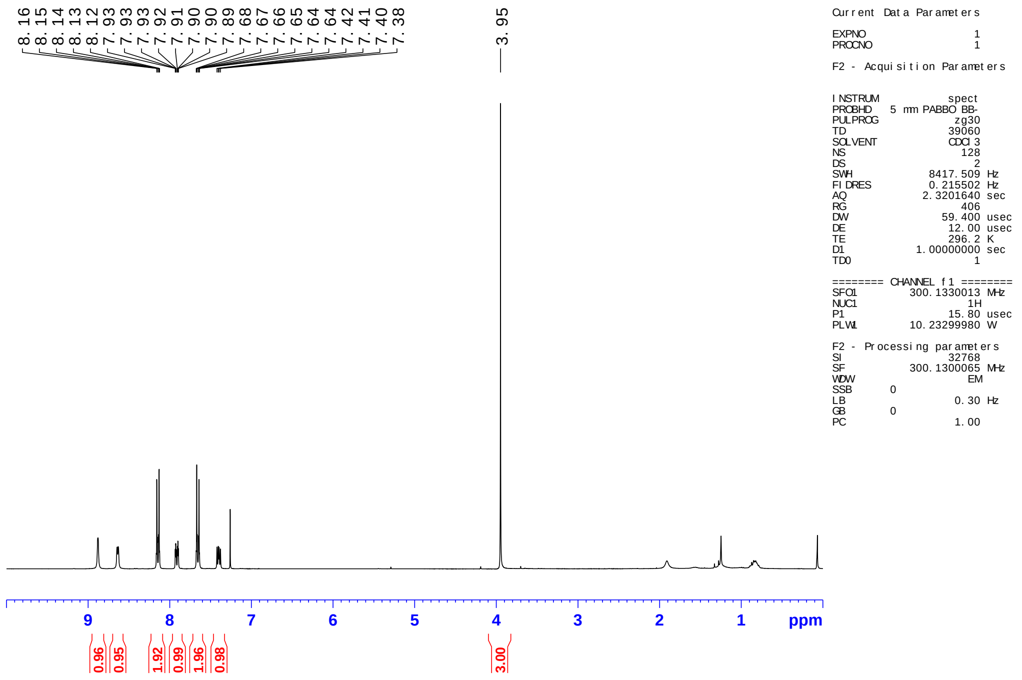

Figure S111: ^{1}H NMR spectrum of **2af**.

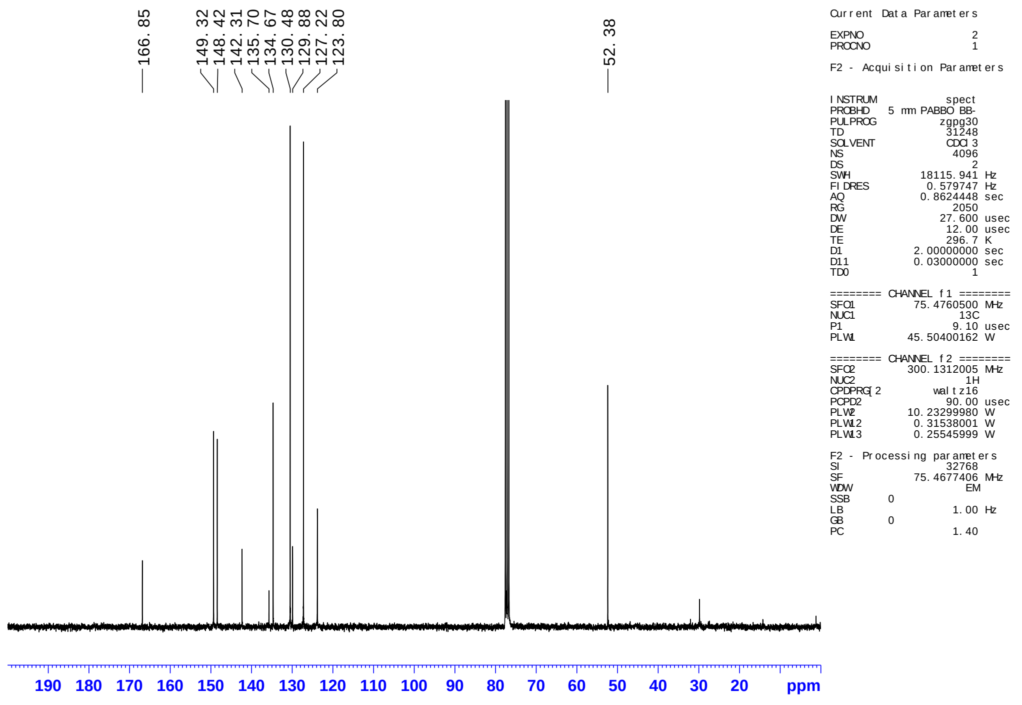

Figure S112: ^{13}C NMR spectrum of **2af**.

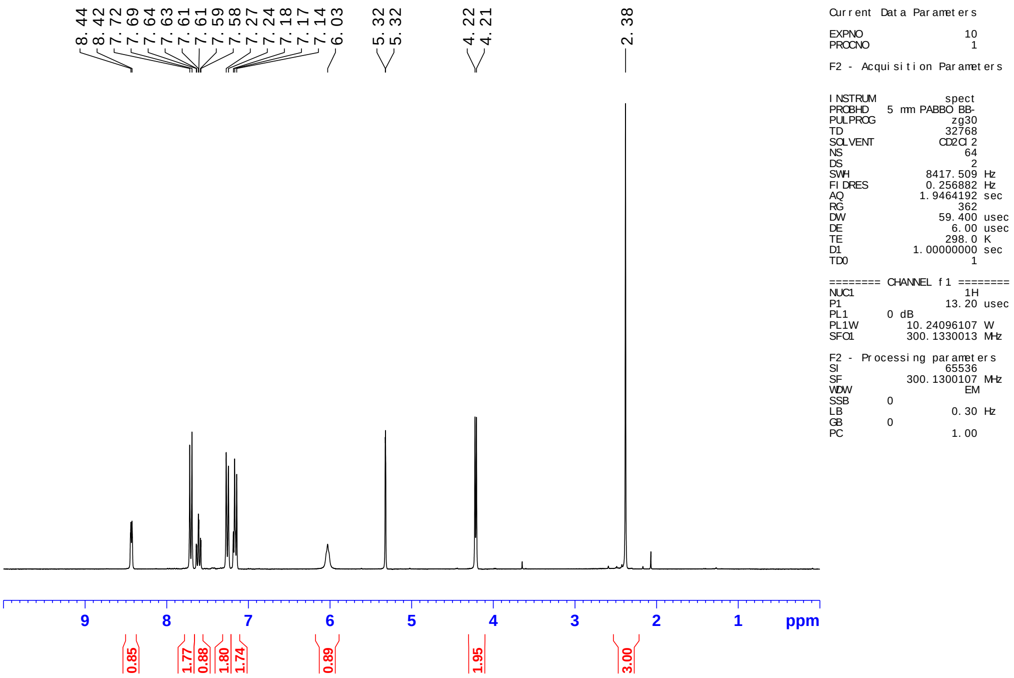

Figure S113: ^{1}H NMR spectrum of **1ag**.

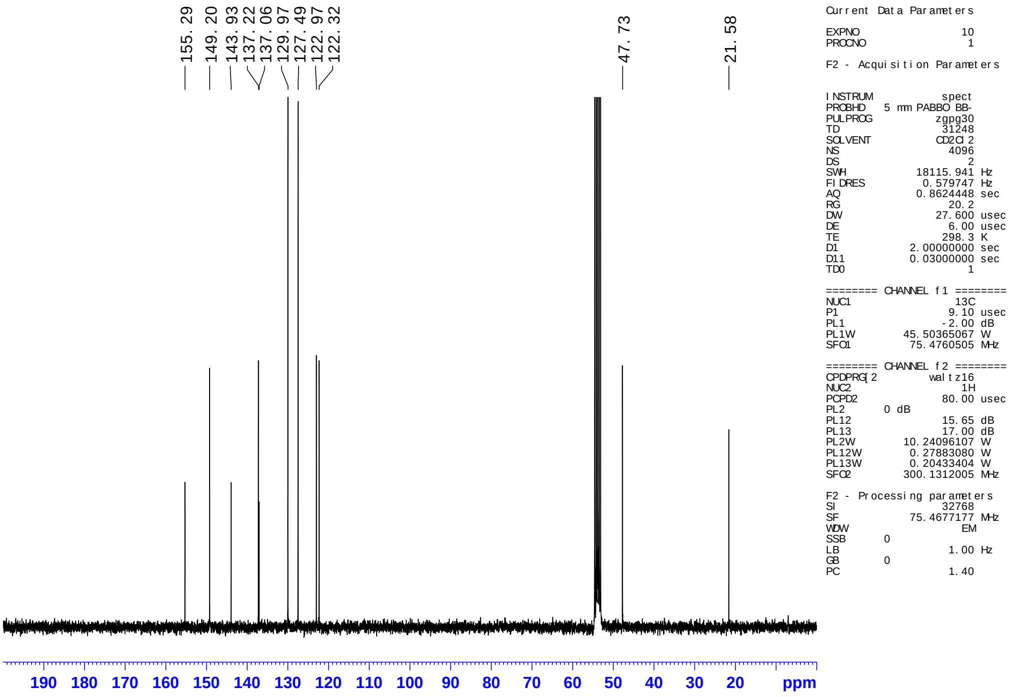

Figure S114: ^{13}C NMR spectrum of **1ag**.

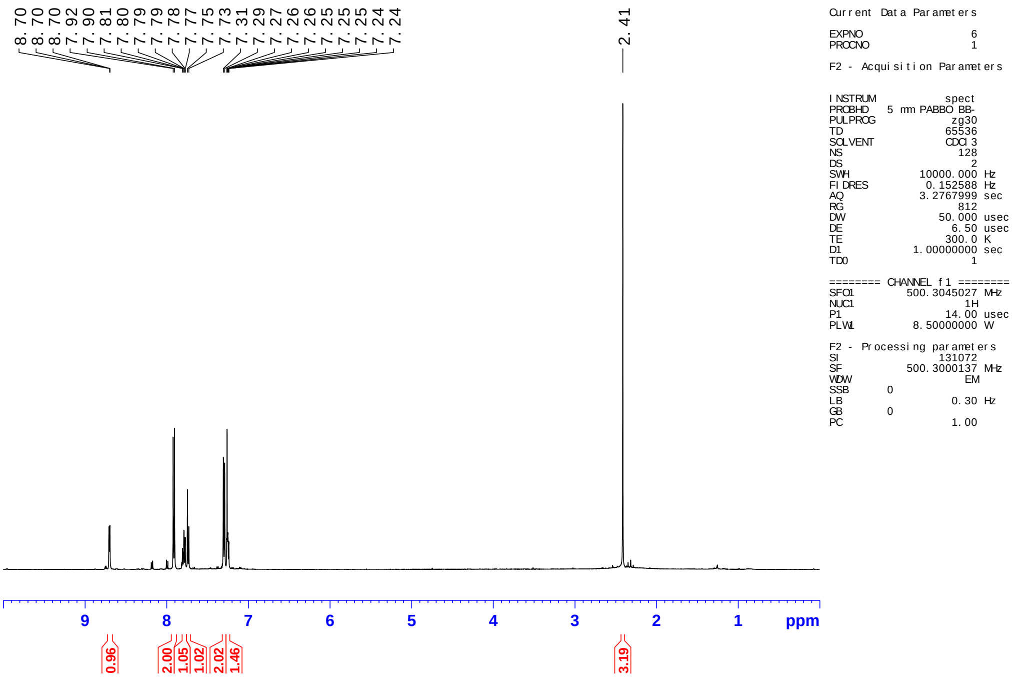

Figure S115: ^{1}H NMR spectrum of **2ag**.

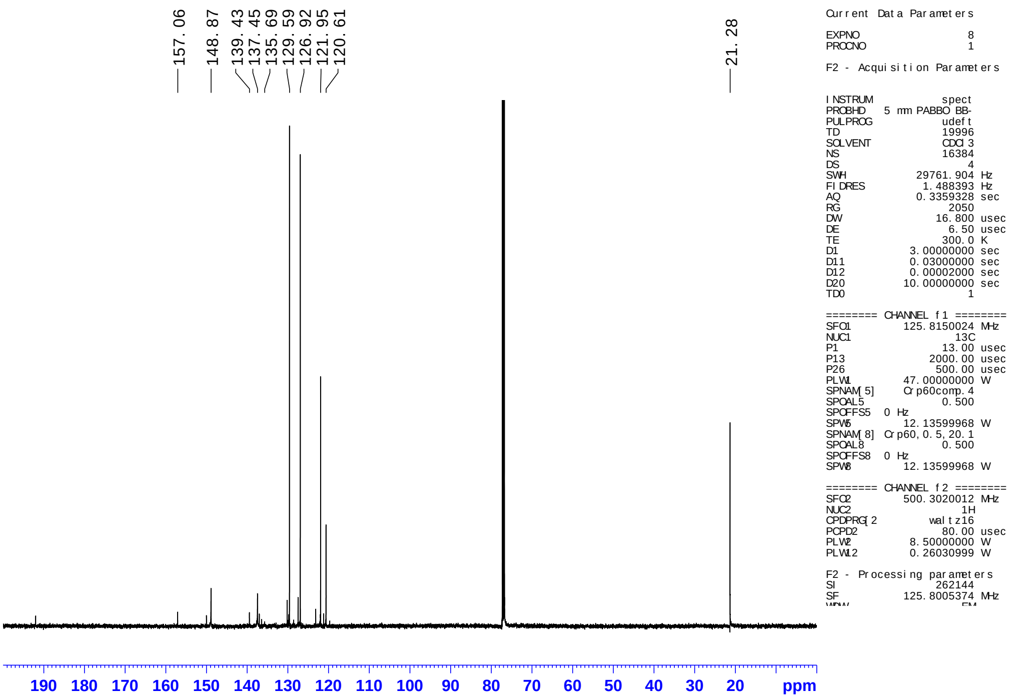

Figure S116: ^{13}C NMR spectrum of **2ag**.

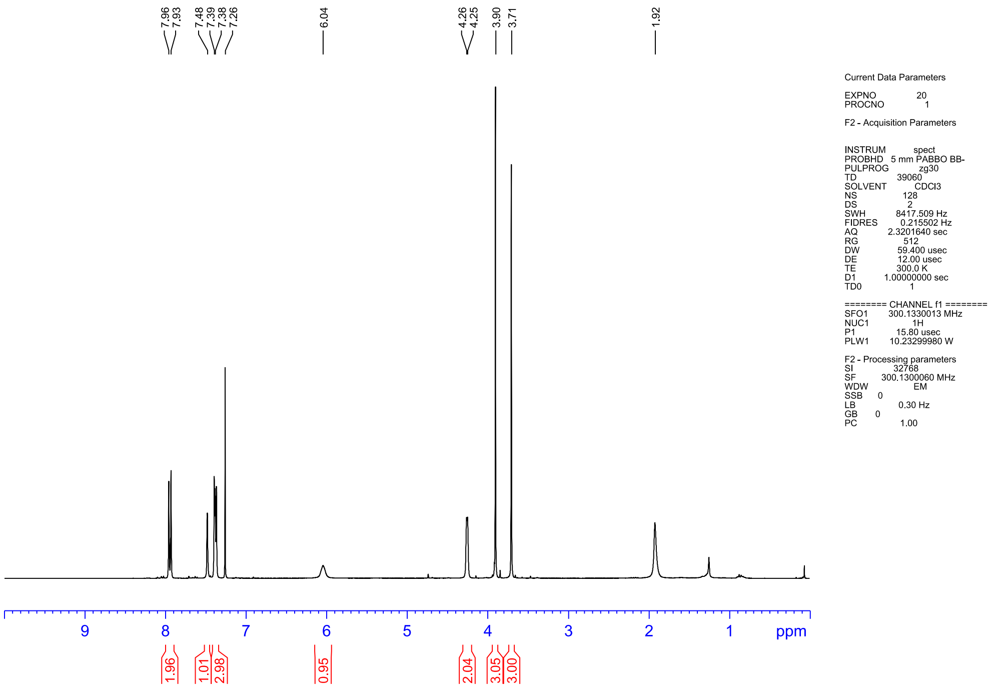

Figure S117: ^{1}H NMR spectrum of **1ah**.

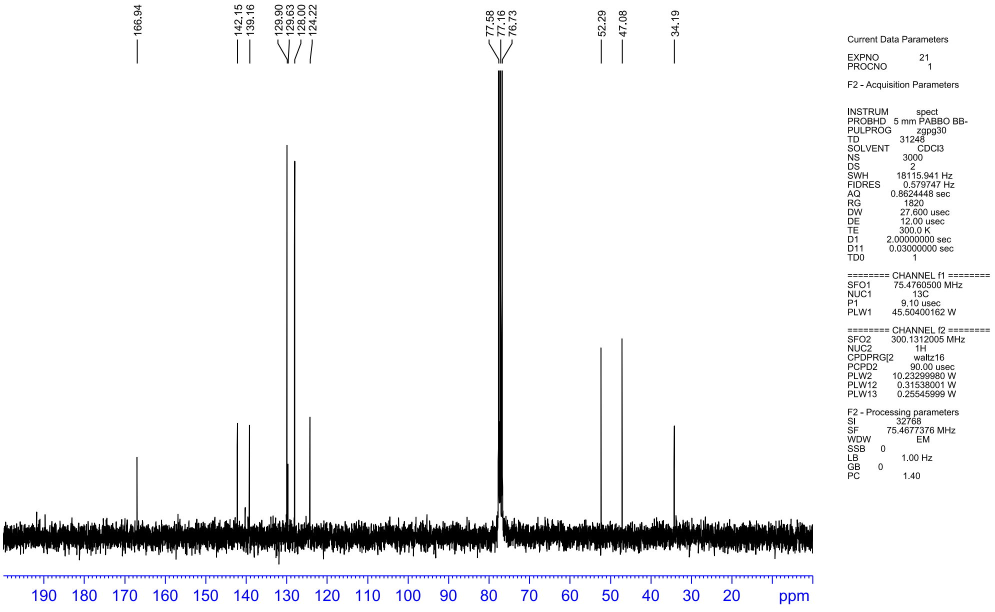

Figure S118: ^{13}C NMR spectrum of **1ah**.

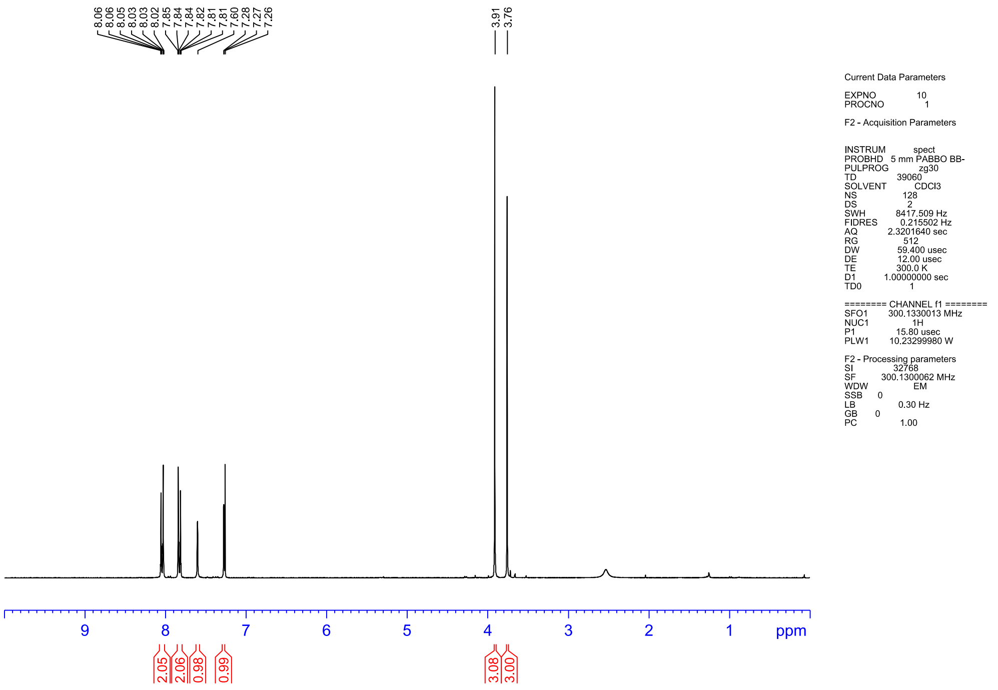

Figure S119: ^{1}H NMR spectrum of **2ah**.

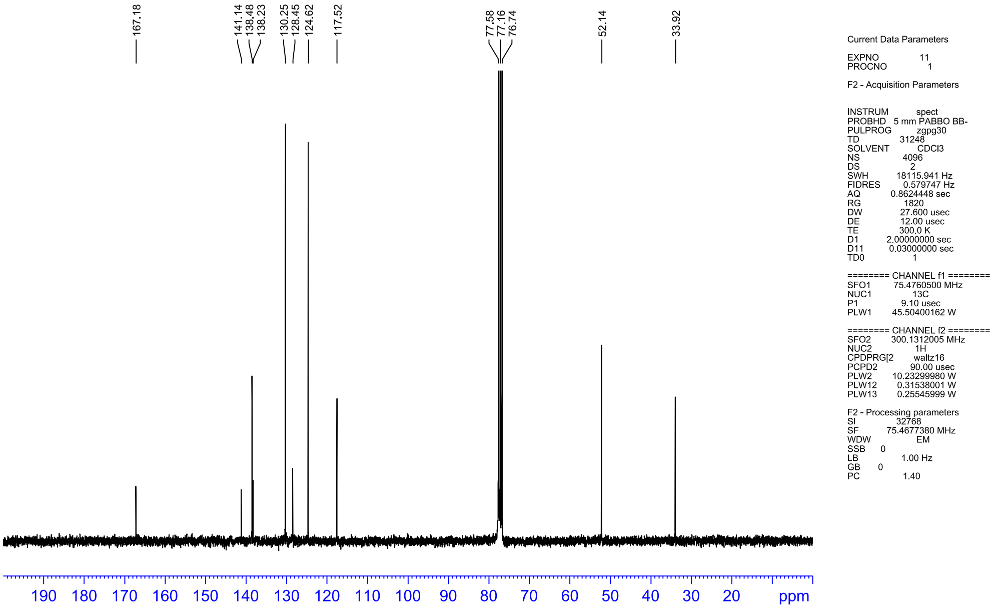

Figure S120: ^{13}C NMR spectrum of **2ah**.

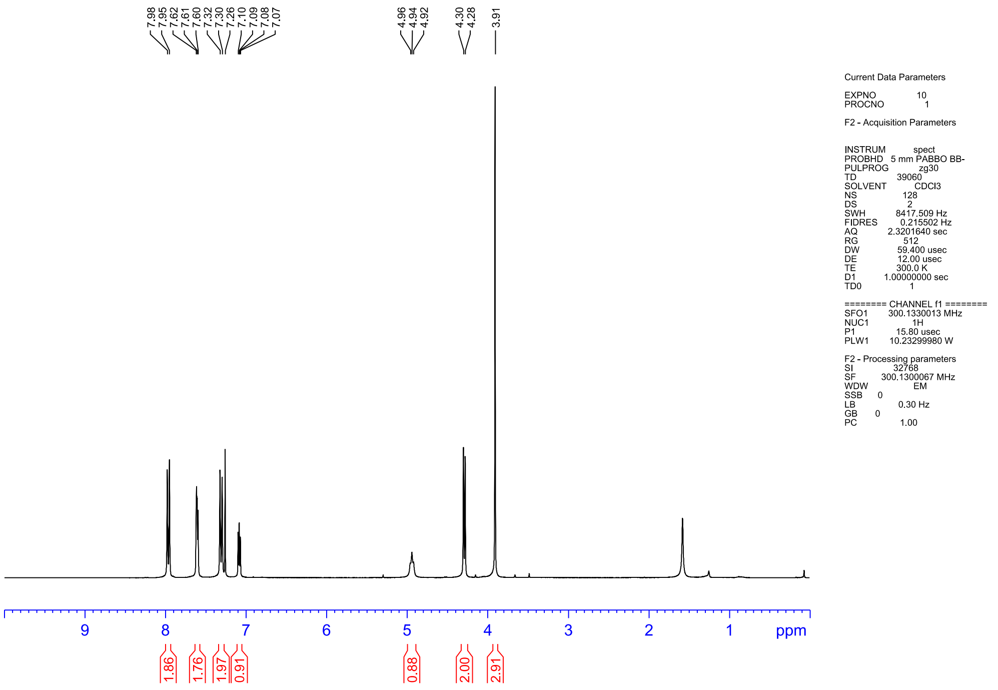

Figure S121: ^{1}H NMR spectrum of **1ai**.

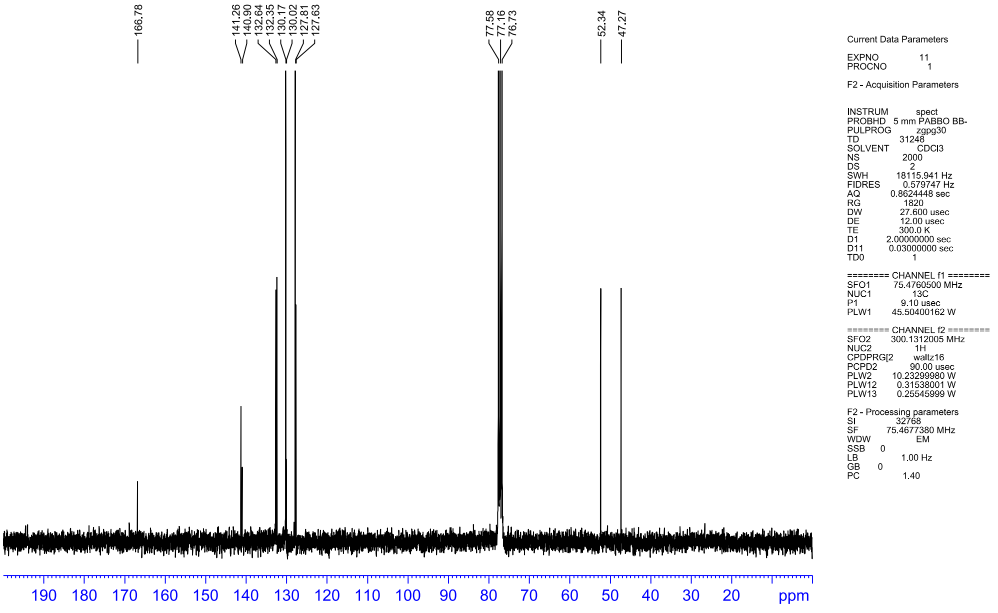

Figure S122: ^{13}C NMR spectrum of **1ai**.

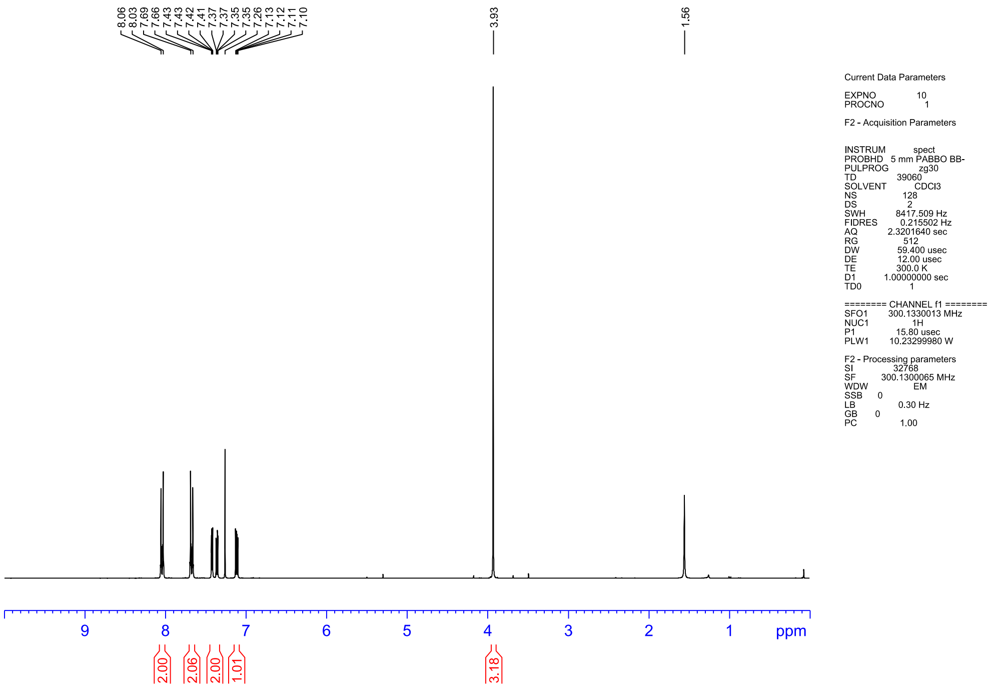

Figure S123: ^{1}H NMR spectrum of **2ai**.

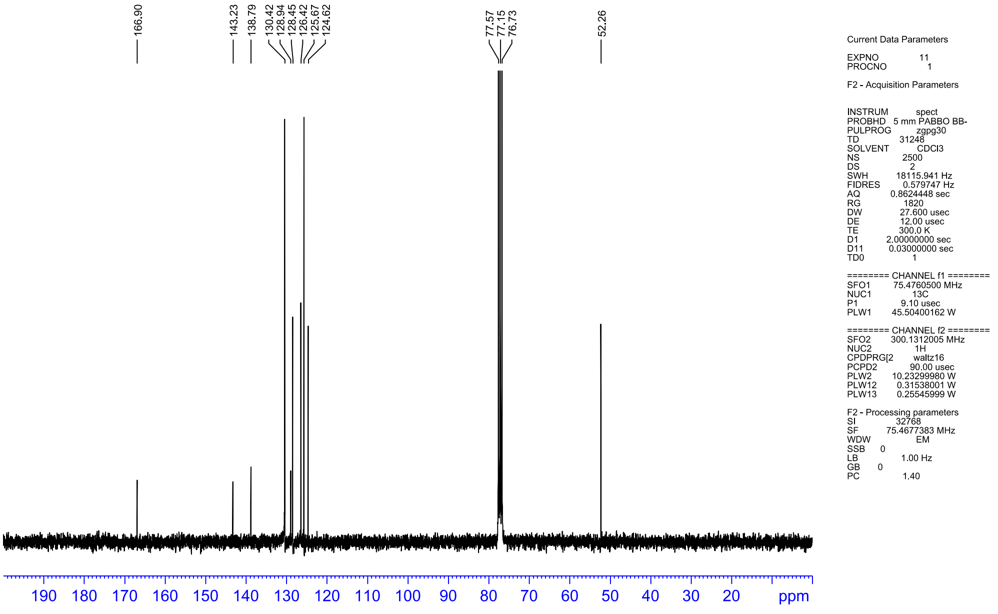

Figure S124: ^{13}C NMR spectrum of **2ai**.

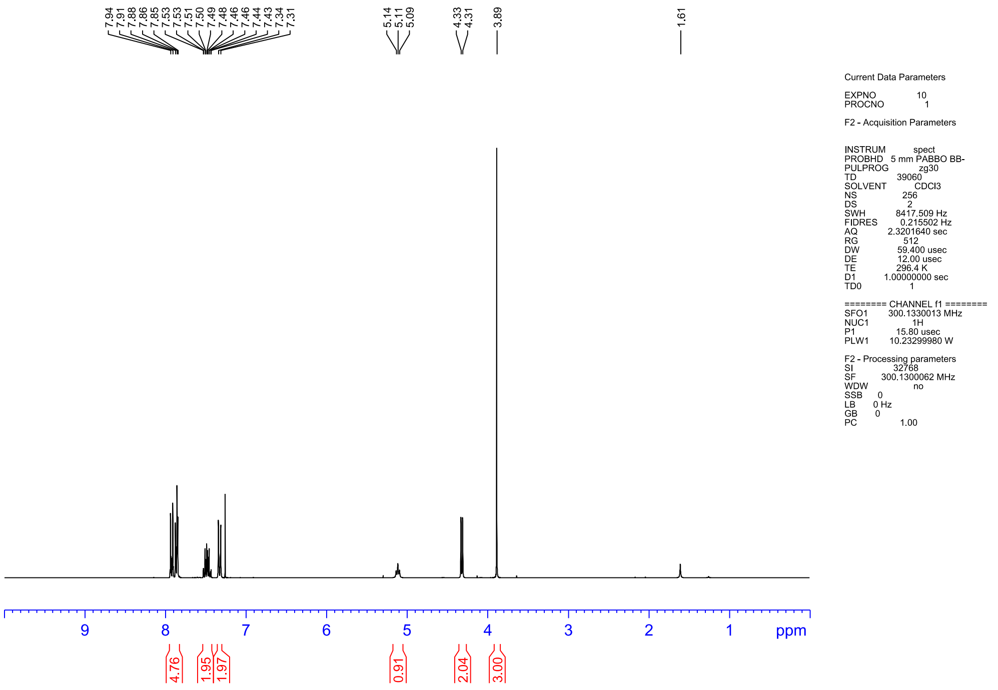

Figure S125: ^{1}H NMR spectrum of **1aj**.

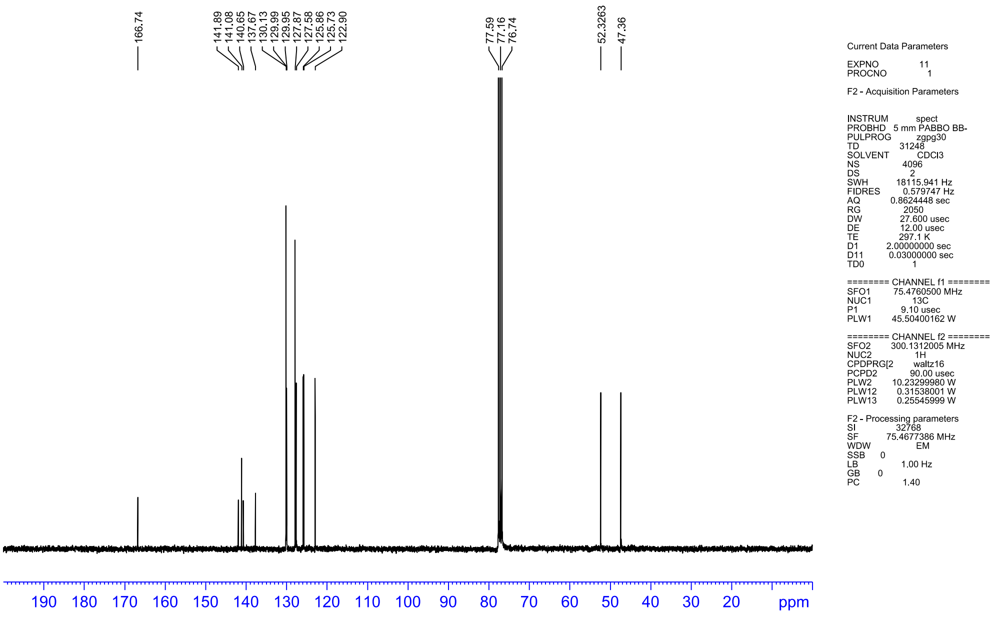

Figure S126: ^{13}C NMR spectrum of **1aj**.

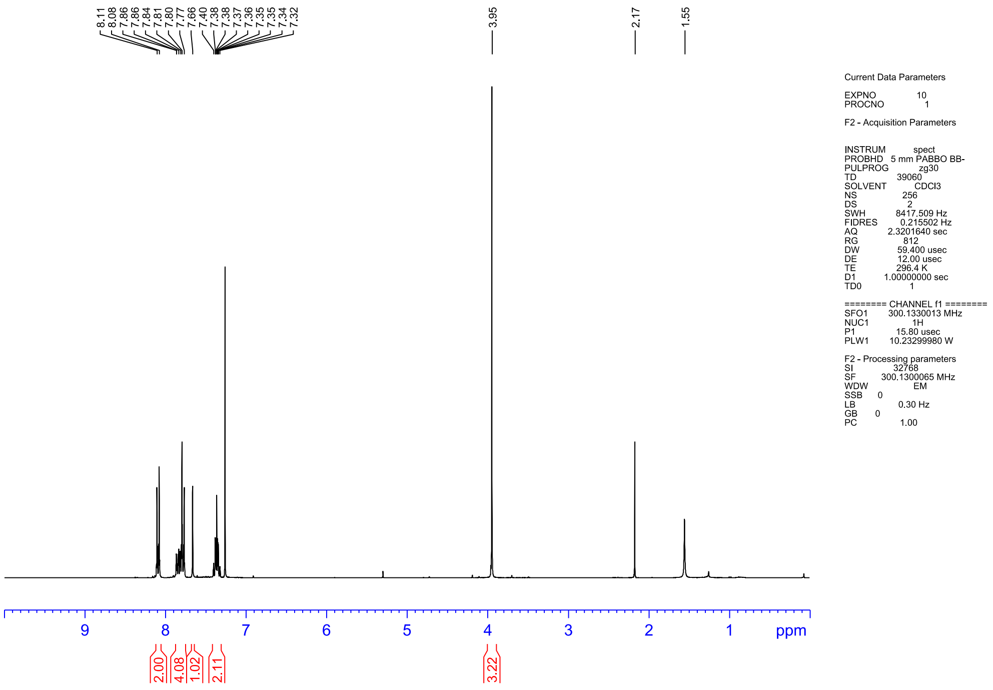

Figure S127: ^{1}H NMR spectrum of **2aj**.

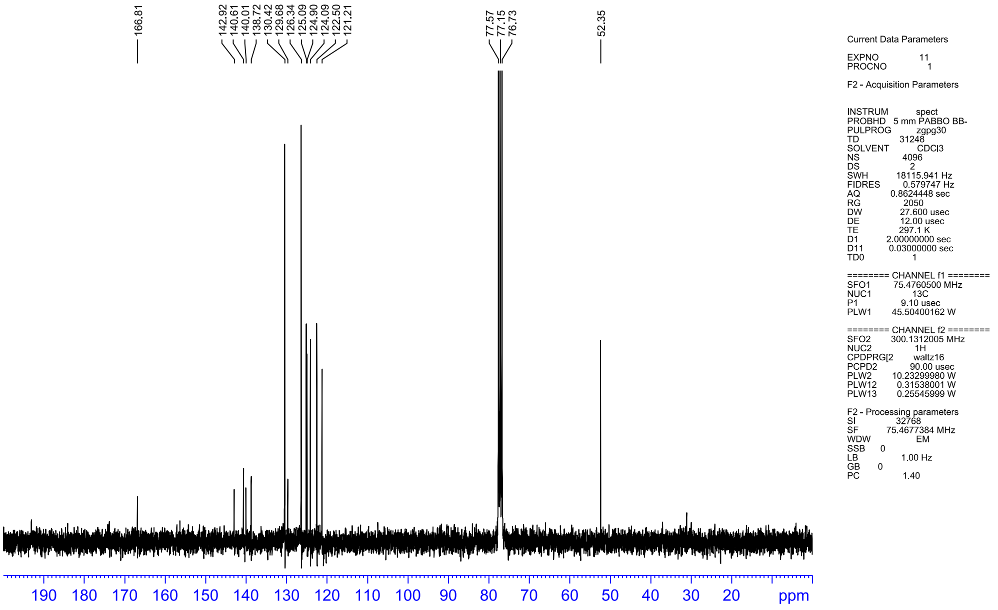

Figure S128: ^{13}C NMR spectrum of **2aj**.

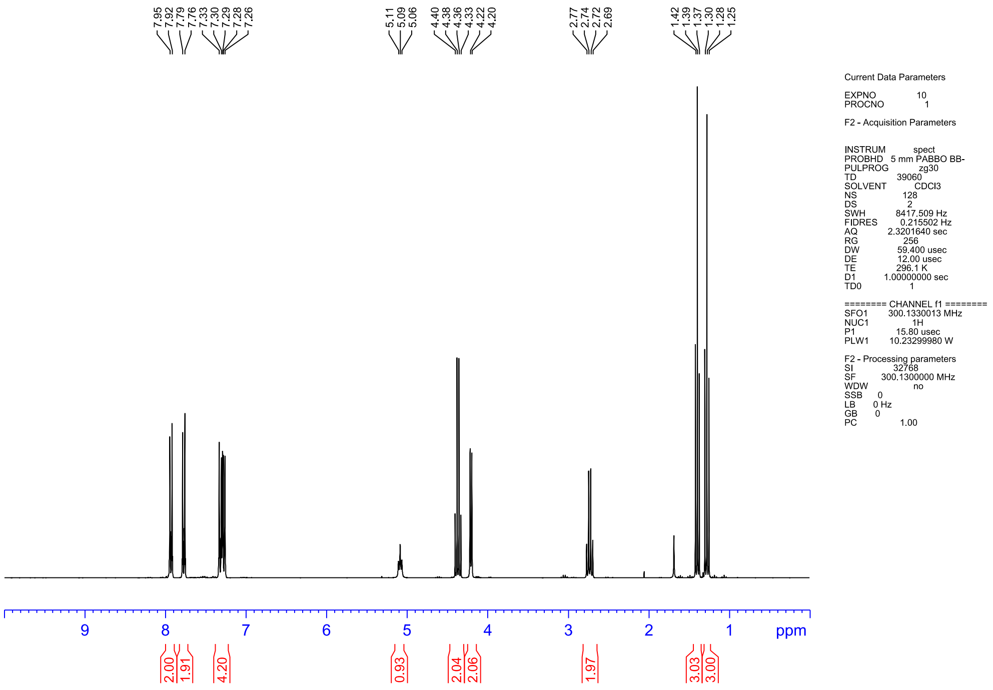

Figure S129: ^{1}H NMR spectrum of **1ak**.

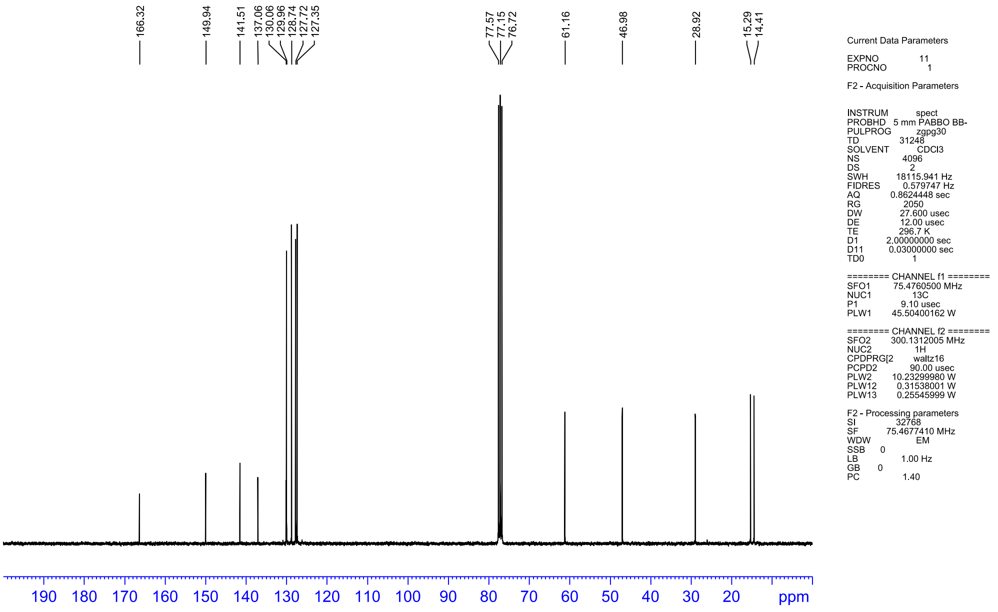

Figure S130: ^{13}C NMR spectrum of **1ak**.

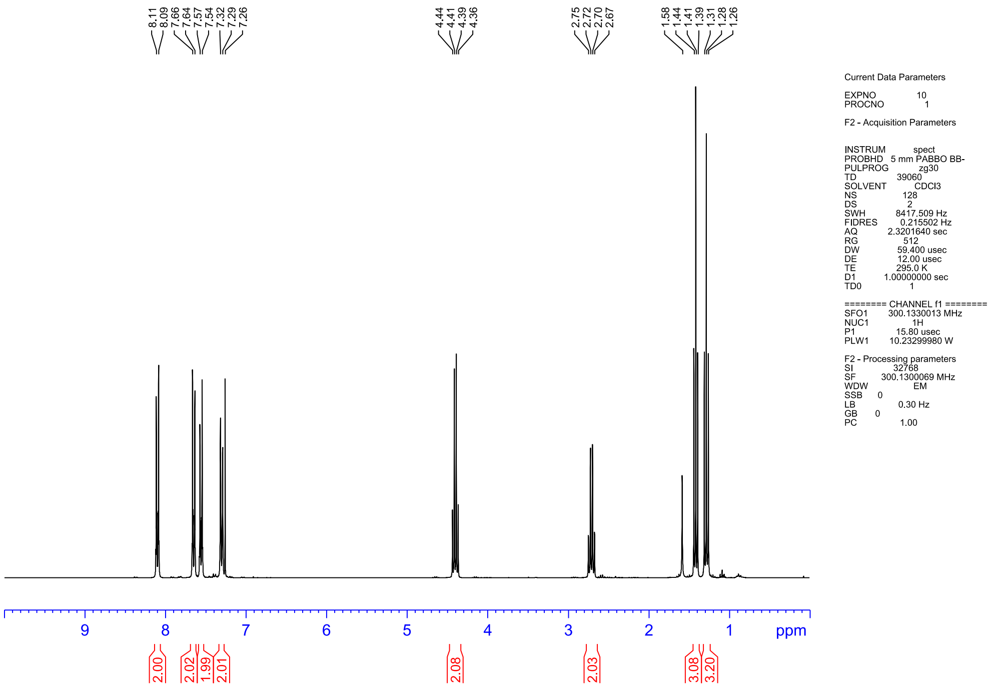

Figure S131: ^{1}H NMR spectrum of **2ak**.

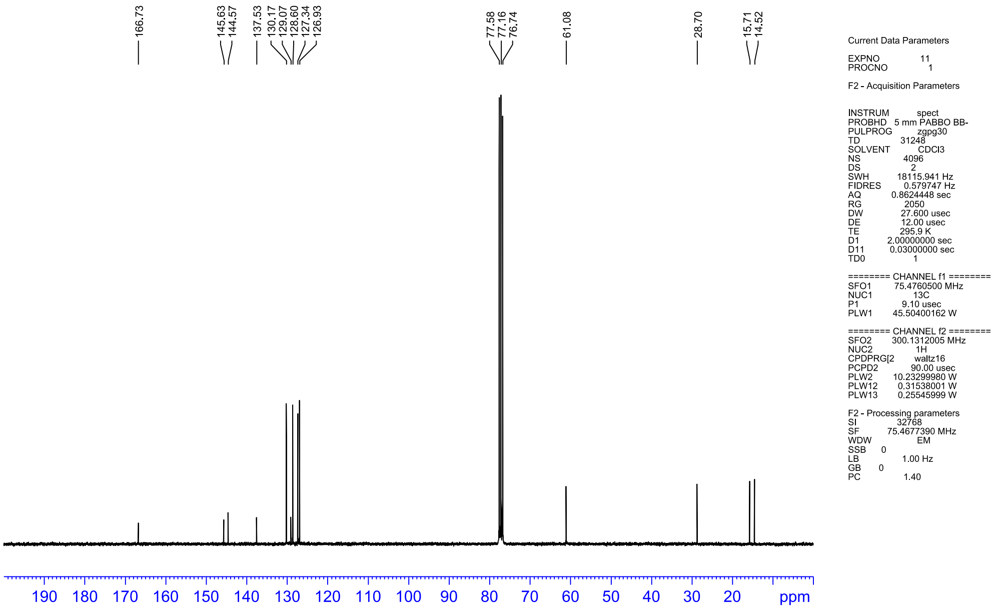

Figure S132: ^{13}C NMR spectrum of **2ak**.

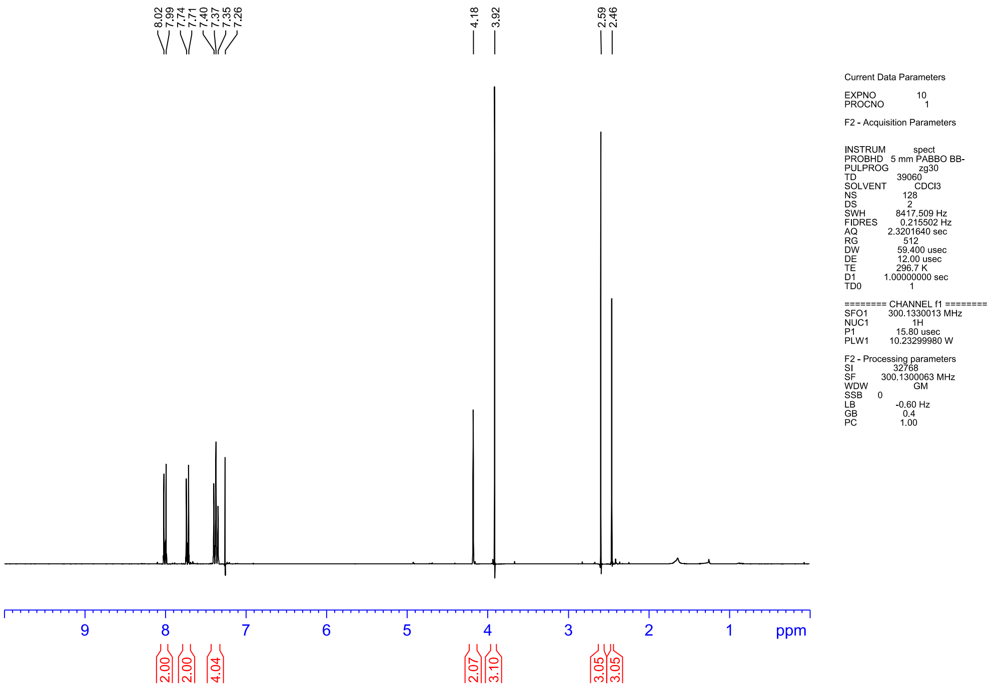

Figure S133: ^{1}H NMR spectrum of **1al**.

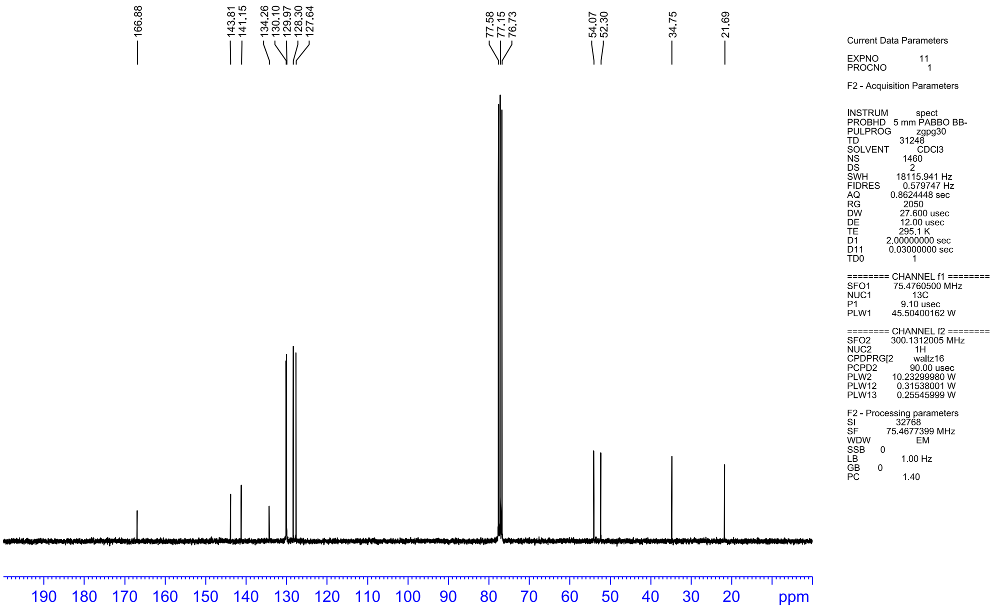

Figure S134: ^{13}C NMR spectrum of **1al**.

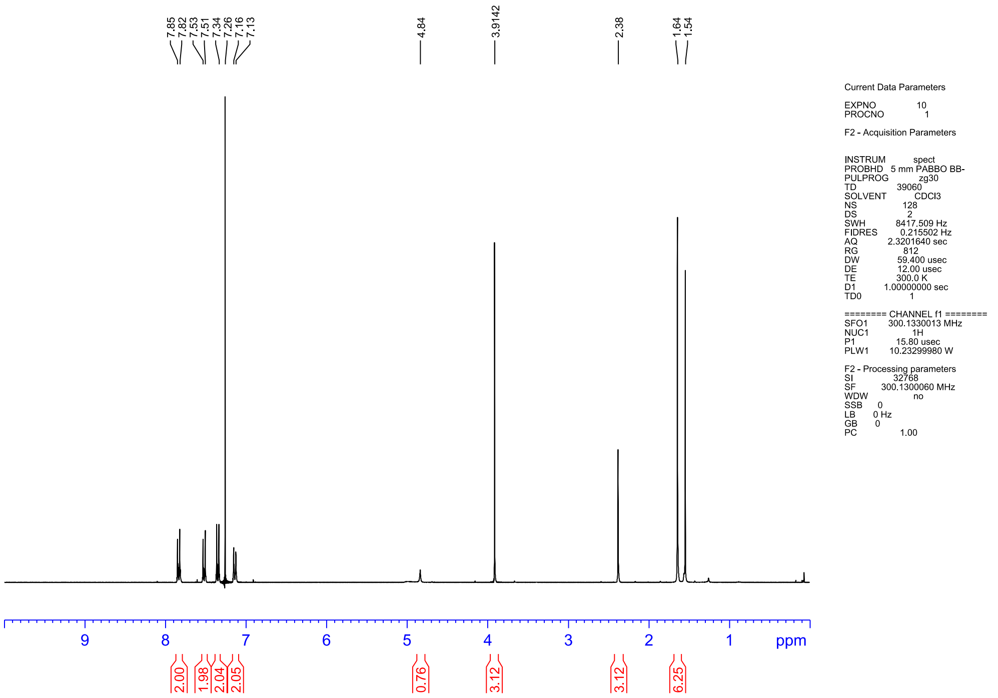

Figure S135: ^{1}H NMR spectrum of **1am**.

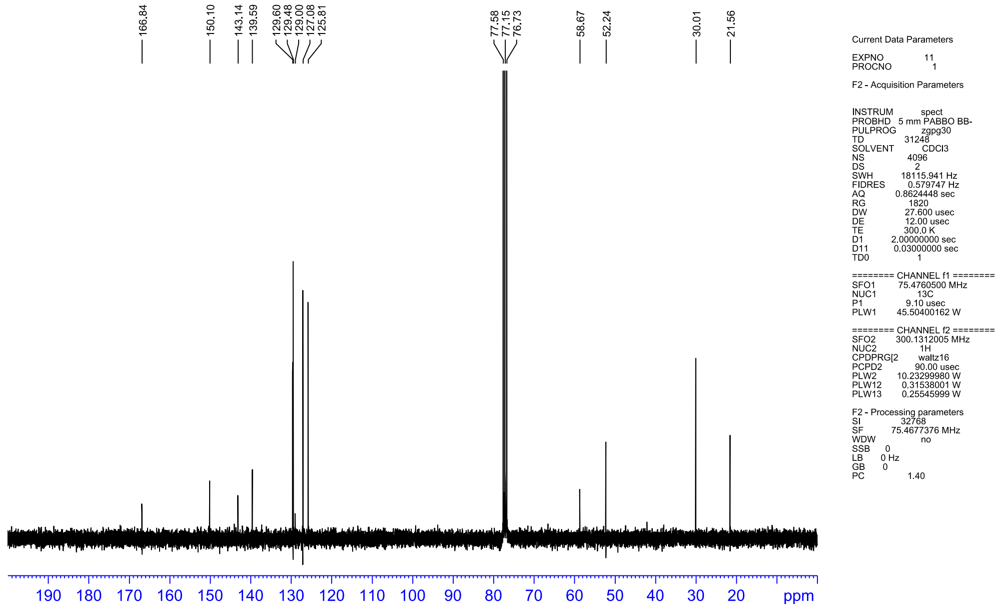

Figure S136: ^{13}C NMR spectrum of **1am**.

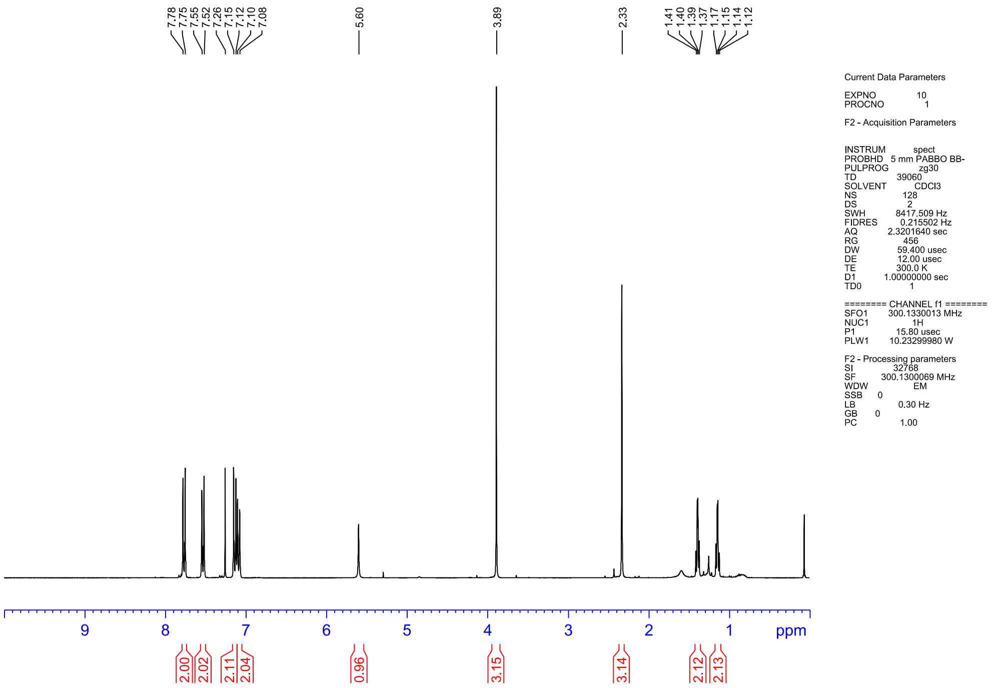

Figure S137: ^{1}H NMR spectrum of **1an**.

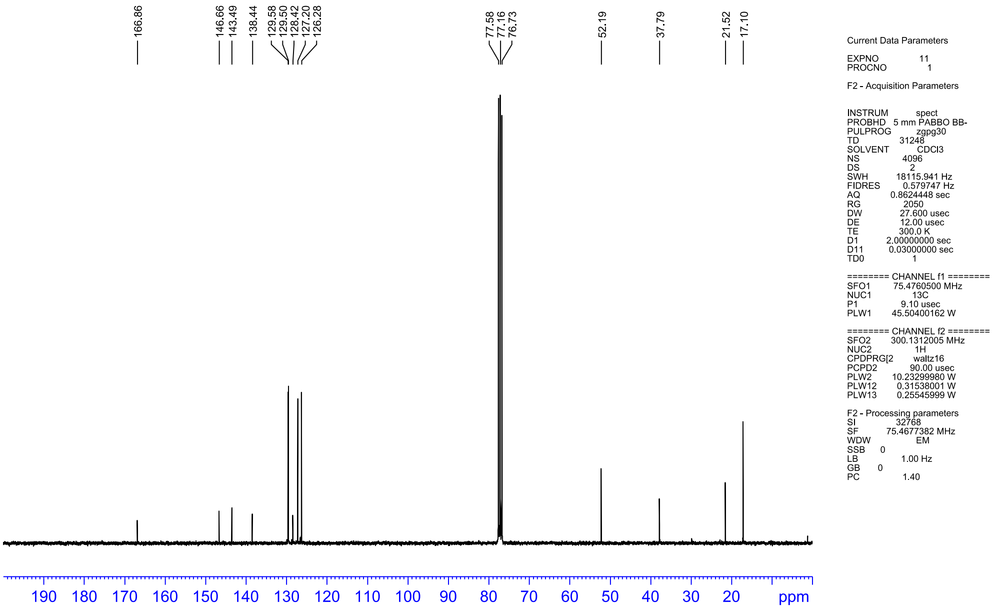

Figure S138: ^{13}C NMR spectrum of **1an**.

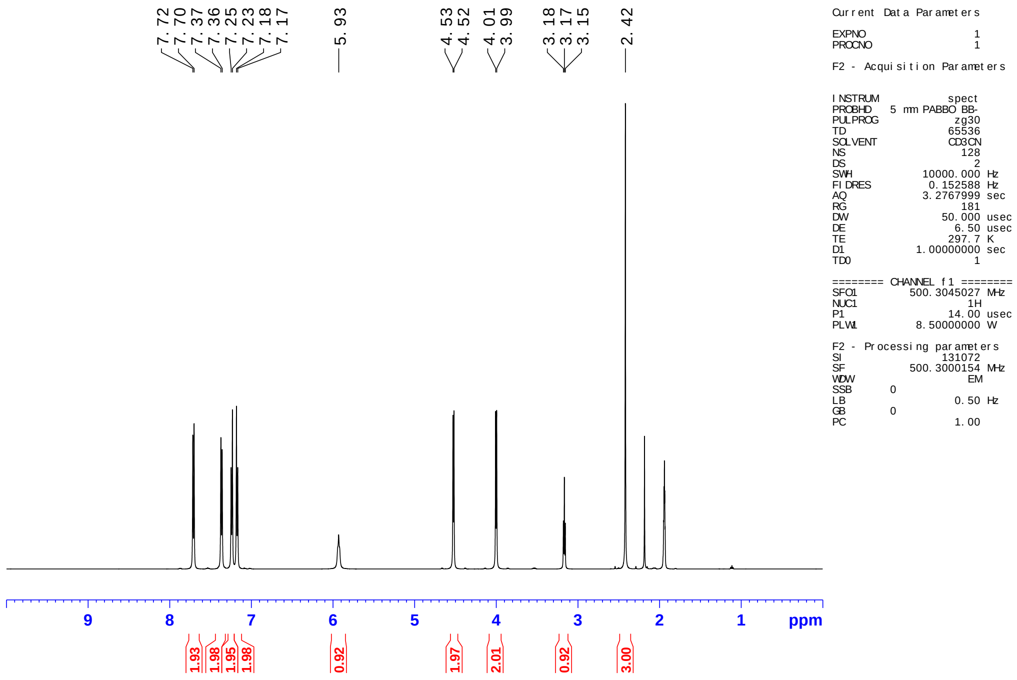

Figure S139: ^{1}H NMR spectrum of **1ao**.

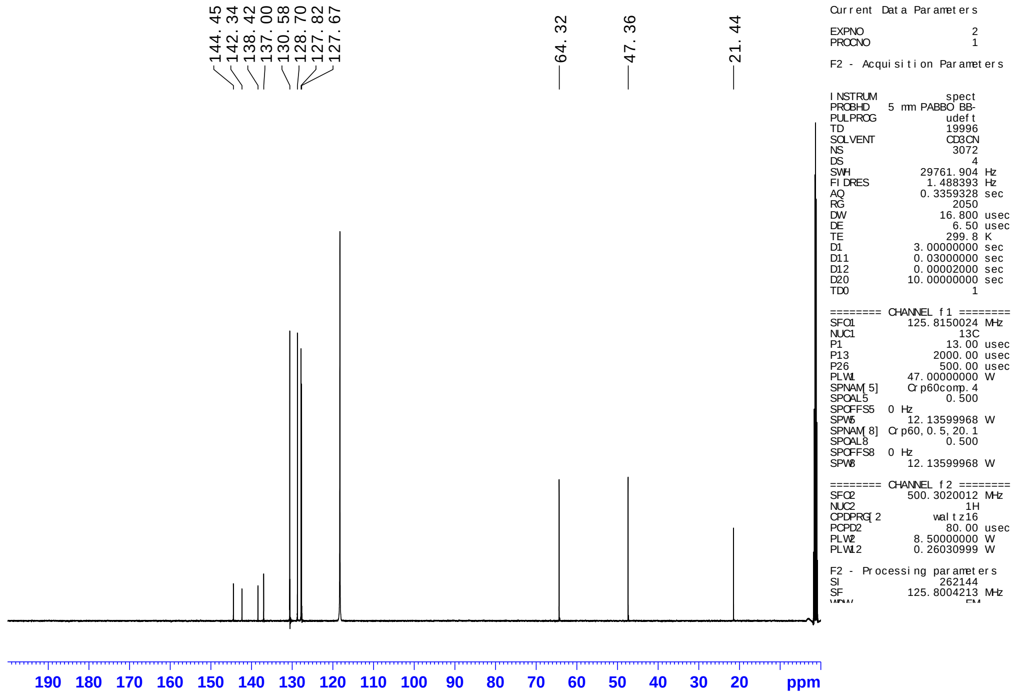

Figure S140: ^{13}C NMR spectrum of **1ao**.
